# Ocellatuspyrones A‒G, new antibacterial polypropionates from the Chinese mollusk *Placobranchus ocellatus*

**DOI:** 10.1007/s42995-023-00179-w

**Published:** 2023-07-06

**Authors:** Song-Wei Li, Dan-Dan Yu, Ming-Zhi Su, Li-Gong Yao, Hong Wang, Xueting Liu, Yue-Wei Guo

**Affiliations:** 1 Collaborative Innovation Center of Yangtze River Delta Region Green Pharmaceuticals, College of Pharmaceutical Science, Zhejiang University of Technology, Hangzhou, 310014 China; 2 State Key Laboratory of Drug Research, Shanghai Institute of Materia Medica, Chinese Academy of Sciences, Shanghai, 201203 China; 3 State Key Laboratory of Bioreactor Engineering, East China University of Science and Technology, Shanghai, 200237 China; 4 Shandong Laboratory of Yantai Drug Discovery, Bohai Rim Advanced Research Institute for Drug Discovery, Yantai, 264117 China

**Keywords:** *Placobranchus ocellatus*, *γ*-pyrone polypropionate, Photosynthetic mollusk, Antibacterial activity, Stereochemistry

## Abstract

Marine invertebrates serve as rich sources of secondary metabolites with intriguing chemical diversities and a wide spectrum of biological activities. Particularly, marine shell-less sacoglossan mollusks have attracted much attentions due to the fact that mollusks apply complex metabolites as chemical defense agents against to their predators. With the purpose of discovering bioactive secondary metabolites to develop marine-derived medicines from the South China Sea, we have conducted a chemical study on the photosynthetic mollusk *Placobranchus ocellatus.* As a result, seven new *γ*-pyrone polypropionates, namely ( ±)-ocellatuspyrone A (**1**), ( ±)-ocellatuspyrone B (**2**), and ocellatuspyrones C−G (**5**, **9**−**12**), along with five known polypropionates, have been isolated and characterized from the South China Sea photosynthetic mollusk *Placobranchus ocellatus*. Extensive spectroscopic analysis, single crystal X-ray diffraction analysis, modified Mosher’s method, ECD comparison, CD exciton chirality method, TDDFT-ECD calculation, and chemical conversion were used to determine the structures and absolute configurations of the new compounds and the stereochemistry of undefined known compounds **4**, **6** and **7**. All these isolated polypropionates were evaluated in bioassays for their biological activities, including antibacterial, neuroprotective effect, anti-inflammatory, PTP1B inhibitory, and antiviral activities. Compounds **7**, **8** and **11** were found for the first time to show antibacterial activity against fish pathogenic bacteria *Streptococcus parauberis* (the main pathogen causing fish streptococcal infections and acute death) with MIC values of 35.8, 34.2, and 37.4 μg/mL, respectively, which might be potential novel antibacterial agents for the treatment of fish infectious diseases.

**Supplementary Information:**

The online version contains supplementary material available at 10.1007/s42995-023-00179-w.

## Introduction

Fish farming has a significant economic market in Asian countries. However, the acute death of fish affected by numerous bacterial (such as *Streptococcus parauberis*) infections are the most important reasons for the economic losses in fish farming business (Park et al. 2018). The prevention and treatment of fish infections mainly rely on antibacterial drugs, such as ampicillin, oxytetracycline, etc., while the frequent use of antibiotics has led to the development of drug resistance in pathogenic bacteria. Therefore, studies related to the exploring novel antibacterial drugs of fish infectious disease are of great value. Sacoglossans are small colorful marine mollusks, and most are characterized by reduced shell or exposed mantle (Cimino 1999). The degeneration of the protective shell forced mollusks to secrete large amounts of possibly toxic mucous to defend against the attack of predators (Bornancin et al. 2017). From this interesting point of view, marine sacoglossan mollusks have long been investigated both chemically and biologically. Many bioactive marine natural products have been isolated from animals of this order (Bornancin et al. 2017; Cimino 1999). Among them, metabolites derived from the condensation of propionate units are characteristic chemical defense compounds of mollusks, which have shown a wide spectrum of biological activities ranging from cytotoxic, ichthyotoxic, antibiotic, antifungal to antiviral properties (Davies-Coleman and Garson 1998; Gavagnin et al. 1994a, b; Liu et al. 2020). In addition, terpenes and alkaloids with feeding deterrent activities and/or color deterrence are also regarded as important defensive chemicals of mollusks (Gavagnin et al. 1994a, b; Mudianta et al. 2014).

Polypropionates are among the most structurally complex polyketides, which represent a large family of typical bioactive natural products, and have been isolated from many species of the order sacoglossa, such as *Elysia chlorotica* (Dawe and Wright 1986), *E. timida*, and *E. viridis* (Marin and Ros 2004). Examples include simple acyclic polypropionates and complicated polypropionates containing furanone, 2-pyrone, and 4-pyrone (*γ*-pyrone) rings (Davies-Coleman and Garson 1998). Previous reports have indicated that polypropionate-derived secondary metabolites containing the *γ*-pyrone moiety exhibited promising in vitro growth-inhibitory activity against human cancer cell lines (Carbone et al. 2013a, b; Rodriguez et al. 1992a, b; Zhou et al. 2018). Moreover, pyrone polypropionates are likely functioning as a natural sunscreen against photochemical damages (Faulkner 2000; Ireland and Scheuer 1979; Manzo et al. 2005; Zuidema et al. 2005) and/or as a scavenger of harmful reactive oxygen species (ROS) (Powell et al. 2018) due to the presence of a polyunsaturated side chain.

More recently, significant attention has been directed towards studying the biosynthesis of these sacolossan polypropionates. Feeding experiments in sacoglossans have shown that mollusks can utilize C_3_ units for the biosynthesis of pyrone polypropionates (Cutignano et al. 2009, 2012; Di Marzo et al. 1991). In addition, some sacoglossans have been proven to prey on algae, digesting the cells but maintaining functional chloroplasts, and incorporating the fixed carbon obtained from de novo chloroplast photosynthesis into polypropionate metabolites using fatty acid synthase-(FAS)-like polyketide synthase (PKS) proteins, which open the door to understanding the metabolic patterns of marine mollusk metabolites (Torres et al. 2020).

**Fig. 1:**
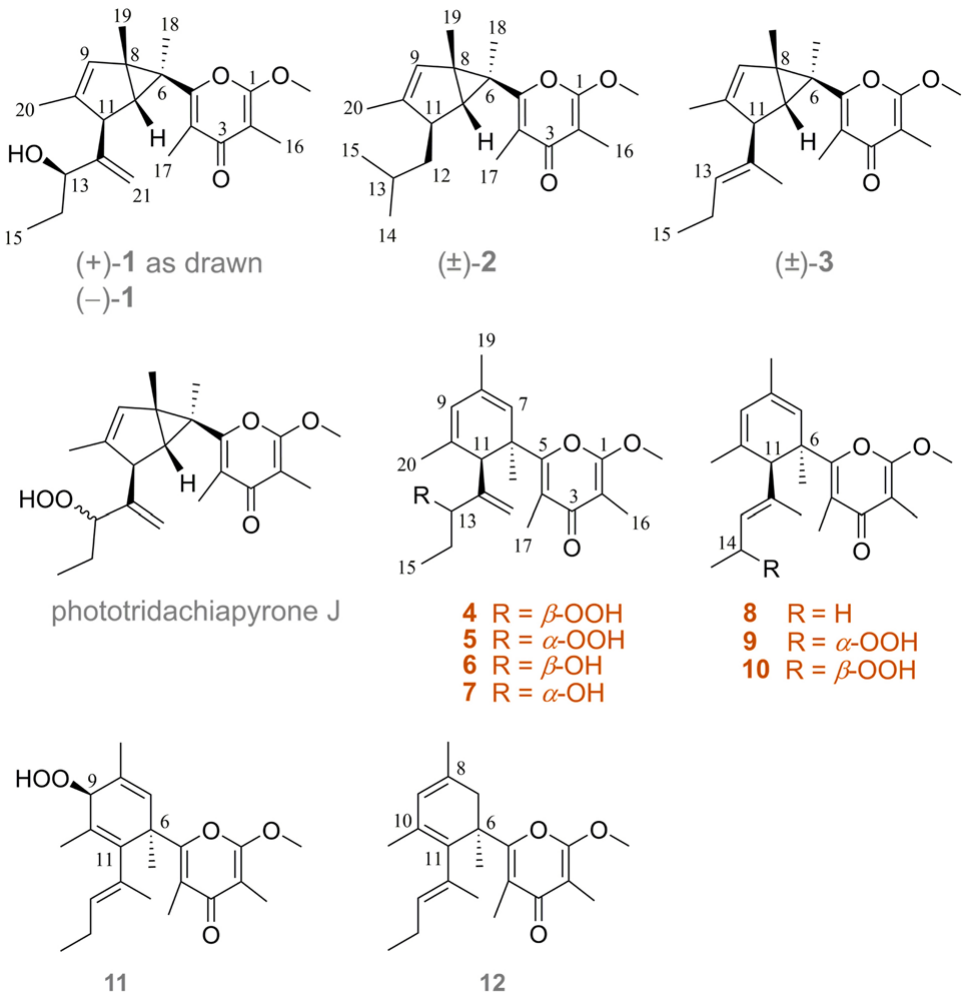
The structures of compounds **1**−**12**

While continuing our research with the purpose of discovering bioactive secondary metabolites from marine mollusks in the South China Sea, we have very recently conducted a chemical study on the photosynthetic mollusk *Placobranchus ocellatus* (phylum Mollusca, class Gastropoda, subclass Opisthobranchia, order Sacoglossa) collected off Ximao Island, Hainan Province, China. We have isolated a series of racemic rearranged *γ*-pyrone polypropionates with novel skeletons, named ocellatusones (Wu et al. 2020), and several racemic endoperoxide-bridged *γ*-pyrone polypropionates, named ocellatuperoxides (Li et al. 2022). In the present study, we describe the results of the following chemical investigation of the titled animals, intending to expand structural diversity and biological activity of the polypropionates, leading to the discovery of seven new *γ*-pyrone polypropionates, including two pairs of enantiomers with a bicyclohexane fragment, namely ( ±)-ocellatuspyrone A (**1**) and ( ±)-ocellatuspyrone B (**2**), and five optically active compounds ocellatuspyrones C−G (**5**, **9**−**12**) (Fig. 1). Herein, we report the isolation, structural elucidation, and biological activity evaluation of these new compounds.

## Results

The frozen bodies of *P. ocellatus* (500 specimens, 55.0 g, dry weight) collected off shallow water of Ximao Island, Hainan Province, China, were extracted with MeOH−CH_2_Cl_2_ (1:1). Twelve polypropionates (**1**−**12**) were isolated from the Et_2_O-soluble portion, and the structures of known compounds were clearly identified as ( ±)-photodeoxytridachione (**3**), tridachiapyrone J (**4**), tridachiapyrone G (**6**), tridachiapyrone H (**7**), and ( +)-9,10-deoxytridachione (**8**), the previously reported polypropionates isolated from the Philippine sacoglossan mollusk *P. ocellatus* (Fu et al. 2000) and Mediterranean sacoglossan mollusk *Elysia timida* (Gavagnin et al. 1994a, b), by comparing the NMR data (Supplementary Figs. S10a−S14c) and specific rotation values with those of reported in the literatures.

( ±)-Ocellatuspyrone A (**1**) was isolated as a colorless oil, and its molecular formula was determined to be C_22_H_30_O_4_ by the ion peak at *m/z* 359.2210 [M + H]^+^ (calcd. for C_22_H_31_O_4_, 359.2217) in the high-resolution (HR) ESI–MS, indicating eight degrees of unsaturation. The IR spectrum (KBr, *ν* = 3395, 1658, 1584 cm^−1^) indicated the presence of hydroxyl and unsaturated carbonyl groups. The ^1^H and ^13^C NMR (Tables 1 and 2), together with HMBC and HSQC spectra (Supplementary Figs. S1g and S1h) disclosed 22 carbon signals, including seven methyls, one sp^3^ methylene, three sp^3^ methines, two sp^3^ quaternary carbons, one sp^2^ methylene, one sp^2^ methine, and seven sp^2^ quaternary carbons. The characteristic NMR data of a ketone carbonyl (*δ*_C_ 181.6, qC), two tetrasubstituted double bonds (*δ*_C_ 99.8, qC, *δ*_C_ 162.7, qC; *δ*_C_ 120.5, qC, 160.2, qC), two methyls (*δ*_C_ 7.1, CH_3_-16; *δ*_C_ 11.0, CH_3_-17), and one methoxyl (*δ*_C_ 55.4, −OMe) identified the existence of a typical tetrasubstituted *γ*-pyrone moiety in **1**, which was supported by UV spectrum (MeOH, *λ*_max_ = 257 nm, log*ε* = 2.8). In addition, one trisubstituted double bond (*δ*_C_ 144.5, qC, *δ*_C_ 129.6, CH) and one disubstituted double bond (*δ*_C_ 151.9, qC, *δ*_C_ 113.5, CH_2_) were also observed in the ^1^H and ^13^C spectroscopic data of **1**. The above data accounted for six degrees of unsaturation, suggesting that **1** has another bicyclic structure. Furthermore, the remaining NMR signals indicated the presence of three singlet methyls (*δ*_H_ 1.14, s, *δ*_C_ 13.3, CH_3_-18; *δ*_H_ 1.20, s, *δ*_C_ 17.5, CH_3_-19; *δ*_H_ 1.64, s, *δ*_C_ 14.0, CH_3_-20), one triplet methyl (*δ*_H_ 0.96, t, *J* = 7.4 Hz, *δ*_C_ 10.4, CH_3_-15), one methylene (*δ*_H_ 1.68, m, 2H, *δ*_C_ 28.5), and one oxygenated methine (*δ*_H_ 4.08, t, *J* = 6.4 Hz, *δ*_C_ 76.8). These data suggested that **1** was a *γ*-pyrone polypropionate.

**Table 1:** ^1^H NMR data of compounds **1**−**5**, and **9**−**12** in CDCl_3_

No.	*δ*_H_ mult (*J* in Hz)								
	**1**	**2**	**3**	**4**	**5**	**9**	**10**	**11**	**12**
7	1.25, br s	1.25, br s	1.43, br s	5.53, s	5.47, s	5.61, s	5.63, s	5.68, s	2.84, d (17.9)
									1.99, d (17.9)
9	5.42, s	5.22, s	5.33, s	5.71, s	5.73, s	5.72, s	5.72, s	4.47, s	5.59, s
11	2.90, br s	2.19, d (10.8)	2.73, br s	3.06, s	2,58	2.78, s	2.81, s		
12		1.50, m							
		1.21, m							
13	4.08, t (6.4)	1.75, m	5.30, t (7.3)	5.53, br s	3.58, br s	5.06, d (8.2)	5.03, d (8.6)	4.92, br s	5.13, t (7.3)
14	1.68, m	0.97, d (6.6)	2.04, dq (2.6, 7.5)	1.65, m	1.68, m	4.57, dq (6.4, 8.2)	4.57, dq (6.3, 8.6)	1.96, q (7.4)	1.93, m
	1.68, m		2.04, dq (2.6, 7.5)	1.32, m	1.38, m			1.96, q (7.4)	1.93, m
15	0.96, t (7.4)	0.95, d (6.6)	0.97, t, (7.5)	0.72, t (7.3)	0.88, t (7.3)	1.09, d (6.4)	0.89, d (6.3)	0.84, t (7.4)	0.84, t (7.5)
16	1.84, s	1.85, s	1.84, s	1.84, s	1.93, s	1.88, s	1.81, s	1.84, s	1.84, s
17	1.97, s	1.98, s	1.97, s	1.98, s	2.07, s	2.10, s	2.09, s	1.77, s	1.98, s
18	1.14, s	1.07, s	1.10, s	1.49, s	1.52, s	1.48, s	1.46, s	1.51, s	1.53, s
19	1.20, s	1.18, s	1.19, s	1.81, s	1.82, s	1.80, s	1.79, s	1.90, s	1.77, s
20	1.64, s	1.68, s	1.57, s	1.77, s	1.73, s	1.73, s	1.75, s	1.79, s	1.72, s
21	5.18, s		1.49, s	5.19, s	5.20, s	1.45, s	1.46, s	1.57, br s	1.51, s
	4.90, s			5.12, s	5.17, s				
OMe	3.95, s	3.96, s	3.96, s	4.02, s	4.03, s	4.03, s	3.99, s	3.93, s	3.95, s

^1^H NMR at 600 MHz, values are reported in ppm referenced to CHCl_3_ (*δ*_H_ 7.26). Assignments were aided by HSQC and HMBC experiments

**Table 2:** ^13^C NMR data of compounds **1−5**, and **9−12** in CDCl_3_

No.	*δ*_C_, type								
	**1**	**2**	**3**	**4**	**5**	**9**	**10**	**11**	**12**
1	162.7, C	162.5, C	162.5, C	162.1, C	162.0, C	162.2, C	161.8, C	161.8, C	161.9, C
2	99.8, C	99.5, C	99.6, C	99.4, C	99.4, C	99.4, C	98.9, C	99.5, C	99.1, C
3	181.6, C	181.8, C	181.8, C	181.7, C	182.3, C	182.1, C	181.8, C	181.3, C	181.8, C
4	120.5, C	120.5, C	120.5, C	120.5, C	121.3, C	120.4, C	120.1, C	121.2, C	120.1, C
5	160.2, C	160.9, C	160.7, C	160.7, C	160.0, C	161.1, C	160.7, C	158.0, C	163.1, C
6	41.2, C	40.4, C	40.8, C	47.6, C	48.0, C	47.6, C	47.6, C	47.2, C	45.6, C
7	29.6, CH	29.6, CH	36.9, CH	123.9, CH	124.4, CH	124.1, CH	124.2, CH	133.6, CH	43.2, CH_2_
8	32.8, C	31.6, C	31.9, C	128.3, C	128.1, C	128.2, C	128.0, C	128.7, C	132.0, C
9	129.6, CH	127.3, CH	128.8, CH	122.7, CH	122.8, CH	123.1, CH	123.3, CH	84.0, CH	123.6, CH
10	144.5, C	146.1, C	144.2, C	136.2, C	135.7, C	134.2, C	133.7, C	125.3, C	126.0, C
11	49.9, CH	46.7, CH	58.5, CH	53.2, CH	52.4, CH	59.3, CH	59.6, C	143.3, C	136.3, C
12	151.9, C	42.0, CH_2_	134.1, C	Not detected	143.6, C	138.1, C	138.7, C	131.9, C	132.1, C
13	76.8, CH	25.9, CH	128.9, CH	87.9, CH	88.1, CH	129.6, CH	129.2, CH	132.6, CH	133.0, C
14	28.5, CH_2_	24.2, CH_3_	21.4, CH_2_	Not detected	25.2, CH_2_	77.5, CH	77.9, CH	21.4, CH_2_	21.4, CH_2_
15	10.4, CH_3_	21.7, CH_3_	14.4, CH_3_	10.7, CH_3_	9.8, CH_3_	18.4, CH_3_	18.2, CH_3_	13.9, CH_3_	14.0, CH_3_
16	7.1, CH_3_	7.0, CH_3_	7.0, CH_3_	7.0, CH_3_	7.1, CH_3_	7.1, CH_3_	6.9, CH_3_	7.1, CH_3_	7.0, CH_3_
17	11.0, CH_3_	10.9, CH_3_	10.9, CH_3_	12.5, CH_3_	12.6, CH_3_	12.6, CH_3_	12.4, CH_3_	9.2, CH_3_	13.1, CH_3_
18	13.3, CH_3_	13.1, CH_3_	13.2, CH_3_	26.3, CH_3_	26.9, CH_3_	27.0, CH_3_	27.1, CH_3_	25.1, CH_3_	24.0, CH_3_
19	17.5, CH_3_	17.7, CH_3_	17.3, CH_3_	21.5, CH_3_	21.4, CH_3_	21.7, CH_3_	21.7, CH_3_	19.6, CH_3_	23.1, CH_3_
20	14.0, CH_3_	13.7, CH_3_	13.8, CH_3_	22.4, CH_3_	22.5, CH_3_	22.4, CH_3_	22.5, CH_3_	16.8, CH_3_	20.4, CH_3_
21	113.5, CH_2_		12.9, CH_3_	116.8, CH_2_	114.3, CH_2_	14.9, CH_3_	13.5, CH_3_	17.5, CH_3_	17.0, CH_3_
OMe	55.4, CH_3_	55.3, CH_3_	55.4, CH_3_	55.8, CH_3_	55.7, CH_3_	55.8, CH_3_	55.6, CH_3_	55.5, CH_3_	55.5, CH_3_

^13^C NMR at 150 MHz, values are reported in ppm referenced to CDCl_3_ (*δ*_C_ 77.16). Assignments were aided by HSQC and HMBC experiments

**Fig. 2:**
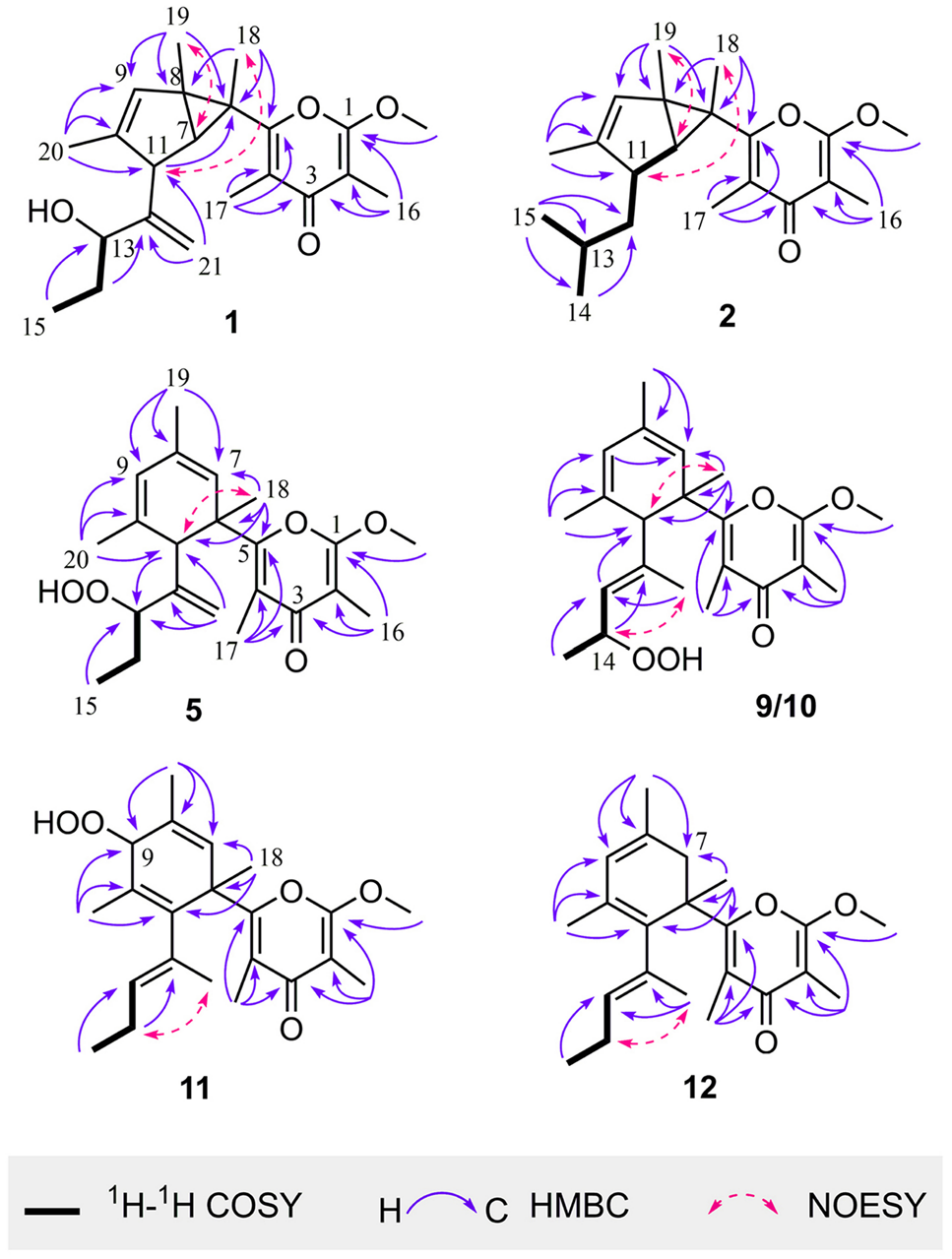
^1^H−^1^H COSY, key HMBC and NOESY correlations for new compounds

A detailed comparison of the NMR data of **1** with those of the co-occurring known polypropionate photodeoxytridachione (**3**) and the model compound phototridachiapyrone J, a known compound isolated previously from the Argentina sacoglossan *Elysia patagonica* (Carbone et al. 2013a, b), revealed that they were structural analogues with the only difference being the presence of a hydroxyl group at C-13 in **1**, in agreement with the mass data and the chemical shift of C-13 at *δ*_C_ 76.8. The hydroxyl group was connected to C-13, as evidenced by the observation of ^1^H−^1^H COSY cross peaks of H-13 (*δ*_H_ 4.08) with both H_2_-14 (*δ*_H_ 1.68) and the HMBC correlation (Fig. 2) from H_3_-15 (*δ*_H_ 0.96) to C-13. The geometry of the double bond at Δ^9(10)^ was assigned to be *Z* by the NOESY correlation of H_3_-20 (*δ*_H_ 1.64) with H-9 (*δ*_H_ 5.42). The relative configurations of C-6, C-7, C-8, and C-11 in **1** were proven to be the same as those of **3** and phototridachiapyrone J due to the similar carbon chemical shifts, which were further supported by the observed NOESY correlations (Fig. 2) of H_3_-18 (*δ*_H_ 1.14) with H-11 (*δ*_H_ 2.90) and of H_3_-19 (*δ*_H_ 1.20) with H-7 (*δ*_H_ 1.25). Considering that compound **1** contains a chiral center C-13 at the rotatable side chain, QM-NMR calculations (Lodewyk et al. 2012) were applied to assign the relative configuration of all the chiral carbons on this molecule to give two possible diastereoisomers (Supplementary Fig. S15a). Geometrical optimization at the DFT level was performed following the DP4 + protocol (Grimblat et al. 2015) using the B3LYP functional with the 6-31G* basis set, followed by NMR calculations at the PCM/mPW1PW91/6-31G(d) level. The experimental NMR data of compound **1** gave the best match for the isomer 6S*, 7R*, 8S*, 11R*, 13R* (**1a)** with over 99% probability (Supplementary Fig. S15c).

**Fig. 3:**
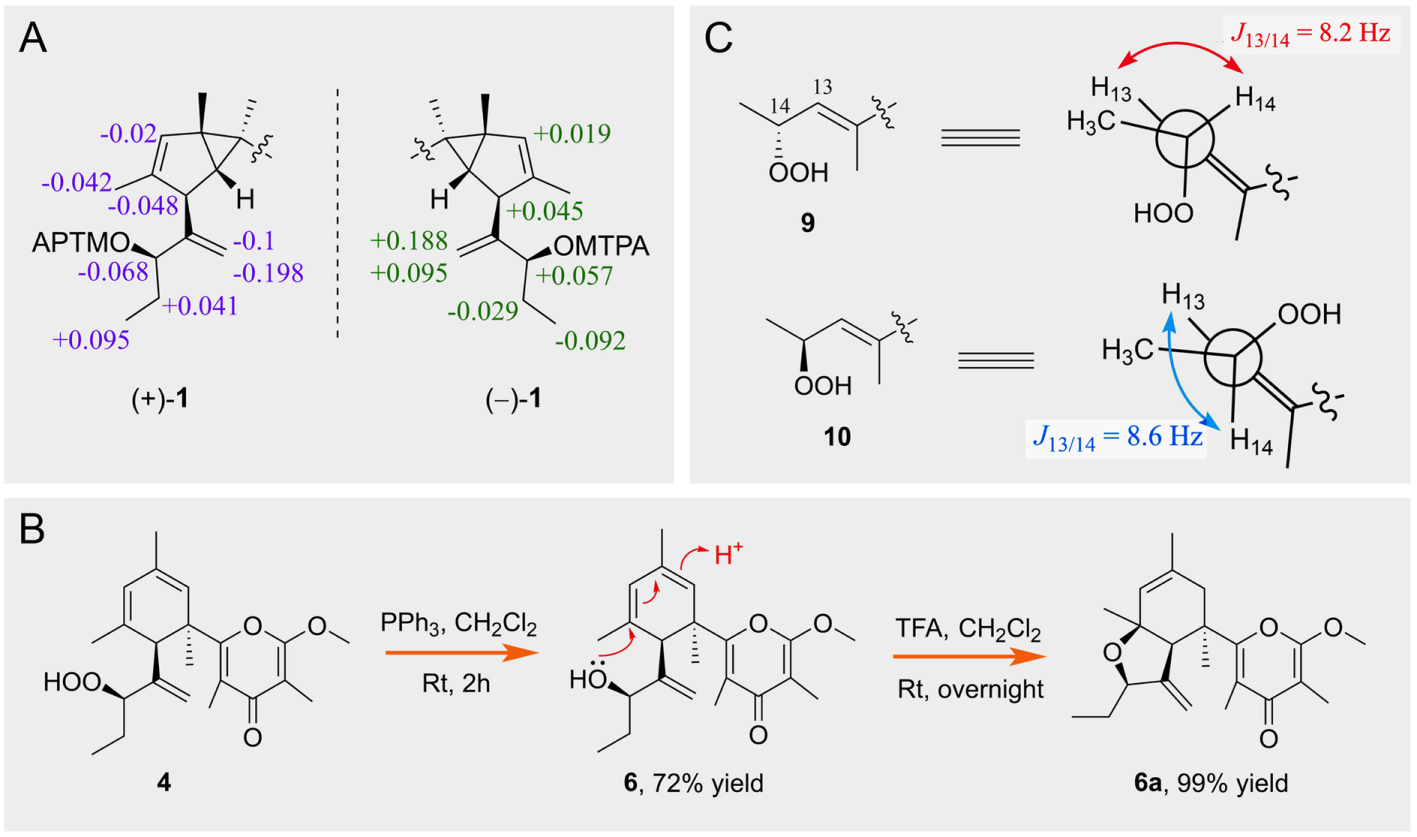
**A** The absolute configurations determination of compounds ( ±)-**1**. **B** Chemical conversion from **4** to **6** and **6a**. **C** Newman projections of the dominant conformation of **9** and **10**, and the observed different *J*_13/14_ values for the two 14-epimers relative to the coupled protons H-13 and H-14

Subsequently, the racemic isolates ( ±)-**1** were successfully separated by chiral HPLC to yield a pair of optically pure enantiomers. The absolute configurations at C-13 of ( ±)-**1** were both determined by the modified Mosher’s method (Li et al. 2018). Esterification of ( ±)-**1** with (*R*)- and (*S*)-MTPA chloride occurred at the C-13 hydroxyl group to give the (*S*)- and (*R*)-MTPA ester derivatives, respectively. The observed Δ*δ*_H(*S*-*R*)_ value distribution pattern (Fig. 3A) established the 13*R*-configuration for ( +)-**1** and 13*S*-configuration for (−)-**1**, respectively. Finally, the structures of ( ±)-**1** were characterized as shown in Fig. 1.

( ±)-Ocellatuspyrone B (**2**), which was isolated as a colorless oil, gave the molecular formula C_21_H_30_O_3_ on the basis of the HR-ESIMS ion peak at *m/z* 331.2272 [M + H]^+^ (calcd. for C_21_H_31_O_3_, 331.2268), requiring seven degrees of unsaturation. The IR spectrum (KBr) displayed the obvious absorption at 1662 and 1602 cm^−1^, consistent with the presence of an unsaturated carbonyl group. The ^1^H and ^13^C NMR data (Tables 1 and 2) of **2** were nearly identical to those of **3**, with exception of an isobutyl group at C-11 in **2** instead of the 1-methyl-1-butenyl group in **3**. This replacement caused the ^13^C NMR resonance of C-11 to be shifted upfield from *δ*_C_ 58.5 to *δ*_C_ 46.7. The position of the isobutyl group at C-11 was further confirmed by the ^1^H−^1^H COSY cross peaks (Fig. 2) from H-11 (*δ*_H_ 2.19) to H_3_-15 (*δ*_H_ 0.95). A detailed analysis of the HMBC correlations from CH_3_-15 to C-12, C-13, and C-14; CH_3_-16 to C-1, C-2, and C-3; CH_3_-17 to C-3, C-4, and C-5; CH_3_-18 to C-5, C-6, and C-8; CH_3_-19 to C-6, C-8, and C-9; CH_3_-20 to C-9, C-10, and C-11; −OCH_3_ to C-1, established the planar structure of **2** (Fig. 2). The geometry of the double bond at Δ^9(10)^ was assigned to be *Z* by the NOESY correlation of H_3_-20 (*δ*_H_ 1.68) with H-9 (*δ*_H_ 5.22). The similar NOESY correlation patterns of **2** and **1**, indicated that they have the same relative configuration. Unfortunately, the absolute configurations of ( ±)-**2** were only tentatively assumed to be the same as ( ±)-**1** base on biogenetic grounds due to the failed attempts to separate ( ±)-**2** by chiral HPLC.

Ocellatuspyrone C (**5**) was obtained as an optically active colorless oil {[*α*] + 83.3 (*c* 0.02 CHCl_3_)}. Its molecular formula of C_22_H_30_O_5_ was deduced from the HR-ESIMS ion peak at *m/z* 375.2169 [M + H]^+^ (calcd. for C_22_H_31_O_5_, 375.2166), suggesting that **5** possessed eight degrees of unsaturation. Its IR spectrum exhibited a broad absorption at 3332 cm^−1^ (−OH) and strong absorptions at 1651 and 1574 cm^−1^, consistent with the presence of an unsaturated carbonyl group. Similarly, the typical tetrasubstituted *γ*-pyrone moiety in **5** was also identified by the characteristic NMR data of a ketone carbonyl (*δ*_C_ 182.3, qC), two tetrasubstituted double bonds (*δ*_C_ 99.4, qC, *δ*_C_ 162.0, qC; *δ*_C_ 121.3, qC, 160.0, qC), two methyls (*δ*_C_ 7.1, CH_3_-16; *δ*_C_ 12.6, CH_3_-17), and one methoxyl (*δ*_C_ 55.7, OMe), which was also supported by its UV spectrum (MeOH, *λ*_max_ = 257 nm, log*ε* = 2.8). The ^1^H NMR data (Table 1) displayed two vinyl methyls at *δ*_H_ 1.82 (3H, s, H_3_-19) and *δ*_H_ 1.73 (3H, s, H_3_-20) and two olefinic protons appearing at *δ*_H_ 5.47 (1H, s, H-7) and 5.73 (1H, s, H-9), which were attributed to two trisubstituted double bonds. In addition, proton signals were also observed for one terminal double bond at *δ*_H_ 5.20 (1H, s, H-21) and 5.17 (1H, s, H-21), one oxymethine at *d*_H_ 3.58 (1H, br s, H-13), and one triplet methyl at *δ*_H_ 0.88 (3H, t, *J* = 7.3 Hz, H_3_-15) in the ^1^H NMR spectrum. Further analysis of the ^13^C NMR data (Table 2) and HSQC experiment of **5**, disclosed 22 signals for seven methyls, one sp^3^ methylene, two sp^3^ methines, one sp^3^ quaternary carbon, one sp^2^ methylene, two sp^2^ methines, and eight sp^2^ quaternary carbons. The NMR signal of an oxygenated carbon was observed at *δ*_C_ 88.1, implying that the remaining two oxygen atoms were involved in a hydroperoxyl group. Finally, the ^1^H−^1^H COSY cross peaks readily assigned the only one spin system from H-13 to H_3_-15, along with the significant HMBC correlations from the protons of seven methyls to the carbon signals, via two and three bonds, elucidated the planar structure of **5** as depicted in Fig. 2.

**Fig. 4:**
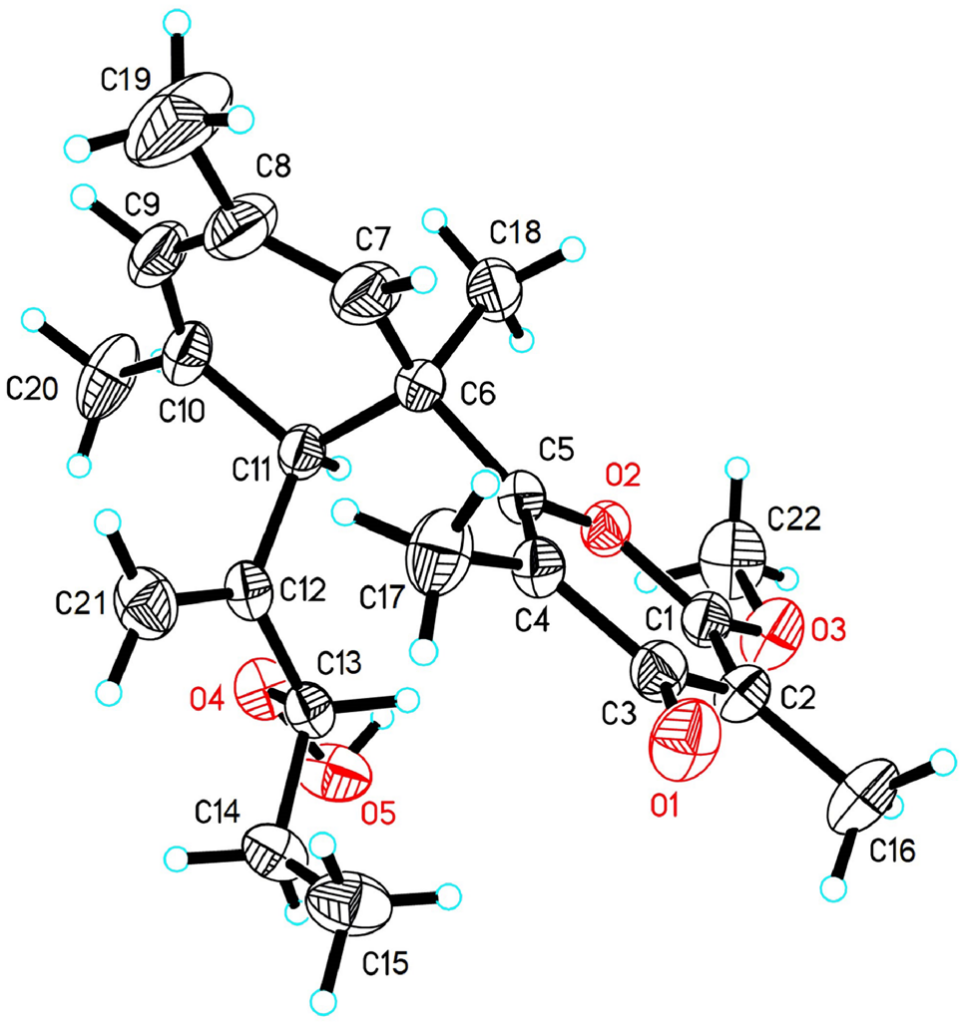
The ORTEP drawing of tridachiapyrone J (**4**)

The detailed comparison of the NMR data of **5** with those of the co-occurring known compound, tridachiapyrone J (**4**), revealed that the two compounds are stereoisomers with different configurations at C-13. Notably, tridachiapyrone J (**4**) together with the hydroxyl analogues tridachiapyrone G (**6**) and H (**7**) were first isolated by Schmitz’s group with the stereochemistry unassigned (Fu et al. 2000). The absolute configuration of **4** was unambiguously determined to be 6*R*, 11*R*, 13*R* (as shown in Fig. 1) by X-ray diffraction analysis using Cu K*α* radiation (*λ* = 1.54178 Å) (Fig. 4, CCDC deposition number 1953946) with the absolute structure parameter as − 0.07(6) in this study. Therefore, the absolute configuration of **5** was indirectly determined to be 6*R*, 11*R*, 13*S*. The chemical conversion from **4** to **6** with the triphenylphosphine (PPh_3_) as reductant was accomplished (Fig. 3B), which assigned the stereochemistry of **6** and **7**. Moreover, a simple protonic acid (TFA) catalyzed reaction of **6** was further completed, giving a high yield epoxidation product **6a**.

Ocellatuspyrone D (**9**) was isolated as an optically active colorless oil ([*α*] + 89.9 (*c* 0.24 CHCl_3_)), possessing the same molecular formula of C_22_H_30_O_5_ as **5** by the HR-ESIMS ion peak at *m/z* 375.2175 [M + H]^+^ (calcd. for C_22_H_31_O_5_, 375.2166). The IR and UV spectra of **9** closely resembled those of **5**, suggesting similar functionalities in the molecule. Analysis of the ^1^H and ^13^C NMR data (Tables 1 and 2) of **9** were also similar to those of **5**, except for the migration of the Δ^12(21)^ double bond in **5** to Δ^12(13)^ [*δ*_H_ 5.06 (1H, d, *J* = 8.2 Hz, H-13); *δ*_C_ 138.1 (qC, C-12) and 129.6 (CH, C-13)] and the hydroperoxyl group at C-13 in **5** transferred to C-14 [*δ*_H_ 4.57 (1H, dq, *J* = 8.2, 6.4 Hz, H-14); *δ*_C_ 77.5 (CH, C-14)]. These observations were supported by the ^1^H−^1^H COSY cross peak from H-13 to H-14 and the HMBC correlations (Fig. 2) from olefinic proton H-13 resonating at *δ*_H_ 5.06 to C-11 (*δ*_C_ 59.3) and C-15 (*δ*_C_ 18.4). The geometry of the double bond at Δ^12(13)^ was assigned to be *E* by the NOESY correlation (Fig. 2) of H_3_-21 (*δ*_H_ 1.45) with H-14 (*δ*_H_ 4.57). The relative stereochemistry of C-6 and C-11 was assigned to be 6R*, 11R* from a NOESY correlation observed between H-11 (*δ*_H_ 2.78) and H_3_-18 (*δ*_H_ 1.48).

Ocellatuspyrone E (**10**) was also isolated as an optically active colorless oil ([*α*] + 96.7 (*c* 0.17 CHCl_3_)), with the same molecular formula of C_22_H_30_O_5_ as **9** by the HR-ESIMS ion peak at *m/z* 375.2166 [M + H]^+^ (calcd. for C_22_H_31_O_5_, 375.2166). Compound **10** had the same IR and UV absorptions with those for **9**. Comparison of ^1^H and ^13^C NMR data of **10** and **9** demonstrated that almost all of these data were virtually identical except the chemical shift of H_3_-15 (*δ*_H_ 0.89, d, *J* = 6.3 Hz) in **10** instead of the H_3_-15 (*δ*_H_ 1.09, d, *J* = 6.4 Hz) in **9**, suggesting that they are 14-epimers. The geometry of the double bond at Δ^12(13)^ was also assigned to be *E* by the NOESY correlation of H_3_-21 (*δ*_H_ 1.46) with H-14 (*δ*_H_ 4.57), and the relative configuration of C-6 and C-11 was determined to be the same 6R*, 11R* as those of **9** on the basis of the NOESY experiment (Fig. 2). Furthermore, the relative configurations at C-14 in **9** and **10** were deduced to be 14R* and 14S*, respectively, by the analysis of the vicinal proton-proton coupling constants (Fig. 3C) based on the well-known Karplus-equation (Haasnoot et al. 1980).

**Fig. 5:**
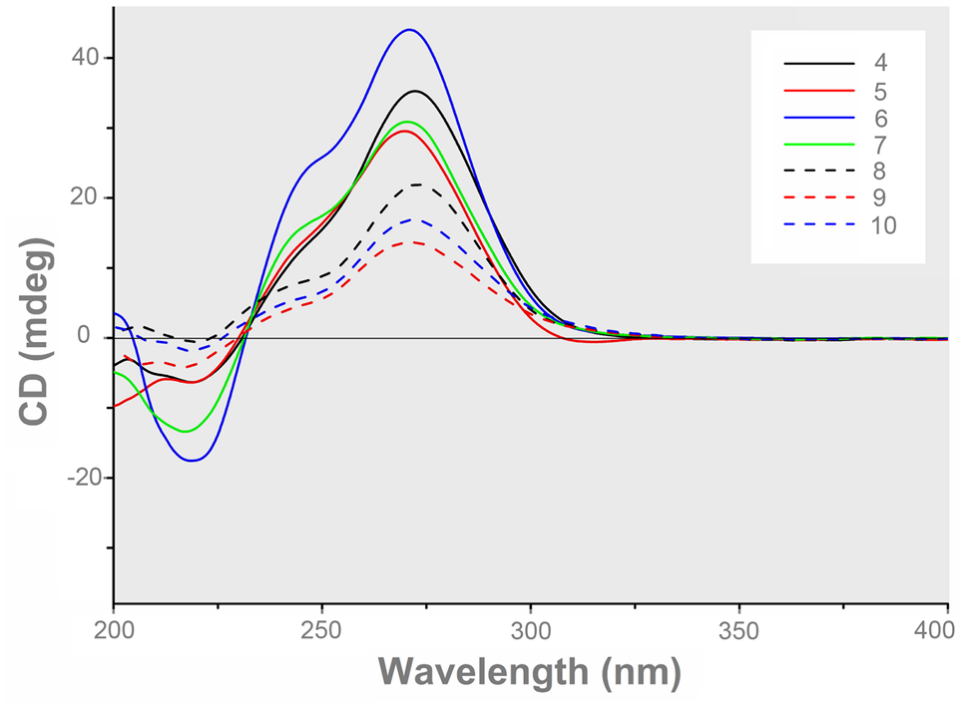
The experimental ECD spectra of compounds **4**−**10**

The absolute configurations of all the stereogenic centers in **9** and **10** were defined by ECD experiments. The ECD spectrum of **9** showed a positive Cotton effect at 272 nm (Δ*ε* =  + 13.6) and a negative Cotton effect at 217 nm (Δ*ε* =  − 4.2), while the ECD spectrum of **10** showed a positive Cotton effect at 272 nm (Δ*ε* =  + 16.9) and a negative Cotton effect at 218 nm (Δ*ε* =  − 1.8), consistent with those of **4**−**8** (Fig. 5), indicative of (6*R*, 11*R*, 14*R*)- and (6*R*, 11*R*, 14*S*)-configurations, respectively. Accordingly, the structures of **9** and **10** were characterized as shown in Fig. 1.

Ocellatuspyrone F (**11**) was isolated as a colorless oil. The HR-ESIMS of **11** showed a fragment ion peak at *m/z* 375.2177 [M + H]^+^ (calcd. for C_22_H_31_O_5_, 375.2166), suggesting a molecular formula of C_22_H_30_O_5_ with eight degrees of unsaturation. Similarly, the ^1^H and ^13^C NMR data (Tables 1 and 2) of **11** showed typical signals for a *α*-methoxy-*β*-methyl-*γ*-pyrone polypropionate nucleus with eight methyls, one sp^3^ methylene, one sp^3^ methine, one sp^3^ quaternary carbon, two sp^2^ methines, and nine sp^2^ quaternary carbons, which was also supported by a UV absorption at 252 nm (log*ε* = 2.9) and IR band at 1652 and 1580 cm^−1^. The remaining two oxygen atoms were assigned to be a hydroperoxyl group, which was confirmed by the presence of a proton signal at *δ*_H_ 4.47 (1H, s, H-9) and an oxygenated methine carbon at *δ*_C_ 84.0 (CH, C-9), signaling that **11** was also the isomer of **9**. In fact, the NMR data of **11** were nearly identical to those of **9**, with the exception of the hydroperoxyl group substituted at C-9 in **11** instead of the C-14 in **9** and the migration of Δ^9(10)^ double bond in **9** to Δ^10(11)^ [*δ*_C_ 125.3 (qC, C-10), 143.3 (qC, C-11)] in **11**. This replacement led the ^13^C resonance of C-9, C-21 to be shifted upfield and C-11 downfield, respectively. The position of the hydroperoxyl group at C-9 was further secured by the HMBC correlations (Fig. 2) from both H_3_-19 (*δ*_H_ 1.90) and H_3_-20 (*δ*_H_ 1.79) to C-9. The geometry of the Δ^12(13)^ double bond in **11** was determined to be *E* by the NOESY cross peaks of H_3_-21 (*δ*_H_ 1.57) and H_2_-14 (*δ*_H_ 1.96). The determination of relative configurations of C-6 and C-9 of **11** were further determined by QM-NMR calculations following the DP4 + protocol. Two possible candidate isomers of **11** were built (**11a** and **11b**, Supplementary Fig. S15b). Next, the conformational searches followed by DFT optimization and calculation of NMR parameters at PCM/mPW1PW91/6-31G(d) level of theory were undertaken. As a result, the candidate structure with the relative configuration 6R*, 9S* gave a best match between experimental and calculated data with over 99% probability (Supplementary Fig. S15d).

Ocellatuspyrone G (**12**) was isolated as a colorless oil and exhibited the molecular formula C_22_H_30_O_3_ as determined by its HR-ESIMS ion peak at *m/z* 343.2274 [M + H]^+^ (calcd. for C_22_H_31_O_3_, 343.2268), which was 32 mass units less than that of **11**, appropriate for eight degrees of unsaturation. The ^1^H and ^13^C NMR data (Tables 1 and 2) of **12** closely resembled those of **11**, except for the migration of the Δ^7(8)^ double bond in **11** to the Δ^8(9)^ double bond [*δ*_H_ 5.59 (1H, s, H-9); *δ*_C_ 132.0 (qC, C-8) and 123.6 (CH, C-9)] in **12**, led to the appearance of a sp^2^ methylene [*δ*_H_ 2.84 (1H, d, *J* = 17.9 Hz, H-7a), 1.99 (1H, d, *J* = 17.9 Hz, H-7b); *δ*_C_ 43.2 (CH_2_, C-7)] and the absence of the hydroperoxyl group at C-9. These observations were supported by the missing molecular mass and the HMBC correlations (Fig. 2) from the H_3_-19 (*δ*_H_ 1.77) to C-7, C-8, C-9 and from H_3_-20 (*δ*_H_ 1.72) to C-9, C-10 (*δ*_C_ 126.0, qC), C-11 (*δ*_C_ 136.3, qC). Therefore, the planar structure of **12** was elucidated as depicted. Analogously, the *E* geometry of the Δ^12(13)^ double bond in **12** was also determined by the NOESY cross peaks of H_3_-21 (*δ*_H_ 1.51) and H_2_-14 (*δ*_H_ 1.93).

**Fig. 6:**
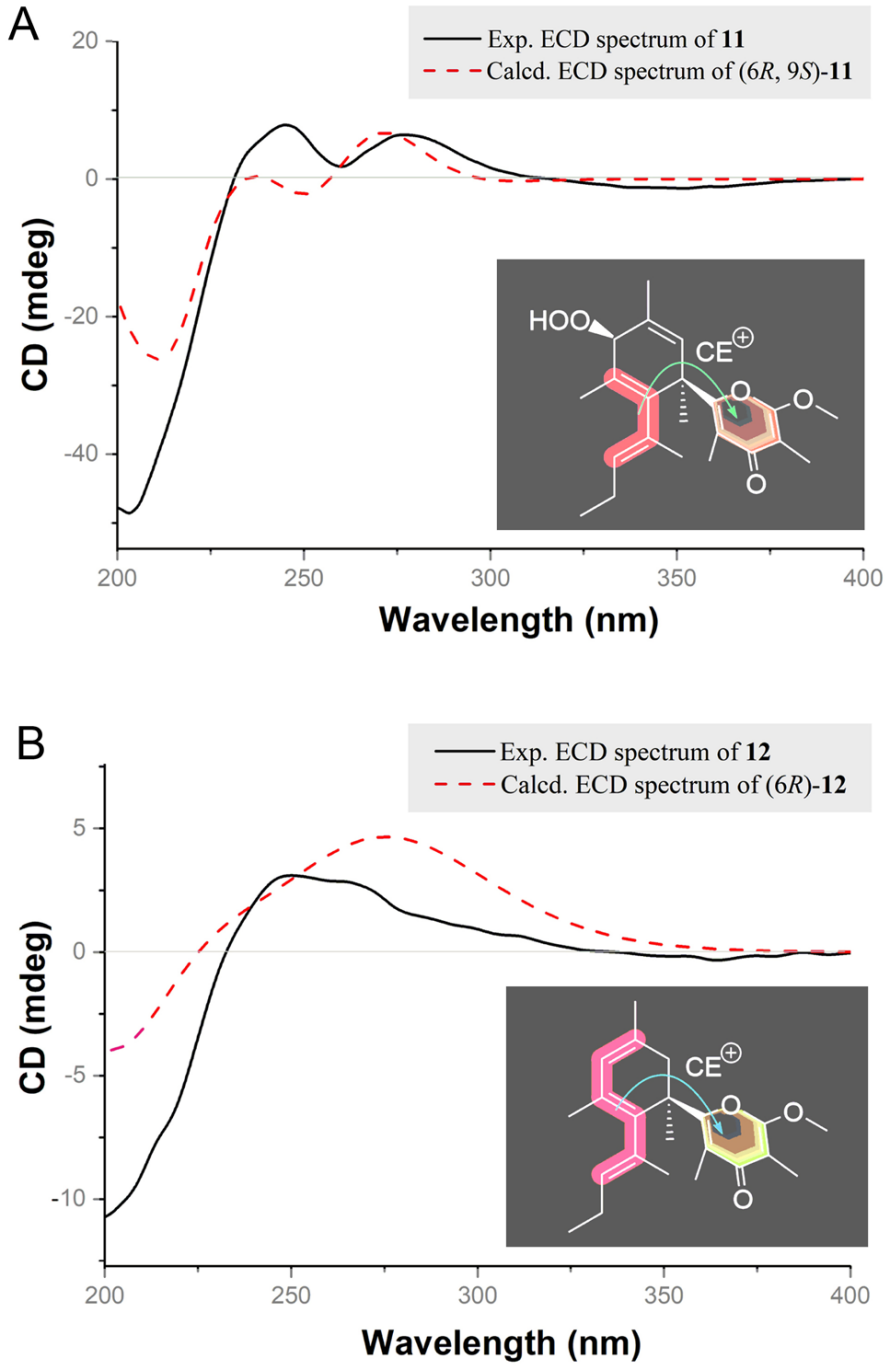
The electric transition dipole of the main chromophores for compounds **11** and **12**, and the assignment of the absolute configurations of **11** and **12** by comparing TDDFT-ECD calculated and the experimental spectra. *CE* cotton effect

To determine the absolute configurations of **11** and **12**, the CD exciton chirality method (Zhang et al. 2013) was applied. As shown in Fig. 6, the ECD spectra of **11** and **12** showed similar positive chirality owing to the exciton coupling between the chromophores *γ*-pyrone and conjugated double bonds, suggested the C-6 connected *γ*-pyrone as the main chromophore to impact the ECD spectrum of such compounds. Thus, the transition dipole moments of the two chromophores oriented in a clockwise manner determined the 6*R* absolute configurations of **11** and **12**. Moreover, from a biogenetic point of view, the absolute configurations at C-6 of **11** and **12** were also suggested to be the *R*-configuration as in the co-occurring compounds **4**−**10**. Meanwhile, for further confirmation of the absolute configurations of compounds **11** and **12**, the time-dependent density functional theory electronic circular dichroism (TDDFT-ECD) calculations (Ye et al. 2017) were performed. Finally, the calculated ECD spectra of (6*R*, 9*S*)-**11** and (6*R*)-**12** appeared to be similar to positive Cotton effect curves and highly matched to those experimental ones (Fig. 6). Therefore, the absolute configurations of ocellatuspyrone F (**11**) and ocellatuspyrone G (**12**) were assigned to be (6*R*, 9*S*) and (6*R*), respectively.

In in vitro bioassays, the isolates were tested for antibacterial activities against *Staphylococcus aureus*, *Streptococcus parauberis, Lactococcus garviea, Aeromon*as *salmonicida*, *Pseudomonas aeruginosa*, and *Photobacterium halotolerans,* and only three compounds (**7**, **8**, and **11**) showed weak antibacterial activities against *Streptococcus parauberis* with the MIC value of 35.8, 34.2, and 37.4 μg/mL, respectively. Comparing the active compounds and the inactive compounds **1**−**3**, we found that, the bicyclo[3.1.0]hexene fragment was not essential for the activity. Moreover, a comparison of active **11** and the inactive **12** indicated that the introduction of a hydroxyl group at C-9 is beneficial for the antibacterial activity. In addition, the anti-inflammatory effect in lipopolysaccharide (LPS) induced RAW264.7 cell inflammation, the neuroprotective effect on hydrogen peroxide (H_2_O_2_) induced SH-SY5Y cell damage, the PTP1B inhibitory activity, and the antiviral activity against 2019-nCoV of these metabolites were also screened, and all these compounds were inactive at the 20 μmol/L level (see the details in Supplementary Tables S2−S6).

## Discussion

**Fig. 7:**
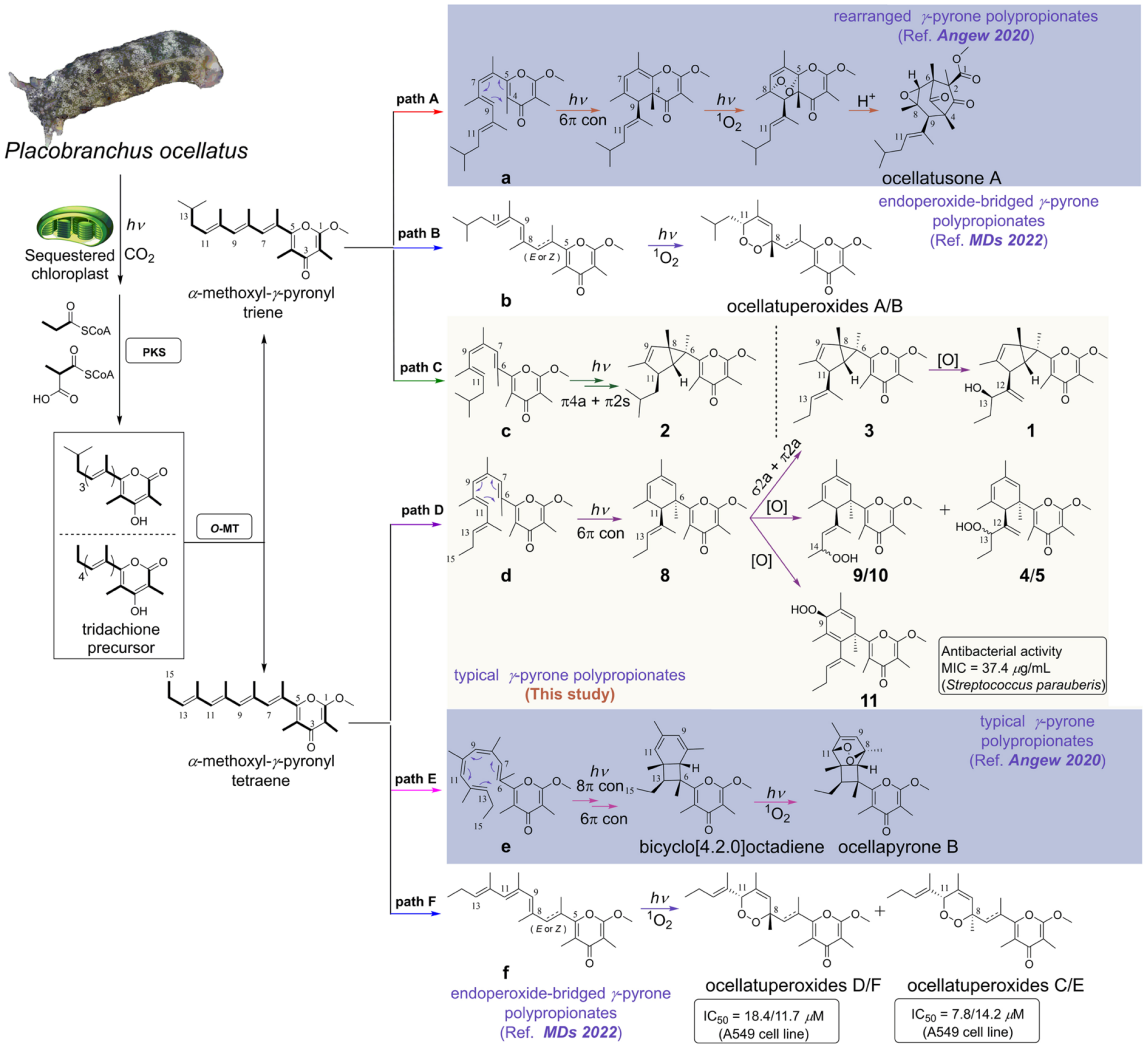
Proposed photobiosynthetic pathway of *P. ocellatus*-derived *γ*-pyrone polypropionates

Compounds **1**−**12** belong to three biogenetically related types of *γ*-pyrone polypropionates. As summarized in Fig. 7, the characterized polypropionates from *P. ocellatus* were categorized into three groups, i.e., rearranged, endoperoxide-bridged, and classical *γ*-pyrone polypropionates. The biosynthetic process of all these isolated pyrone polypropionates could be classified into six routes (**path A**−**F**) using *α*-methoxyl-*γ*-pyronyl triene and *α*-methoxyl-*γ*-pyronyl tetraene as the precursors. Photobiosynthetic pathways of rearranged (Fig. 7**path A**) bicyclo[4.2.0]octadiene containing (Fig. 7**path E**) and endoperoxide-bridged (Fig. 7**path B** and **path F**) *γ*-pyrones have been proposed in our previous work (Li et al. 2022; Wu et al. 2020).

In this study, starting from the *α*-methoxyl-*γ*-pyronyl triene precursor, compound **2** could be directly formed via a π4a + π2a electrocyclization (Fig. 7**path C**). As for **path D**, using *α*-methoxyl-*γ*-pyronyl tetraene as the precursor, the common branched compound **8** is generated through a photochemical 6π conrotatory electrocyclization on intermediate **d**. Subsequent σ2a + π2a electrocyclization leads to the formation of **3**, which might be further oxidized to give **1**. Peroxidation occurred on different carbon positions of **8,** offering a series of downstream metabolites **4**, **5**, and **9**−**11**.

Chemically, we performed a photochemical reaction on **6** and **8**, respectively, with the treatment of methylene blue and rose Bengal in CH_2_Cl_2_ followed with exposure to sunlight for serval hours. As a result, neither compound **1** nor **3** was detected accordingly as previously reported (Supplementary Figs. S17a and S17b) (Zuidema et al. 2005). Therefore, it is speculated that the selection of appropriate photosensitizer and the light radiation energy play the important role in this reaction. We further treated compound **8** with TFA and/or silica gel in CH_2_Cl_2_ at room temperature overnight and a new constituent was not formed (Supplementary Fig. S17c). Thus, we can conclude that bicyclo[3.1.0]hexene containing pyrones are not artifacts from the purification process.

## Conclusions

In summary, further chemical investigation of the South China Sea sacoglossan *P. ocellatus* has resulted in the isolation and characterization of seven new *γ*-pyrone polypropionates, namely ( ±)-ocellatuspyrone A (**1**) and ( ±)-ocellatuspyrone B (**2**) and ocellatuspyrones C−G (**5**, **9**−**12**), along with five known compounds (**3**, **4**, **6**−**8**). Our continuous chemical study on this animal has not only enriched the chemical diversity of *γ*-pyrone polypropionates but also expanded the total number of compounds within the polypropionate family. In particular, compounds ( ±)-**1** and ( ±)-**2**, two pairs of enantiomers, represent new examples of *γ*-pyrone polypropionate featuring an unprecedented bicyclo[3.1.0]hexene scaffold. Moreover, the structures of stereochemistry undefined known compounds **4**, **6** and **7** were determined for the first time in the present work. It is interesting to note that ( ±)-photodeoxytridachione (**3**) was first reported to be the photochemical rearranged products of 9,10-deoxytridachione (**8**) upon exposure to sunlight, and the ^14^C-labeling experiments indicated that this photoisomerization conversion was a non-enzymatic light-catalyzed reaction occurring in vivo (Ireland and Scheuer 1979). The impressive structures with their photochemical properties attracted much attention from natural products chemists and synthetic chemists. Several types of mechanistic and synthetic evidence were also reported previously to support this biosynthesis isomerization process (Bruckner et al. 2003; Eade et al. 2008; Jeffery and Perkins 2004; Zuidema et al. 2005). Similarly, molecules ( ±)-**1** were proposed to be the rearranged products of tridachiapyrone G (**6**) by the same photochemical conversion mechanism. In bioassays, compounds **7**, **8**, and **11** showed weak antibacterial activities against *S. parauberis* with MIC values of 35.8, 34.2, and 37.4 μg/mL, respectively. These active *γ*-pyrones provide promising starting points for further functional optimization. In addition, further studies could be conducted to complete the characterization of the proposed biosynthetic pathway. At the same time, because of the unique ecological and living environment of marine mollusks, many silent genes may need to be activated by external stimuli, leading to promising future prospects of even greater chemical diversity and biological activity.

## Materials and methods

### General experimental procedures

Melting points were measured on an X-4 digital micro-melting point apparatus. IR spectrum was recorded on a Nicolet 6700 spectrometer (Thermo Scientific, Waltham, MA, USA), peaks are reported in cm^–1^. UV and ECD spectra were recorded on a Jasco J-815 spectropolarimeter (JASCO, Japan) at ambient temperature using chromatographic grade CH_3_OH and CH_3_CN as solvents. Optical rotations were measured on a PerkinElmer 241MC polarimeter (PerkinElmer, Fremont, CA, USA). ^1^H and ^13^C NMR spectra were acquired on a Bruker DRX 400, 500 and Avance 600 MHz NMR spectrometers (Bruker Biospin AG, Fallanden, Germany). Chemical shifts are reported in parts per million (*δ*) in CDCl_3_ with the residual CHCl_3_ (*δ*_H_ 7.26 × 10^–6^) as the internal standard for ^1^H NMR spectrometry and CDCl_3_ (*δ*_C_ 77.16 × 10^–6^) for ^13^C NMR spectrometry. The HR-ESIMS spectra were recorded on an Agilent G6250 Q-TOF (Agilent, Santa Clara, CA, USA). Semi-preparative HPLC was performed on an Agilent 1260 series liquid chromatography system equipped with a DAD G1315D detector at 210 and 254 nm (Agilent, Santa Clara, CA, USA), and an Agilent semi-preparative XDB-C18 column (5 μm, 250 mm × 9.4 mm) was employed for the purification. Commercial silica gel (200˗300 mesh and 300˗400 mesh; Qingdao Haiyang Chemical Co., Ltd., Qingdao, China) was used for column chromatography, and precoated silica gel GF254 plates (Sinopharm Chemical Reagent Co., Shanghai, China) were used for analytical TLC. Spots were detected on TLC under UV light or by heating after spraying with anisaldehyde H_2_SO_4_ reagent. Sephadex LH-20 (Pharmacia, USA) was also used for column chromatography. All solvents used for column chromatography and HPLC were of analytical grade (Shanghai Chemical Reagents Co., Ltd., Shanghai, China) and chromatographic grade (Dikma Technologies Inc., CA, USA), respectively.

### Biological material

The mollusk *P. ocellatus* (500 specimens) was collected off the shallow water area, Ximao Island, Hainan Province, China, in 2017. The biological samples and high-definition pictures of the mollusk were sent to marine biologist Dr. Christiane Waldrich and identified as *P. ocellatus*. The voucher specimen (No. 17XD-12) is available for inspection at the Shanghai Institute of Materia Medica, CAS.

### Extraction and isolation

The frozen animals (55.0 g, dry weight) were directly extracted with MeOH–CH_2_Cl_2_ (1:1) in sonicate at room temperature (6 × 500 mL). The organic extract was evaporated to give a brown residue, and the residue was then partitioned between H_2_O and Et_2_O. The upper layer was concentrated under reduced pressure to give a brown crude extract 1.5 g. The resulting residue was separated into seven fractions (A–G) by gradient Silica-gel column chromatography (CC) (100–200 mesh) eluting with petroleum ether (PE, 60–90 °C)–Et_2_O (100:0 to 0:100). Purification of fraction C (483 mg) by gradient Silica-gel CC (200–300 mesh) eluting with PE–Et_2_O (90:10 to 25:75) afforded 5 subfractions (C1–C5). Subfraction C3 (243 mg) was initially fractioned by gradient Silica-gel CC (300–400 mesh) eluting with PE–Et_2_O (75:25 to 50:50) to afford colorless oil compound **8** (85.8 mg) and subfraction C3B (23 mg). C3B was then purified by semi-preparative RP-HPLC (CH_3_OH–H_2_O, 90:10, 3.0 mL/min) to afford colorless oil compounds **12** (6.7 mg, *t*_R_ = 10.3 min), **2** (1.5 mg, *t*_R_ = 11.6 min) and **3** (1.0 mg, *t*_R_ = 14.2 min). The subfraction C4 (16 mg) was further purified by semi-preparative RP-HPLC (CH_3_CN–H_2_O, 70:30, 3.0 mL/min), yielding colorless oil compounds **9** (2.4 mg, *t*_R_ = 12.0 min) and **10** (1.7 mg, *t*_R_ = 16.3 min). The subfraction C5 (92 mg) was purified by Silica-gel CC again to give 2 subfractions (C5A and C5B). C5A (7 mg) was then purified by semi-preparative RP-HPLC (CH_3_CN–H_2_O, 60:40, 3.0 mL/min) to afford colorless oil compounds **11** (0.9 mg, *t*_R_ = 10.5 min), and C5B (42 mg) was further purified by semi-preparative RP-HPLC (CH_3_CN–H_2_O, 60:40, 3.0 mL/min) to afford colorless crystal compound **4** (7.6 mg, *t*_R_ = 13.4 min) and colorless oil compound **5** (1.0 mg, *t*_R_ = 16.6 min). The fraction F (54 mg) was initially fractioned by Sephadex LH-20 CC eluting with PE–CH_2_Cl_2_–CH_3_OH (2:1:1) to give 4 subfractions (F1–F4). Finally, the colorless oil compounds **1** (3.0 mg, *t*_R_ = 7.6 min), **6** (4.2 mg, *t*_R_ = 8.1 min) and **7** (1.7 mg, *t*_R_ = 9.9 min) were yielded from the subfraction F4 (20 mg) by semi-preparative RP-HPLC (CH_3_CN–H_2_O, 70:30, 3.0 mL/min).

Due to the racemic nature of compound **1**, further chiral HPLC separation was successfully applied to get the optically pure compounds. Compounds ( ±)-**1** were isolated by CHIPALPAK IB N-3 column to afford (−)-**1** {1.2 mg, *t*_R_ = 12.1 min, [*α*] − 14.0 (*c* 0.05 CHCl_3_)} and ( +)-**1** {1.4 mg, *t*_R_ = 12.7 min, [*α*] + 30.8 (*c* 0.04 CHCl_3_)}, eluted with CH_3_CN–H_2_O (85:15) as the mobile phase, flow rate 1.0 mL/min. UV detector was set to 254 nm.

( ±)-Ocellatuspyrone A (**1**): Colorless oil; [*α*] + 3.5 (*c* 0.4 CHCl_3_); UV (MeOH): *λ*_max_ (log*ε*) 257 (2.8) nm; IR (KBr): *ν* = 3395, 2959, 2925, 2854, 1658, 1584, 1464, 1416, 1378, 1332, 1164, 1045, 984 cm^−1^; ^1^H and ^13^C NMR data see Tables 1 and 2; HR-ESIMS *m/z* 359.2210 [M + H]^+^ (calcd. for C_22_H_31_O_4_, 359.2217).

( ±)-Ocellatuspyrone B (**2**): Colorless oil; [*α*] − 0.8 (*c* 0.15 CHCl_3_); UV (MeOH): *λ*_max_ (log*ε*) 257 (3.0) nm; IR (KBr): *ν* = 2955, 2926, 2869, 1662, 1602, 1463, 1408, 1376, 1331, 1253, 1168, 1043, 984 cm^−1^; ^1^H and ^13^C NMR data see Tables 1 and 2; HR-ESIMS *m/z* 331.2272 [M + H]^+^ (calcd. for C_21_H_31_O_3_, 331.2268).

Ocellatuspyrone C (**5**): Colorless oil; [*α*] + 83.3 (*c* 0.02 CHCl_3_); UV (MeOH): *λ*_max_ (log*ε*) 257 (2.8) nm; IR (KBr): *ν* = 3332, 2963, 2932, 2877, 1731, 1651, 1574, 1467, 1378, 1322, 1259, 1167, 736 cm^−1^; ^1^H and ^13^C NMR data see Tables 1 and 2; HR-ESIMS *m/z* 375.2169 [M + H]^+^ (calcd. for C_22_H_31_O_5_, 375.2166).

Ocellatuspyrone D (**9**): Colorless oil; [*α*] + 89.9 (*c* 0.24 CHCl_3_); UV (MeOH): *λ*_max_ (log*ε*) 253 (2.8) nm; IR (KBr): *ν* = 3375, 2965, 2926, 2869, 1714, 1655, 1586, 1464, 1376, 1319, 1255, 1167, 1026 cm^−1^; ^1^H and ^13^C NMR data see Tables 1 and 2; HR-ESIMS *m/z* 375.2175 [M + H]^+^ (calcd. for C_22_H_31_O_5_, 375.2166).

Ocellatuspyrone E (**10**): Colorless oil; [*α*] + 96.7 (*c* 0.17 CHCl_3_); UV (MeOH): *λ*_max_ (log*ε*) 254 (2.9) nm: IR (KBr) *ν* = 3388, 2961, 2927, 2869, 1714, 1652, 1574, 1466, 1378, 1322, 1257, 1167, 1042 cm^−1^; ^1^H and ^13^C NMR data see Tables 1 and 2; HR-ESIMS *m/z* 375.2166 [M + H]^+^ (calcd. for C_22_H_31_O_5_, 375.2166).

Ocellatuspyrone F (**11**): Colorless oil; [*α*] − 41.7 (*c* 0.02 CHCl_3_); UV (MeOH): *λ*_max_ (log*ε*) 252 (2.9) nm; IR (KBr): *ν* = 3375, 2963, 2929, 2875, 1652, 1580, 1465, 1412, 1377, 1319, 1257, 1167, 984 cm^−1^; ^1^H and ^13^C NMR data see Tables 1 and 2; HR-ESIMS *m/z* 375.2177 [M + H]^+^ (calcd. for C_22_H_31_O_5_, 375.2166).

Ocellatuspyrone G (**12**): Colorless oil; [*α*] + 1.0 (*c* 0.7 CHCl_3_); UV (MeOH): *λ*_max_ (log*ε*) 254 (2.5) nm; IR (KBr): *ν* = 3306, 2960, 2930, 2873, 1651, 1574, 1464, 1415, 1378, 1322, 1255, 1171, 736 cm^−1^; ^1^H and ^13^C NMR data see Tables 1 and 2; HR-ESIMS *m/z* 343.2274 [M + H]^+^ (calcd. for C_22_H_31_O_3_, 343.2268).

### Preparation of the (*S*)- and (*R*)-MTPA ester derivatives of compounds ( ±)-1

Compound ( +)-**1** (1.4 mg) was dissolved in 1.0 mL of pyridine-*d*_5_, and the solution was divided into two equal parts and transferred into two NMR tubes. To initiate the reaction, 15 μL of (*R*)- and (*S*)-MTPA-Cl were added to the two NMR tubes, respectively. The reactions were found to be completed in 30 min by the monitoring of ^1^H NMR, and the reaction residues were purified by RP-HPLC, yielding the mono (*S*)- and (*R*)-MTPA ester derivatives of ( +)-**1**, respectively.

Analogously, 1.2 mg of compound (−)-**1** was reacted in two NMR tubes with 15 μL of (*R*)- and (*S*)-MTPA-Cl for 30 min, to afford the mono (*S*)- and (*R*)-MTPA ester derivatives of (−)-**1**, respectively.

(*S*)-MTPA-( +)-**1**: Colorless oil (0.5 mg); ^1^H NMR (400 MHz, CDCl_3_): *δ*_H_ 7.519 (2H, m, Ar-H), 7.397 (3H, m, Ar-H), 5.434 (1H, s, H-9), 5.264 (1H, t, *J* = 6.0 Hz, H-13), 5.016 (1H, s, H-21a), 4.877 (1H, s, H-21b), 4.009 (3H, s, 1-OMe), 3.561 (3H, s, MTPA-OMe), 2.692 (1H, s, H-11), 2.015 (3H, s, H_3_-17), 1.880 (3H, s, H_3_-16), 1.830 (2H, m, H_2_-14), 1.525 (3H, s, H_3_-20), 1.182 (3H, s, H_3_-19), 1.131 (3H, s, H_3_-18), 0.982 (3H, t, *J* = 7.4 Hz, H_3_-15) × 10^–6^.

(*R*)-MTPA-( +)-**1**: Colorless oil (0.8 mg); ^1^H NMR (400 MHz, CDCl_3_): *δ*_H_ 7.520 (2H, m, Ar-H), 7.401 (3H, m, Ar-H), 5.454 (1H, s, H-9), 5.332 (1H, t, *J* = 6.0 Hz, H-13), 5.214 (1H, s, H-21a), 4.977 (1H, s, H-21b), 3.973 (3H, s, 1-OMe), 3.530 (3H, s, MTPA-OMe), 2.740 (1H, s, H-11), 1.998 (3H, s, H_3_-17), 1.864 (3H, s, H_3_-16), 1.789 (2H, m, H_2_-14), 1.567 (3H, s, H_3_-20), 1.183 (3H, s, H_3_-19), 1.131 (3H, s, H_3_-18), 0.887 (3H, t, *J* = 7.4 Hz, H_3_-15) × 10^–6^.

(*S*)-MTPA-(−)-**1**: Colorless oil (0.7 mg); ^1^H NMR (400 MHz, CDCl_3_): *δ*_H_ 7.519 (2H, m, Ar-H), 7.400 (3H, m, Ar-H), 5.448 (1H, s, H-9), 5.339 (1H, t, *J* = 6.0 Hz, H-13), 5.214 (1H, s, H-21a), 4.978 (1H, s, H-21b), 3.957 (3H, s, 1-OMe), 3.530 (3H, s, MTPA-OMe), 2.742 (1H, s, H-11), 1.980 (3H, s, H_3_-17), 1.841 (3H, s, H_3_-16), 1.788 (2H, m, H_2_-14), 1.183 (3H, s, H_3_-19), 1.125 (3H, s, H_3_-18), 0.886 (3H, t, *J* = 7.4 Hz, H_3_-15) × 10^–6^.

(*R*)-MTPA-(−)-**1**: Colorless oil (0.4 mg); ^1^H NMR (400 MHz, CDCl_3_): *δ*_H_ 7.519 (2H, m, Ar-H), 7.396 (3H, m, Ar-H), 5.429 (1H, s, H-9), 5.282 (1H, t, *J* = 6.0 Hz, H-13), 5.026 (1H, s, H-21a), 4.883 (1H, s, H-21b), 3.979 (3H, s, 1-OMe), 3.562 (3H, s, MTPA-OMe), 2.697 (1H, s, H-11), 1.987 (3H, s, H_3_-17), 1.844 (3H, s, H_3_-16), 1.817 (2H, m, H_2_-14), 1.517 (3H, s, H_3_-20), 1.184 (3H, s, H_3_-19), 1.123 (3H, s, H_3_-18), 0.978 (3H, t, *J* = 7.4 Hz, H_3_-15) × 10^–6^.

### Chemical conversion of compounds 4 and 6

A total of 5.0 mg of compound **4** was dissolved in 5.0 mL of CH_2_Cl_2_ and added to excess PPh_3_ (10.0 mg) and stirred for 2 h at room temperature. The residue obtained after the reaction was purified by Silica-gel CC eluting with PE–Et_2_O (50:50) to afford compound **6** (3.6 mg, 72% yield). The NMR data of the obtained compound are consistent with that of isolated natural compound **6** (Supplementary Fig. S8).

A total of 5.5 mg of compound **6** was dissolved in 5.0 mL of CH_2_Cl_2_, and stirred overnight at room temperature with trifluoracetic acid (TFA, 100 μL) as a catalyst. The compound **6a** (5.5 mg, 99% yield) obtained after reaction was identified by 1D and 2D NMR and HR-ESIMS data (Supplementary Figs. S9a–S9g). ^1^H NMR data of **6a** (600 MHz, CDCl_3_): *δ*_H_ 5.39 (1H, s, H-9), 4.79 (1H, s, H-21a), 4.38 (1H, d, *J* = 6.6 Hz, H-13), 4.25 (1H, s, H-21b), 4.06 (3H, s, OMe), 3.30 (1H, s, H-11), 2.71 (1H, d, *J* = 16.3 Hz, H-7a), 2.23 (3H, s, H_3_-17), 2.12 (1H, d, *J* = 16.3 Hz, H-7b), 1.94 (3H, s, H_3_-16), 1.70 (3H, s, H_3_-19), 1.54 (3H, s, H_3_-20), 1.49 (3H, s, H_3_-18), 1.43 (2H, m, H_2_-14), 0.93 (3H, t, *J* = 7.4 Hz, H_3_-15) × 10^–6^; ^13^C NMR data of **6a** (150 MHz, CDCl_3_): *δ*_C_ 182.4 (qC, C-3), 164.1 (qC, C-5), 163.6 (qC, C-1), 152.7 (qC, C-12), 131.7 (qC, C-8), 127.1 (CH, C-9), 119.1 (qC, C-4), 106.1 (CH_2_, C-21), 100.5 (qC, C-2), 81.9 (qC, C-10), 81.5 (CH, C-13), 56.3 (CH_3_, OMe), 52.5 (CH, C-11), 44.0 (qC, C-6), 36.2 (CH_2_, C-7), 29.8 (CH_2_, H-14), 27.6 (CH_3_, C-20), 25.7 (CH_3_, C-18), 23.9 (CH_3_, C-19), 10.5 (CH_3_, C-17), 10.5 (CH_3_, C-15), 7.4 (CH_3_, C-16) × 10^–6^; HR-ESIMS *m/z* 359.2216 [M + H]^+^ (calcd. for C_22_H_31_O_4_, 359.2217).

### X-ray crystallographic analysis for tridachiapyrone J (4)

C_22_H_30_O_5_, colorless crystal (m. p. 157–158 °C) was obtained from methanol at 4 °C. *M* = 374.46 g/mol, *T* = 293(2) K, *λ* = 1.54178 Å, Space group *P* 21, *a* = 7.8138(2) Å, *b* = 14.6718(3) Å, *c* = 9.9809(2) Å, *α* = 90°, *β* = 107.8670(10)°, *γ* = 90°, *V* = 1089.05(4) Å^3^, *Z* = 2, *D*_calcd_ = 1.142 Mg/m^3^, *m* = 0.646 mm^−1^, F(000) = 404. The final *R*_1_ = 0.0358, w*R*_2_ = 0.0940. Absolute structure parameter − 0.07(6). The X-ray measurements were made on a Bruker D8 Venture X-ray diffractometer with Cu K*α* radiation. The structure was solved with the ShelXT structure solution program using Intrinsic Phasing and refined with the ShelXL refinement package using Least Squares minimization. The above crystal data were deposited in the Cambridge Crystallographic Data Centre (CCDC) and assigned the accession number (CCDC 1953946). Copies of the data can be obtained free of charge via www.ccdc.cam.ac.uk/data request/cif or from the Cambridge Crystallographic Data Centre, 12 Union Road, Cambridge CB21EZ, UK. [Fax: (+ 44) 1223–336-033. E-mail: deposit@ccdc.cam.ac.uk.].

### QM-NMR calculation of compounds 1 and 11

All calculations followed the general protocols previously described for DP4 + (Grimblat et al. 2015). Conformational searches were performed in the gas phase applying the MMFF94S force field. NMR calculations were undertaken on structures above 1% Boltzmann population. A theory level of B3LYP/6-31G* was used to optimize structures. The GIAO methodology was used to calculate magnetic shielding constants (σ) at the mPW1PW91/6-31G(d) level of theory as recommended for DP4 + .

### TDDFT-ECD calculation of compounds 11 and 12

Torsional sampling (MCMM) conformational searches using MMFF94S force field were carried out by the means of conformational search module in Macro model 9.9.223 software (Schrodinger, http://www.schrodinger.com/MacroModel), applying an energy window of 21 kJ/mol (5.02 kcal/mol) for saving structures. The dominant conformers with over 1% Boltzmann population were used for re-optimization and the following TDDFT-ECD calculation. The re-optimizations and the TDDFT-ECD calculations were performed with Gaussian 09 (Gaussian, http://www.Gaussian.com) at the same B3LYP/6-311G(d,p) level with the IEFPCM solvent model for acetonitrile. Finally, the SpecDis 1.62 software (Bruhn et al. 2013) was used to obtain the calculated ECD spectrum and visualize the results.

### Antibacterial activity assays

The human pathogens *Staphylococcus aureus* ATCC27154 and *Pseudomonas aeruginosa* ATCC10145 were donated by the Korea Institute of Science and Technology. The marine strains *Streptococcus parauberis* KSP28, *Lactococcus garvieae* MP5245, *Aeromonas salmonicida* AS42, and *Photobacterium halotolerans* LMG22194T were provided by the National Fisheries Research & Development Institute, Korea.

MIC values of the compounds were determined by the modified 0.5 McFarland standard method. Twofold dilutions of the compounds were prepared in 5% DMSO. The turbidity of the bacterial suspensions was measured at 600 nm and adjusted with medium to match the 0.5 McFarland standards (10^5^−10^6^ colony forming units/mL). Subsequently, 95 μL of bacterial culture was added to each well of a 96-well plate followed by the addition of the test solutions (5 μL). Finally, the plates were incubated at 37° for 12 h, and the MIC values were determined in triplicates. To ensure that the vehicles had no significant effect on the bacterial growth, each bacterial species was additionally cultured with vehicle solution containing LB broth media at concentrations equivalent to those of the test solutions.

## Supplementary Information

Below is the link to the electronic supplementary material.

Supplementary file1 (DOCX 11167 KB)

## Data Availability

The data that supports the findings of this study are included in this published article (and its supplementary information file).
