# Supplementary material for: Ocellatuspyrones A‒G, new antibacterial polypropionates from the Chinese mollusk Placobranchus ocellatus
Source: Mar Life Sci Technol. 2023 Jul 6;5(3):373–86. doi: 10.1007/s42995-023-00179-w (PMC10449759; doi:10.1007/s42995-023-00179-w)
Supplement: Supplementary file 1 — Supplementary file1 (DOCX 11167 KB) [file 42995_2023_179_MOESM1_ESM.docx]

*Supporting Information*

**Ocellatuspyrones A‒G, new antibacterial polypropionates from the Chinese mollusk *Placobranchus ocellatus***

Song-Wei Li^1^ Dan-Dan Yu^4^ Ming-Zhi Su^4^ Li-Gong Yao^2^ Hong Wang^1,^* Xueting Liu^3,^* Yue-Wei Guo^1,2,4,^*

^1^Collaborative Innovation Center of Yangtze River Delta Region Green Pharmaceuticals, College of Pharmaceutical Science, Zhejiang University of Technology, Hangzhou 310014, China

^2^State Key Laboratory of Drug Research, Shanghai Institute of Materia Medica, Chinese Academy of Sciences, Shanghai 201203, China

^3^State Key Laboratory of Bioreactor Engineering, East China University of Science and Technology, Shanghai 200237, China

^4^Shandong Laboratory of Yantai Drug Discovery, Bohai Rim Advanced Research Institute for Drug Discovery, Yantai 264117, China

*Corresponding authors:

Hong Wang

E-mail: [hongw@zjut.edu.cn](mailto:hongw@zjut.edu.cn); Phone (Fax): CN +86-0571-88813986

Xueting Liu

E-mail: [liuxueting@ecust.edu.cn](mailto:liuxueting@ecust.edu.cn); Phone (Fax): CN +86-021-64253020

Yue-Wei Guo

E-mail: [ywguo@simm.ac.cn](mailto:ywguo@simm.ac.cn); Phone (Fax): CN +86-021-50805813

**Contents**

**Table S1**. X-ray crystallographic data for tridachiapyrone J (**4**)5

**Figure S1a**. Chiral HPLC analysis chromatography of (±)-ocellatuspyrone A (**1**)6

**Figure S1b**. Specific optical rotation of (±)-ocellatuspyrone A (**1**)6

**Figure S1c**. Specific optical rotation of (−)-ocellatuspyrone A (**1**)6

**Figure S1d**. Specific optical rotation of (+)-ocellatuspyrone A (**1**)6

**Figure S1e**. ^1^H NMR spectrum (600 MHz) of (±)-ocellatuspyrone A (**1**) in CDCl_3_7

**Figure S1f**. ^13^C NMR spectrum (150 MHz) of (±)-ocellatuspyrone A (**1**) in CDCl_3_7

**Figure S1g**. HSQC spectrum (600 MHz) of (±)-ocellatuspyrone A (**1**) in CDCl_3_8

**Figure S1h**. HMBC spectrum (600 MHz) of (±)-ocellatuspyrone A (**1**) in CDCl_3_8

**Figure S1i**. ^1^H-^1^H COSY spectrum (600 MHz) of (±)-ocellatuspyrone A (**1**) in CDCl_3_9

**Figure S1j**. NOESY spectrum (600 MHz) of (±)-ocellatuspyrone A (**1**) in CDCl_3_9

**Figure S1k**. HR-ESIMS (positive mode) spectrum of (±)-ocellatuspyrone A (**1**)10

**Figure S1l**. IR spectrum of (±)-ocellatuspyrone A (**1**)10

**Figure S1m**. ECD and UV spectra of (±)-ocellatuspyrone A (**1**)10

**Figure S1n** ^1^H NMR spectrum (400 MHz) of (*S*)-MTPA-(+)-**1** in CDCl_3_11

**Figure S1o**. ^1^H NMR spectrum (400 MHz) of (*R*)-MTPA-(+)-**1** in CDCl_3_11

**Figure S1p**. ^1^H NMR spectrum (400 MHz) of (*S*)-MTPA-(−)-**1** in CDCl_3_12

**Figure S1q**. ^1^H NMR spectrum (400 MHz) of (*R*)-MTPA-(−)-**1** in CDCl_3_12

**Figure S1r**. ECD and UV spectra of (*S*)-MTPA-(+)-**1**13

**Figure S1s**. ECD and UV spectra of (*R*)-MTPA-(+)-**1**13

**Figure S1t**. ECD and UV spectra of (*S*)-MTPA-(−)-**1**13

**Figure S1u**. ECD and UV spectra of (*R*)-MTPA-(−)-**1**14

**Figure S2a**. ^1^H NMR spectrum (600 MHz) of (±)-ocellatuspyrone B (**2**) in CDCl_3_15

**Figure S2b**. ^13^C NMR spectrum (150 MHz) of (±)-ocellatuspyrone B (**2**) in CDCl_3_15

**Figure S2c**. HSQC spectrum (600 MHz) of (±)-ocellatuspyrone B (**2**) in CDCl_3_16

**Figure S2d**. HMBC spectrum (600 MHz) of (±)-ocellatuspyrone B (**2**) in CDCl_3_16

**Figure S2e**. ^1^H-^1^H COSY spectrum (600 MHz) of (±)-ocellatuspyrone B (**2**) in CDCl_3_17

**Figure S2f**. NOESY spectrum (600 MHz) of (±)-ocellatuspyrone B (**2**) in CDCl_3_17

**Figure S2g**. HR-ESIMS (positive mode) spectrum of (±)-ocellatuspyrone B (**2**)18

**Figure S2h**. IR spectrum of (±)-ocellatuspyrone B (**2**)18

**Figure S2i**. ECD and UV spectra of (±)-ocellatuspyrone B (**2**)18

**Figure S2j**. Specific optical rotation of (±)-ocellatuspyrone B (**2**)18

**Figure S3a**. ^1^H NMR spectrum (600 MHz) of ocellatuspyrone C (**5**) in CDCl_3_19

**Figure S3b**. ^13^C NMR spectrum (150 MHz) of ocellatuspyrone C (**5**) in CDCl_3_19

**Figure S3c**. HSQC spectrum (600 MHz) of ocellatuspyrone C (**5**) in CDCl_3_20

**Figure S3d**. HMBC spectrum (600 MHz) of ocellatuspyrone C (**5**) in CDCl_3_20

**Figure S3e**. ^1^H-^1^H COSY spectrum (600 MHz) of ocellatuspyrone C (**5**) in CDCl_3_21

**Figure S3f**. NOESY spectrum (600 MHz) of ocellatuspyrone C (**5**) in CDCl_3_21

**Figure S3g**. HR-ESIMS (positive mode) spectrum of ocellatuspyrone C (**5**)22

**Figure S3h**. IR spectrum of ocellatuspyrone C (**5**)22

**Figure S3i**. ECD and UV spectra of ocellatuspyrone C (**5**)22

**Figure S3j**. Specific optical rotation of ocellatuspyrone C (**5**)22

**Figure S4a**. ^1^H NMR spectrum (600 MHz) of ocellatuspyrone D (**9**) in CDCl_3_23

**Figure S4b**. ^13^C NMR spectrum (150 MHz) of ocellatuspyrone D (**9**) in CDCl_3_23

**Figure S4c**. HSQC spectrum (600 MHz) of ocellatuspyrone D (**9**) in CDCl_3_24

**Figure S4d**. HMBC spectrum (600 MHz) of ocellatuspyrone D (**9**) in CDCl_3_24

**Figure S4e**. ^1^H-^1^H COSY spectrum (600 MHz) of ocellatuspyrone D (**9**) in CDCl_3_25

**Figure S4f**. NOESY spectrum (600 MHz) of ocellatuspyrone D (**9**) in CDCl_3_25

**Figure S4g**. HR-ESIMS (positive mode) spectrum of ocellatuspyrone D (**9**)26

**Figure S4h**. IR spectrum of ocellatuspyrone D (**9**)26

**Figure S4i**. ECD and UV spectra of ocellatuspyrone D (**9**)26

**Figure S4j**. Specific optical rotation of ocellatuspyrone D (**9**)26

**Figure S5a**. ^1^H NMR spectrum (600 MHz) of ocellatuspyrone E (**10**) in CDCl_3_27

**Figure S5b**. ^13^C NMR spectrum (150 MHz) of ocellatuspyrone E (**10**) in CDCl_3_27

**Figure S5c**. HSQC spectrum (600 MHz) of ocellatuspyrone E (**10**) in CDCl_3_28

**Figure S5d**. HMBC spectrum (600 MHz) of ocellatuspyrone E (**10**) in CDCl_3_28

**Figure S5e**. ^1^H-^1^H COSY spectrum (600 MHz) of ocellatuspyrone E (**10**) in CDCl_3_29

**Figure S5f**. NOESY spectrum (600 MHz) of ocellatuspyrone E (**10**) in CDCl_3_29

**Figure S5g**. HR-ESIMS (positive mode) spectrum of ocellatuspyrone E (**10**)30

**Figure S5h**. IR spectrum of ocellatuspyrone E (**10**)30

**Figure S5i**. ECD and UV spectra of ocellatuspyrone E (**10**)30

**Figure S5j**. Specific optical rotation of ocellatuspyrone E (**10**)30

**Figure S6a**. ^1^H NMR spectrum (600 MHz) of ocellatuspyrone F (**11**) in CDCl_3_31

**Figure S6b**. ^13^C NMR spectrum (150 MHz) of ocellatuspyrone F (**11**) in CDCl_3_31

**Figure S6c**. HSQC spectrum (600 MHz) of ocellatuspyrone F (**11**) in CDCl_3_32

**Figure S6d**. HMBC spectrum (600 MHz) of ocellatuspyrone F (**11**) in CDCl_3_32

**Figure S6e.** ^1^H-^1^H COSY spectrum (600 MHz) of ocellatuspyrone F (**11**) in CDCl_3_33

**Figure S6f**. NOESY spectrum (600 MHz) of ocellatuspyrone F (**11**) in CDCl_3_33

**Figure S6g**. HR-ESIMS (positive mode) spectrum of ocellatuspyrone F (**11**)34

**Figure S6h**. IR spectrum of ocellatuspyrone F (**11**)34

**Figure S6i**. ECD and UV spectra of ocellatuspyrone F (**11**)34

**Figure S6j**. Specific optical rotation of ocellatuspyrone F (**11**)34

**Figure S7a**. ^1^H NMR spectrum (600 MHz) of ocellatuspyrone G (**12**) in CDCl_3_35

**Figure S7b**. ^13^C NMR spectrum (150 MHz) of ocellatuspyrone G (**12**) in CDCl_3_35

**Figure S7c**. HSQC spectrum (600 MHz) of ocellatuspyrone G (**12**) in CDCl_3_36

**Figure S7d.** HMBC spectrum (600 MHz) of ocellatuspyrone G (**12**) in CDCl_3_36

**Figure S7e**. ^1^H-^1^H COSY spectrum (600 MHz) of ocellatuspyrone G (**12**) in CDCl_3_37

**Figure S7f**. NOESY spectrum (600 MHz) of ocellatuspyrone G (**12**) in CDCl_3_37

**Figure S7g**. HR-ESIMS (positive mode) spectrum of ocellatuspyrone G (**12**)38

**Figure S7h**. IR spectrum of ocellatuspyrone G (**12**)38

**Figure S7i**. ECD and UV spectra of ocellatuspyrone G (**12**)38

**Figure S7j**. Specific optical rotation of ocellatuspyrone G (**12**)38

**Figure S8**. The comparison of ^1^H NMR spectrum (400 MHz) for the reduction product of **4** and the isolated **6** in CDCl_3_39

**Figure S9a**. ^1^H NMR spectrum (600 MHz) of compound **6a** in CDCl_3_39

**Figure S9b**. ^13^C NMR spectrum (150 MHz) of compound **6a** in CDCl_3_40

**Figure S9c**. HSQC spectrum (600 MHz) of compound **6a** in CDCl_3_40

**Figure S9d.** HMBC spectrum (600 MHz) of compound **6a** in CDCl_3_41

**Figure S9e**. ^1^H-^1^H COSY spectrum (600 MHz) of compound **6a** in CDCl_3_41

**Figure S9f**. NOESY spectrum (600 MHz) of compound **6a** in CDCl_3_42

**Figure S9g**. HR-ESIMS (positive mode) spectrum of compound **6a**42

**Figure S10a**. ^1^H NMR spectrum (600 MHz) of (±)-photodeoxytridachione (**3**) in CDCl_3_43

**Figure S10b**. ^13^C NMR spectrum (150 MHz) of (±)-photodeoxytridachione (**3**) in CDCl_3_43

**Figure S10c**. ECD and UV spectra of (±)-photodeoxytridachione (**3**)44

**Figure S11a**. ^1^H NMR spectrum (600 MHz) of tridachiapyrone J (**4**) in CDCl_3_44

**Figure S11b**. ^13^C NMR spectrum (150 MHz) of tridachiapyrone J (**4**) in CDCl_3_45

**Figure S11c**. ECD and UV spectra of tridachiapyrone J (**4**)45

**Figure S12a**. ^1^H NMR spectrum (600 MHz) of tridachiapyrone G (**6**) in CDCl_3_46

**Figure S12b**. ^13^C NMR spectrum (150 MHz) of tridachiapyrone G (**6**) in CDCl_3_46

**Figure S12c**. ECD and UV spectra of tridachiapyrone G (**6**)47

**Figure S13a**. ^1^H NMR spectrum (600 MHz) of tridachiapyrone H (**7**) in CDCl_3_47

**Figure S13b**. ECD and UV spectra of tridachiapyrone H (**7**)48

**Figure S14a**. ^1^H NMR spectrum (600 MHz) of (+)-9,10-deoxytridachione (**8**) in CDCl_3_48

**Figure S14b**. ^13^C NMR spectrum (150 MHz) of (+)-9,10-deoxytridachione (**8**) in CDCl_3_49

**Figure S14c**. ECD and UV spectra of (+)-9,10-deoxytridachione (**8**)49

**Figure S15a**. Structures of isomers **1a** and **1b** for compound **1**.50

**Figure S15b**. Structures of isomers **11a** and **11b** for compound **11**.50

**Figure S15c**. DP4+ results obtained using Exp. data of compound **1** *versus* isomers **1a** and **1b**.51

**Figure S15d**. DP4+ results obtained using Exp. data of compound **11** *versus* isomers **11a** and **11b**.52

**Figure S15e**. The Cartesian Coordinates of the dominant conformers for **1a** and **1b**.53

**Figure S15f**. The Cartesian Coordinates of the dominant conformers for **11a** and **11b**.64

**Figure S16**. The Cartesian Coordinates of the dominant conformers for **12**.78

**Figure S17a**. The HPLC analysis of residue (80%-100% CH_3_CN/H_2_0, 10 min, 3.0 mL/min), which was obtained after the treatment of Methylene Blue with compound **6** in CH_2_Cl_2_ at room temperature and exposed to sunlight for 2 hours.82

**Figure S17b**. The HPLC analysis of residue (80%-100% CH_3_CN/H_2_0, 10 min, 3.0 mL/min), which was obtained after the treatment of Methylene Blue and Rose Bengal with compound **8** in CH_2_Cl_2_ at room temperature and exposed to sunlight for 4 hours.83

**Figure S17c**. The HPLC analysis of residue (80%-100% CH_3_CN/H_2_0, 10 min, 3.0 mL/min), which was obtained after the treatment of trifluoroacetic acid (TFA) and/or silica gel with compound **8** in CH_2_Cl_2_ at room temperature overnight.84

**Table S2**. The antibacterial activity assays.85

**Table S3**. Neuroprotective effect of tested compounds on hydrogen peroxide (H_2_O_2_) induced SH-SY5Y cell damage.85

**Table S4**. The protein tyrosine phosphatase 1B (PTP1B) inhibitory activity assay.87

**Table S5**. The antiviral activity against 2019-nCoV RDRPro.87

**Table S6**. The ameliorative effect of tested compounds on lipopolysaccharide (LPS) induced cellular inflammation.87

**Table S1**. X-ray crystallographic data for tridachiapyrone J (**4**)

| Identification code | cu_d8v19519_0m |  |
| --- | --- | --- |
| Empirical formula | C22 H30 O5 |  |
| Formula weight | 374.46 |  |
| Temperature | 293(2) K |  |
| Wavelength | 1.54178 Å |  |
| Crystal system | Monoclinic |  |
| Space group | P 21 |  |
| Unit cell dimensions | a = 7.8138(2) Å | α= 90°. |
|  | b = 14.6718(3) Å | β= 107.8670(10)°. |
|  | c = 9.9809(2) Å | γ = 90°. |
| Volume | 1089.05(4) Å3 |  |
| Z | 2 |  |
| Density (calculated) | 1.142 Mg/m3 |  |
| Absorption coefficient | 0.646 mm-1 |  |
| F(000) | 404 |  |
| Crystal size | 0.200 x 0.160 x 0.140 mm3 |  |
| Theta range for data collection | 6.333 to 67.497°. |  |
| Index ranges | -8<=h<=9, -17<=k<=16, -11<=l<=11 |  |
| Reflections collected | 19767 |  |
| Independent reflections | 3820 [R(int) = 0.0334] |  |
| Completeness to theta = 67.679° | 97.7 % |  |
| Absorption correction | Semi-empirical from equivalents |  |
| Max. and min. transmission | 0.7533 and 0.6040 |  |
| Refinement method | Full-matrix least-squares on F2 |  |
| Data / restraints / parameters | 3820 / 1 / 256 |  |
| Goodness-of-fit on F2 | 1.036 |  |
| Final R indices [I>2sigma(I)] | R1 = 0.0358, wR2 = 0.0940 |  |
| R indices (all data) | R1 = 0.0371, wR2 = 0.0953 |  |
| Absolute structure parameter | -0.07(6) |  |
| Extinction coefficient | 0.023(6) |  |
| Largest diff. peak and hole | 0.122 and -0.113 e.Å-3 |  |

**Figure S1a**. Chiral HPLC analysis chromatography of (±)-ocellatuspyrone A (**1**)


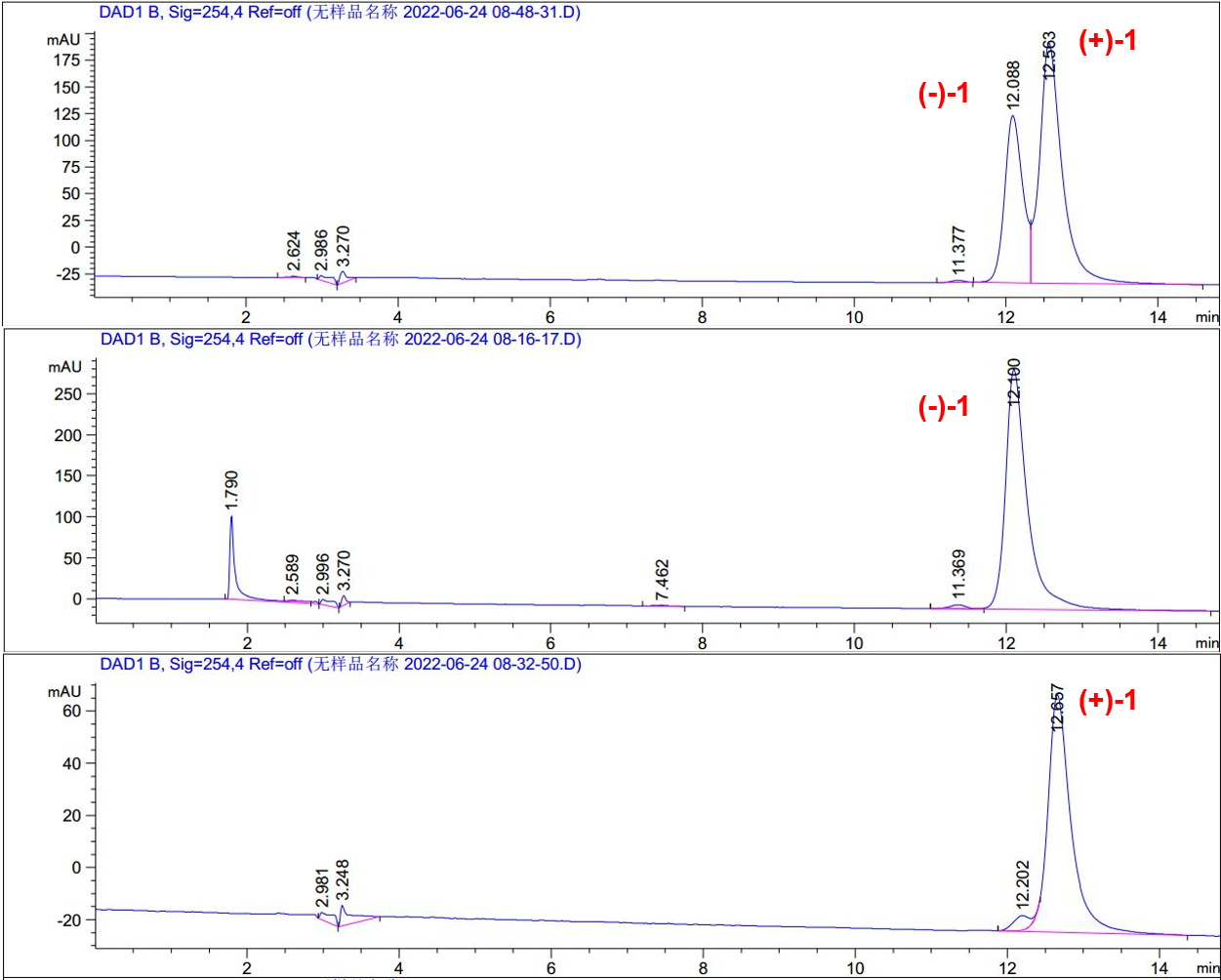


**Figure S1b**. Specific optical rotation of (±)-ocellatuspyrone A (**1**)


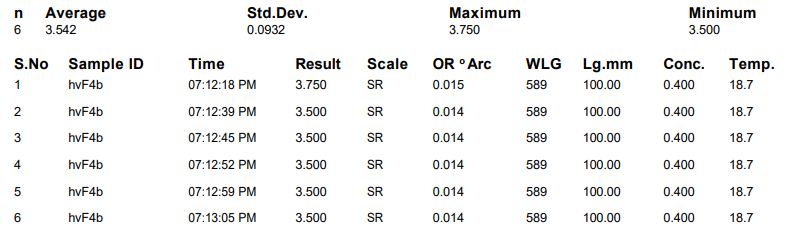


**Figure S1c**. Specific optical rotation of (−)-ocellatuspyrone A (**1**)


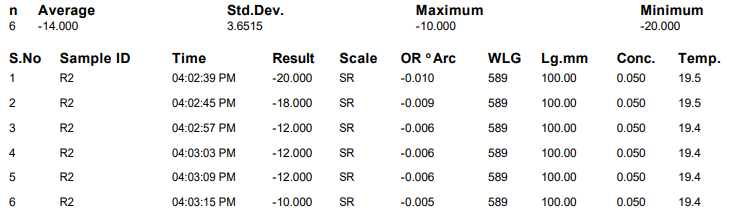


**Figure S1d**. Specific optical rotation of (+)-ocellatuspyrone A (**1**)


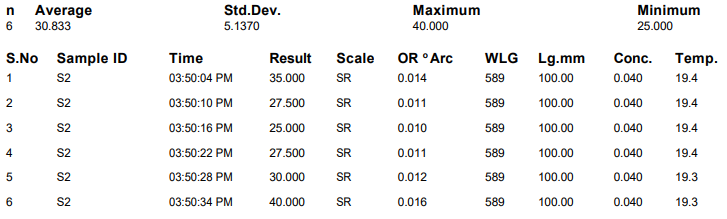


**Figure S1e**. ^1^H NMR spectrum (600 MHz) of (±)-ocellatuspyrone A (**1**) in CDCl_3_


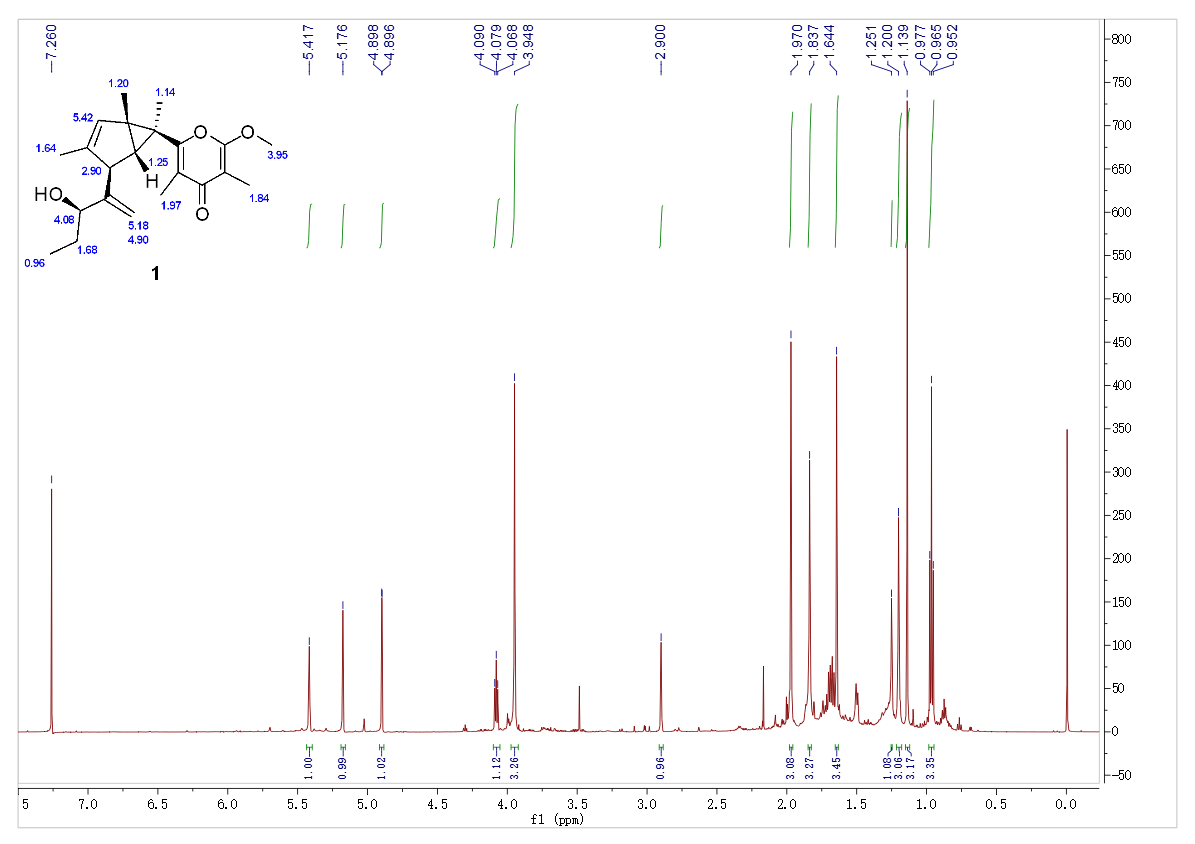


**Figure S1f**. ^13^C NMR spectrum (150 MHz) of (±)-ocellatuspyrone A (**1**) in CDCl_3_


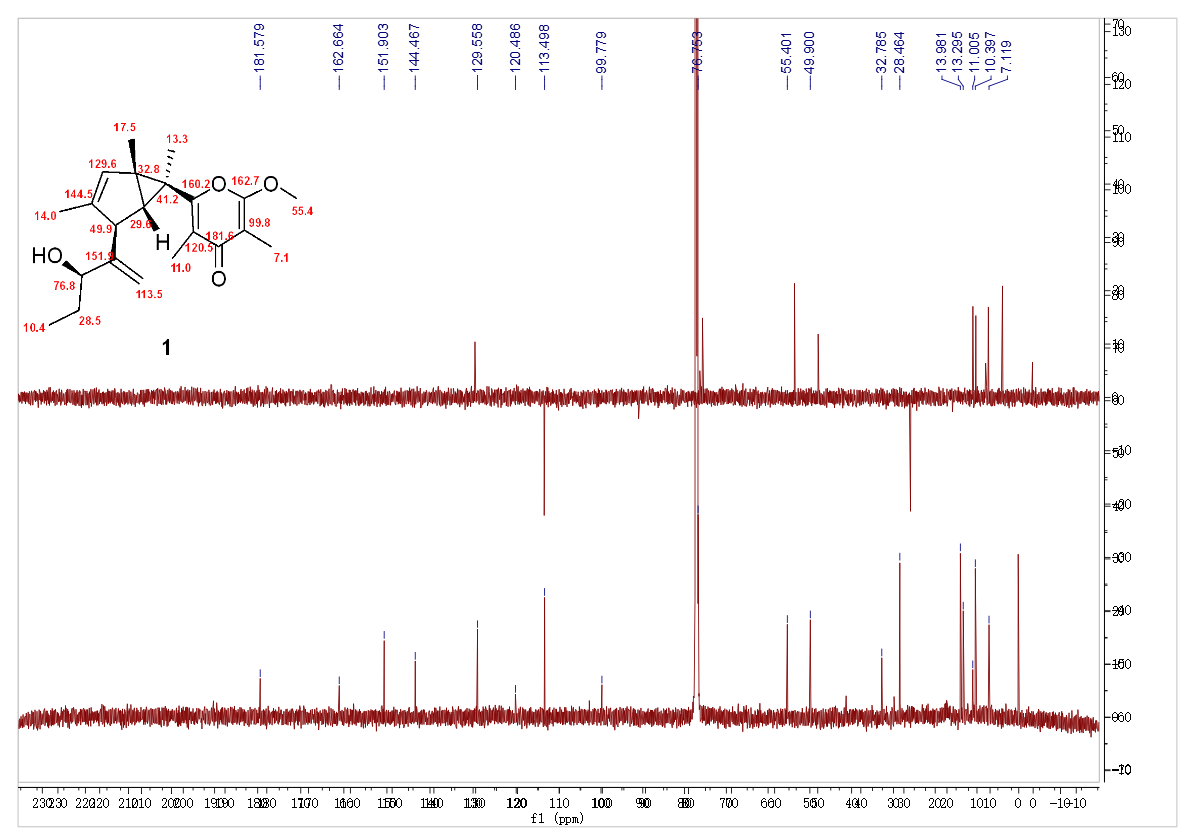


**Figure S1g**. HSQC spectrum (600 MHz) of (±)-ocellatuspyrone A (**1**) in CDCl_3_


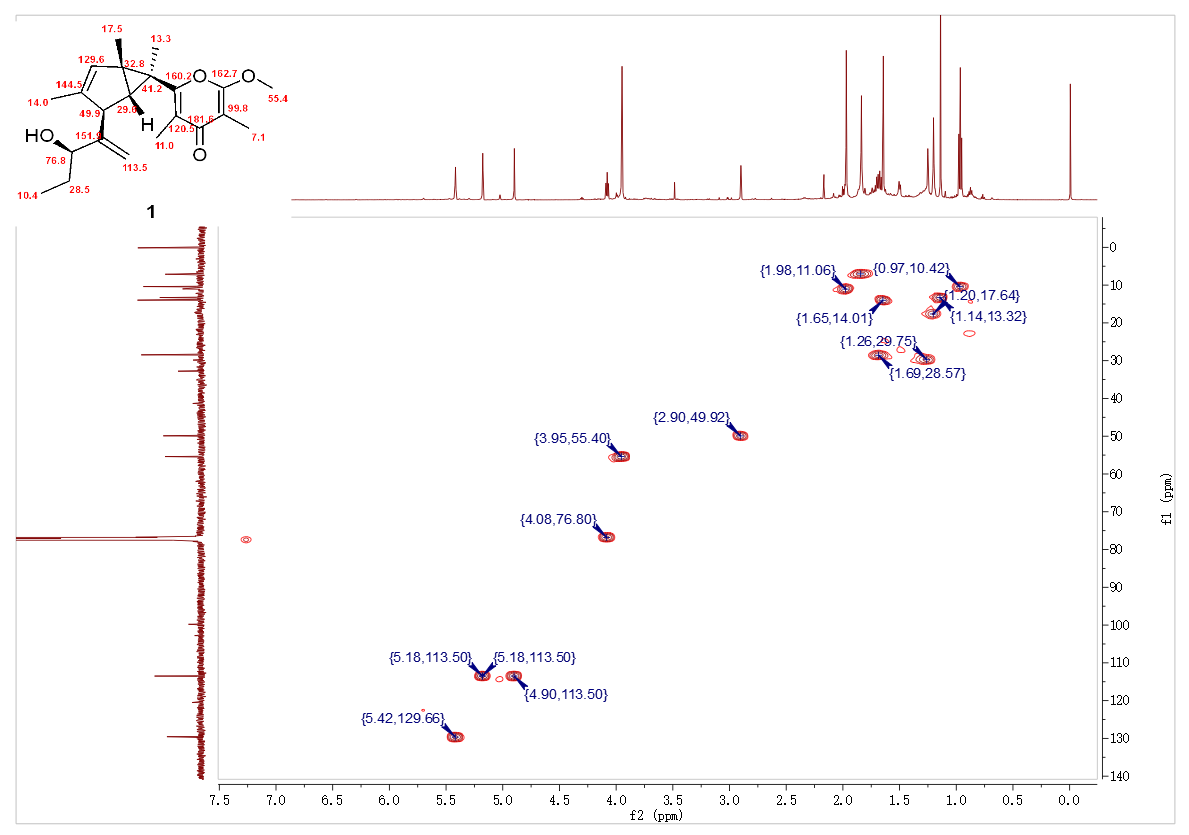


**Figure S1h**. HMBC spectrum (600 MHz) of (±)-ocellatuspyrone A (**1**) in CDCl_3_

**
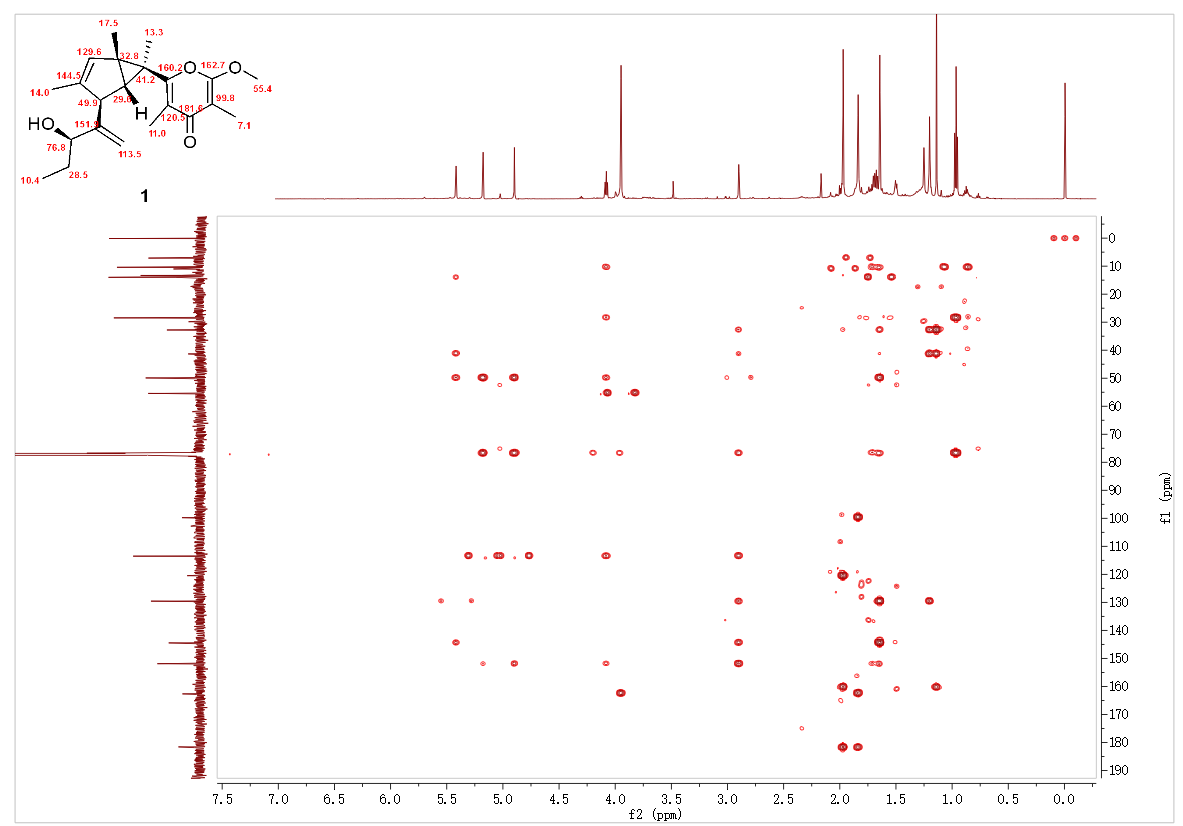
**

**Figure S1i**. ^1^H-^1^H COSY spectrum (600 MHz) of (±)-ocellatuspyrone A (**1**) in CDCl_3_

**
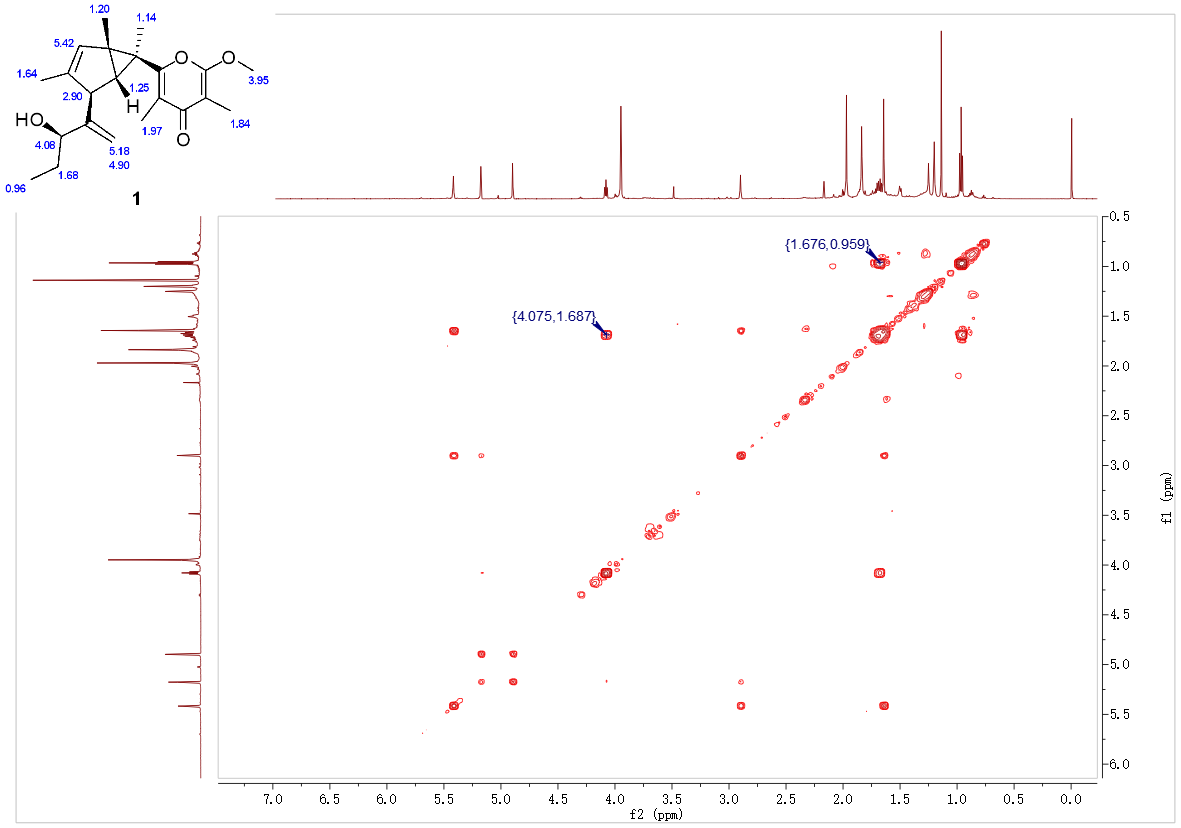
**

**Figure S1j**. NOESY spectrum (600 MHz) of (±)-ocellatuspyrone A (**1**) in CDCl_3_

**
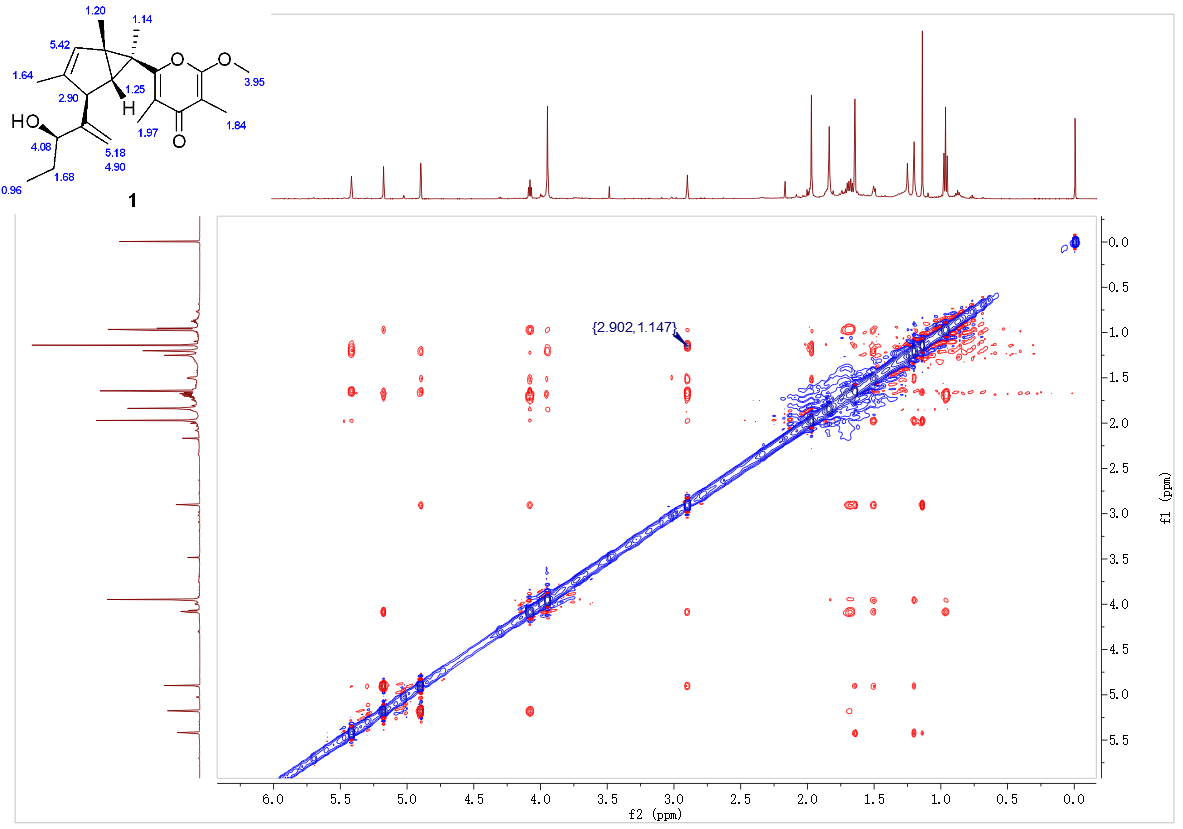
**

**Figure S1k**. HR-ESIMS (positive mode) spectrum of (±)-ocellatuspyrone A (**1**)

**
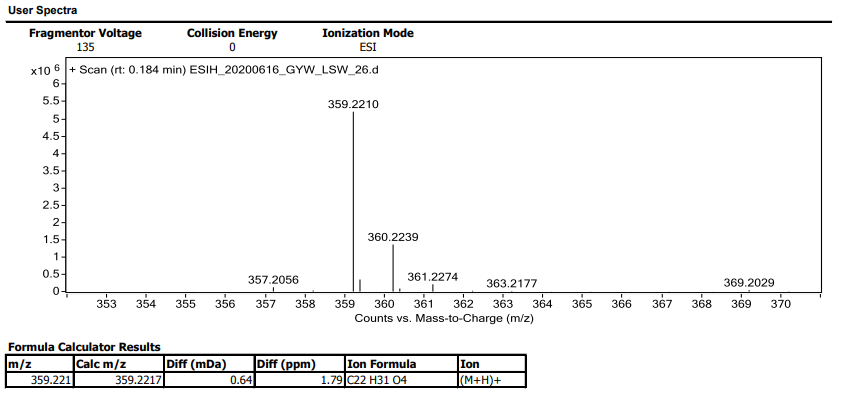
**

**Figure S1l**. IR spectrum of (±)-ocellatuspyrone A (**1**)


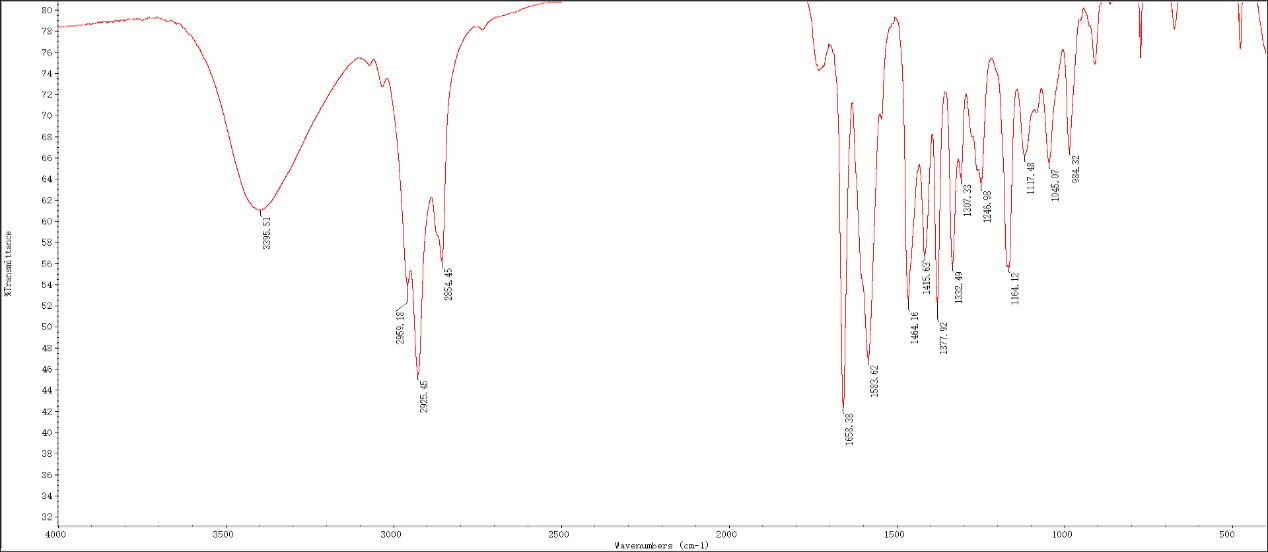


**Figure S1m**. ECD and UV spectra of (±)-ocellatuspyrone A (**1**)


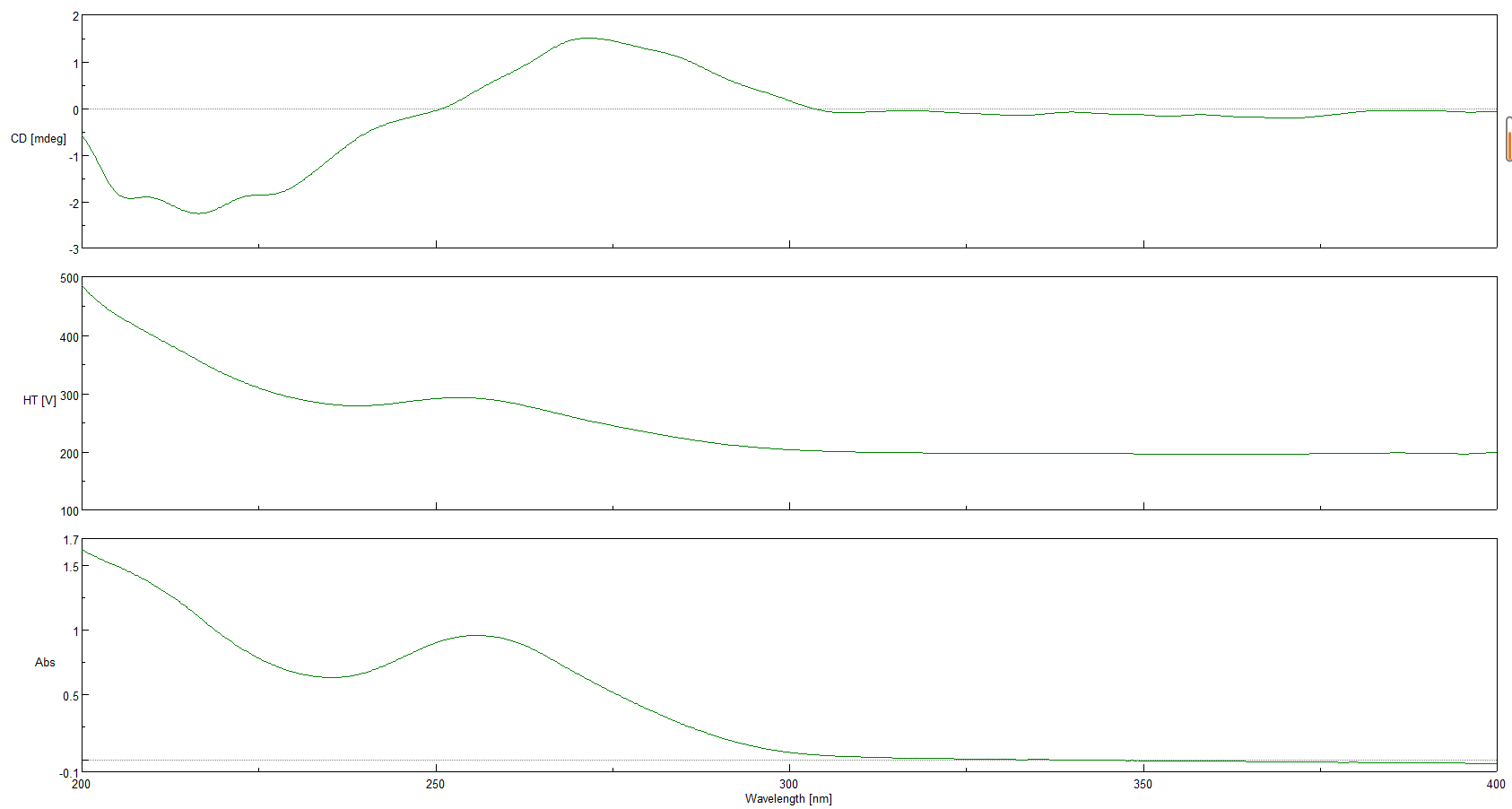


**Figure S1n**. ^1^H NMR spectrum (400 MHz) of (*S*)-MTPA-(+)-**1** in CDCl_3_

**
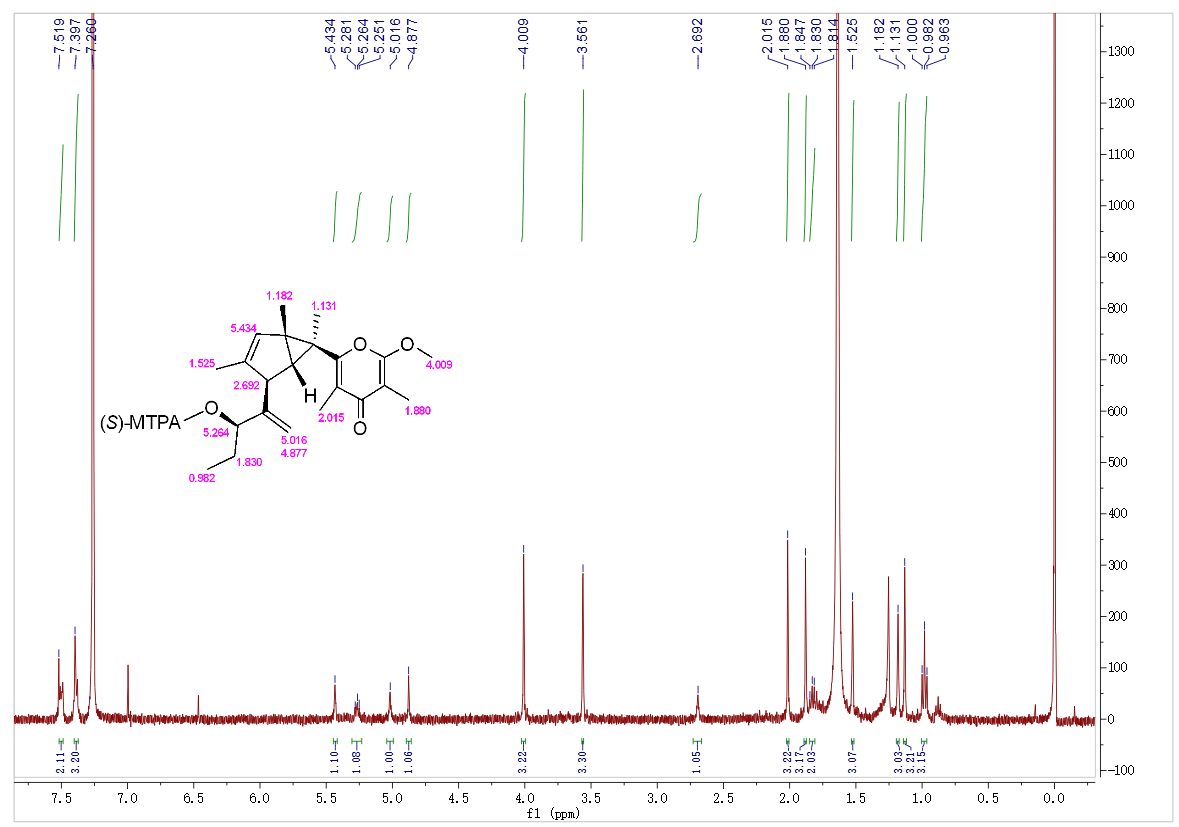
**

**Figure S1o**. ^1^H NMR spectrum (400 MHz) of (*R*)-MTPA-(+)-**1** in CDCl_3_

**
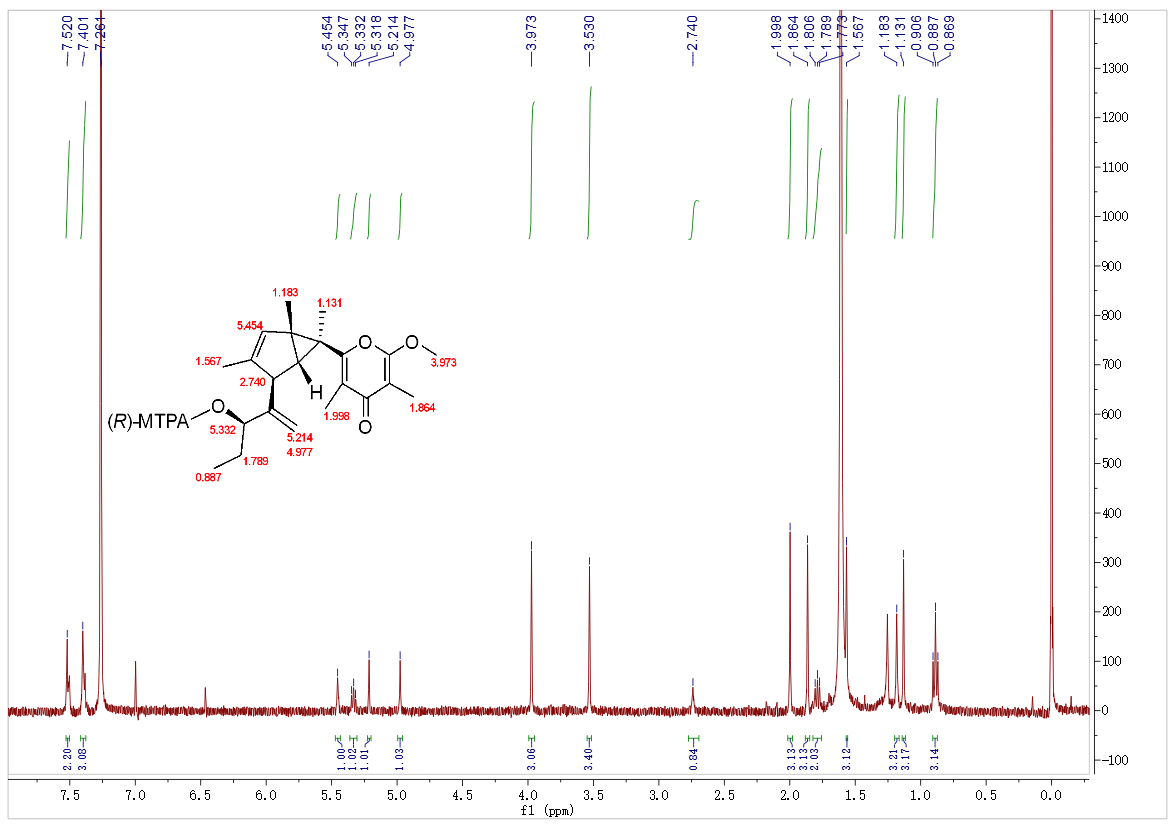
**

**Figure S1p**. ^1^H NMR spectrum (400 MHz) of (*S*)-MTPA-(-)-**1** in CDCl_3_

**
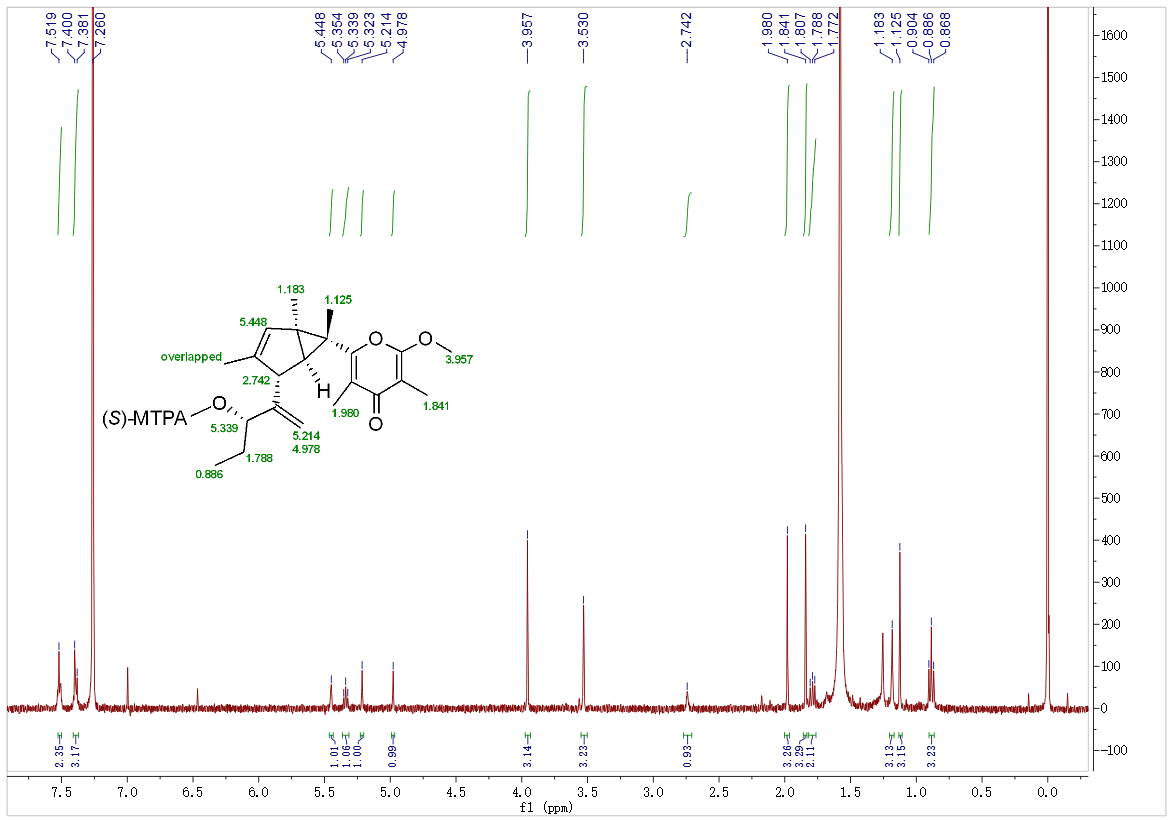
**

**Figure S1q**. ^1^H NMR spectrum (400 MHz) of (*R*)-MTPA-(-)-**1** in CDCl_3_

**
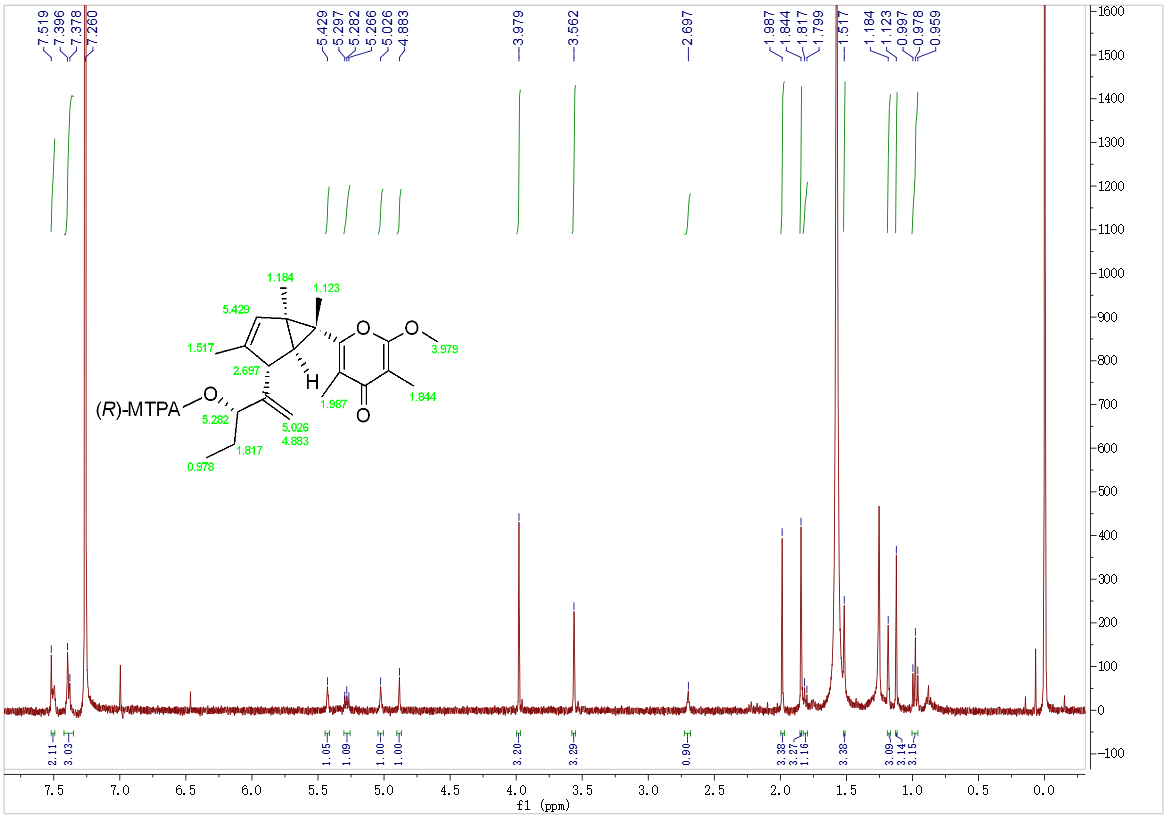
**

**Figure S1r**. ECD and UV spectra of (*S*)-MTPA-(+)-**1**


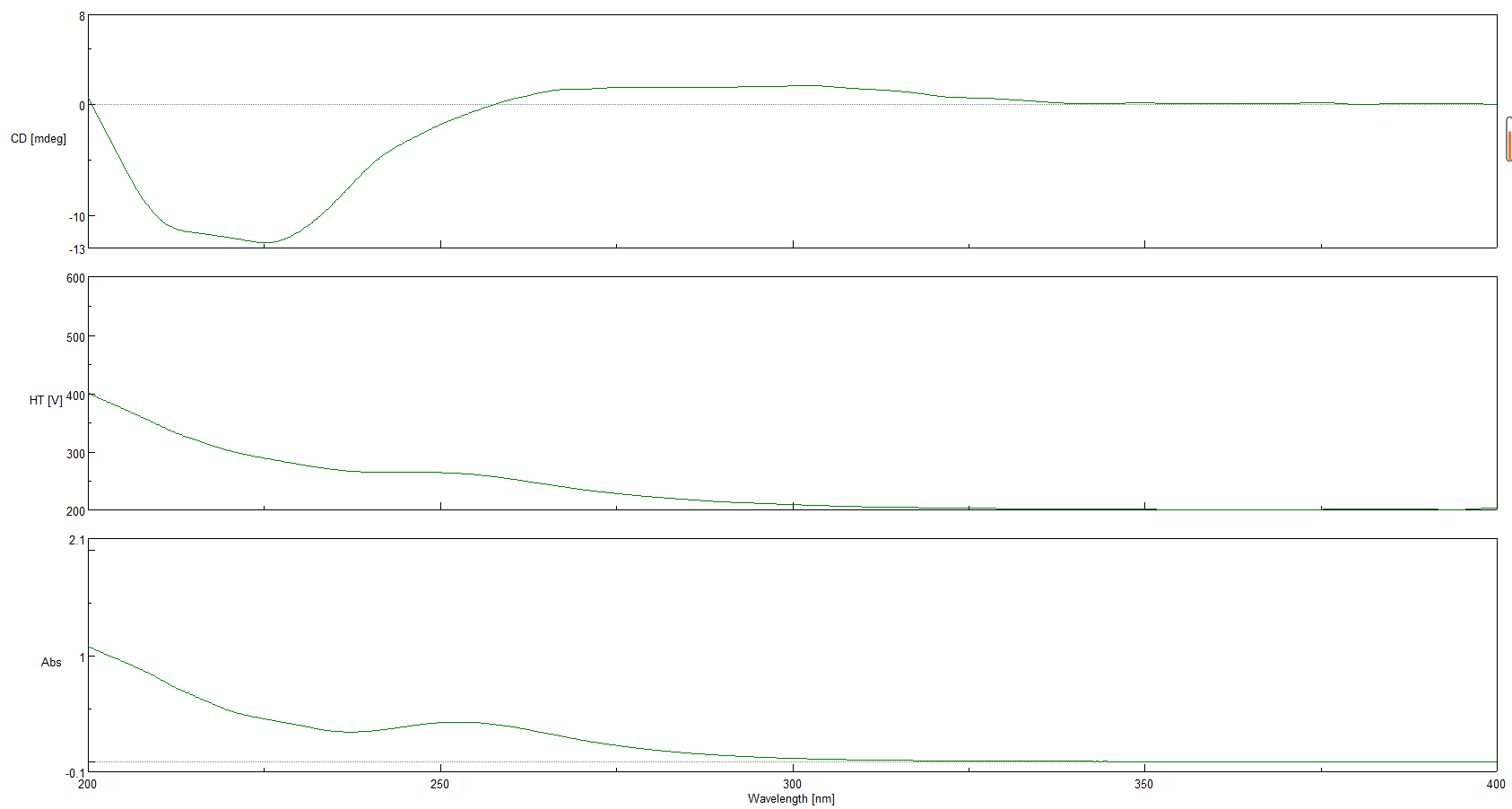


**Figure S1s**. ECD and UV spectra of (*R*)-MTPA-(+)-**1**


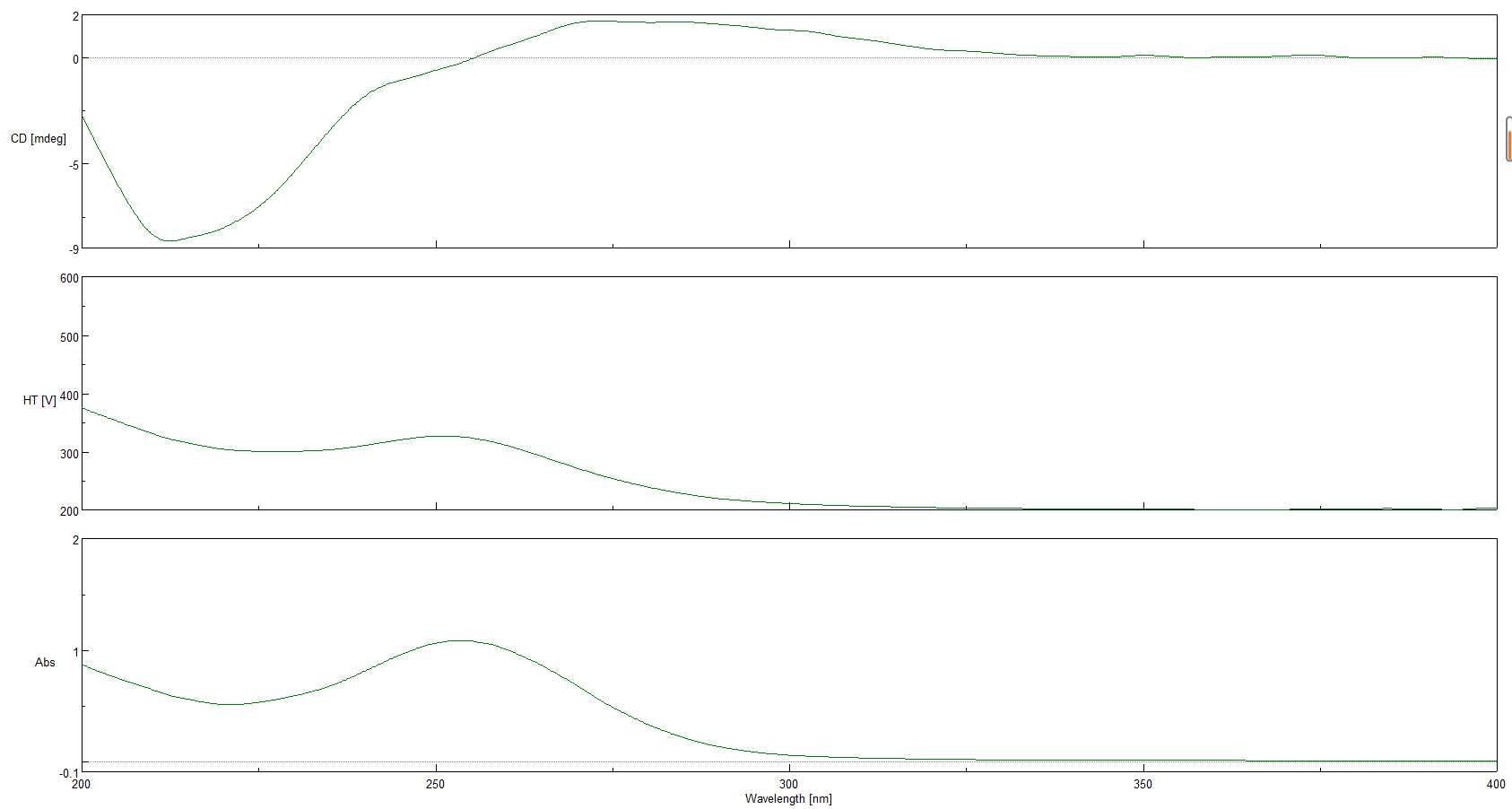


**Figure S1t**. ECD and UV spectra of (*S*)-MTPA-(−)-**1**


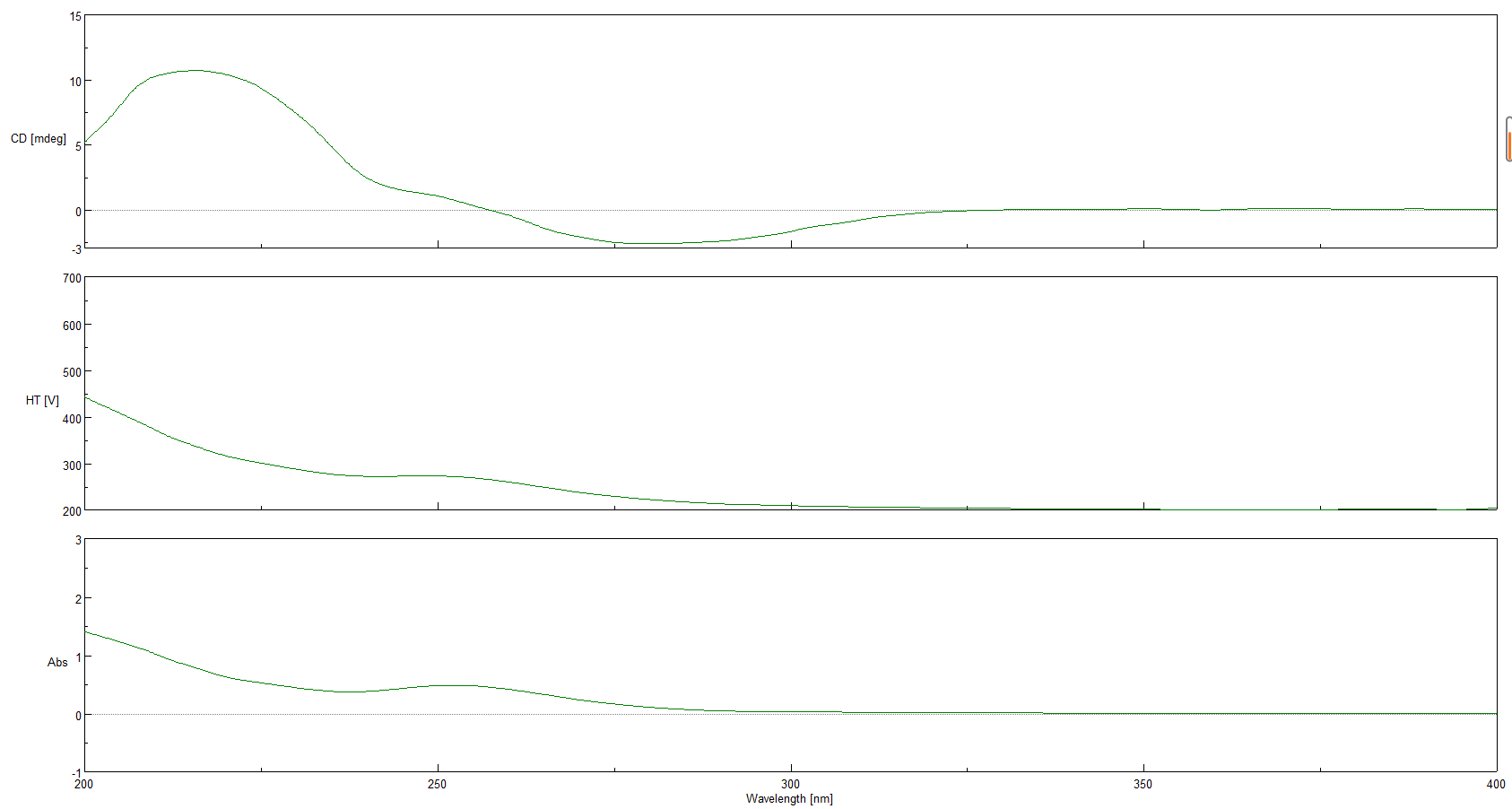


**Figure S1u**. ECD and UV spectra of (*R*)-MTPA-(−)-**1**


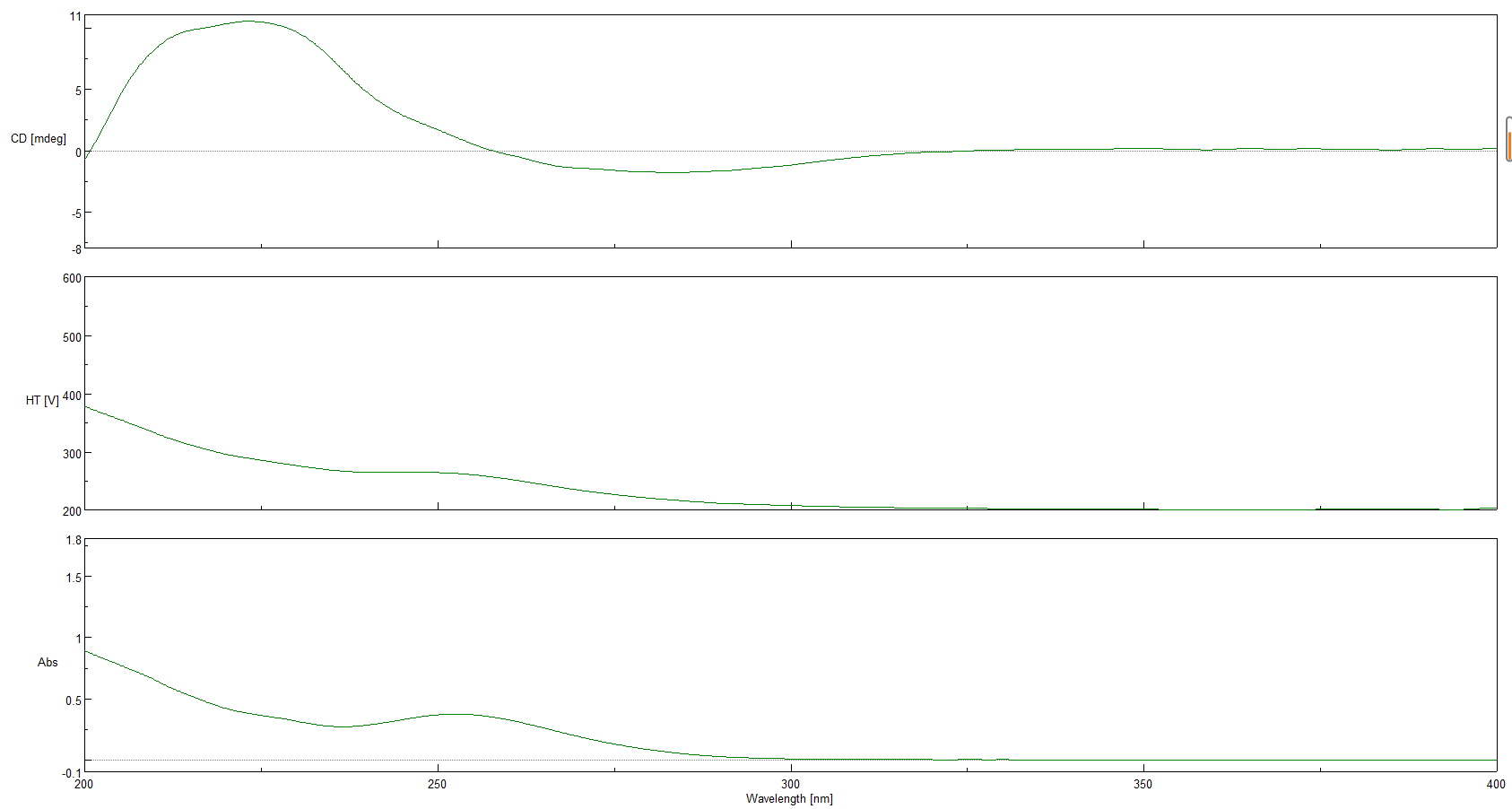


**Figure S2a**. ^1^H NMR spectrum (600 MHz) of (±)-ocellatuspyrone B (**2**) in CDCl_3_

**
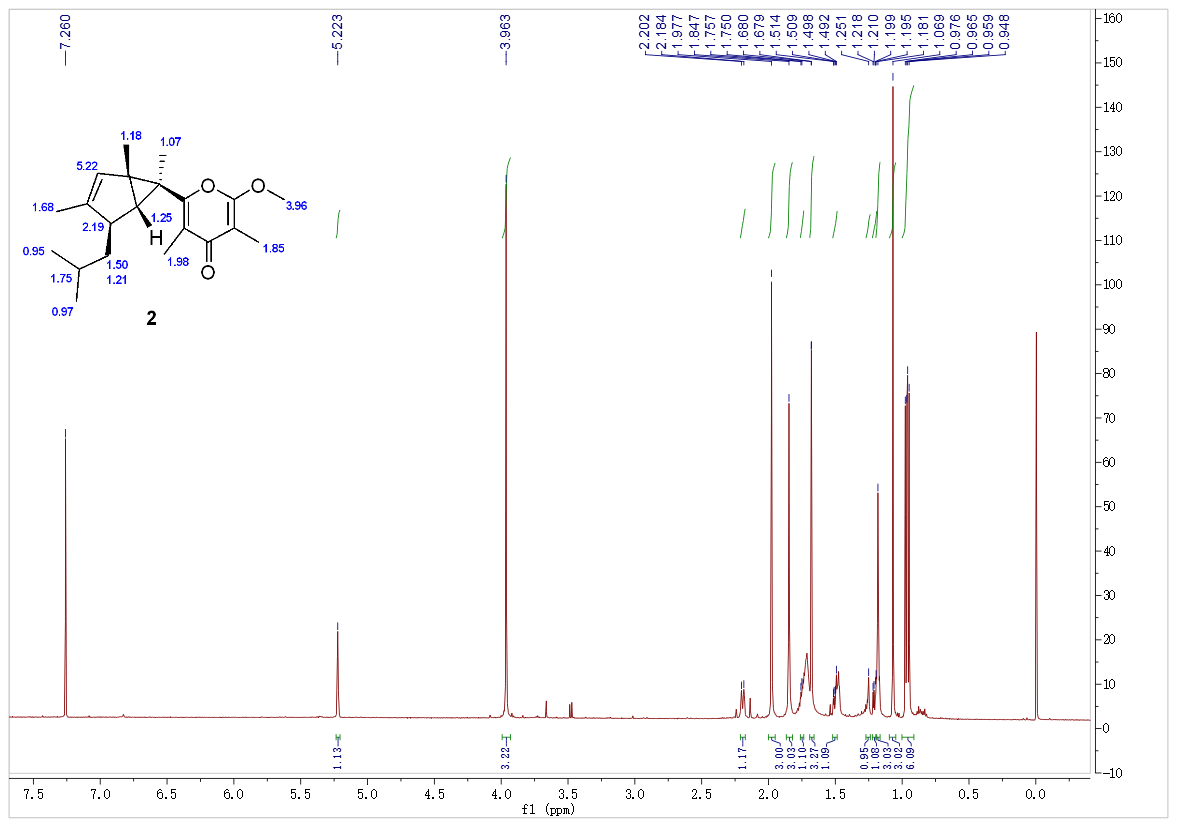
**

**Figure S2b**. ^13^C NMR spectrum (150 MHz) of (±)-ocellatuspyrone B (**2**) in CDCl_3_

**
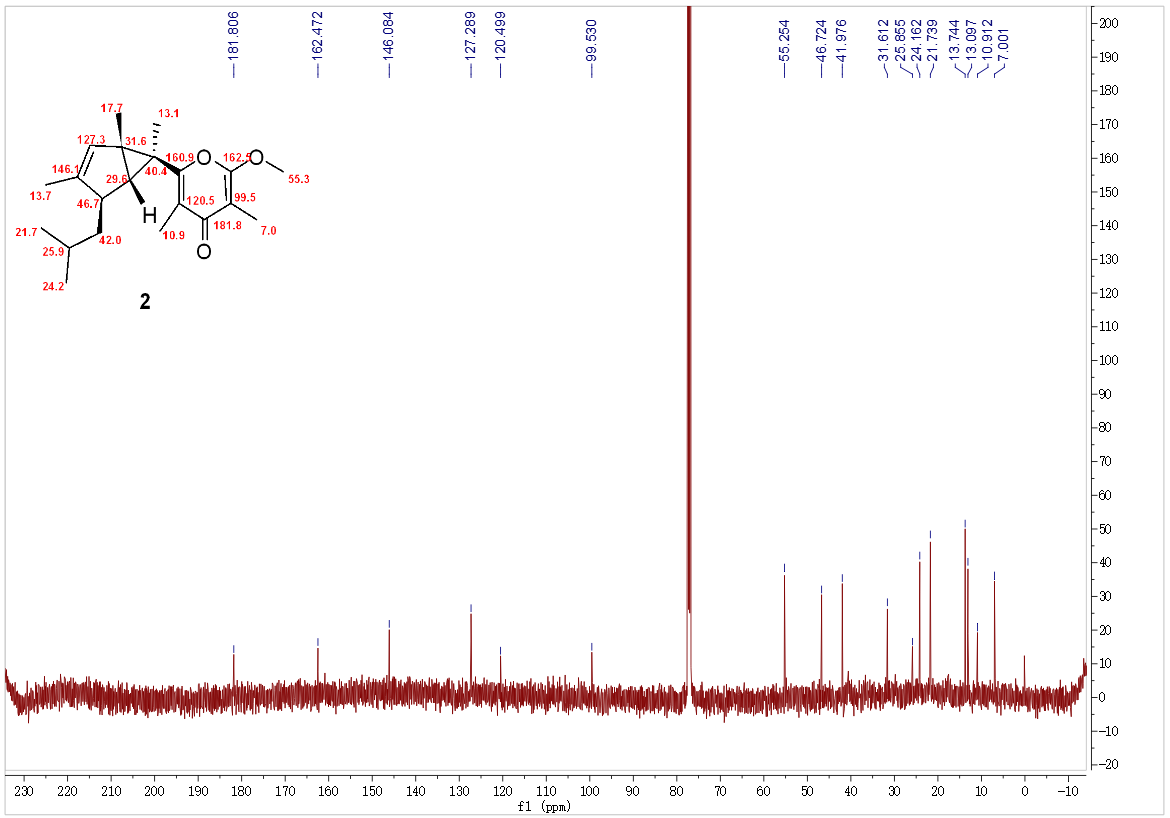
**

**Figure S2c**. HSQC spectrum (600 MHz) of (±)-ocellatuspyrone B (**2**) in CDCl_3_

**
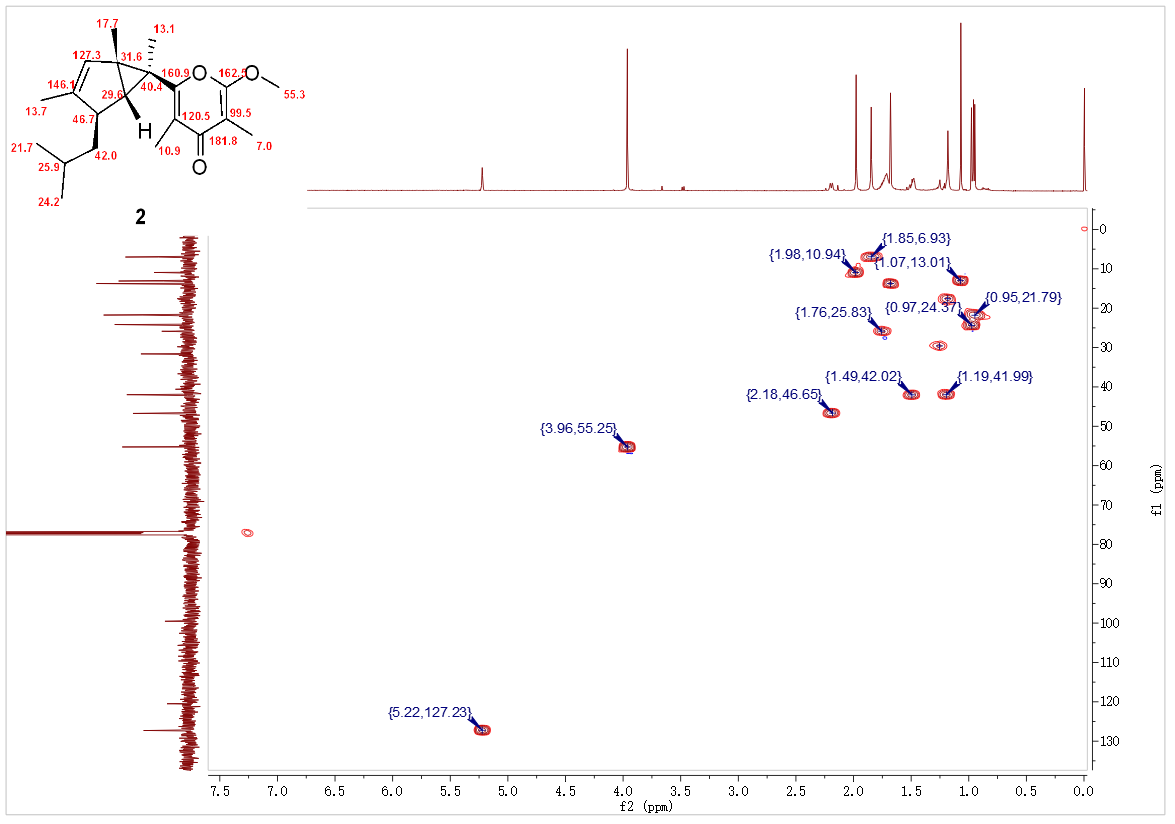
**

**Figure S2d**. HMBC spectrum (600 MHz) of (±)-ocellatuspyrone B (**2**) in CDCl_3_

**
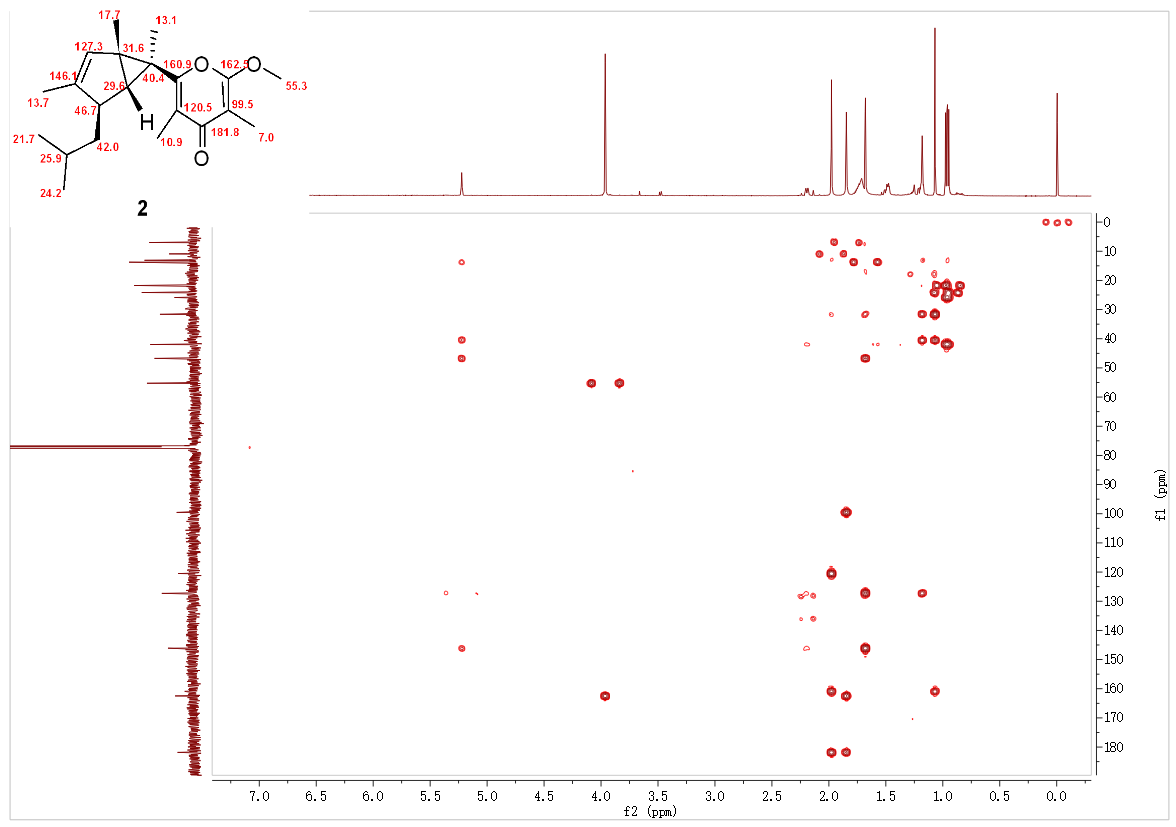
**

**Figure S2e**. ^1^H-^1^H COSY spectrum (600 MHz) of (±)-ocellatuspyrone B (**2**) in CDCl_3_

**
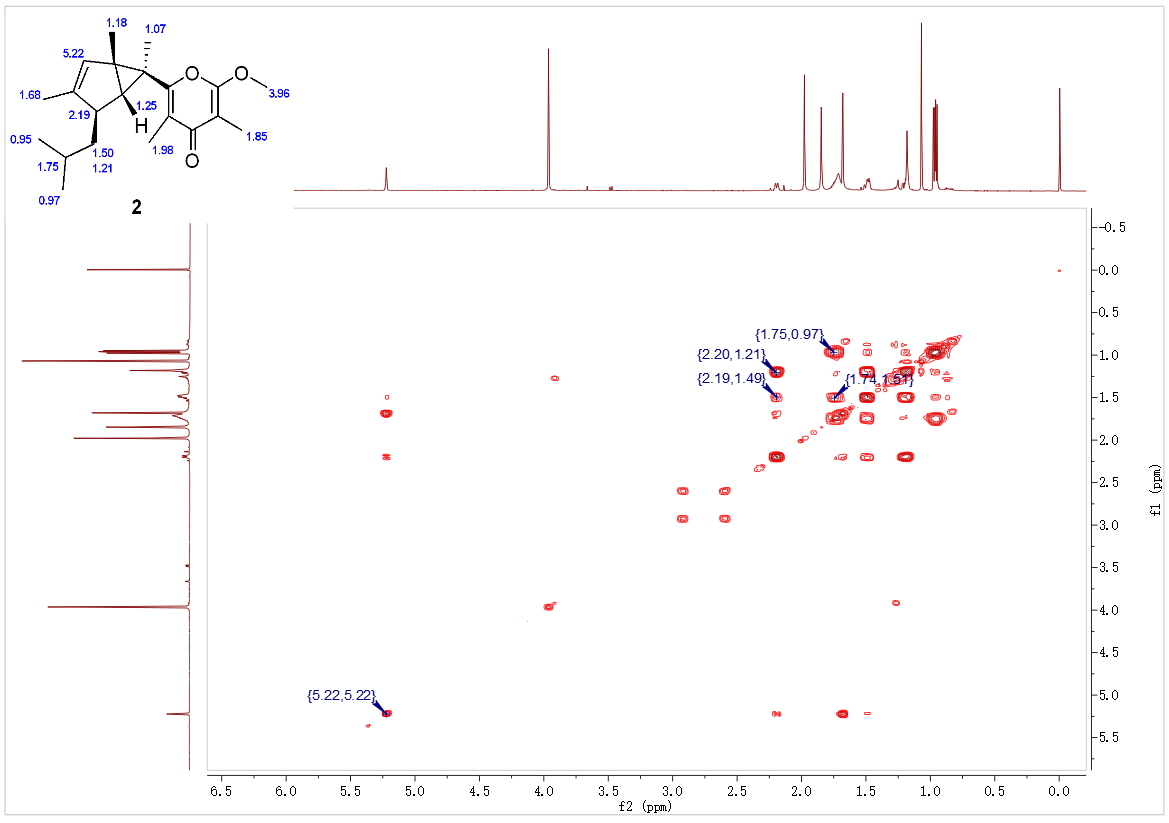
**

**Figure S2f**. NOESY spectrum (600 MHz) of (±)-ocellatuspyrone B (**2**) in CDCl_3_

**
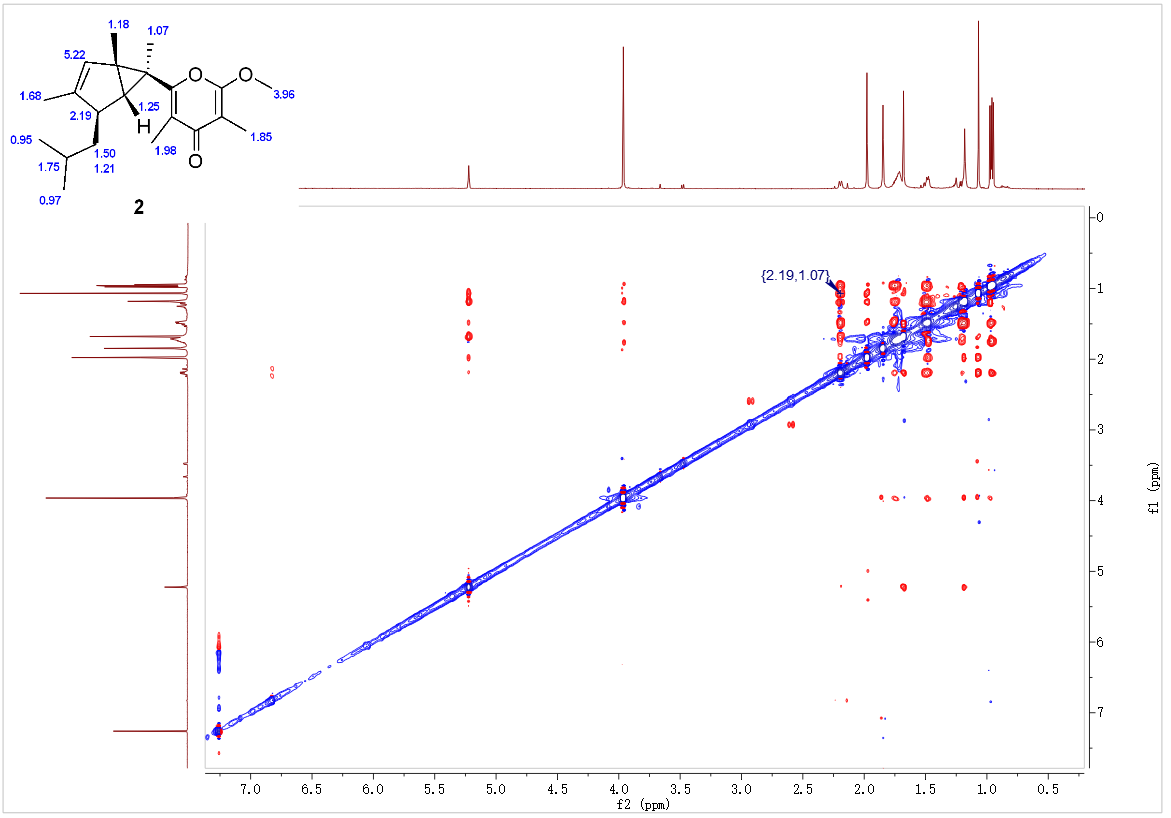
**

**Figure S2g**. HR-ESIMS (positive mode) spectrum of (±)-ocellatuspyrone B (**2**)

**
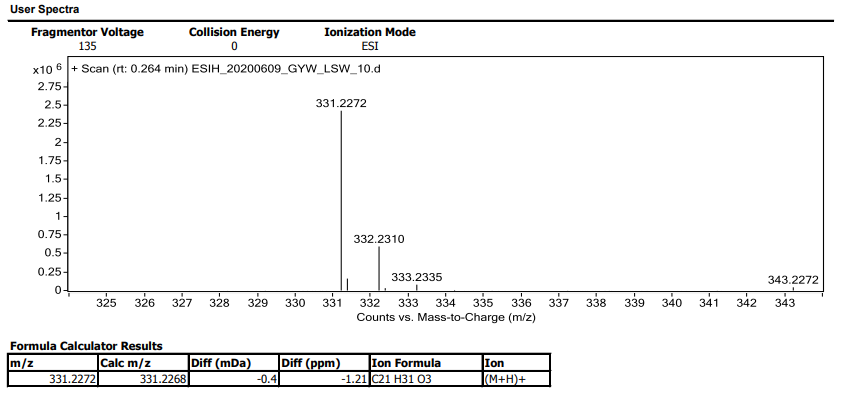
**

**Figure S2h**. IR spectrum of (±)-ocellatuspyrone B (**2**)


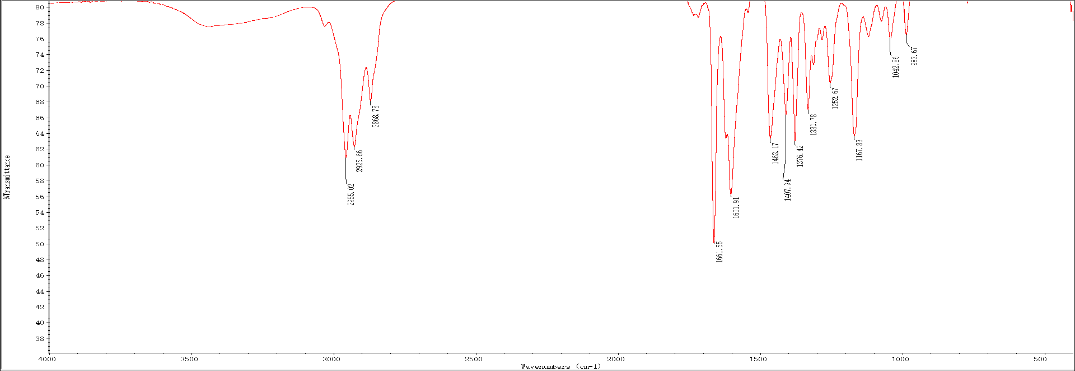


**Figure S2i**. ECD and UV spectra of (±)-ocellatuspyrone B (**2**)


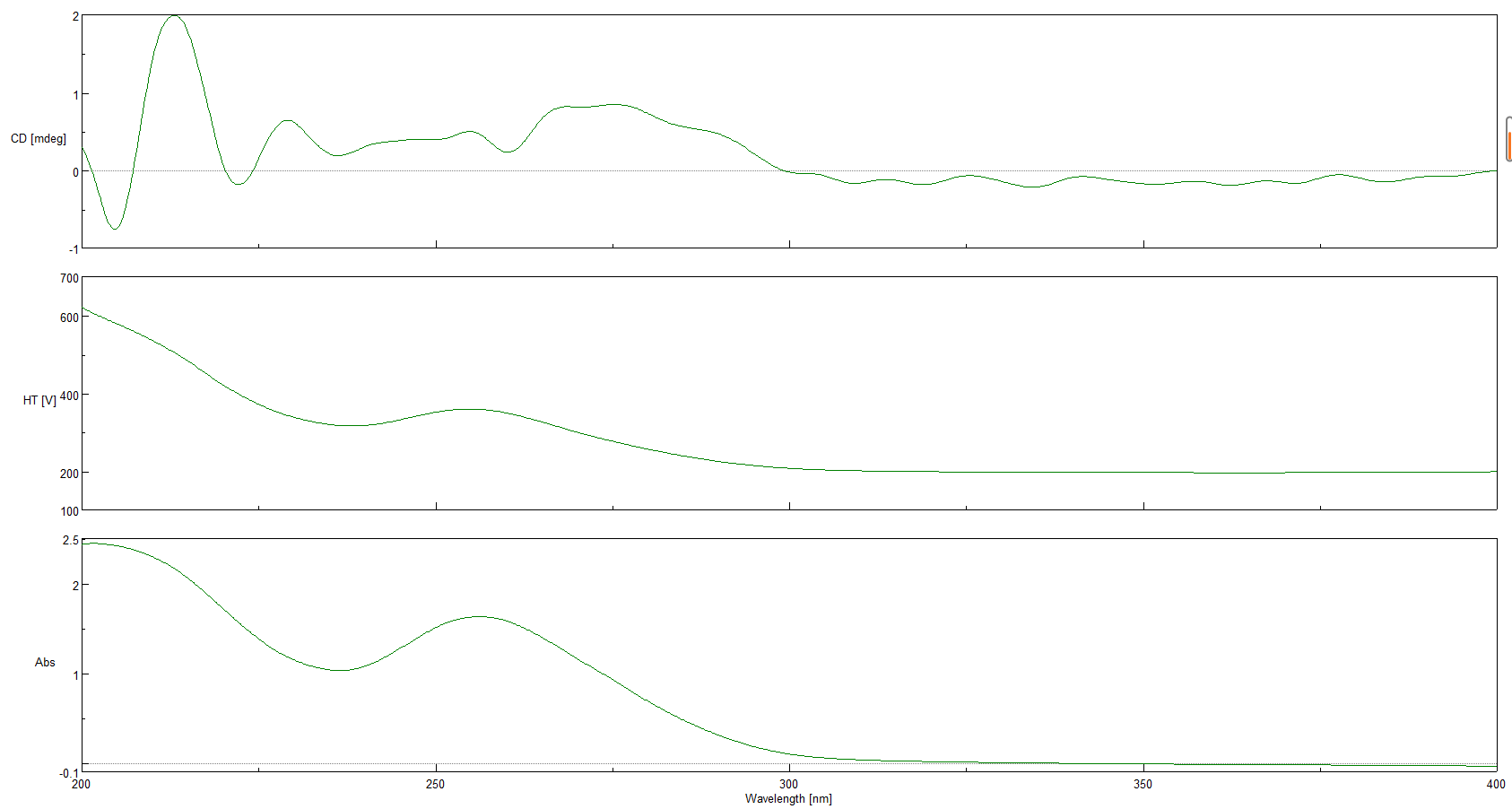


**Figure S2j**. Specific optical rotation of (±)-ocellatuspyrone B (**2**)

**
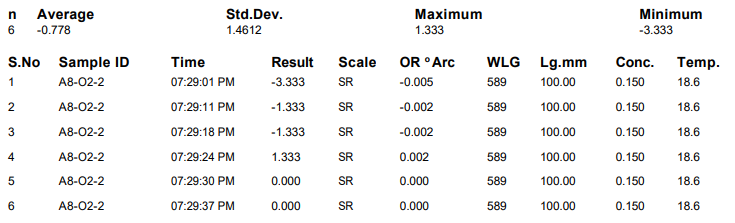
**

**Figure S3a**. ^1^H NMR spectrum (600 MHz) of ocellatuspyrone C (**5**) in CDCl_3_

**
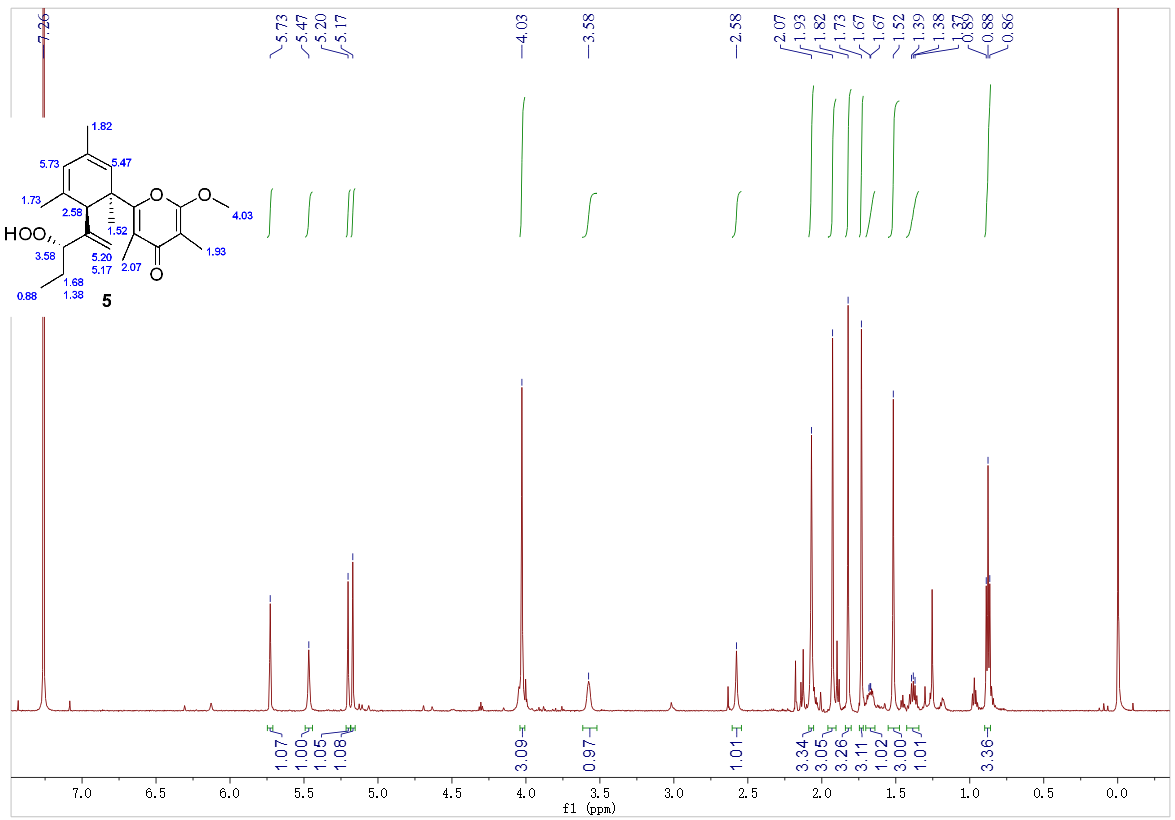
**

**Figure S3b**. ^13^C NMR spectrum (150 MHz) of ocellatuspyrone C (**5**) in CDCl_3_

**
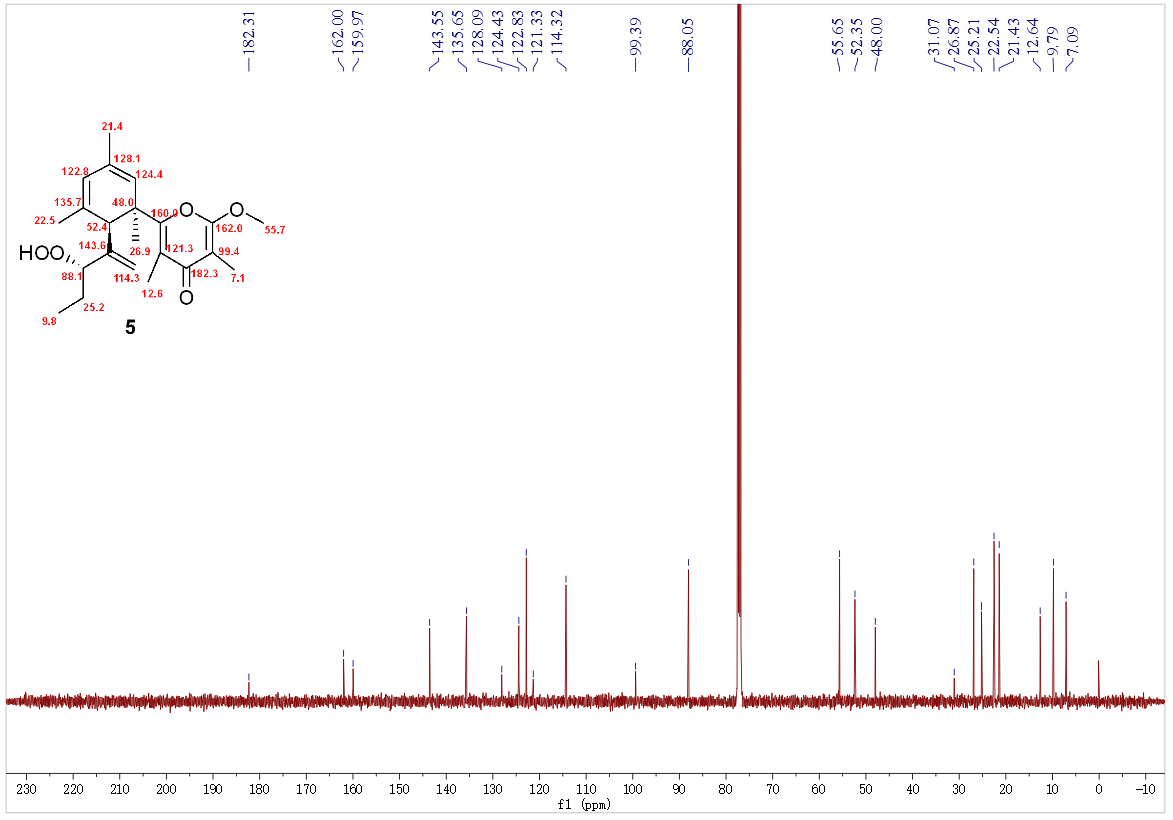
**

**Figure S3c**. HSQC spectrum (600 MHz) of ocellatuspyrone C (**5**) in CDCl_3_

**
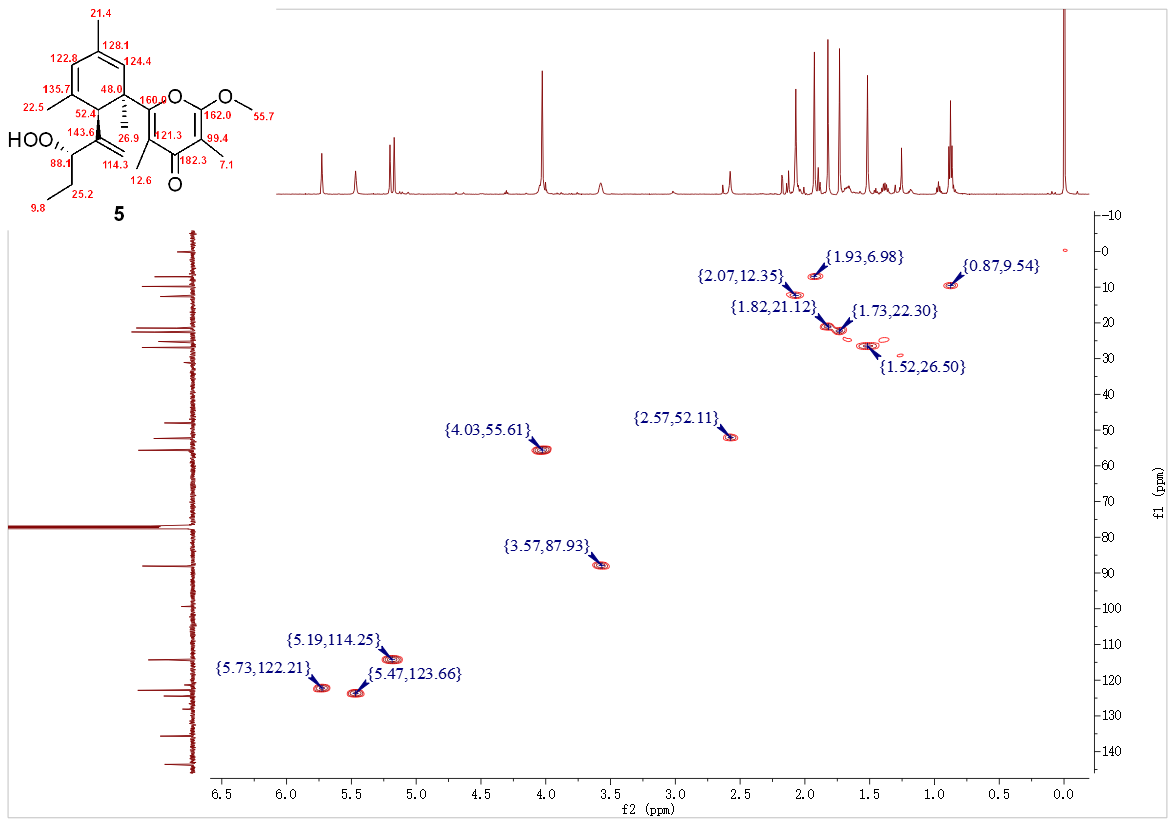
**

**Figure S3d**. HMBC spectrum (600 MHz) of ocellatuspyrone C (**5**) in CDCl_3_

**
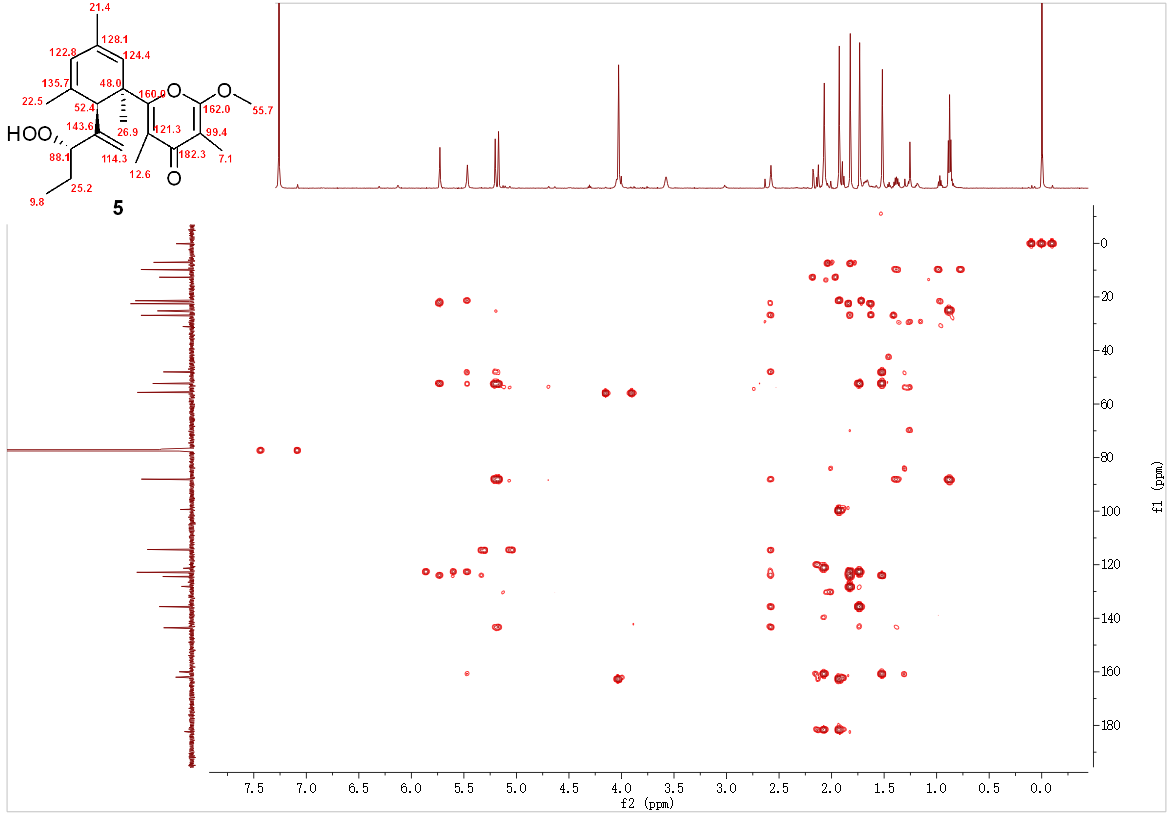
**

**Figure S3e**. ^1^H-^1^H COSY spectrum (600 MHz) of ocellatuspyrone C (**5**) in CDCl_3_

**
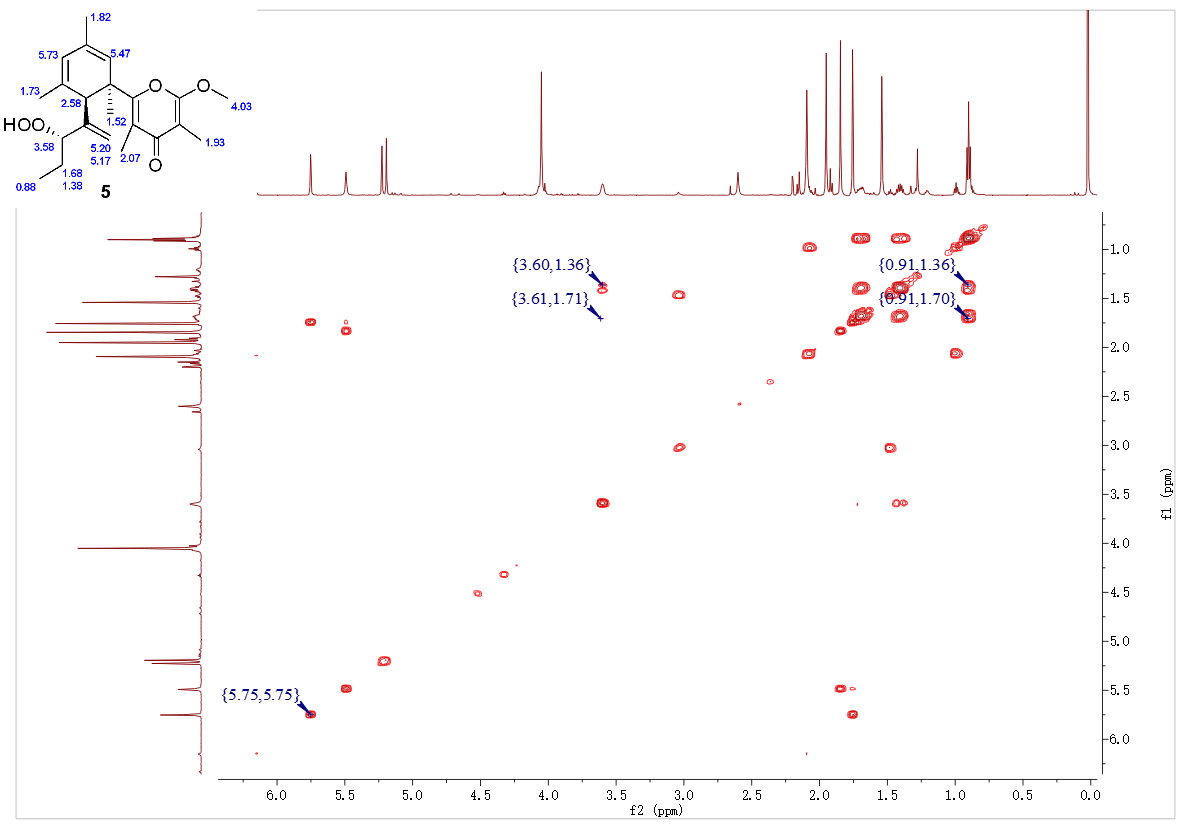
**

**Figure S3f**. NOESY spectrum (600 MHz) of ocellatuspyrone C (**5**) in CDCl_3_

**
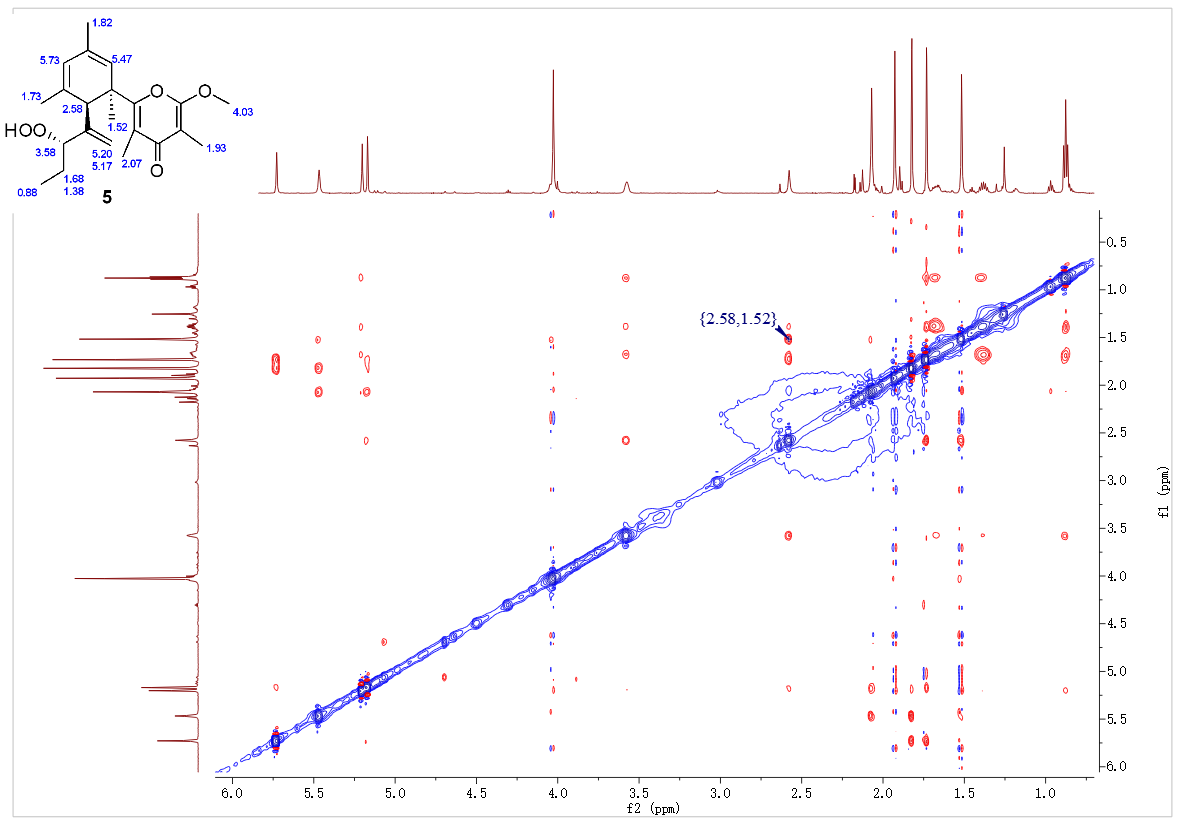
**

**Figure S3g**. HR-ESIMS (positive mode) spectrum of ocellatuspyrone C (**5**)

**
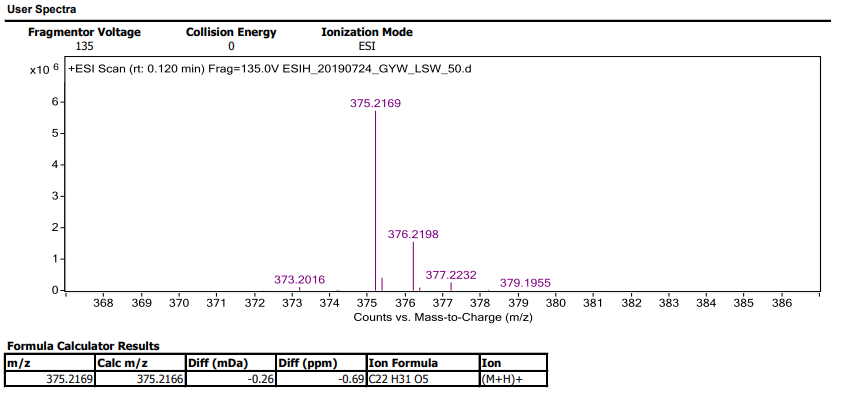
**

**Figure S3h**. IR spectrum of ocellatuspyrone C (**5**)


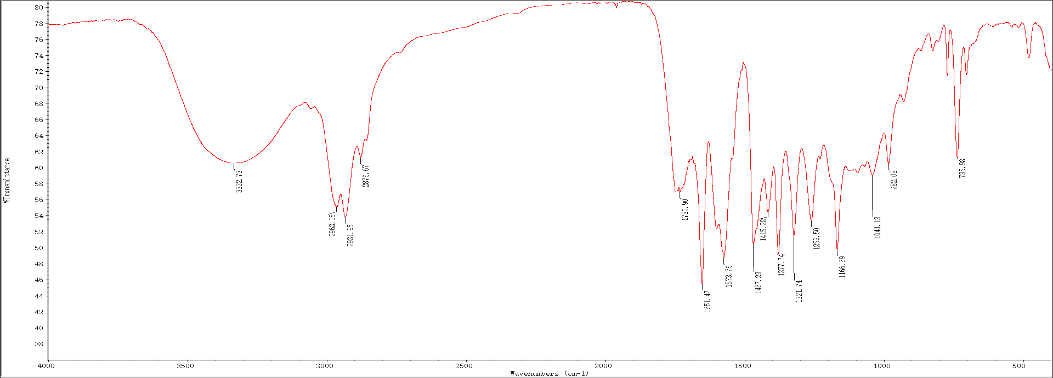


**Figure S3i**. ECD and UV spectra of ocellatuspyrone C (**5**)


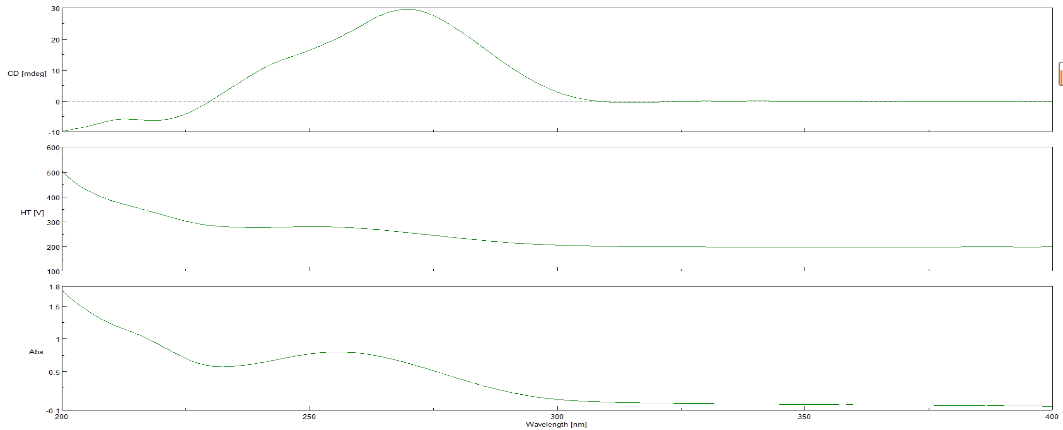


**Figure S3j**. Specific optical rotation of ocellatuspyrone C (**5**)

**
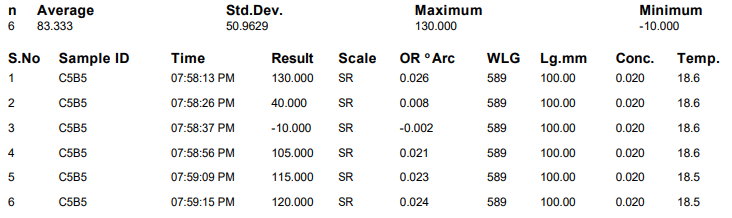
**

**Figure S4a**. ^1^H NMR spectrum (600 MHz) of ocellatuspyrone D (**9**) in CDCl_3_

**
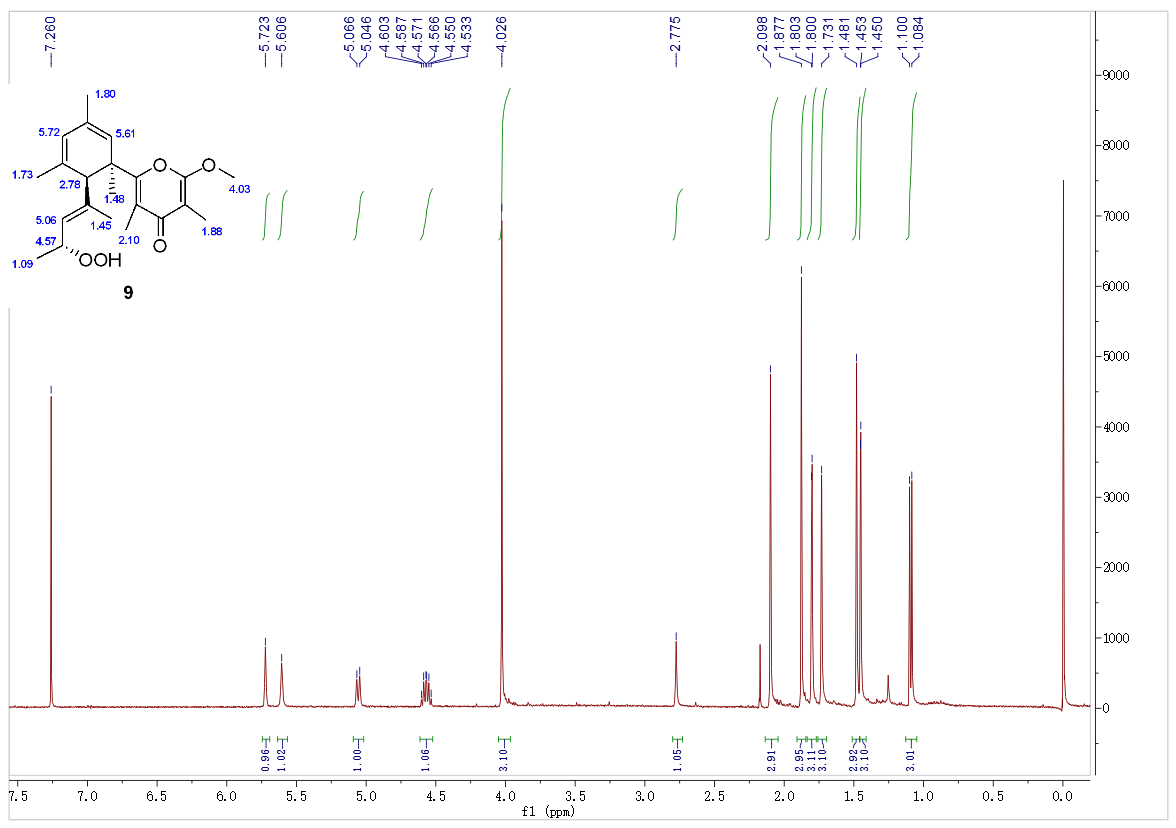
**

**Figure S4b**. ^13^C NMR spectrum (150 MHz) of ocellatuspyrone D (**9**) in CDCl_3_

**
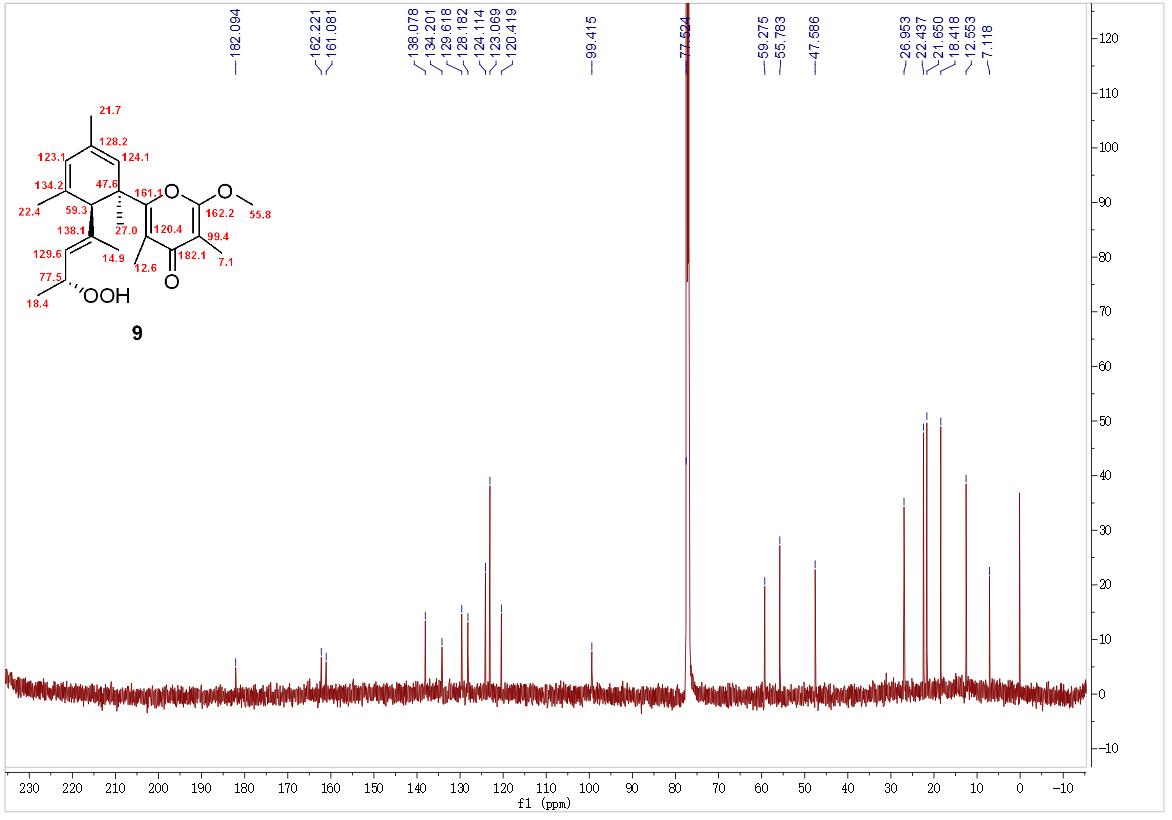
**

**Figure S4c**. HSQC spectrum (600 MHz) of ocellatuspyrone D (**9**) in CDCl_3_

**
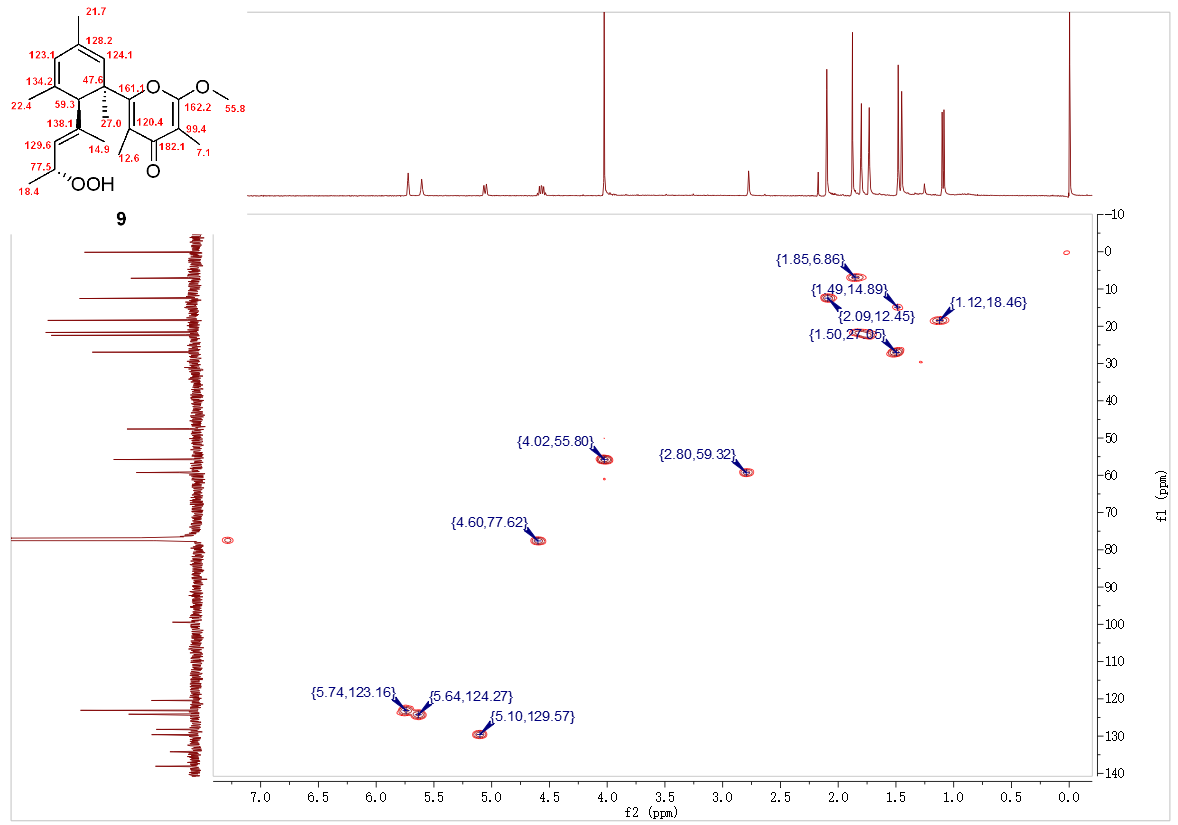
**

**Figure S4d**. HMBC spectrum (600 MHz) of ocellatuspyrone D (**9**) in CDCl_3_

**
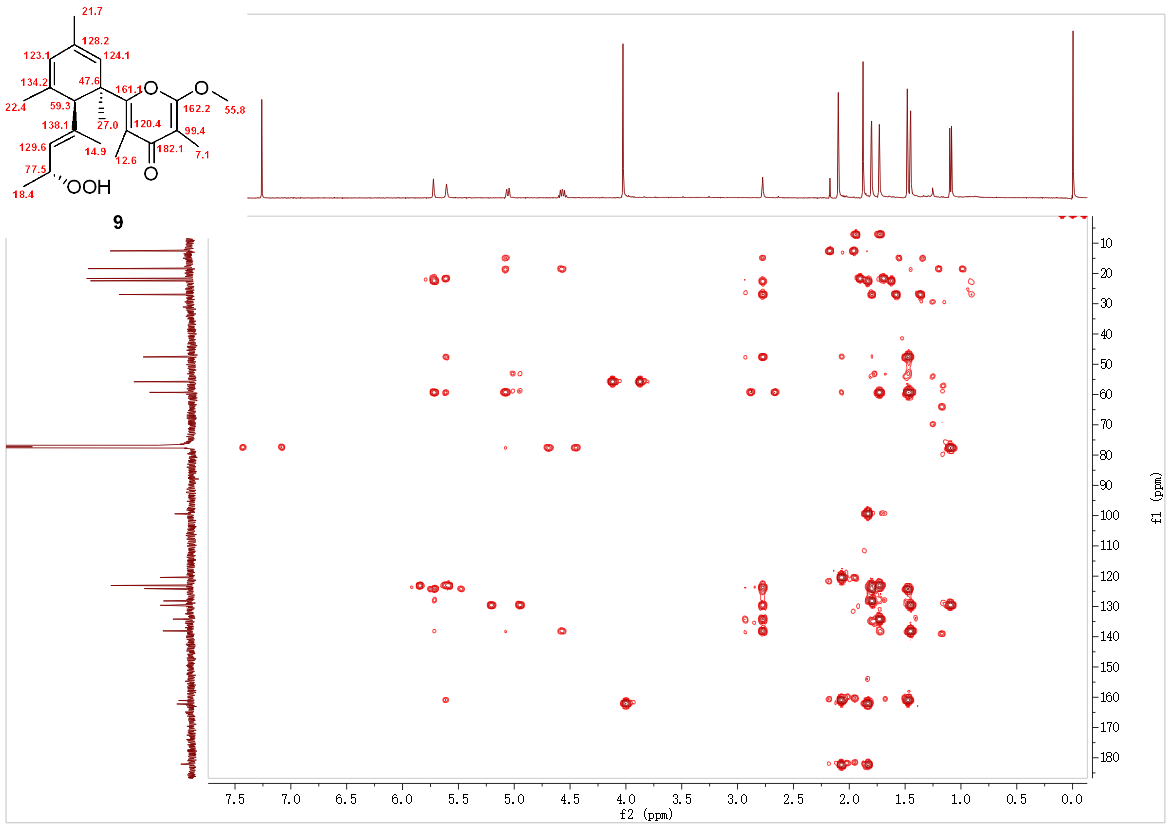
**

**Figure S4e**. ^1^H-^1^H COSY spectrum (600 MHz) of ocellatuspyrone D (**9**) in CDCl_3_

**
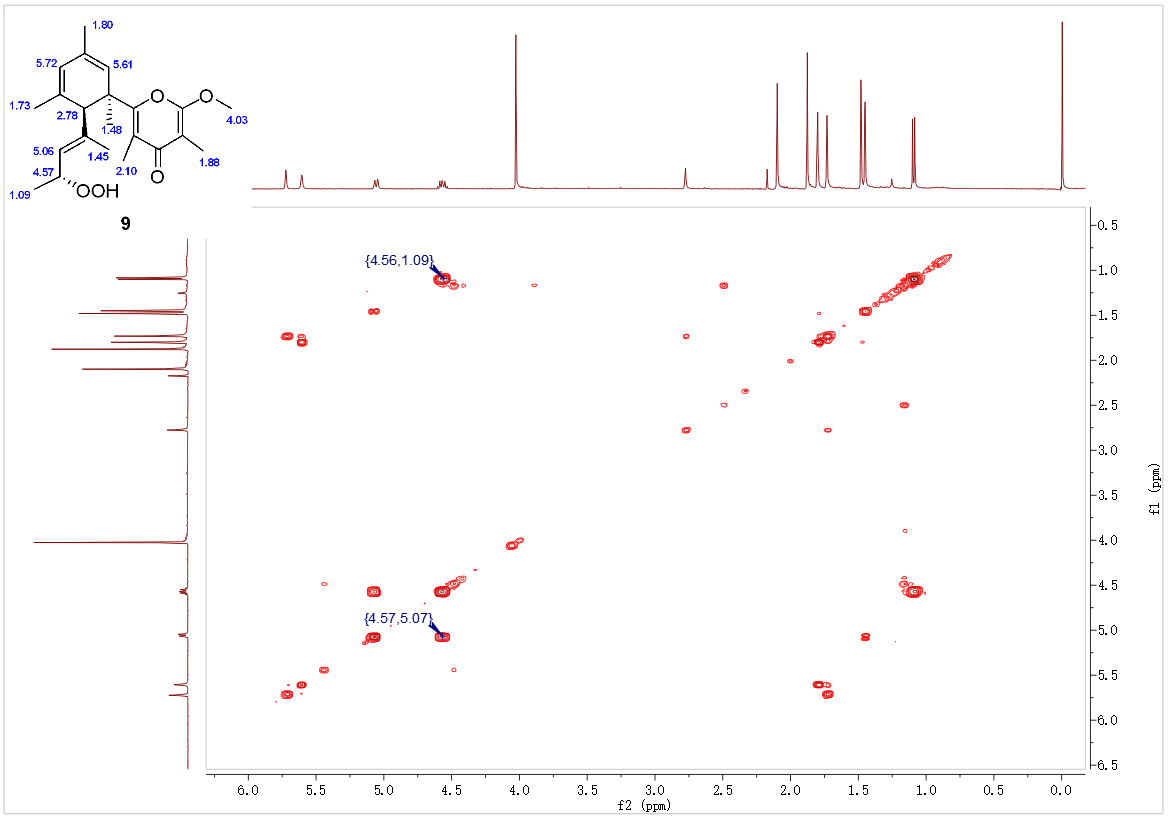
**

**Figure S4f**. NOESY spectrum (600 MHz) of ocellatuspyrone D (**9**) in CDCl_3_

**
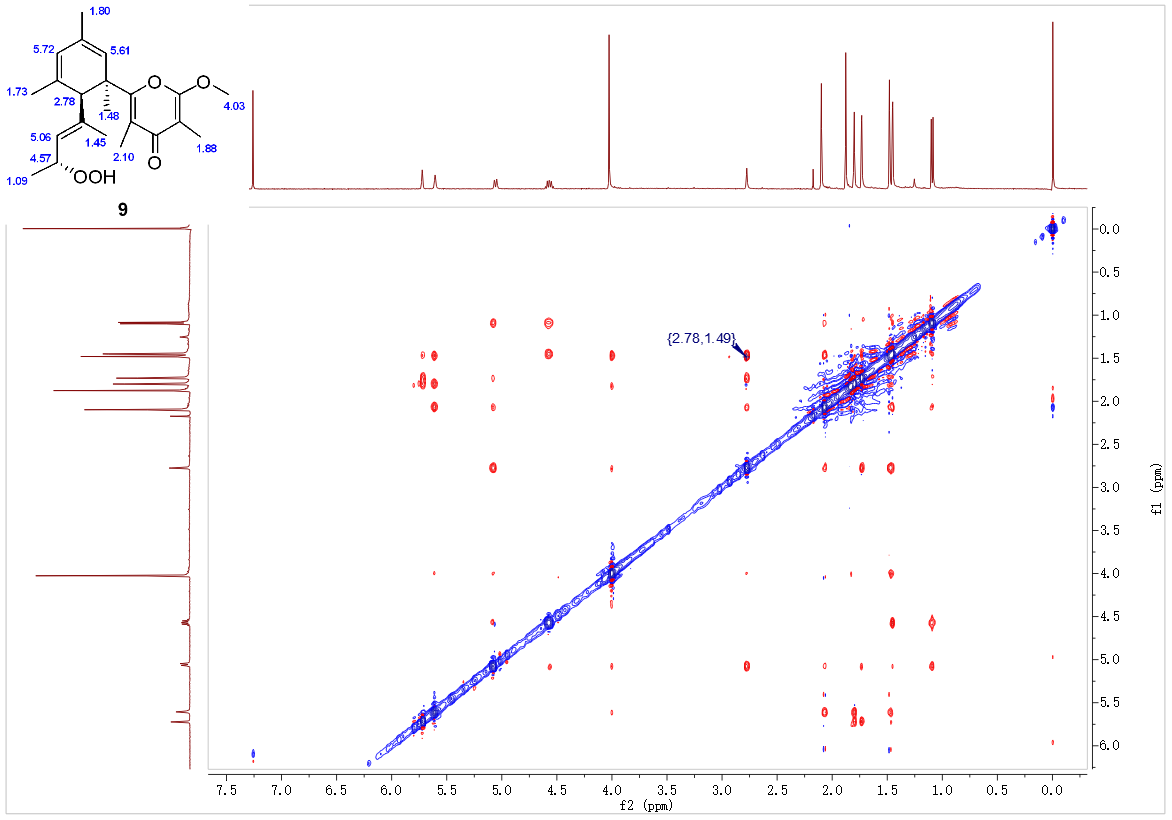
**

**Figure S4g**. HR-ESIMS (positive mode) spectrum of ocellatuspyrone D (**9**)

**
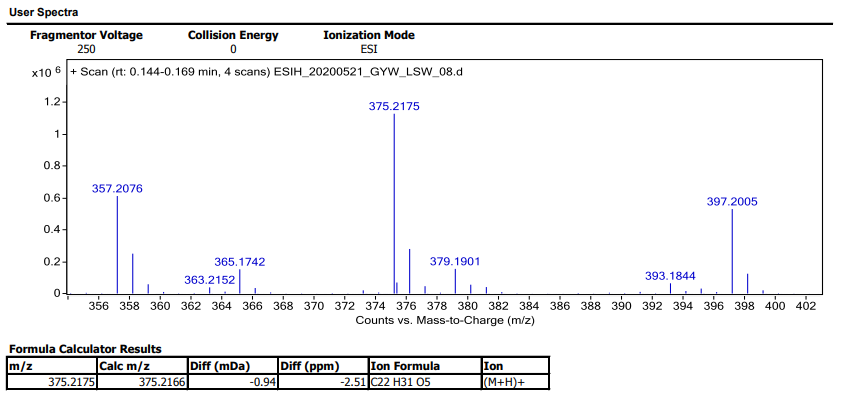
**

**Figure S4h**. IR spectrum of ocellatuspyrone D (**9**)


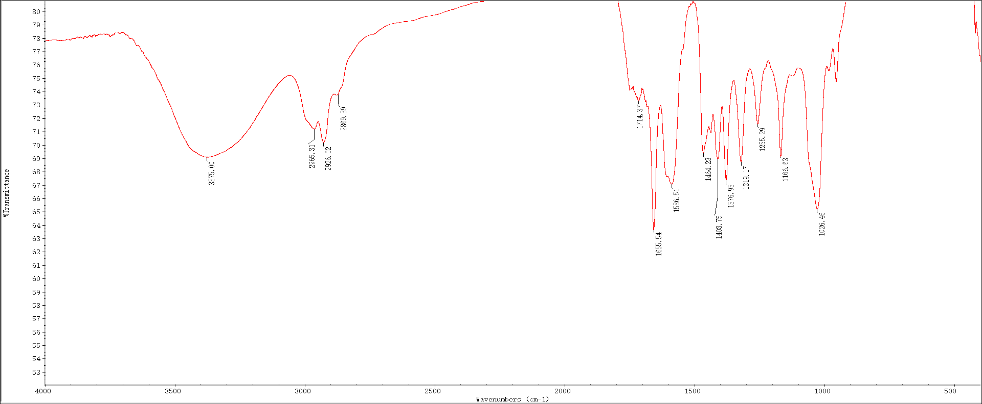


**Figure S4i**. ECD and UV spectra of ocellatuspyrone D (**9**)


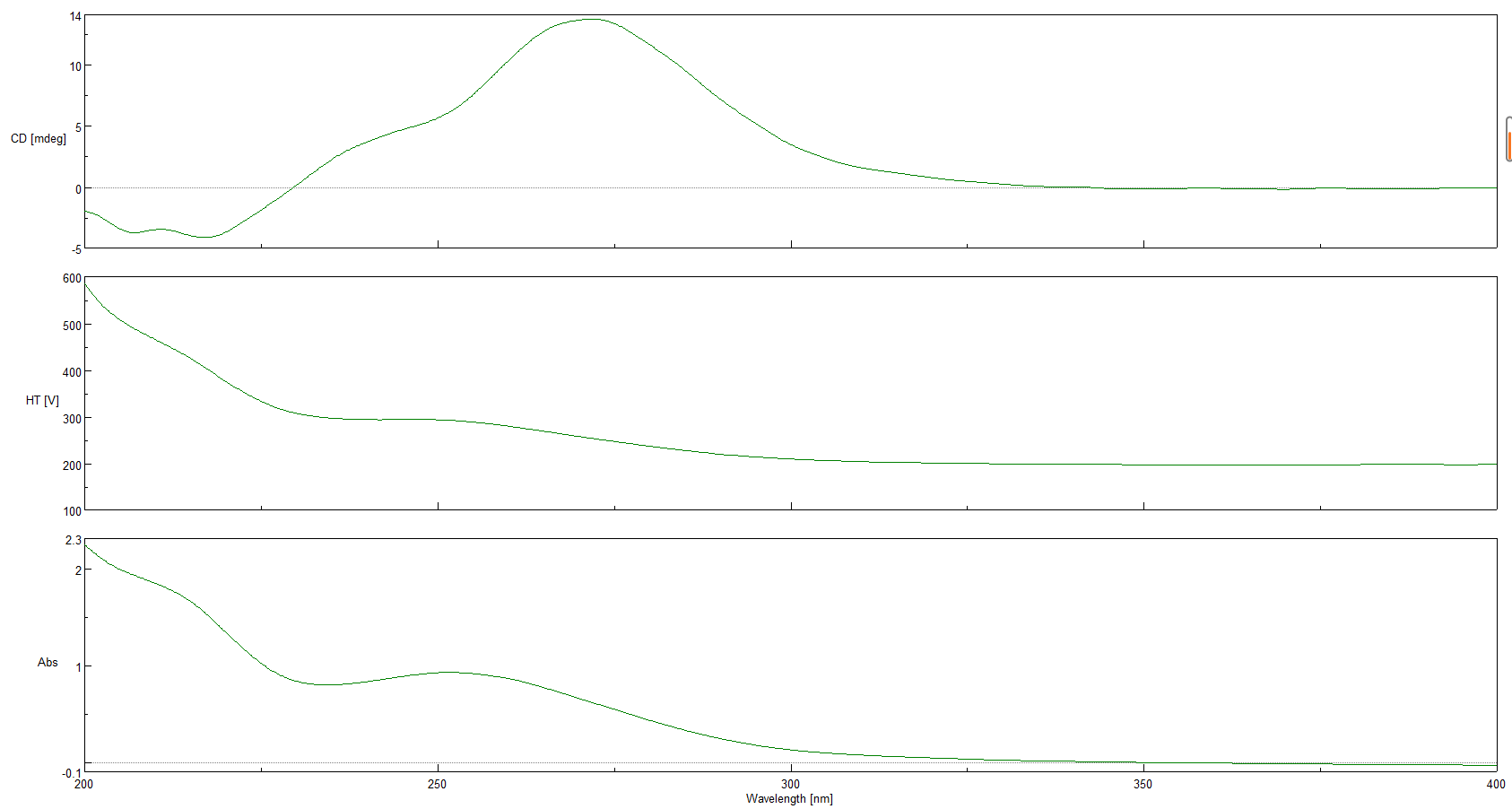


**Figure S4j**. Specific optical rotation of ocellatuspyrone D (**9**)

**
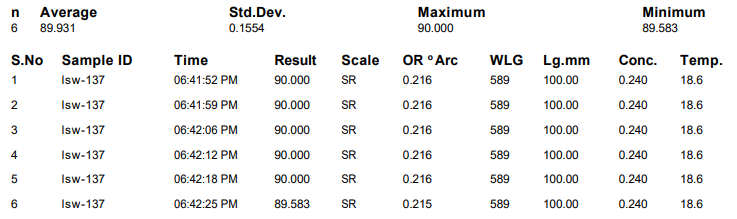
**

**Figure S5a**. ^1^H NMR spectrum (600 MHz) of ocellatuspyrone E (**10**) in CDCl_3_

**
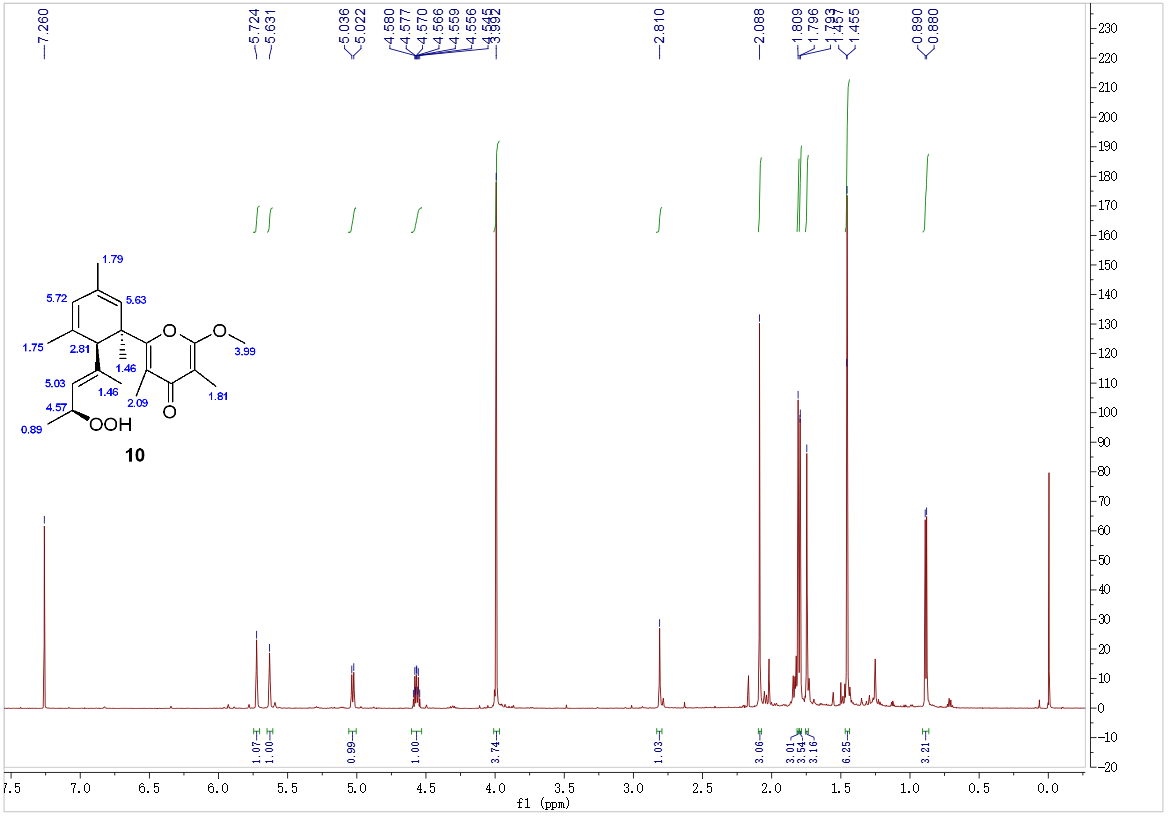
**

**Figure S5b**. ^13^C NMR spectrum (150 MHz) of ocellatuspyrone E (**10**) in CDCl_3_

**
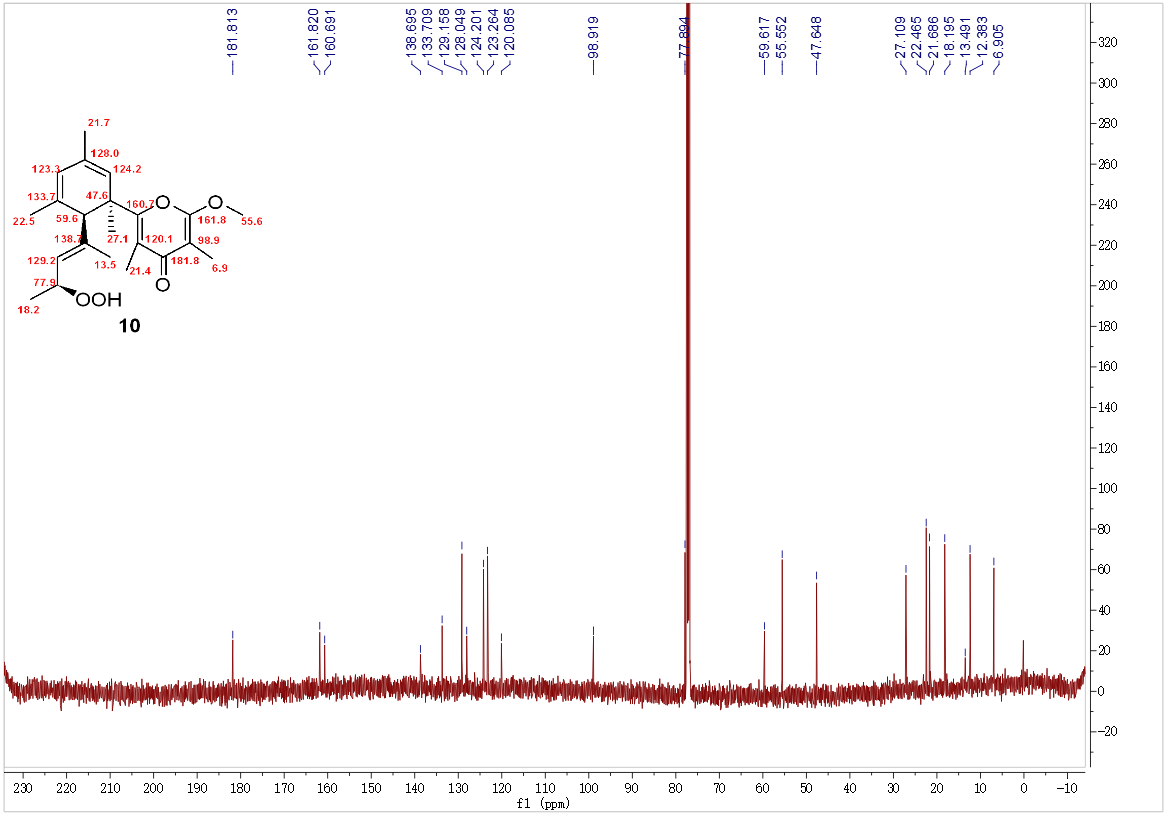
**

**Figure S5c**. HSQC spectrum (600 MHz) of ocellatuspyrone E (**10**) in CDCl_3_

**
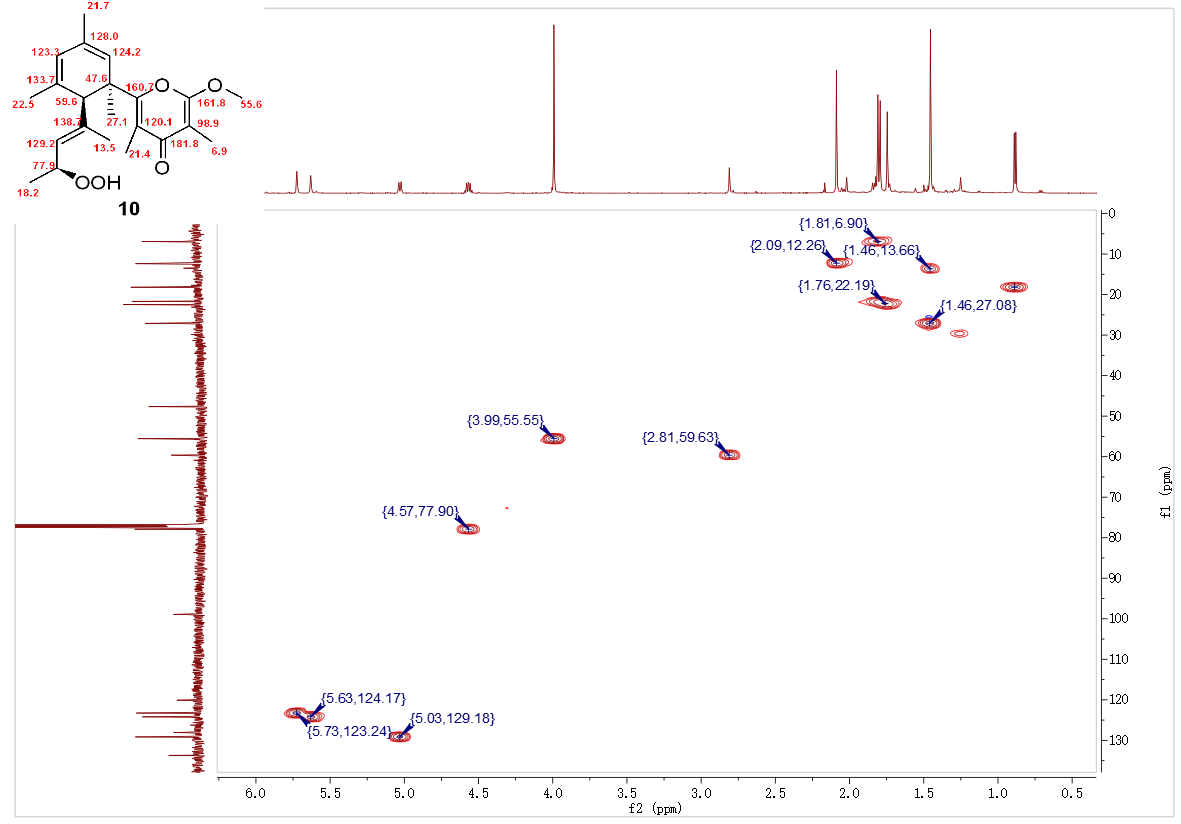
**

**Figure S5d**. HMBC spectrum (600 MHz) of ocellatuspyrone E (**10**) in CDCl_3_

**
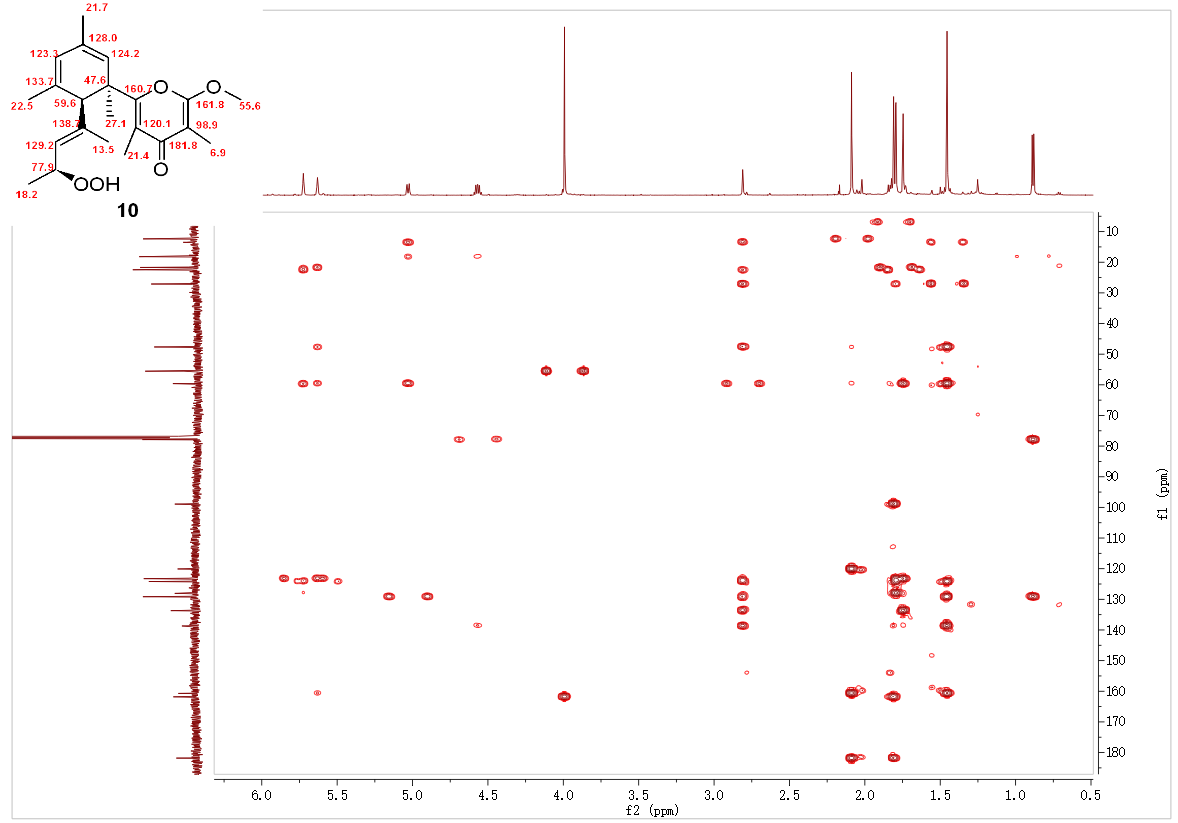
**

**Figure S5e**. ^1^H-^1^H COSY spectrum (600 MHz) of ocellatuspyrone E (**10**) in CDCl_3_

**
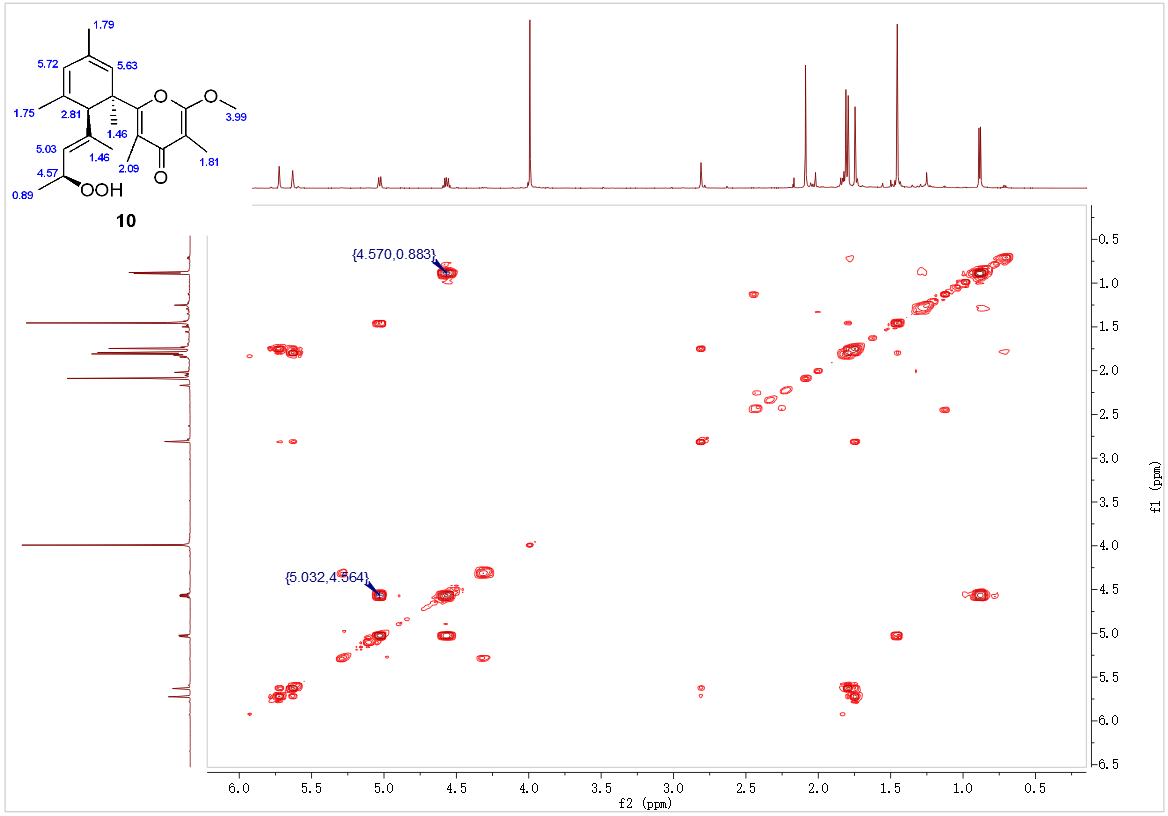
**

**Figure S5f**. NOESY spectrum (600 MHz) of ocellatuspyrone E (**10**) in CDCl_3_

**
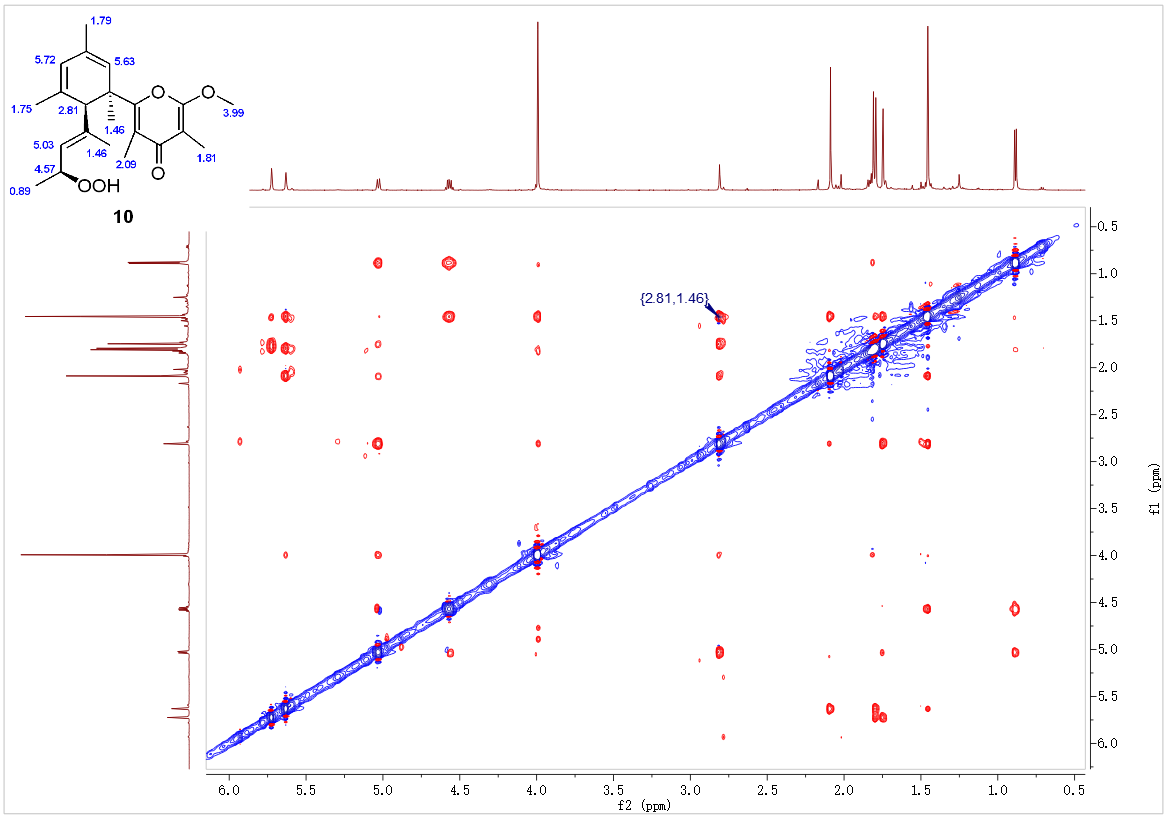
**

**Figure S5g**. HR-ESIMS (positive mode) spectrum of ocellatuspyrone E (**10**)

**
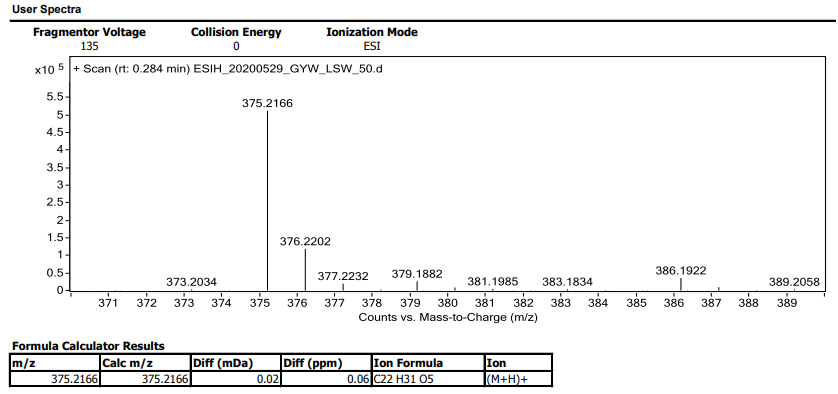
**

**Figure S5h**. IR spectrum of ocellatuspyrone E (**10**)


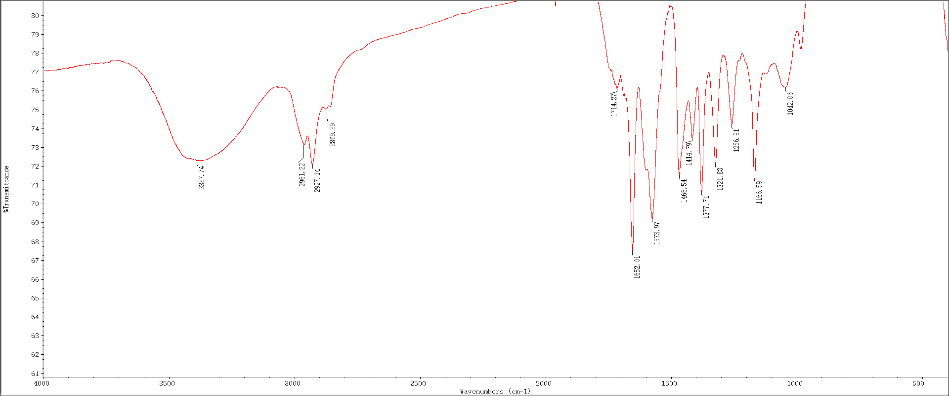


**Figure S5i**. ECD and UV spectra of ocellatuspyrone E (**10**)


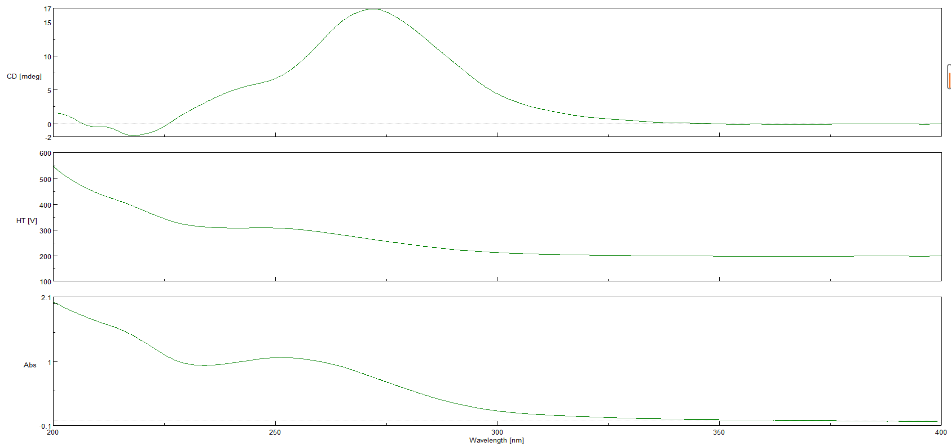


**Figure S5j**. Specific optical rotation of ocellatuspyrone E (**10**)

**
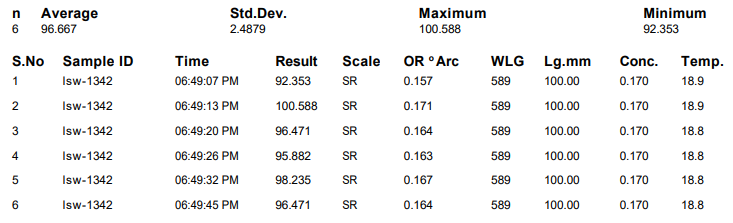
**

**Figure S6a**. ^1^H NMR spectrum (600 MHz) of ocellatuspyrone F (**11**) in CDCl_3_

**
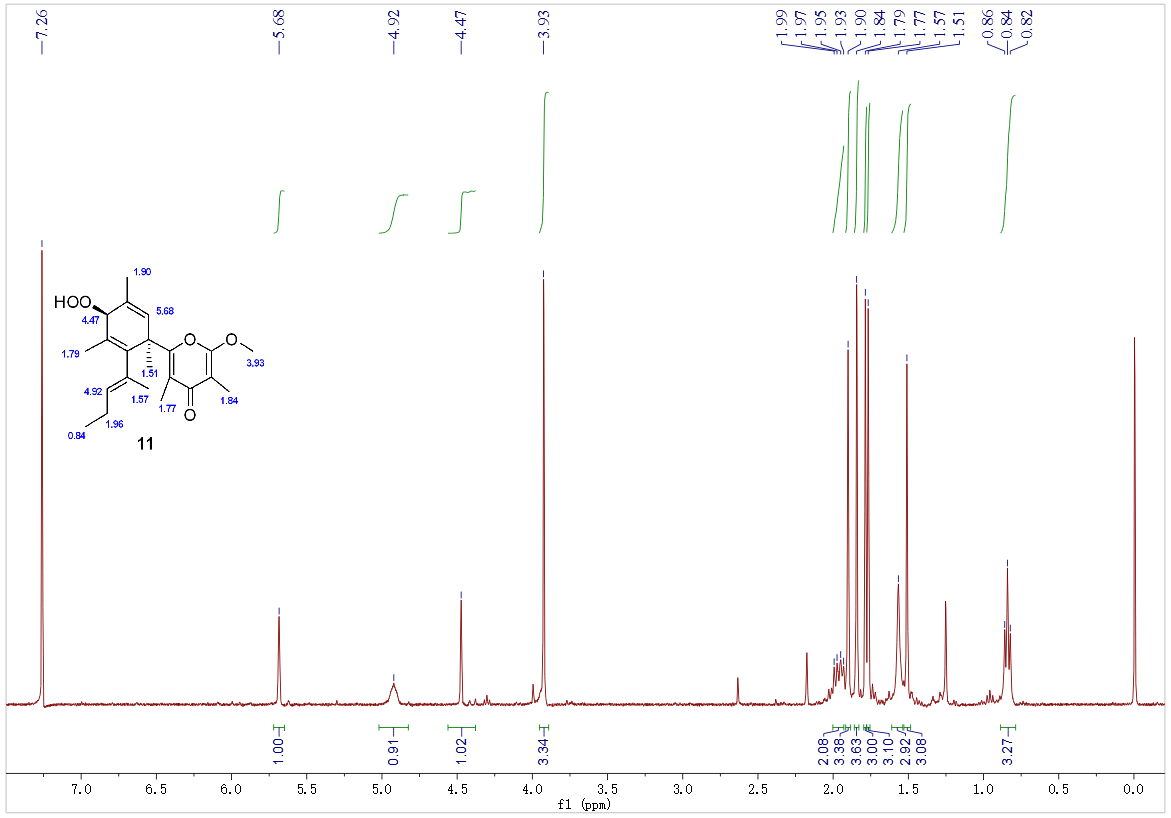
**

**Figure S6b**. ^13^C NMR spectrum (150 MHz) of ocellatuspyrone F (**11**) in CDCl_3_

**
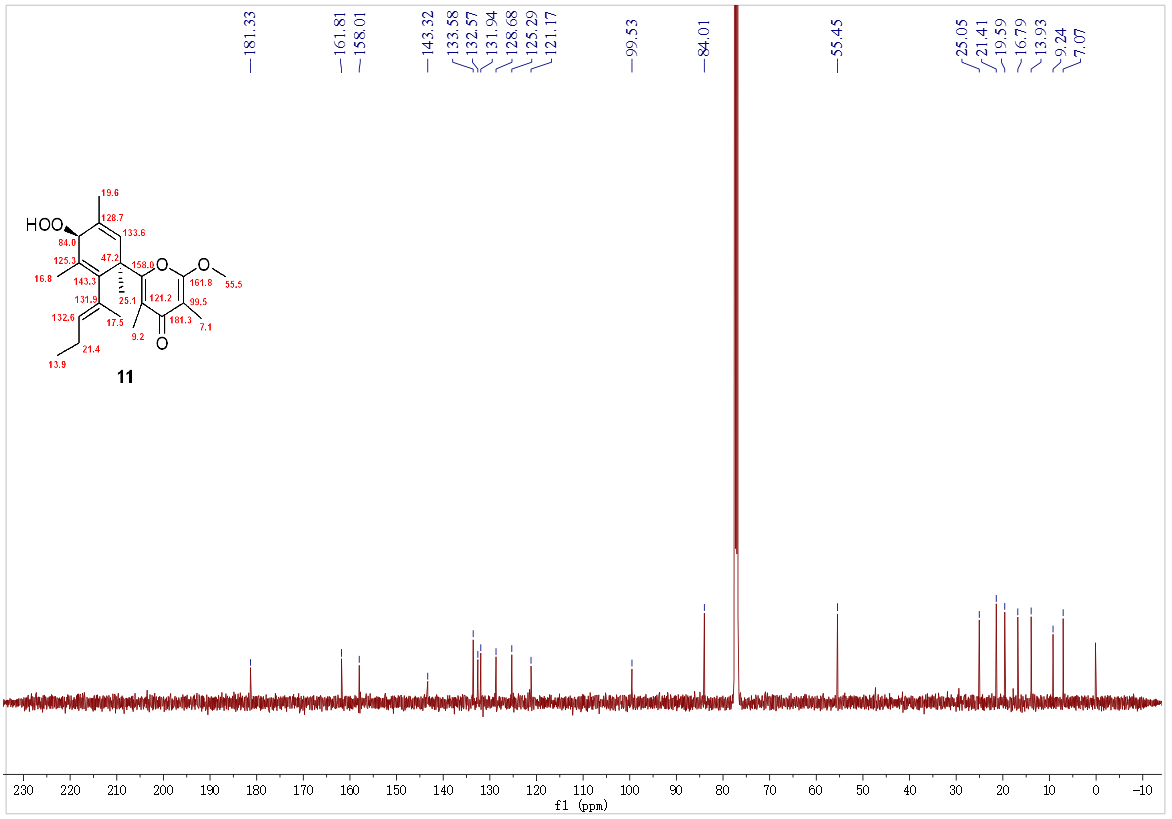
**

**Figure S6c**. HSQC spectrum (600 MHz) of ocellatuspyrone F (**11**) in CDCl_3_

**
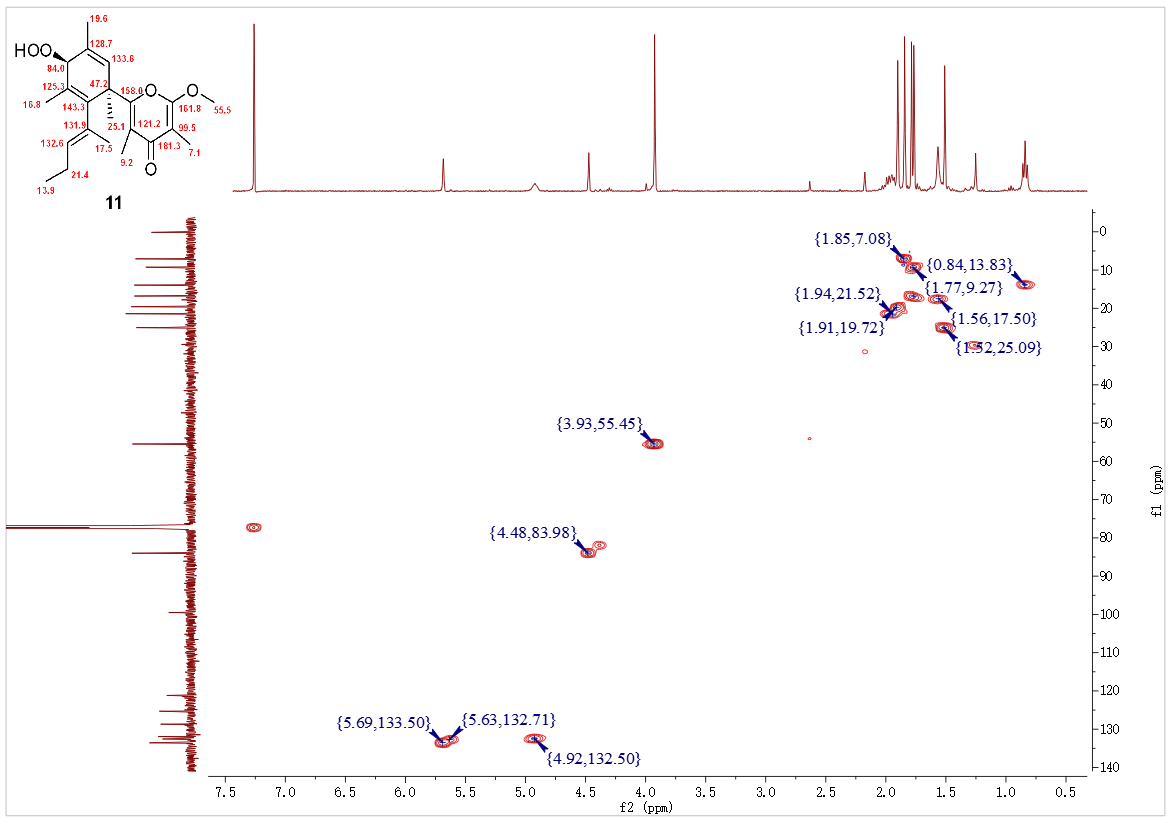
**

**Figure S6d**. HMBC spectrum (600 MHz) of ocellatuspyrone F (**11**) in CDCl_3_

**
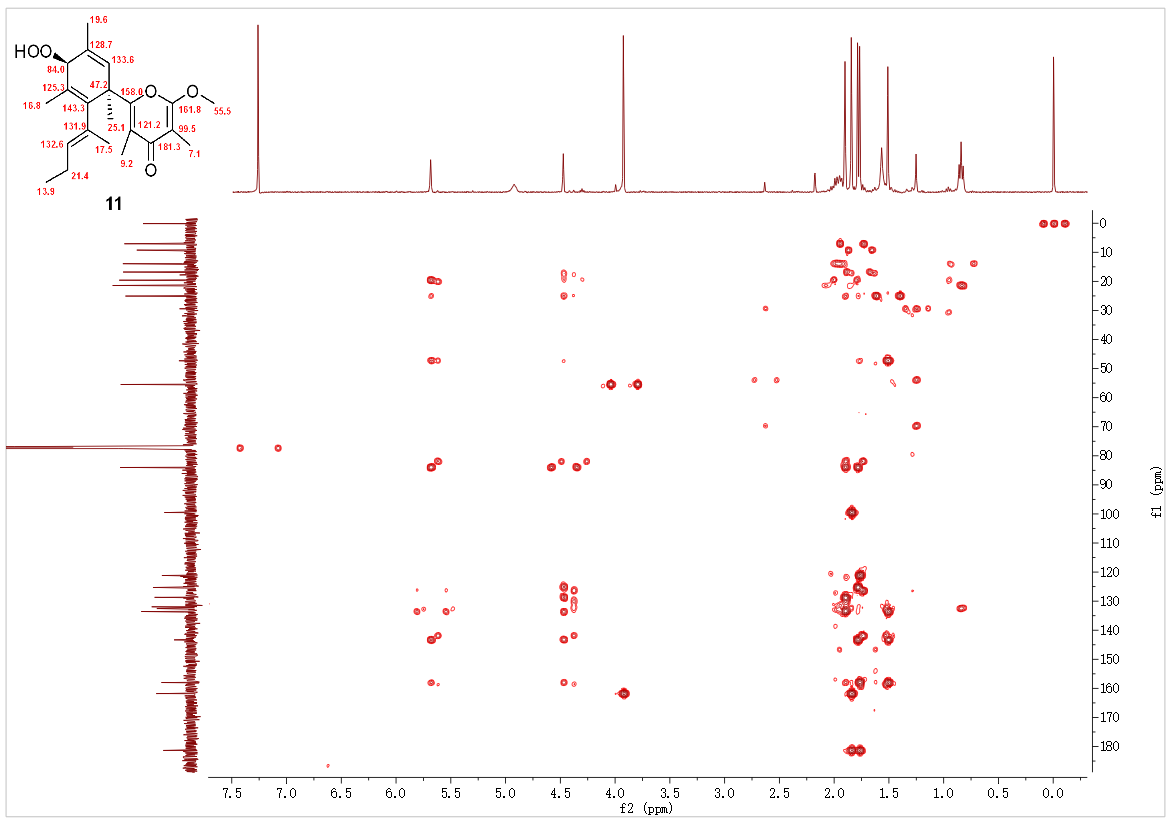
**

**Figure S6e.** ^1^H-^1^H COSY spectrum (600 MHz) of ocellatuspyrone F (**11**) in CDCl_3_

**
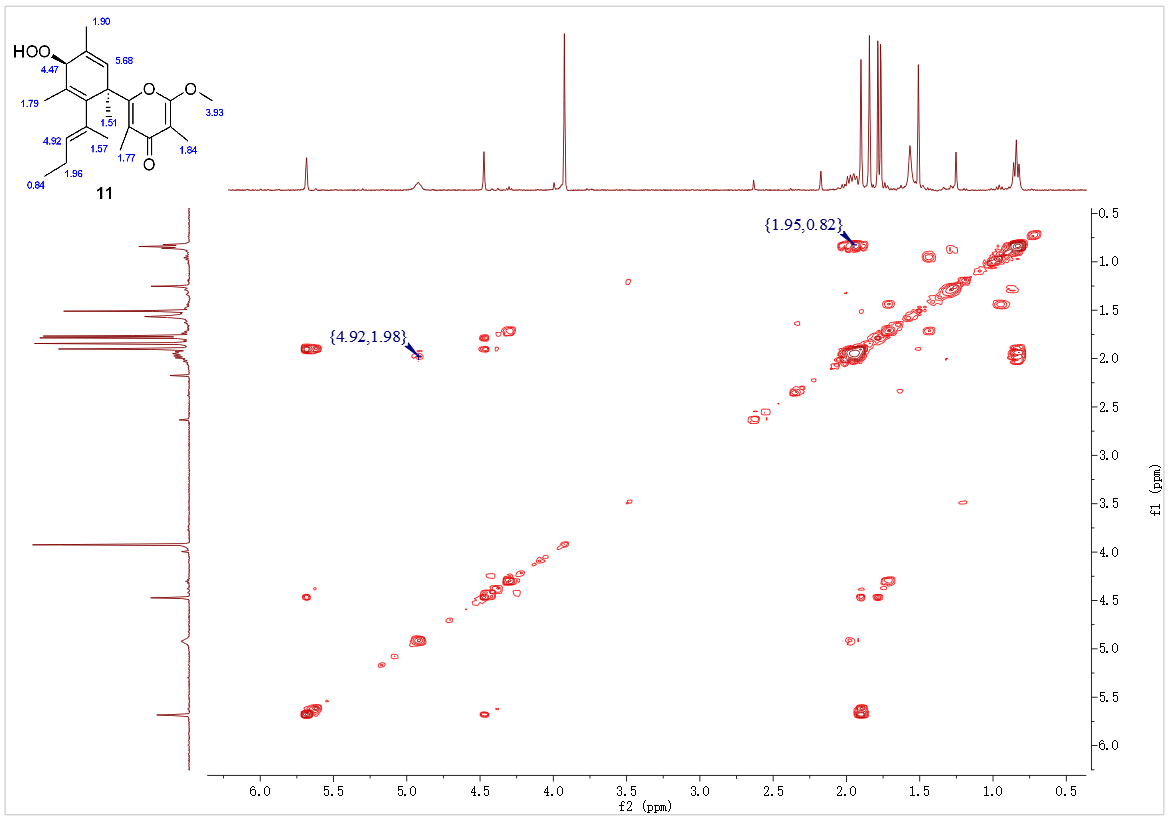
**

**Figure S6f**. NOESY spectrum (600 MHz) of ocellatuspyrone F (**11**) in CDCl_3_

**
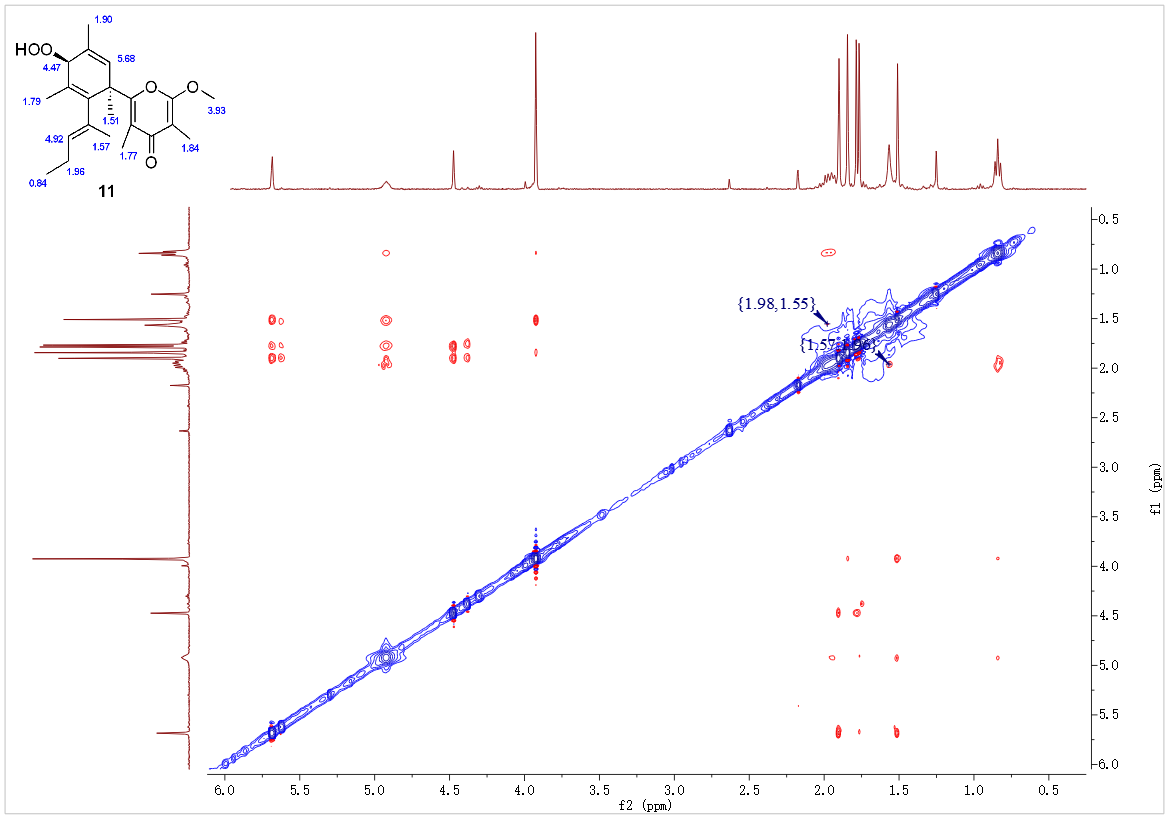
**

**Figure S6g**. HR-ESIMS (positive mode) spectrum of ocellatuspyrone F (**11**)

**
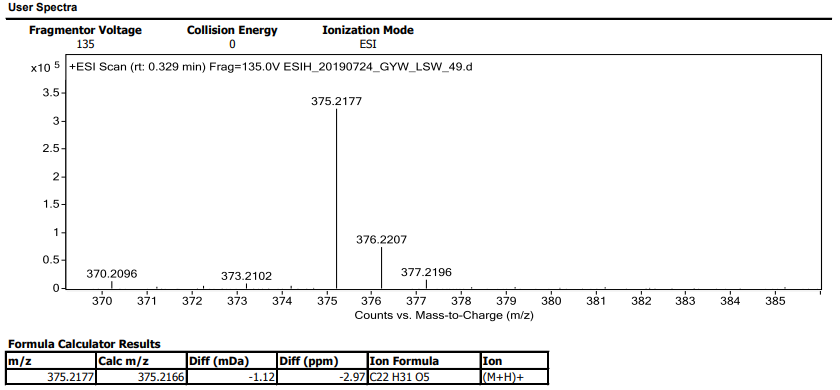
**

**Figure S6h**. IR spectrum of ocellatuspyrone F (**11**)


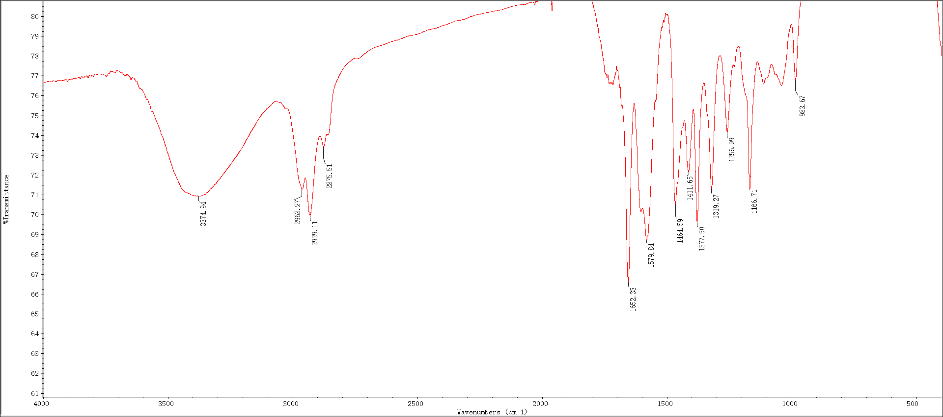


**Figure S6i**. ECD and UV spectra of ocellatuspyrone F (**11**)


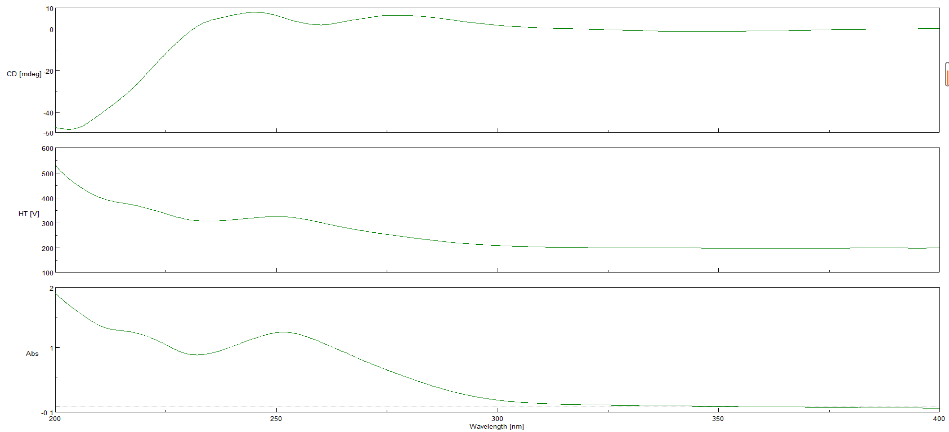


**Figure S6j**. Specific optical rotation of ocellatuspyrone F (**11**)

**
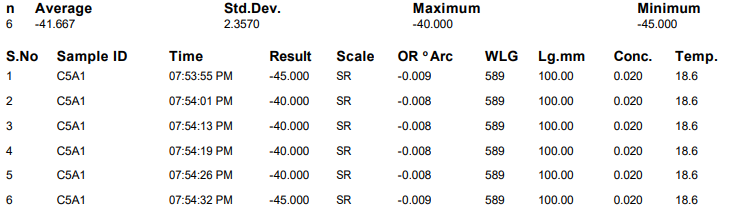
**

**Figure S7a**. ^1^H NMR spectrum (600 MHz) of ocellatuspyrone G (**12**) in CDCl_3_

**
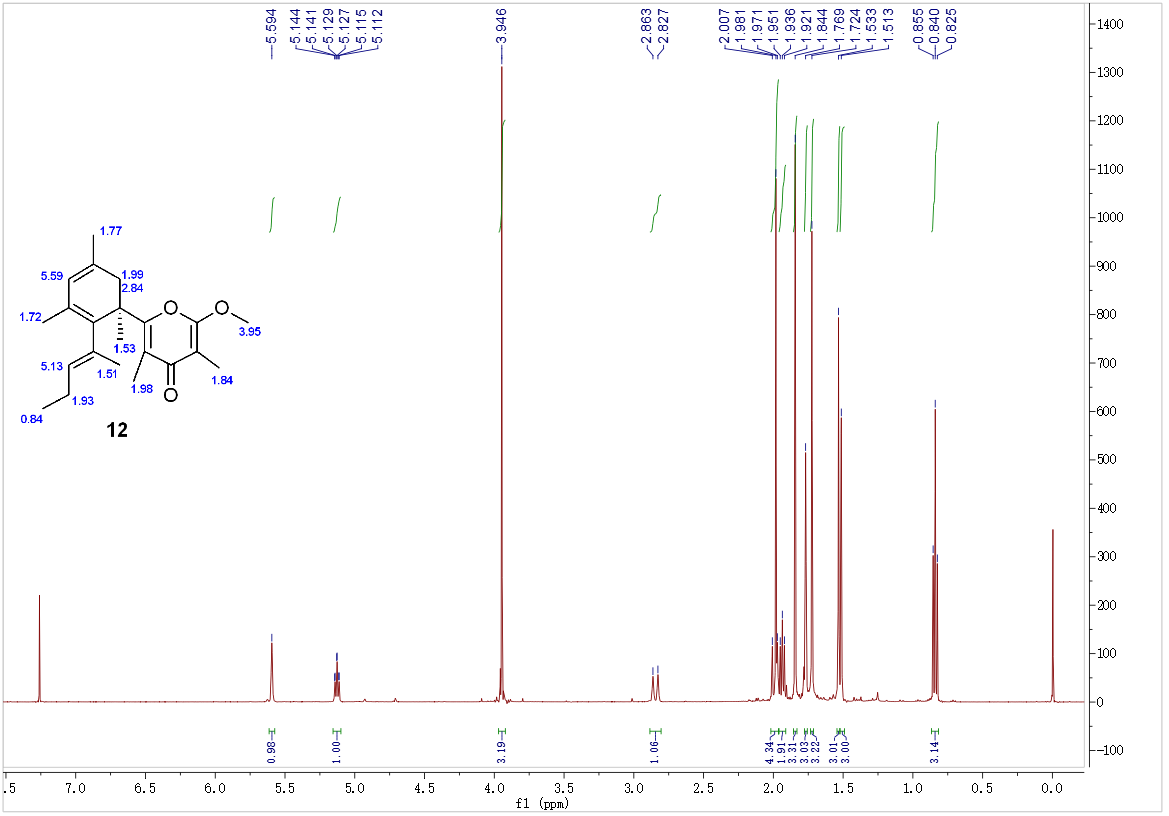
**

**Figure S7b**. ^13^C NMR spectrum (150 MHz) of ocellatuspyrone G (**12**) in CDCl_3_

**
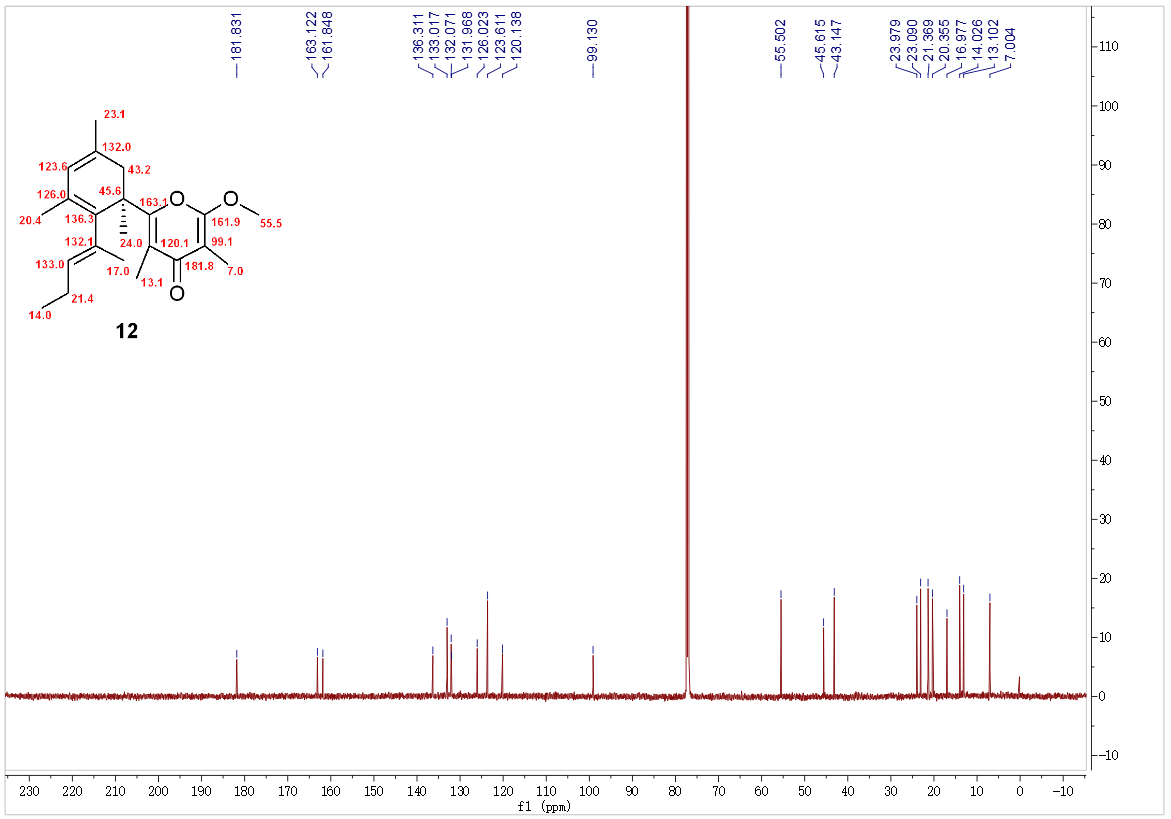
**

**Figure S7c**. HSQC spectrum (600 MHz) of ocellatuspyrone G (**12**) in CDCl_3_

**
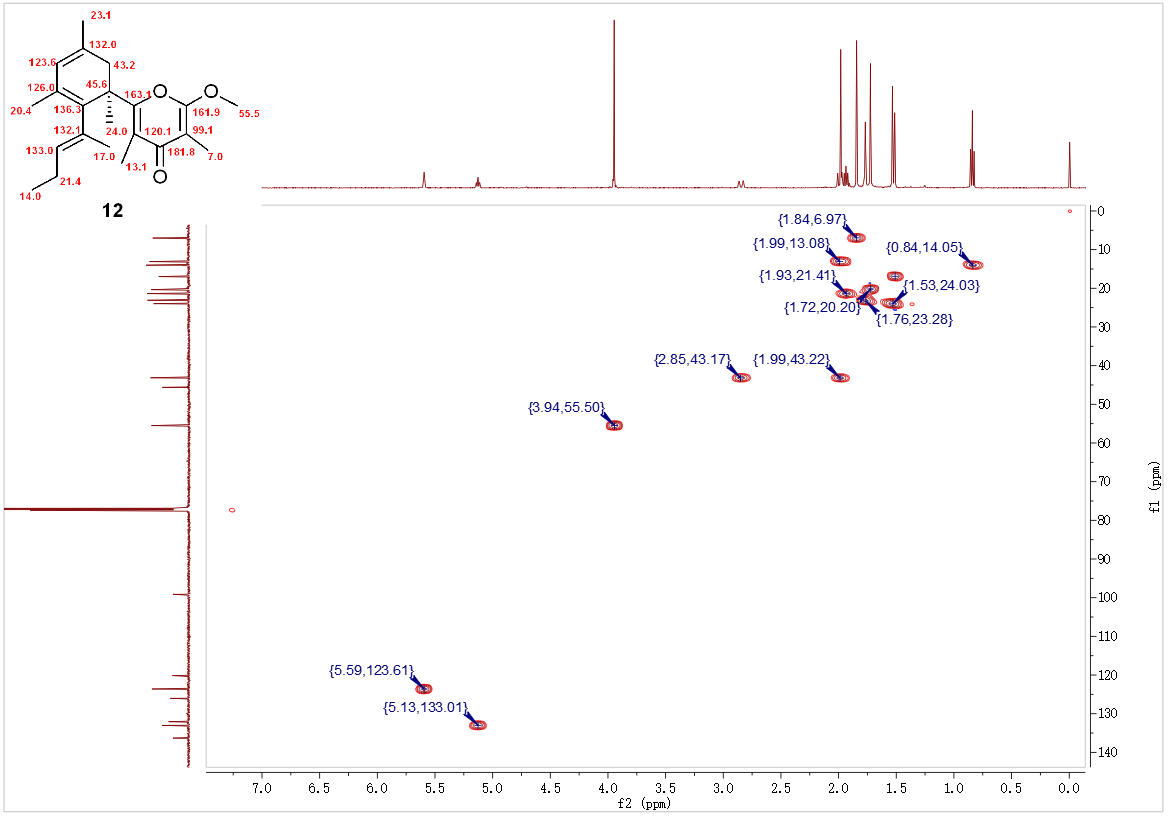
**

**Figure S7d.** HMBC spectrum (600 MHz) of ocellatuspyrone G (**12**) in CDCl_3_

**
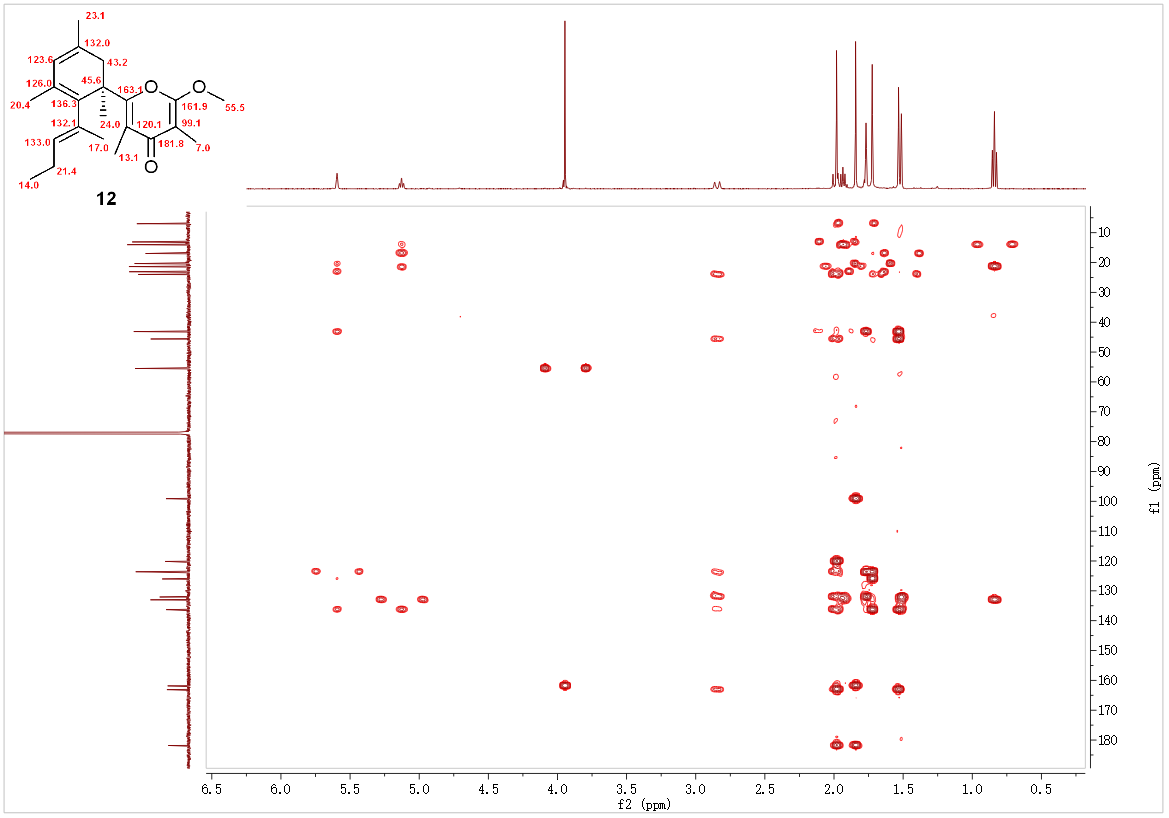
**

**Figure S7e**. ^1^H-^1^H COSY spectrum (600 MHz) of ocellatuspyrone G (**12**) in CDCl_3_

**
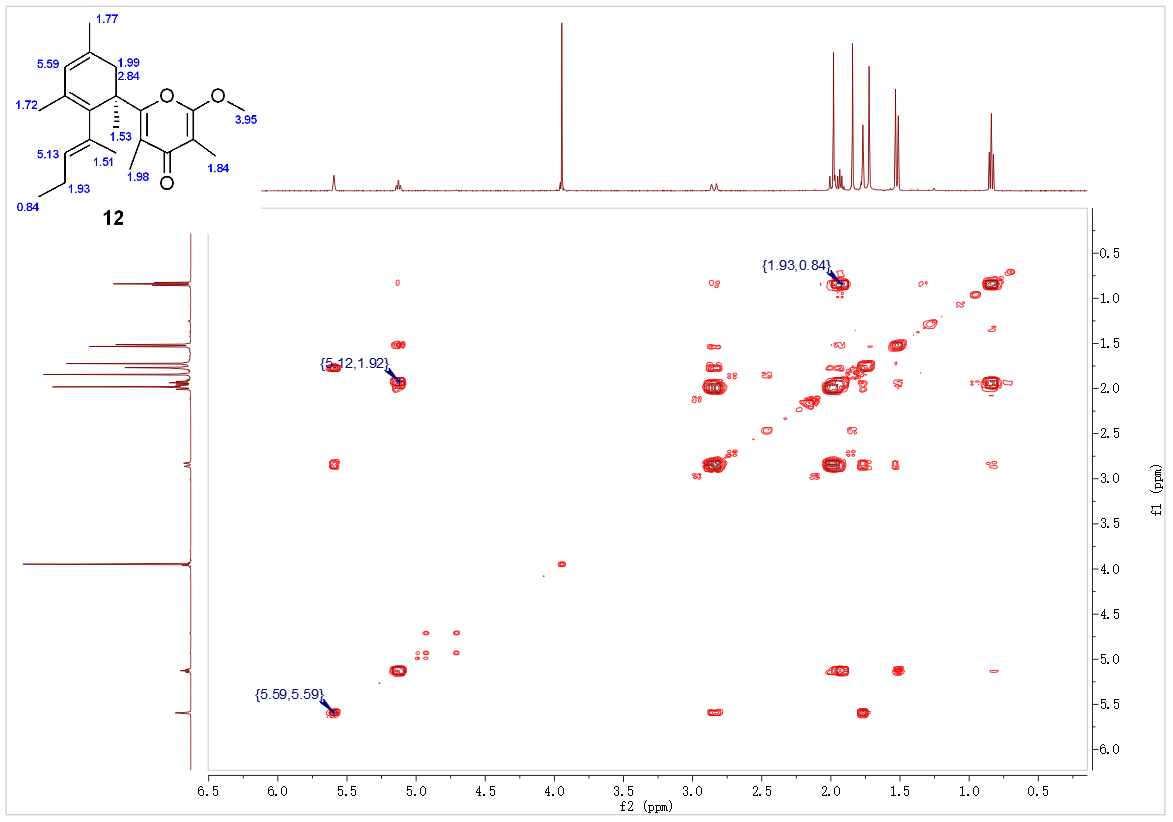
**

**Figure S7f**. NOESY spectrum (600 MHz) of ocellatuspyrone G (**12**) in CDCl_3_

**
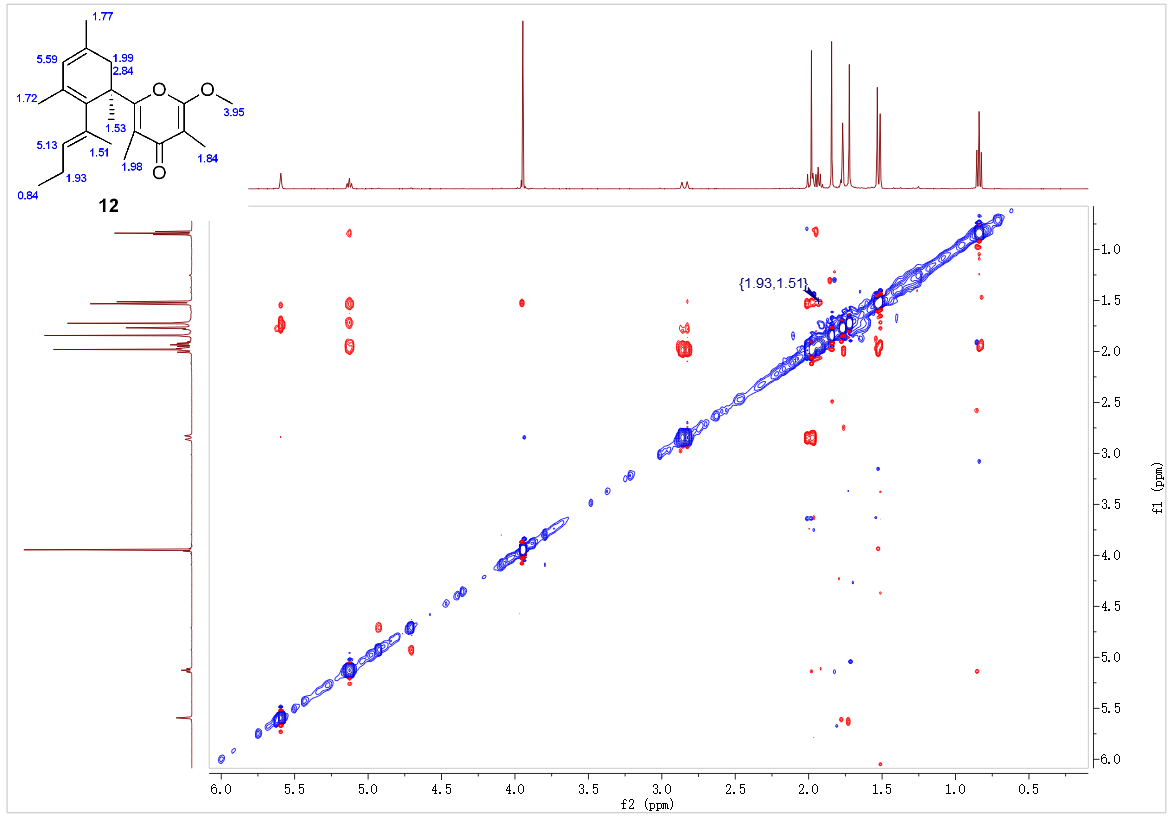
**

**Figure S7g**. HR-ESIMS (positive mode) spectrum of ocellatuspyrone G (**12**)

**
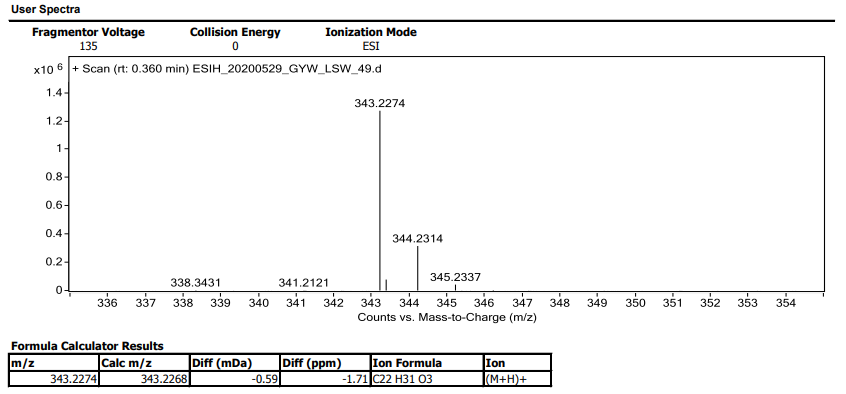
**

**Figure S7h**. IR spectrum of ocellatuspyrone G (**12**)


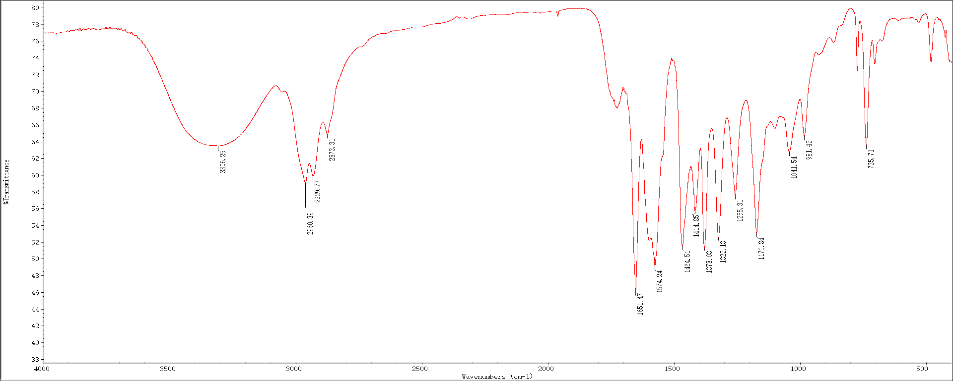


**Figure S7i**. ECD and UV spectra of ocellatuspyrone G (**12**)

**
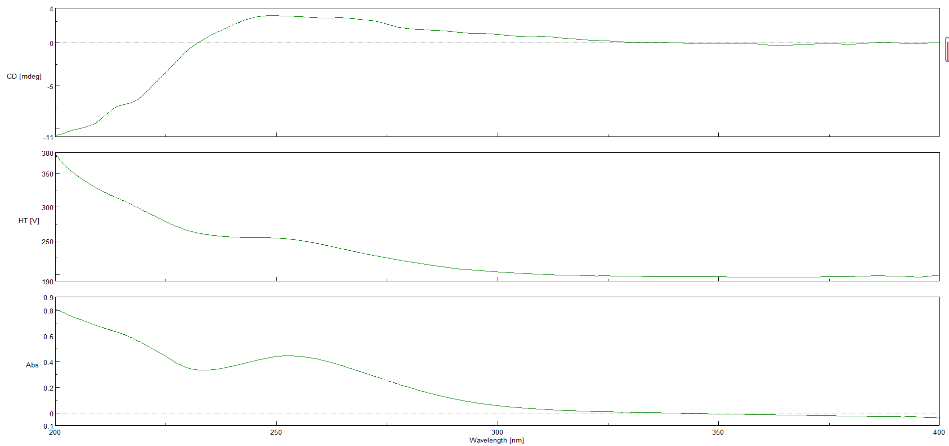
**

**Figure S7j**. Specific optical rotation of ocellatuspyrone G (**12**)

**
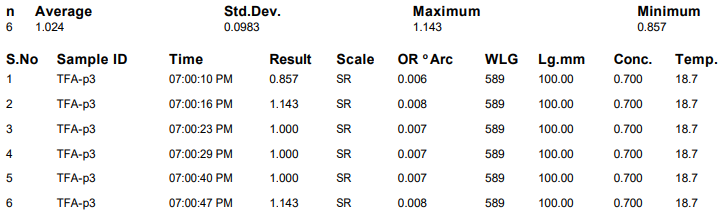
**

**Figure S8**. The comparison of ^1^H NMR spectrum (400 MHz) for the reduction product of **4** and the isolated **6** in CDCl_3_


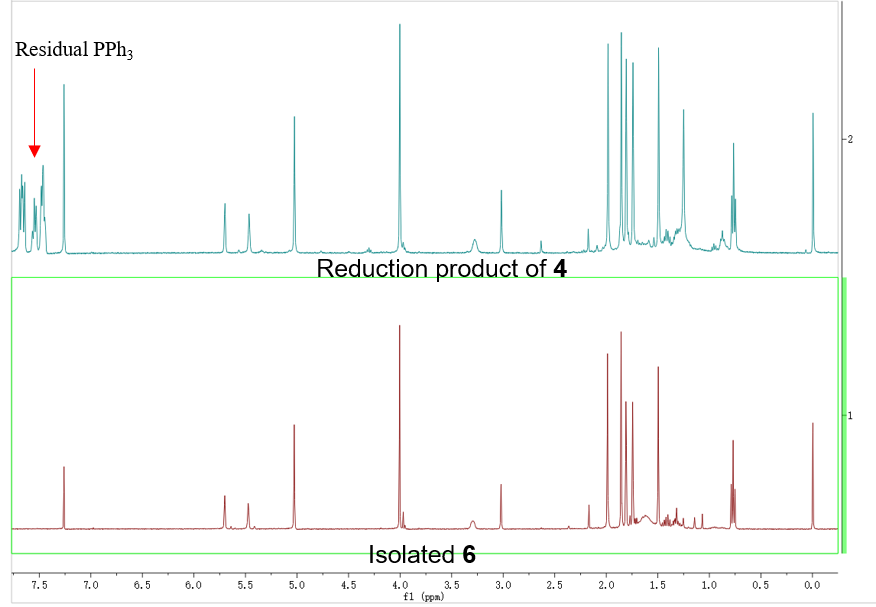


**Figure S9a**. ^1^H NMR spectrum (600 MHz) of compound **6a** in CDCl_3_

**
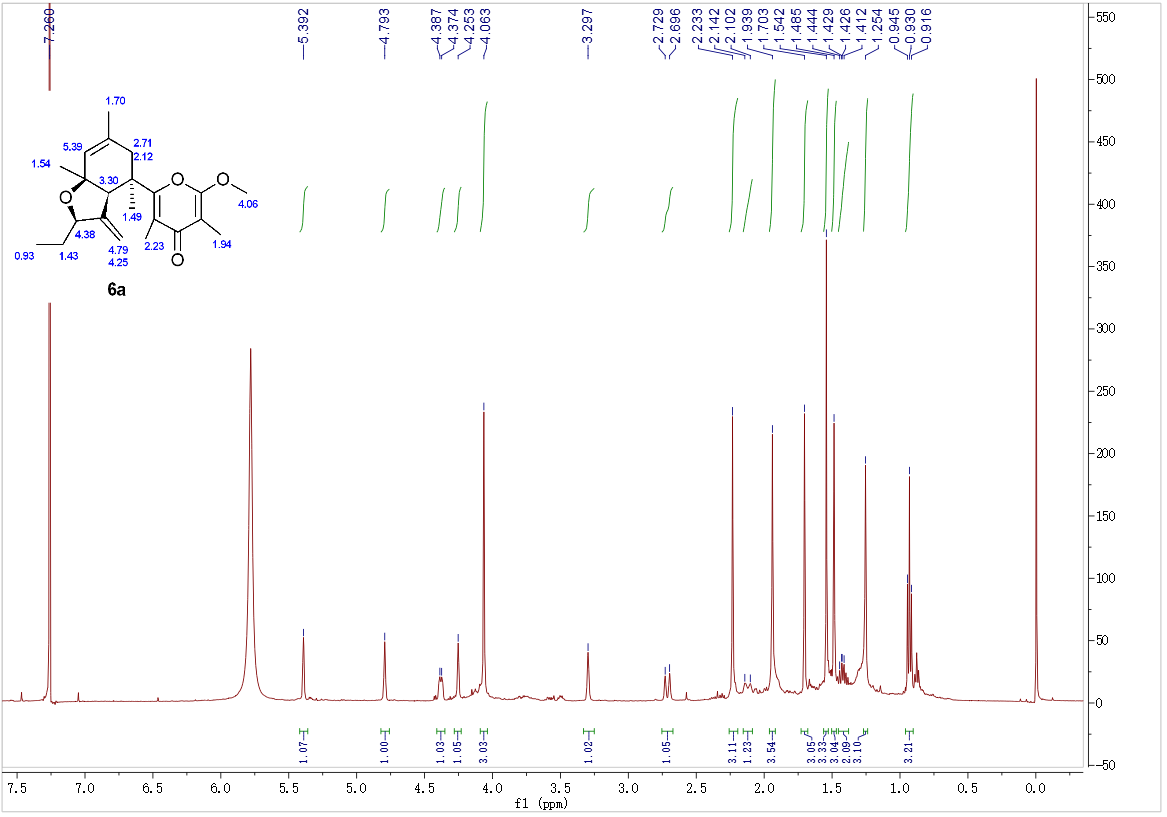
**

**Figure S9b**. ^13^C NMR spectrum (150 MHz) of compound **6a** in CDCl_3_

**
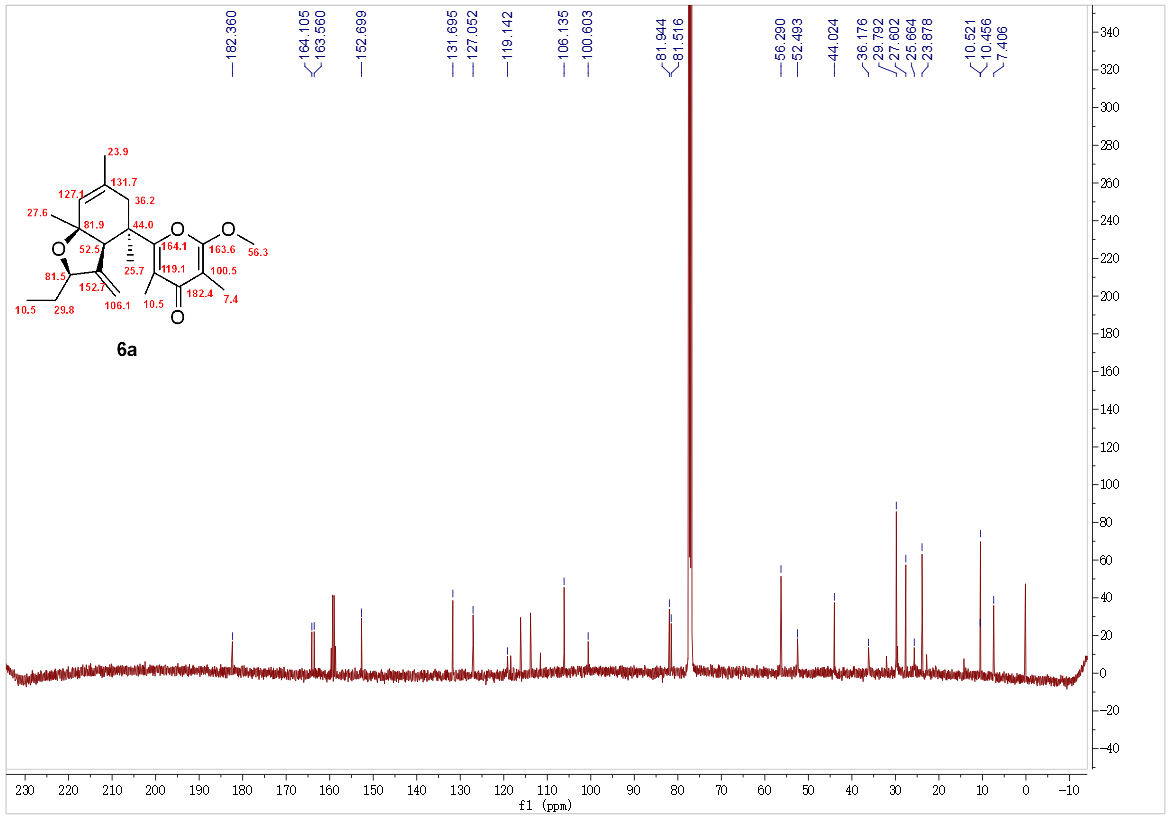
**

**Figure S9c**. HSQC spectrum (600 MHz) of compound **6a** in CDCl_3_

**
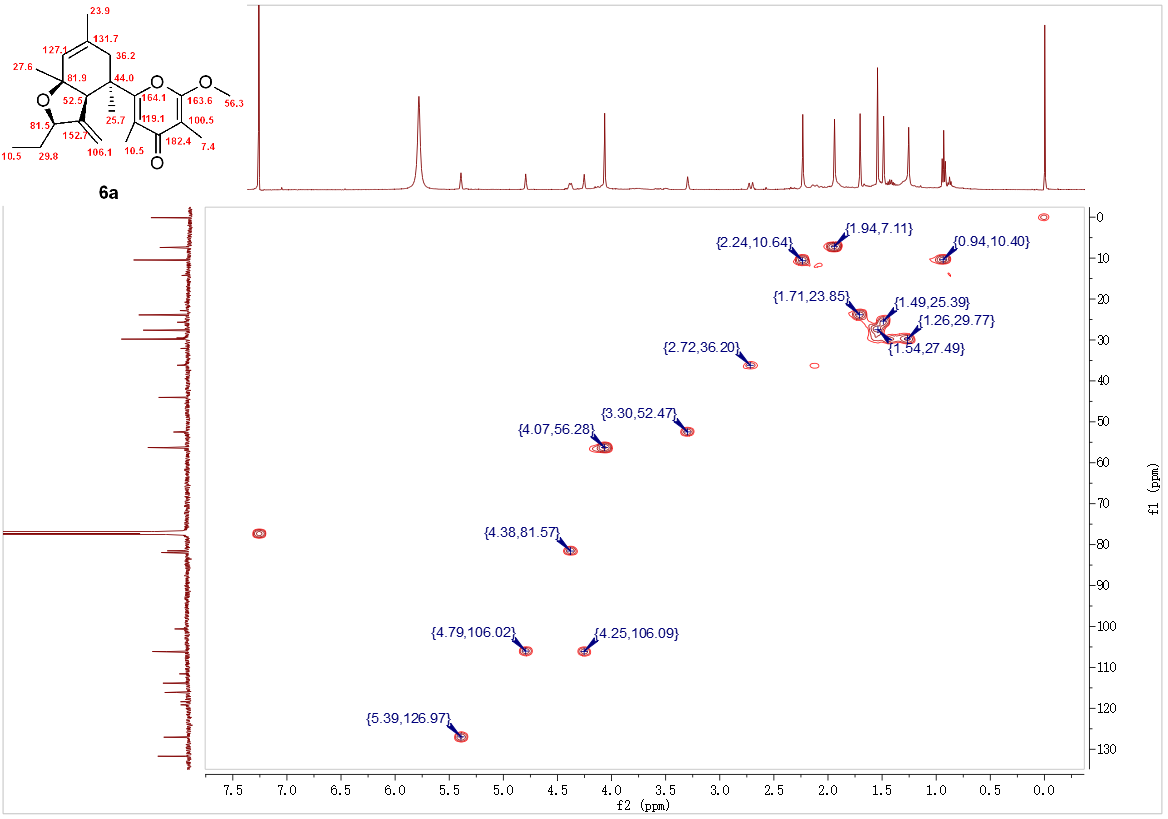
**

**Figure S9d.** HMBC spectrum (600 MHz) of compound **6a** in CDCl_3_

**
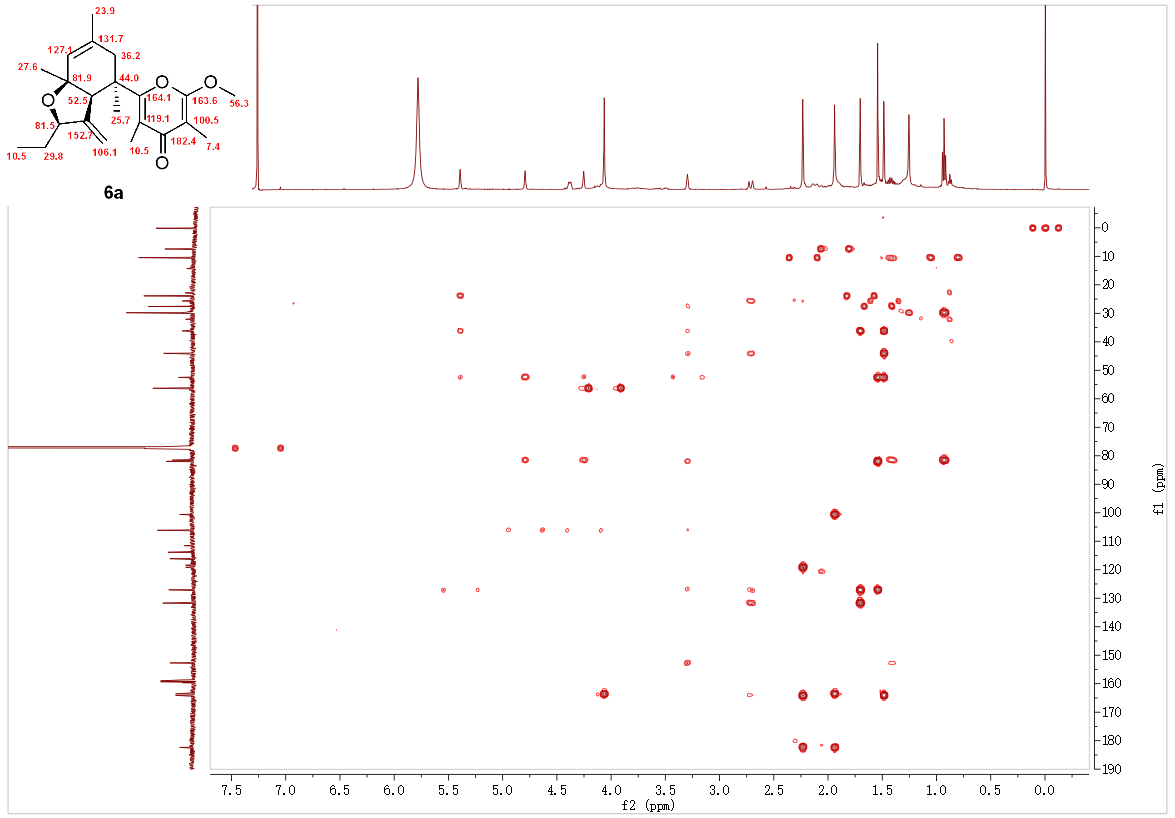
**

**Figure S9e**. ^1^H-^1^H COSY spectrum (600 MHz) of compound **6a** in CDCl_3_

**
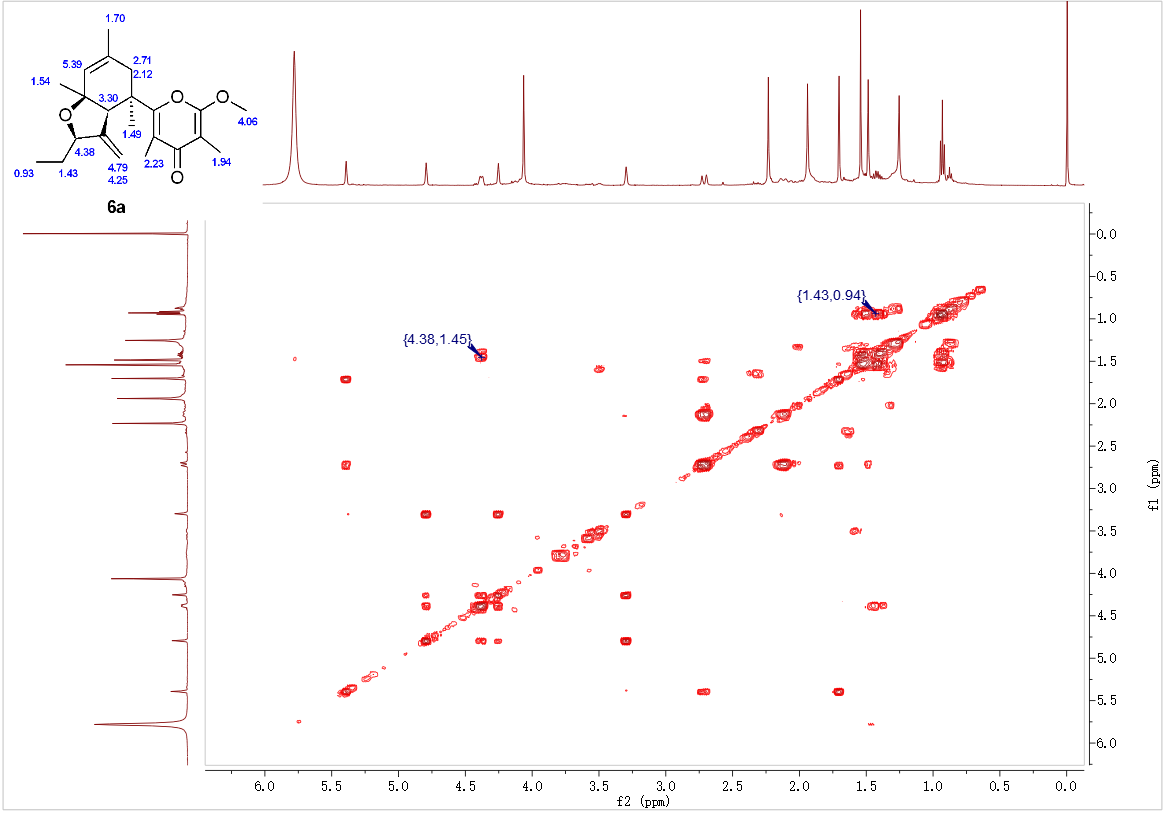
**

**Figure S9f**. NOESY spectrum (600 MHz) of compound **6a** in CDCl_3_

**
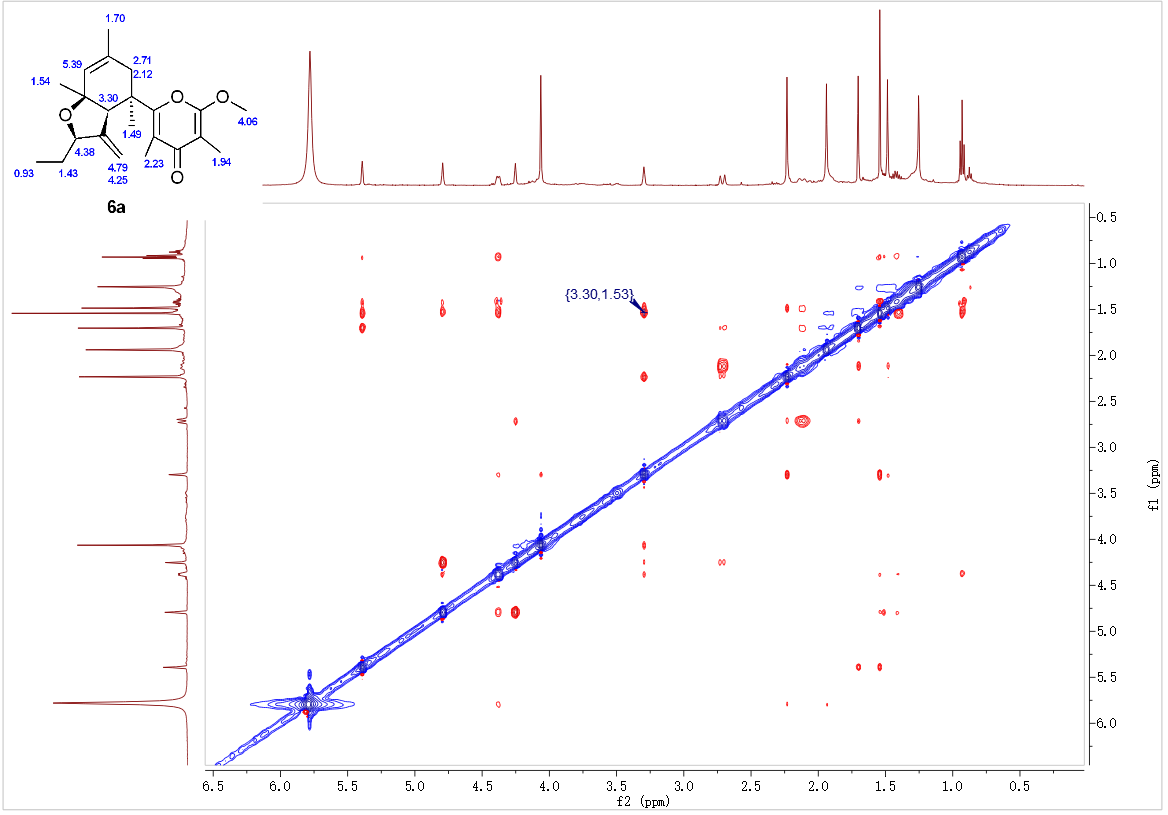
**

**Figure S9g**. HR-ESIMS (positive mode) spectrum of compound **6a**


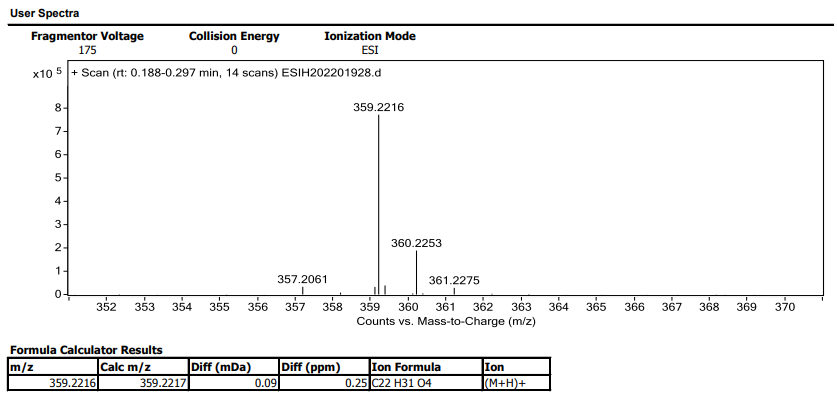


**Figure S10a**. ^1^H NMR spectrum (600 MHz) of (±)-photodeoxytridachione (**3**) in CDCl_3_

**
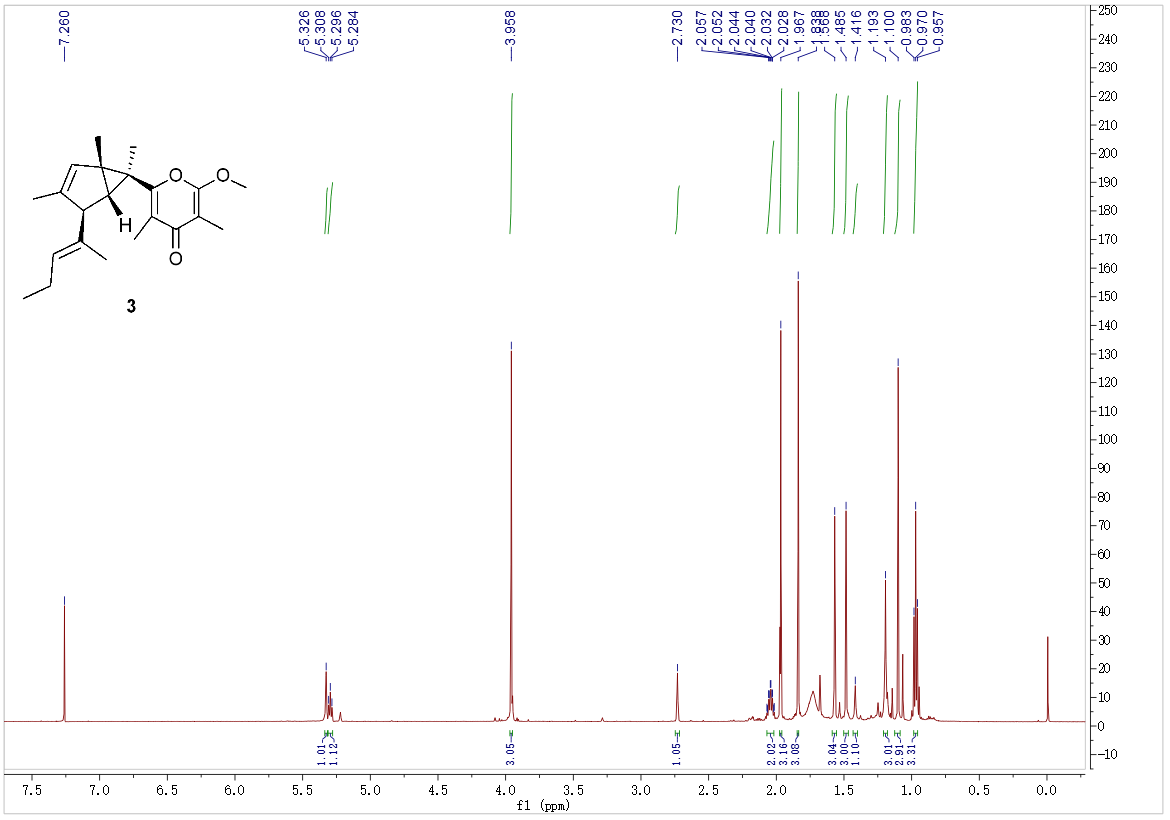
**

**Figure S10b**. ^13^C NMR spectrum (150 MHz) of (±)-photodeoxytridachione (**3**) in CDCl_3_

**
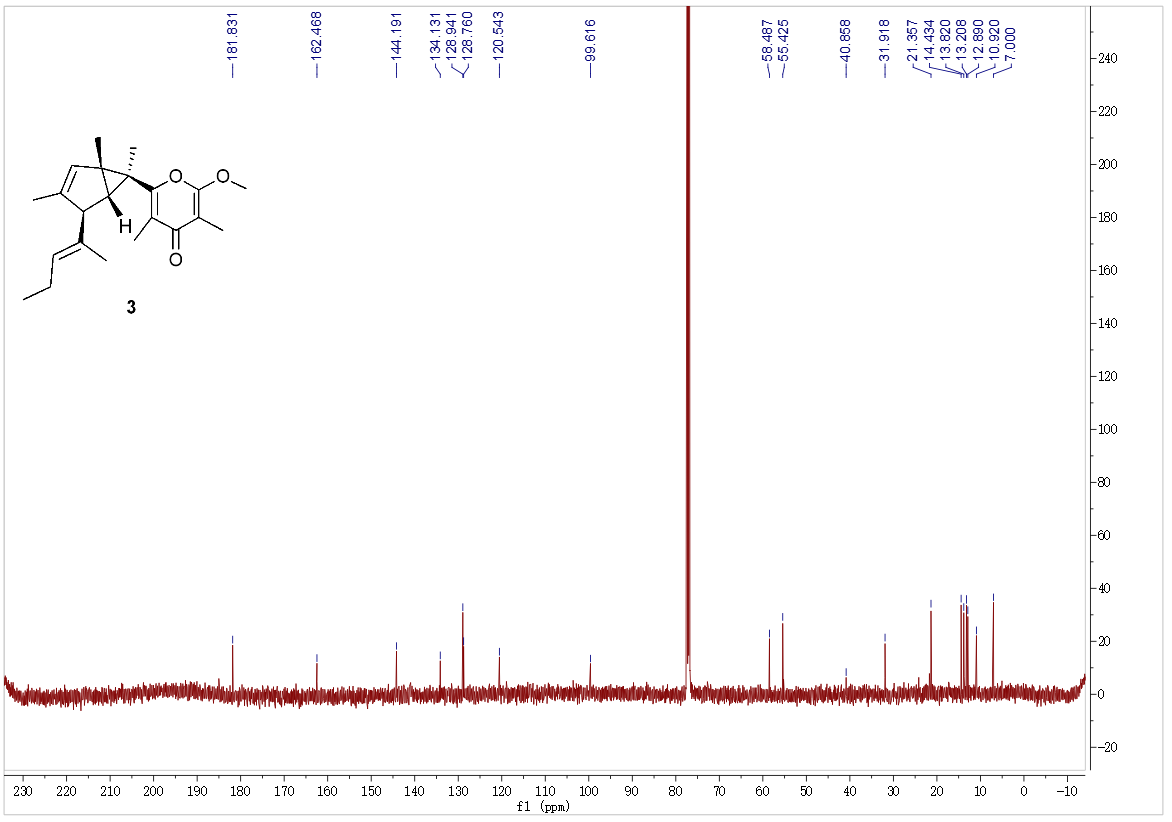
**

**Figure S10c**. ECD and UV spectrum of (±)-photodeoxytridachione (**3**)
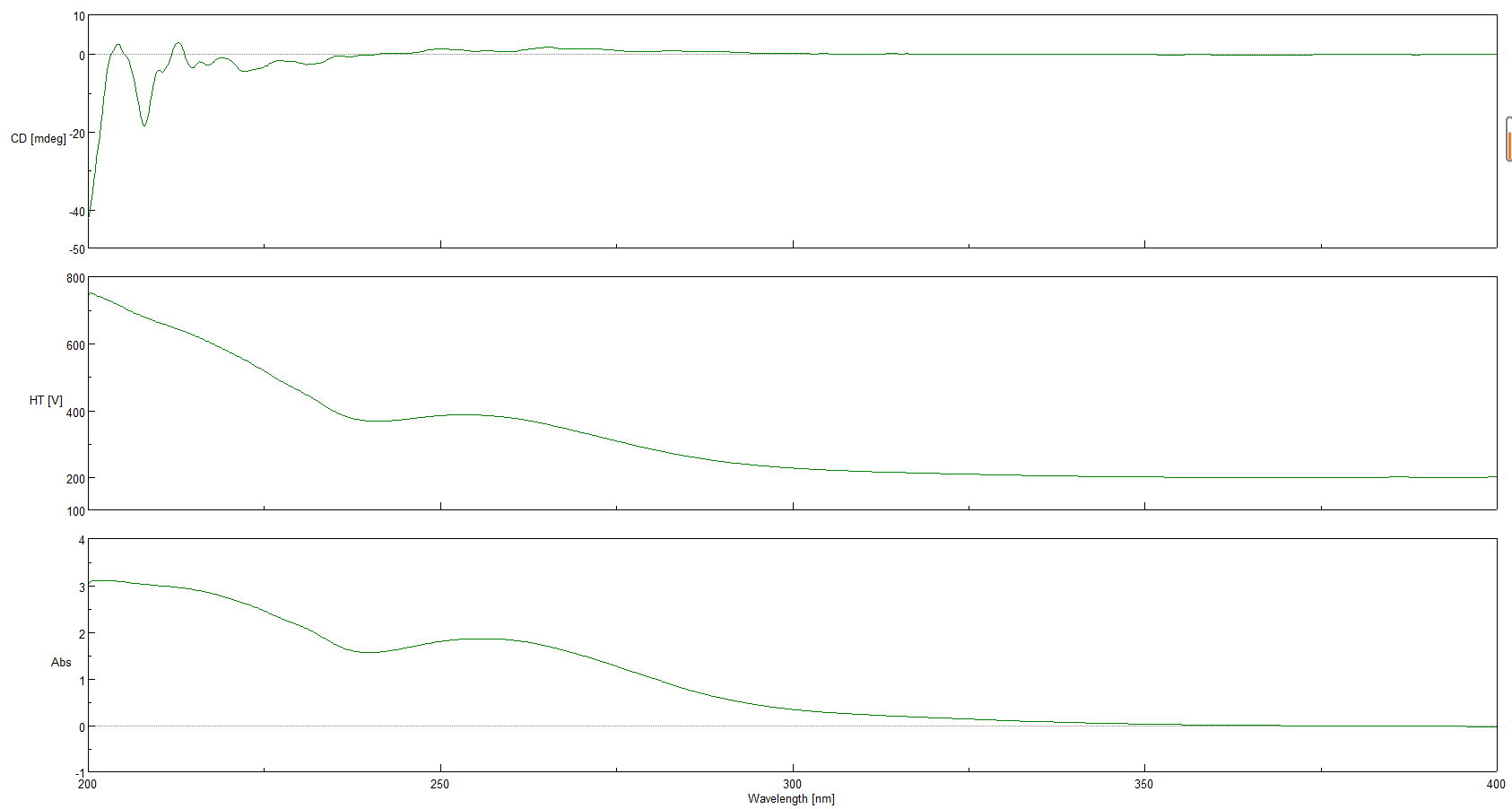


**Figure S11a**. ^1^H NMR spectrum (600 MHz) of tridachiapyrone J (**4**) in CDCl_3_

**
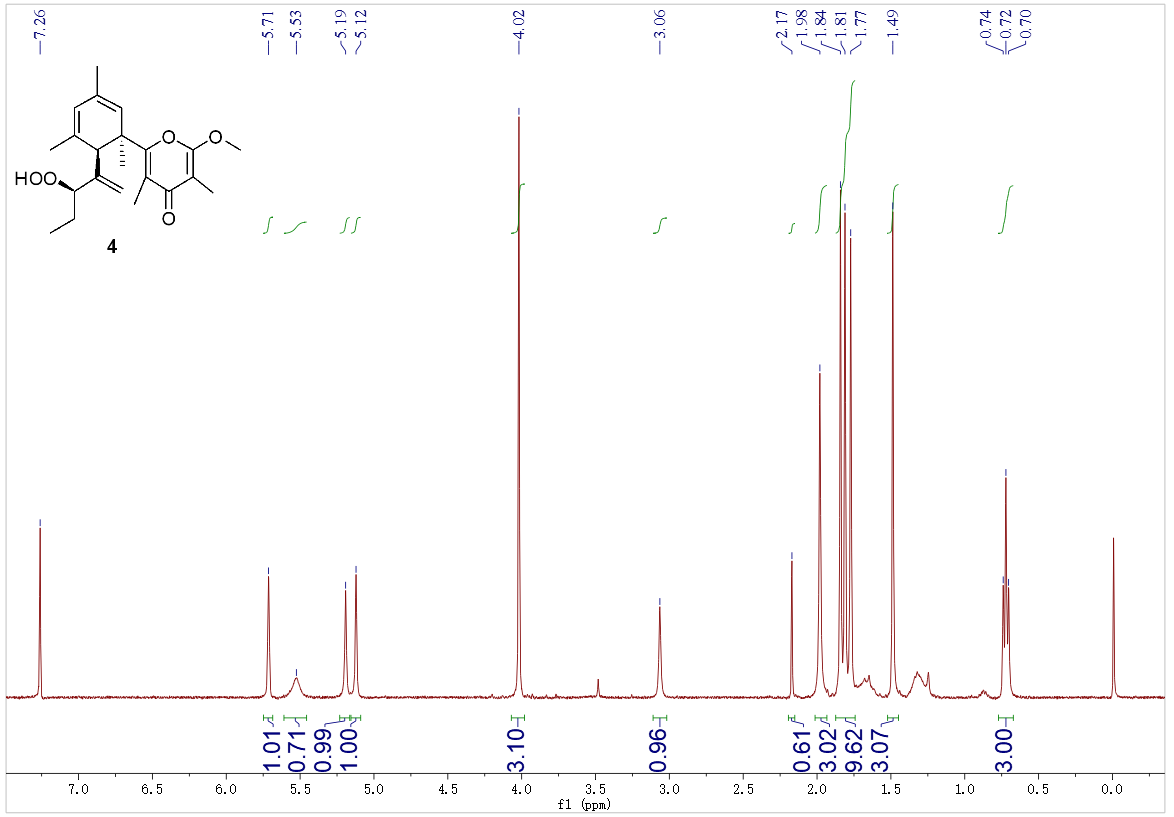
**

**Figure S11b**. ^13^C NMR spectrum (150 MHz) of tridachiapyrone J (**4**) in CDCl_3_

**
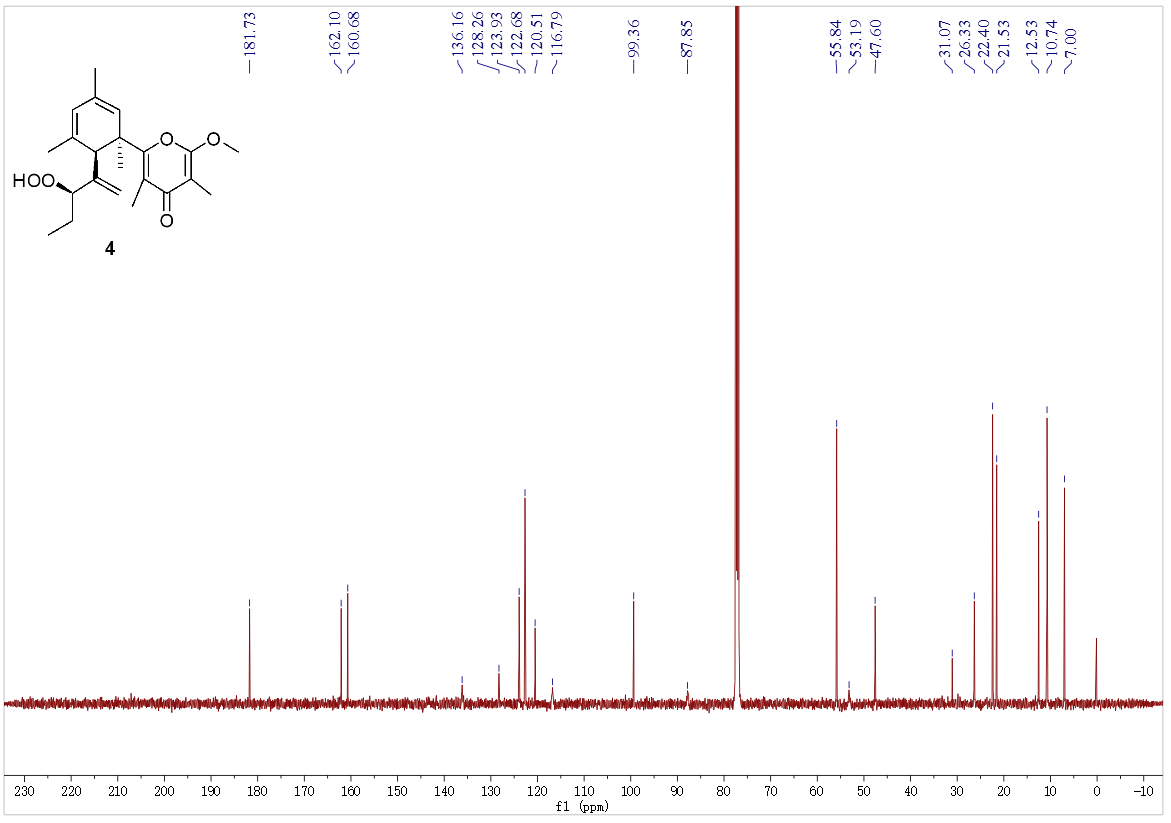
**

**Figure S11c**. ECD and UV spectra of tridachiapyrone J (**4**)


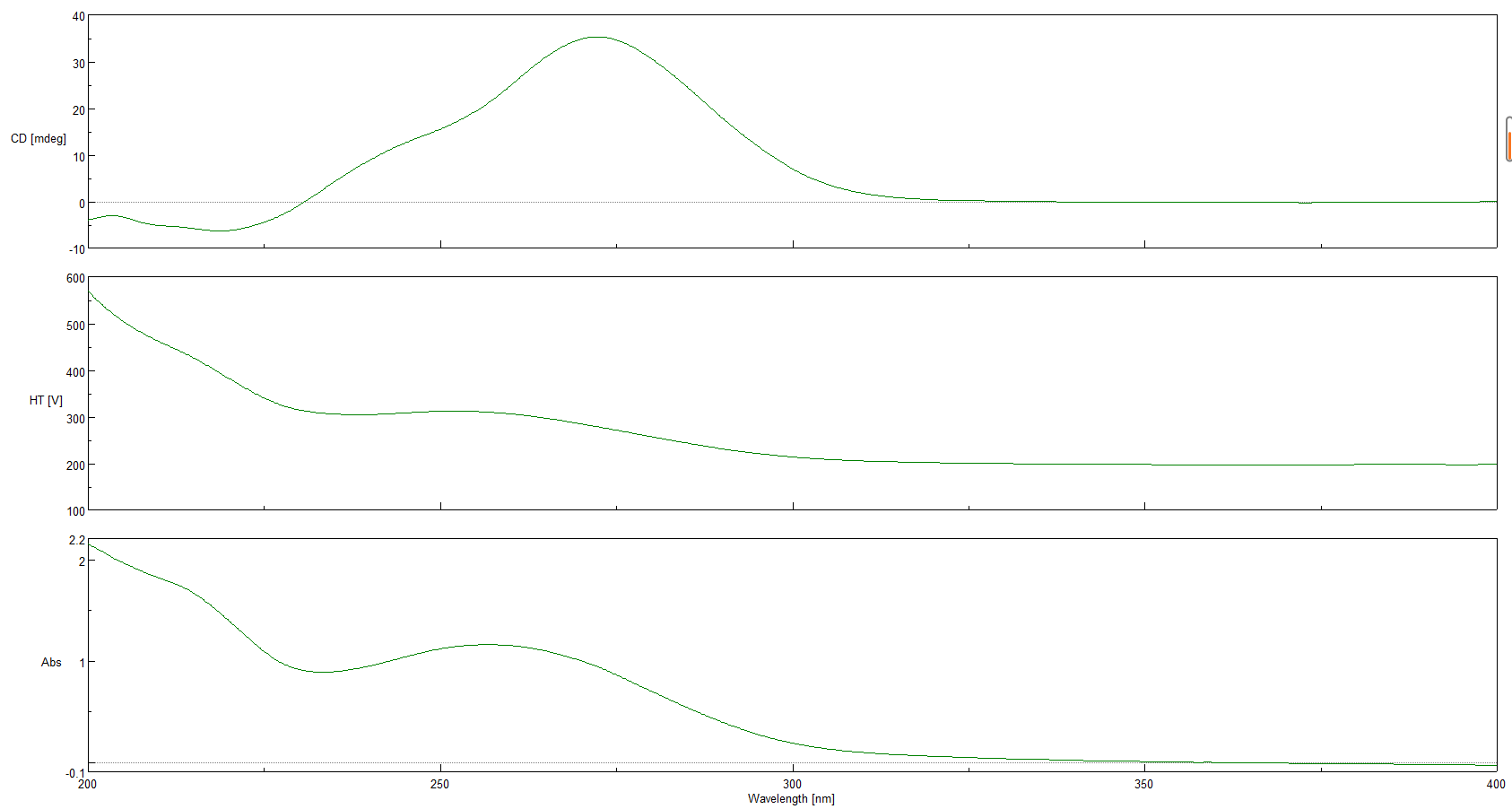


**Figure S12a**. ^1^H NMR spectrum (600 MHz) of tridachiapyrone G (**6**) in CDCl_3_

**
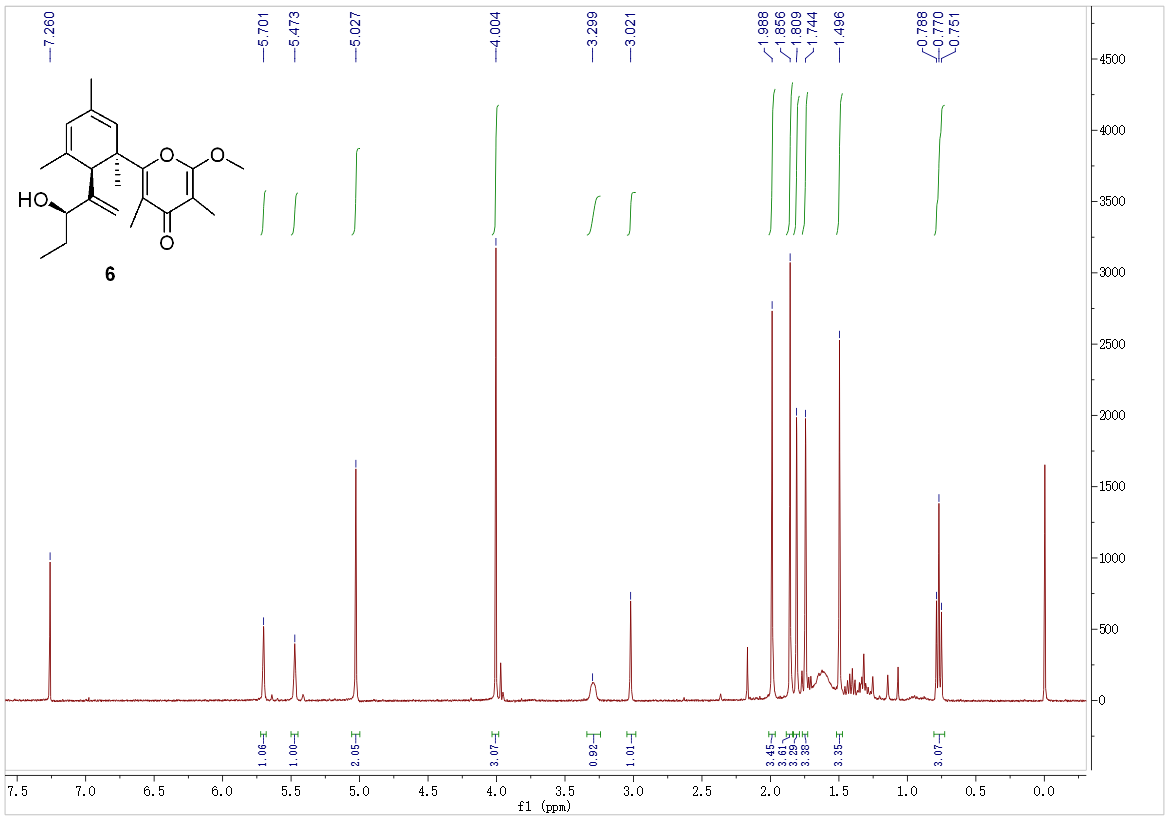
**

**Figure S12b**. ^13^C NMR spectrum (150 MHz) of tridachiapyrone G (**6**) in CDCl_3_

**
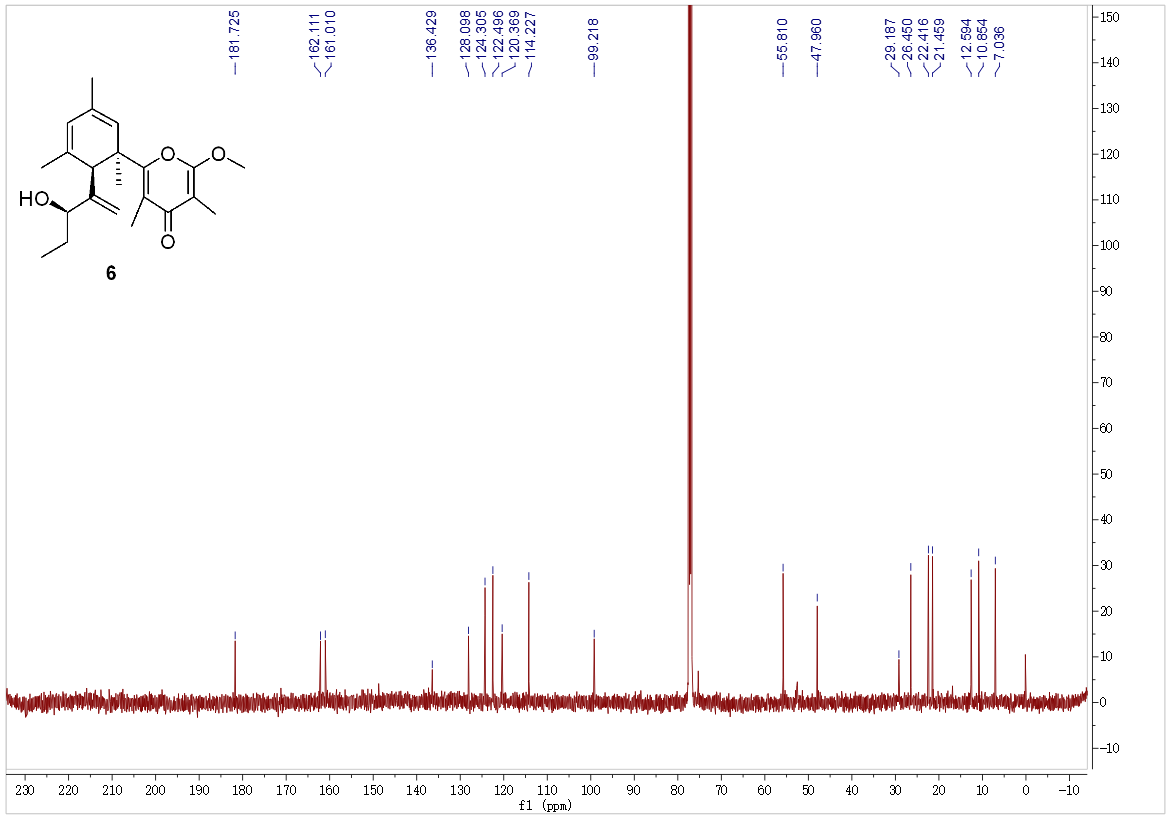
**

**Figure S12c**. ECD and UV spectra of tridachiapyrone G (**6**)


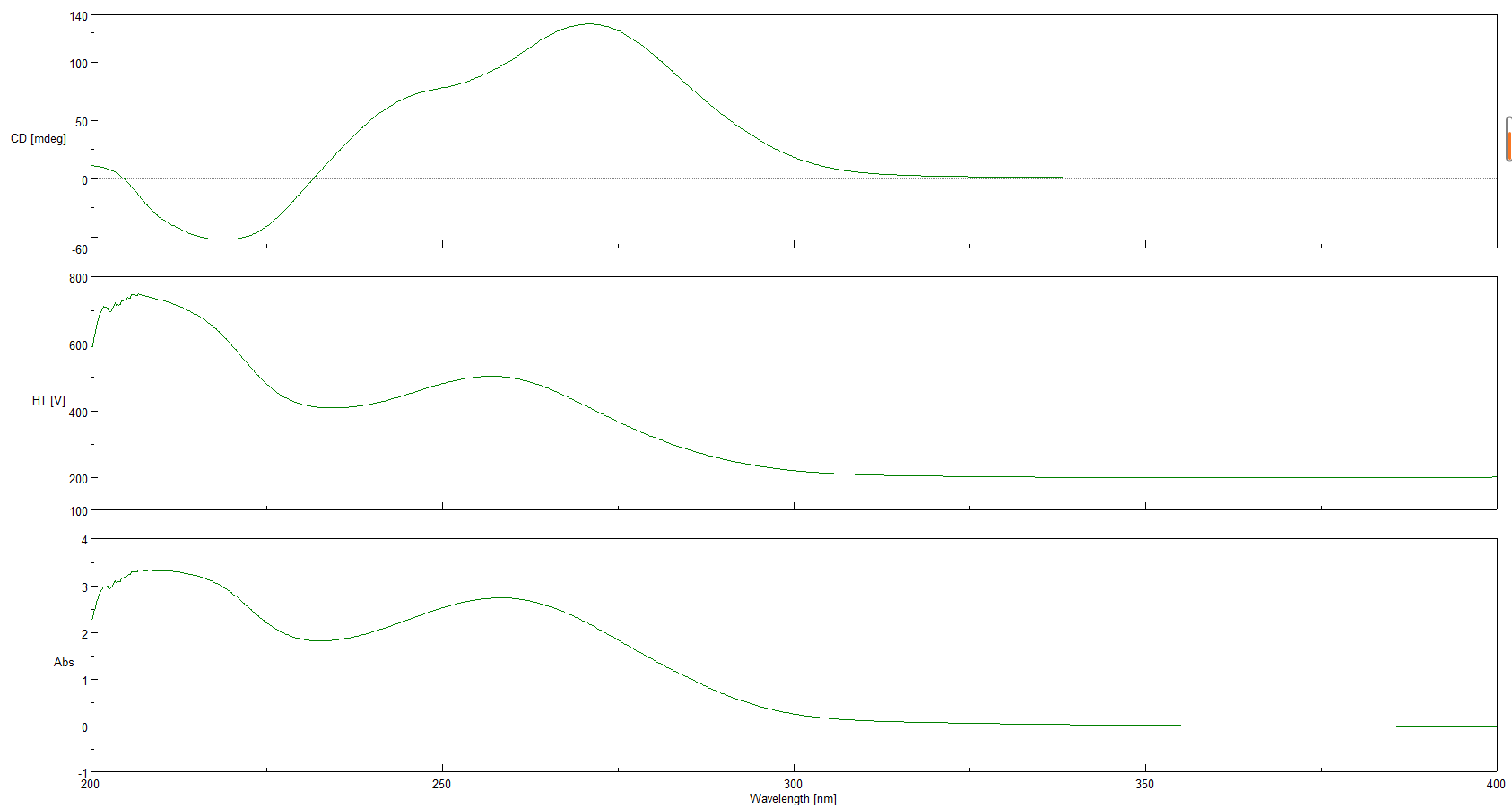


**Figure S13a**. ^1^H NMR spectrum (600 MHz) of tridachiapyrone H (**7**) in CDCl_3_

**
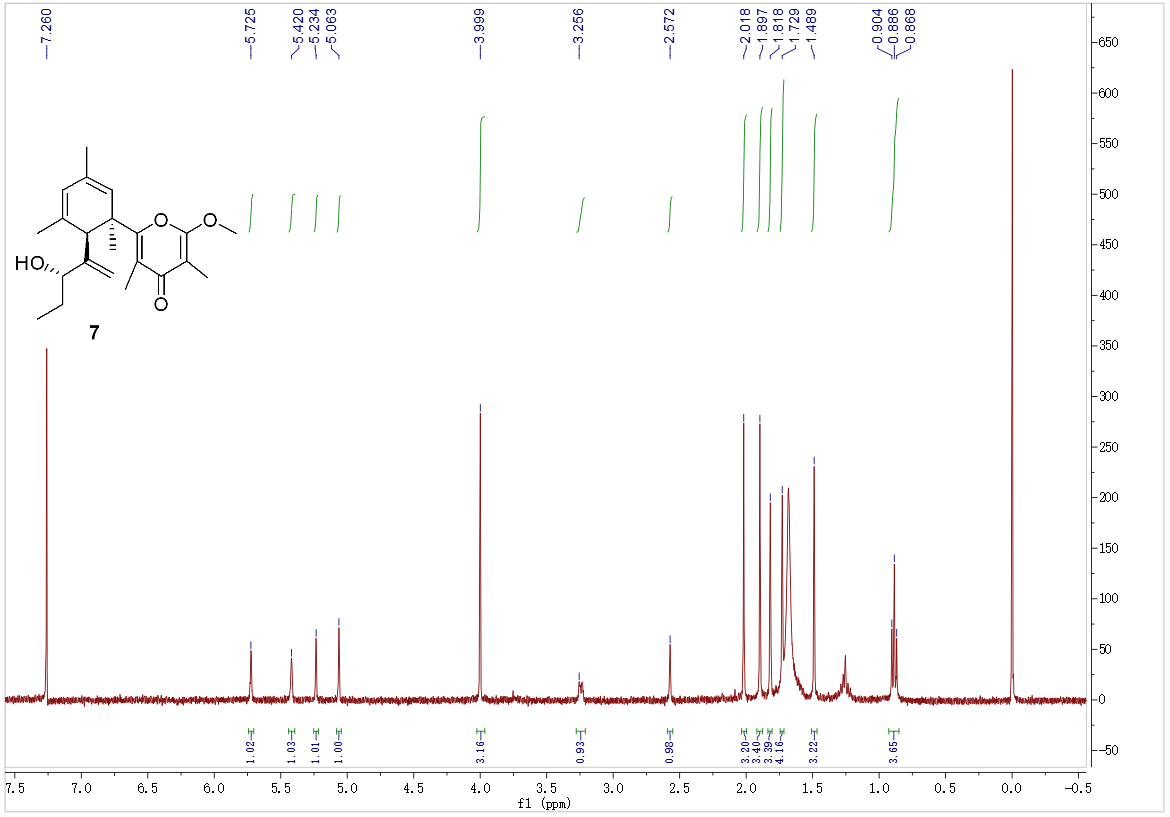
**

**Figure S13b**. ECD and UV spectra of tridachiapyrone H (**7**)


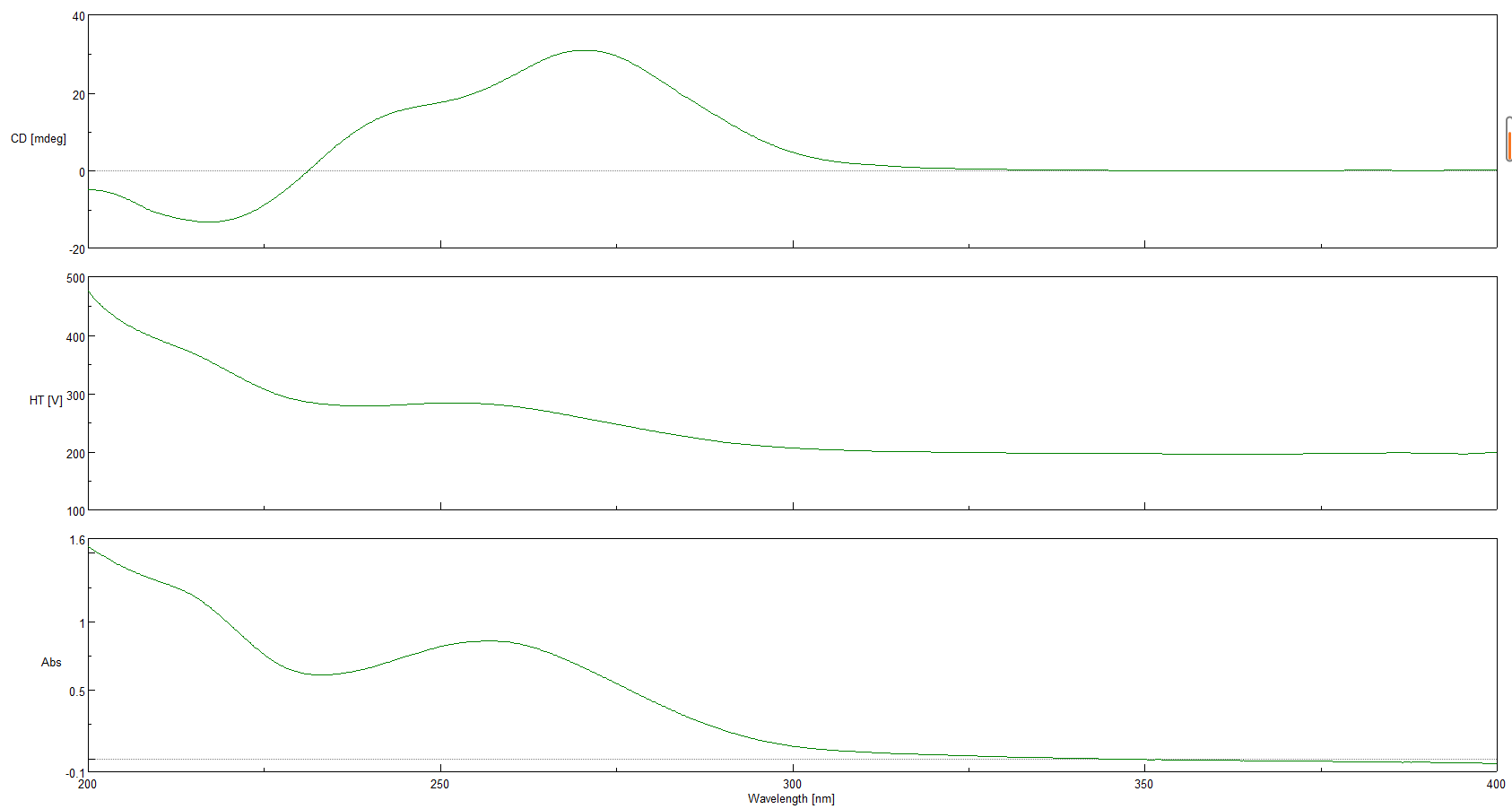


**Figure S14a**. ^1^H NMR spectrum (600 MHz) of (+)-9,10-deoxytridachione (**8**) in CDCl_3_

**Figure S14b**. ^13^C NMR spectrum (150 MHz) of (+)-9,10-deoxytridachione (**8**) in CDCl_3_

**Figure S14c**. ECD and UV spectra of (+)-9,10-deoxytridachione (**8**)

**Figure S15a**. Structures of isomers **1a** and **1b** for compound **1**

**Figure S15b**. Structures of isomers **11a** and **11b** for compound **11**

**Figure S15c**. DP4+ results obtained using experimental data of compound **1** *versus* isomers **1a** and **1b**

**Figure S15d**. DP4+ results obtained using experimental data of compound **11** *versus* isomers **11a** and **11b**

**Figure S15e**. The Cartesian Coordinates of the dominant conformers for **1a** and **1b**

**Conformer 1a1**

Calculation method: mPW1PW91

Basic Set: 6-31G(d)

E (mPW1PW91): -1156.95779088 a. u.

Boltzmann population: 10.212%

| **Conformer 1a1** | Coordinates (Angstroms) | | |
| --- | --- | --- | --- |
| Atom | X | Y | Z |
| C | 2.65487 | -1.96836 | -0.24375 |
| C | 2.30269 | -0.48322 | -0.13326 |
| C | 0.84148 | -0.51001 | 0.35372 |
| C | 0.44077 | -1.94693 | 0.60908 |
| C | 1.63158 | -2.73559 | 0.1646 |
| C | -0.24764 | -1.11809 | -0.51524 |
| C | -0.02499 | -1.39883 | -1.99915 |
| C | -1.62477 | -0.57612 | -0.26481 |
| C | -0.29358 | -2.40738 | 1.85246 |
| C | 3.20381 | 0.3255 | 0.79995 |
| C | 3.95119 | -2.43815 | -0.83603 |
| C | 3.86929 | -0.24114 | 1.81344 |
| C | 3.26746 | 1.83767 | 0.62015 |
| O | 1.99144 | 2.44385 | 0.90847 |
| C | 3.65934 | 2.32572 | -0.77727 |
| C | 5.05145 | 1.86637 | -1.21889 |
| C | -2.80249 | -1.24051 | -0.24065 |
| C | -4.0711 | -0.49849 | -0.05879 |
| C | -3.96743 | 0.952 | 0.05979 |
| C | -2.72993 | 1.51109 | 0.02221 |
| O | -1.59144 | 0.80074 | -0.13317 |
| C | -2.92563 | -2.72891 | -0.40684 |
| C | -5.22533 | 1.75671 | 0.2211 |
| O | -5.15728 | -1.08948 | -0.02865 |
| O | -2.51654 | 2.82783 | 0.1713 |
| C | -1.30058 | 3.39173 | -0.35213 |
| H | 2.35606 | -0.02802 | -1.13319 |
| H | 0.53 | 0.27396 | 1.03266 |
| H | 1.63776 | -3.82354 | 0.15429 |
| H | -0.10873 | -0.46796 | -2.57349 |
| H | 0.95715 | -1.83423 | -2.19331 |
| H | -0.78162 | -2.09207 | -2.38194 |
| H | 0.39885 | -2.45277 | 2.70182 |
| H | -1.1116 | -1.73176 | 2.11781 |
| H | -0.72142 | -3.40831 | 1.71939 |
| H | 4.03675 | -3.52877 | -0.79435 |
| H | 4.03637 | -2.13664 | -1.88983 |
| H | 4.81323 | -2.00504 | -0.31481 |
| H | 4.48506 | 0.34934 | 2.48842 |
| H | 3.82303 | -1.30931 | 2.00694 |
| H | 4.01506 | 2.2234 | 1.33163 |
| H | 1.76391 | 2.20465 | 1.82106 |
| H | 2.90037 | 2.00357 | -1.50052 |
| H | 3.61245 | 3.42113 | -0.75564 |
| H | 5.30307 | 2.28162 | -2.2005 |
| H | 5.82174 | 2.19558 | -0.51098 |
| H | 5.11627 | 0.77493 | -1.29021 |
| H | -3.45656 | -3.15954 | 0.44908 |
| H | -3.53885 | -2.95677 | -1.28646 |
| H | -1.95646 | -3.22021 | -0.51165 |
| H | -5.0419 | 2.82393 | 0.07862 |
| H | -5.97746 | 1.41432 | -0.49696 |
| H | -5.65884 | 1.60958 | 1.21836 |
| H | -1.37693 | 4.45993 | -0.14601 |
| H | -0.41328 | 2.97697 | 0.1314 |
| H | -1.23562 | 3.22809 | -1.43365 |

**Conformer 1a2**

Calculation method: mPW1PW91

Basic Set: 6-31G(d)

E (mPW1PW91): -1156.95797773 a. u.

Boltzmann population: 9.213%

| **Conformer 1a2** | Coordinates (Angstroms) | | |
| --- | --- | --- | --- |
| Atom | X | Y | Z |
| C | 2.41475 | -1.94278 | -0.18095 |
| C | 2.33807 | -0.41612 | -0.1196 |
| C | 0.89578 | -0.16032 | 0.36286 |
| C | 0.22716 | -1.49499 | 0.61586 |
| C | 1.25846 | -2.49963 | 0.21336 |
| C | -0.27605 | -0.55796 | -0.51901 |
| C | -0.10129 | -0.90785 | -1.99383 |
| C | -1.57222 | 0.1496 | -0.2769 |
| C | -0.61499 | -1.79159 | 1.84144 |
| C | 3.36981 | 0.2466 | 0.79144 |
| C | 3.62567 | -2.65637 | -0.70378 |
| C | 3.72376 | -0.28075 | 1.9683 |
| C | 3.94055 | 1.57643 | 0.32383 |
| O | 2.90236 | 2.44927 | -0.14517 |
| C | 4.93732 | 1.45126 | -0.83824 |
| C | 6.25289 | 0.77076 | -0.45326 |
| C | -1.82623 | 1.47226 | -0.15201 |
| C | -3.21752 | 1.94107 | 0.06656 |
| C | -4.25856 | 0.92241 | 0.08022 |
| C | -3.88766 | -0.37213 | -0.09692 |
| O | -2.61073 | -0.77259 | -0.26608 |
| C | -0.78394 | 2.5546 | -0.23426 |
| C | -5.68454 | 1.35215 | 0.27605 |
| O | -3.4698 | 3.14255 | 0.21586 |
| O | -4.77416 | -1.38257 | -0.10911 |
| C | -4.31237 | -2.71413 | -0.35841 |
| H | 2.46936 | -0.00183 | -1.12882 |
| H | 0.73635 | 0.65143 | 1.06504 |
| H | 1.06599 | -3.57035 | 0.22907 |
| H | -0.98626 | -1.4369 | -2.36514 |
| H | 0.01768 | 0.0038 | -2.59135 |
| H | 0.76659 | -1.54804 | -2.16398 |
| H | 0.03199 | -2.03053 | 2.69418 |
| H | -1.23991 | -0.93949 | 2.12453 |
| H | -1.27734 | -2.65043 | 1.67533 |
| H | 3.49809 | -3.74289 | -0.66317 |
| H | 3.8308 | -2.38082 | -1.7478 |
| H | 4.5214 | -2.39427 | -0.12753 |
| H | 4.4209 | 0.22549 | 2.63211 |
| H | 3.33391 | -1.23758 | 2.30652 |
| H | 4.46726 | 2.04556 | 1.17054 |
| H | 2.30336 | 2.62523 | 0.59735 |
| H | 4.45989 | 0.91647 | -1.66937 |
| H | 5.12847 | 2.46933 | -1.19815 |
| H | 6.92667 | 0.71463 | -1.31493 |
| H | 6.77143 | 1.32662 | 0.33767 |
| H | 6.0881 | -0.24818 | -0.08702 |
| H | -1.17015 | 3.36752 | -0.85656 |
| H | -0.60919 | 2.99869 | 0.75525 |
| H | 0.16642 | 2.20908 | -0.64304 |
| H | -6.37573 | 0.50973 | 0.20734 |
| H | -5.80819 | 1.83351 | 1.25335 |
| H | -5.95798 | 2.10325 | -0.47351 |
| H | -5.21076 | -3.33209 | -0.33863 |
| H | -3.82845 | -2.79064 | -1.33706 |
| H | -3.61506 | -3.04477 | 0.41722 |

**Conformer 1a3**

Calculation method: mPW1PW91

Basic Set: 6-31G(d)

E (mPW1PW91): -1156.95654725 a. u.

Boltzmann population: 8.776%

| **Conformer 1a3** | Coordinates (Angstroms) | | |
| --- | --- | --- | --- |
| Atom | X | Y | Z |
| C | 2.56588 | -2.18483 | -0.30262 |
| C | 2.30374 | -0.68057 | -0.41549 |
| C | 0.88952 | -0.52583 | 0.18144 |
| C | 0.43326 | -1.88092 | 0.68382 |
| C | 1.53993 | -2.80814 | 0.29668 |
| C | -0.29839 | -1.18786 | -0.50276 |
| C | -0.19539 | -1.691 | -1.94063 |
| C | -1.64589 | -0.58437 | -0.23305 |
| C | -0.23207 | -2.09199 | 2.02934 |
| C | 3.35433 | 0.17596 | 0.29274 |
| C | 3.8053 | -2.81249 | -0.86616 |
| C | 3.78766 | -0.11148 | 1.52461 |
| C | 3.88864 | 1.36977 | -0.48868 |
| O | 5.09089 | 1.90285 | 0.05409 |
| C | 2.89301 | 2.54256 | -0.57956 |
| C | 2.60046 | 3.2202 | 0.76199 |
| C | -2.82346 | -1.23119 | -0.07678 |
| C | -4.07988 | -0.46631 | 0.09067 |
| C | -3.96534 | 0.98551 | 0.06097 |
| C | -2.73028 | 1.52712 | -0.10189 |
| O | -1.59895 | 0.80142 | -0.24319 |
| C | -2.96148 | -2.72772 | -0.07352 |
| C | -5.21151 | 1.81048 | 0.21226 |
| O | -5.16427 | -1.0423 | 0.23924 |
| O | -2.52578 | 2.8549 | -0.13142 |
| C | -1.21421 | 3.35654 | -0.40685 |
| H | 2.30234 | -0.39603 | -1.47991 |
| H | 0.68922 | 0.36335 | 0.77114 |
| H | 1.4919 | -3.87966 | 0.47803 |
| H | -0.26926 | -0.84818 | -2.63913 |
| H | 0.74682 | -2.20971 | -2.12728 |
| H | -1.01114 | -2.38344 | -2.17215 |
| H | -0.74081 | -3.06229 | 2.07807 |
| H | 0.51961 | -2.06982 | 2.82758 |
| H | -0.97571 | -1.31931 | 2.24343 |
| H | 3.83784 | -3.88735 | -0.6623 |
| H | 3.86287 | -2.6755 | -1.9553 |
| H | 4.7074 | -2.35503 | -0.44114 |
| H | 4.51074 | 0.52145 | 2.0298 |
| H | 3.42276 | -0.98117 | 2.06428 |
| H | 4.07216 | 1.02264 | -1.52233 |
| H | 5.71392 | 1.16569 | 0.15454 |
| H | 3.31933 | 3.27542 | -1.27558 |
| H | 1.96475 | 2.17531 | -1.03634 |
| H | 1.91815 | 4.06707 | 0.62532 |
| H | 2.14284 | 2.52527 | 1.47489 |
| H | 3.52283 | 3.59851 | 1.21111 |
| H | -3.49513 | -3.0484 | 0.82733 |
| H | -3.58031 | -3.04978 | -0.91952 |
| H | -1.99886 | -3.23948 | -0.12368 |
| H | -5.01527 | 2.87412 | 0.06317 |
| H | -5.96878 | 1.47826 | -0.50613 |
| H | -5.64836 | 1.66898 | 1.20824 |
| H | -1.3249 | 4.4414 | -0.42241 |
| H | -0.50561 | 3.06676 | 0.37438 |
| H | -0.84959 | 3.00537 | -1.37726 |

**Conformer 1a4**

Calculation method: mPW1PW91

Basic Set: 6-31G(d)

E (mPW1PW91): -1156.9584671 a. u.

Boltzmann population: 8.177%

| **Conformer 1a4** | Coordinates (Angstroms) | | |
| --- | --- | --- | --- |
| Atom | X | Y | Z |
| C | 2.31713 | -2.16799 | -0.19852 |
| C | 2.33012 | -0.64436 | -0.06466 |
| C | 0.89803 | -0.32417 | 0.40841 |
| C | 0.14498 | -1.62614 | 0.58246 |
| C | 1.12198 | -2.67167 | 0.14864 |
| C | -0.27782 | -0.6044 | -0.51171 |
| C | -0.09594 | -0.89269 | -1.99881 |
| C | -1.53316 | 0.1688 | -0.25672 |
| C | -0.73885 | -1.9294 | 1.77651 |
| C | 3.38622 | -0.0929 | 0.89027 |
| C | 3.49608 | -2.92418 | -0.73446 |
| C | 3.68274 | -0.69215 | 2.04819 |
| C | 4.06202 | 1.20855 | 0.48889 |
| O | 3.09997 | 2.17905 | 0.04967 |
| C | 5.06627 | 1.036 | -0.65907 |
| C | 5.79788 | 2.3344 | -1.01421 |
| C | -1.70848 | 1.49594 | -0.06175 |
| C | -3.07355 | 2.03782 | 0.15319 |
| C | -4.17511 | 1.0876 | 0.08485 |
| C | -3.88048 | -0.21538 | -0.16017 |
| O | -2.62661 | -0.68568 | -0.3218 |
| C | -0.60049 | 2.51457 | -0.05786 |
| C | -5.57655 | 1.5946 | 0.27317 |
| O | -3.25558 | 3.24235 | 0.36675 |
| O | -4.82768 | -1.16435 | -0.25645 |
| C | -4.44041 | -2.51236 | -0.54135 |
| H | 2.50267 | -0.19155 | -1.05066 |
| H | 0.77623 | 0.4598 | 1.14851 |
| H | 0.86579 | -3.72838 | 0.11008 |
| H | 0.07804 | 0.03899 | -2.55015 |
| H | 0.7417 | -1.56722 | -2.1864 |
| H | -0.9997 | -1.35858 | -2.40751 |
| H | -0.12626 | -2.2552 | 2.62589 |
| H | -1.31139 | -1.05299 | 2.09422 |
| H | -1.45297 | -2.73215 | 1.55413 |
| H | 3.31576 | -4.00392 | -0.72806 |
| H | 3.72231 | -2.62754 | -1.76841 |
| H | 4.39849 | -2.72178 | -0.14476 |
| H | 4.40259 | -0.26533 | 2.74327 |
| H | 3.22325 | -1.63377 | 2.33792 |
| H | 4.61099 | 1.59823 | 1.3614 |
| H | 2.53584 | 2.40305 | 0.80603 |
| H | 5.78542 | 0.26212 | -0.36325 |
| H | 4.53644 | 0.6577 | -1.54276 |
| H | 6.5018 | 2.17216 | -1.83801 |
| H | 5.08773 | 3.10946 | -1.31455 |
| H | 6.36866 | 2.71323 | -0.15721 |
| H | -0.92462 | 3.38704 | -0.63314 |
| H | -0.41883 | 2.88373 | 0.96078 |
| H | 0.33571 | 2.13762 | -0.47168 |
| H | -6.31421 | 0.79599 | 0.17227 |
| H | -5.68489 | 2.05647 | 1.26141 |
| H | -5.79679 | 2.37975 | -0.4591 |
| H | -5.37622 | -3.07055 | -0.58672 |
| H | -3.91763 | -2.58216 | -1.50019 |
| H | -3.80236 | -2.91814 | 0.24963 |

**Conformer 1a5**

Calculation method: mPW1PW91

Basic Set: 6-31G(d)

E (mPW1PW91): -1156.95785669 a. u.

Boltzmann population: 7.534%

| Conformer 1a5 | Coordinates (Angstroms) | | |
| --- | --- | --- | --- |
| Atom | X | Y | Z |
| C | 2.55231 | -2.19157 | -0.34952 |
| C | 2.28619 | -0.69497 | -0.1774 |
| C | 0.83244 | -0.65918 | 0.32843 |
| C | 0.35334 | -2.07987 | 0.53485 |
| C | 1.49307 | -2.91643 | 0.04456 |
| C | -0.29938 | -1.17124 | -0.54808 |
| C | -0.11121 | -1.40788 | -2.04449 |
| C | -1.63776 | -0.5551 | -0.26199 |
| C | -0.39121 | -2.54678 | 1.76983 |
| C | 3.24313 | 0.01657 | 0.77923 |
| C | 3.81789 | -2.70545 | -0.97017 |
| C | 3.82741 | -0.61835 | 1.80175 |
| C | 3.46656 | 1.51109 | 0.59707 |
| O | 2.2346 | 2.24172 | 0.7615 |
| C | 4.03507 | 1.91359 | -0.76756 |
| C | 4.41779 | 3.39563 | -0.83787 |
| C | -2.85396 | -1.1461 | -0.23247 |
| C | -4.07088 | -0.33108 | -0.01424 |
| C | -3.8759 | 1.10891 | 0.12669 |
| C | -2.60734 | 1.59079 | 0.07822 |
| O | -1.51752 | 0.8139 | -0.105 |
| C | -3.07049 | -2.62015 | -0.42776 |
| C | -5.0799 | 1.9854 | 0.32082 |
| O | -5.19111 | -0.85367 | 0.02414 |
| O | -2.30698 | 2.88894 | 0.24684 |
| C | -1.09293 | 3.39096 | -0.33994 |
| H | 2.3544 | -0.20037 | -1.15683 |
| H | 0.57285 | 0.11194 | 1.04405 |
| H | 1.43895 | -4.00196 | -0.00464 |
| H | -0.14843 | -0.45271 | -2.58267 |
| H | 0.84167 | -1.89093 | -2.26941 |
| H | -0.91154 | -2.04197 | -2.44098 |
| H | 0.30651 | -2.66009 | 2.60844 |
| H | -1.16982 | -1.8398 | 2.06997 |
| H | -0.87252 | -3.5184 | 1.6053 |
| H | 3.84885 | -3.79969 | -0.96744 |
| H | 3.91111 | -2.37066 | -2.01311 |
| H | 4.70347 | -2.33346 | -0.44114 |
| H | 4.47927 | -0.09543 | 2.49822 |
| H | 3.67794 | -1.68007 | 1.97804 |
| H | 4.18229 | 1.83771 | 1.36843 |
| H | 1.93047 | 2.08532 | 1.66929 |
| H | 4.91173 | 1.28546 | -0.97138 |
| H | 3.29296 | 1.69006 | -1.54356 |
| H | 4.80637 | 3.65219 | -1.82952 |
| H | 3.55036 | 4.02993 | -0.63414 |
| H | 5.1943 | 3.63937 | -0.10226 |
| H | -3.60385 | -3.03929 | 0.4324 |
| H | -3.71806 | -2.79024 | -1.29564 |
| H | -2.13475 | -3.16419 | -0.56881 |
| H | -4.8305 | 3.04284 | 0.20954 |
| H | -5.85624 | 1.71327 | -0.40165 |
| H | -5.51625 | 1.83405 | 1.3161 |
| H | -1.08456 | 4.45476 | -0.09991 |
| H | -0.20549 | 2.9044 | 0.07116 |
| H | -1.10893 | 3.25809 | -1.42761 |

**Conformer 1a6**

Calculation method: mPW1PW91

Basic Set: 6-31G(d)

E (mPW1PW91): -1156.95744112 a. u.

Boltzmann population: 6.208%

| **Conformer 1a6** | Coordinates (Angstroms) | | |
| --- | --- | --- | --- |
| Atom | X | Y | Z |
| C | 2.46513 | -2.28652 | -0.2921 |
| C | 2.25601 | -0.77013 | -0.29838 |
| C | 0.82349 | -0.61207 | 0.2509 |
| C | 0.29752 | -1.98506 | 0.61712 |
| C | 1.38948 | -2.91811 | 0.20252 |
| C | -0.35593 | -1.16555 | -0.53505 |
| C | -0.20829 | -1.5511 | -2.0048 |
| C | -1.68799 | -0.52903 | -0.26418 |
| C | -0.43594 | -2.2824 | 1.90979 |
| C | 3.29406 | 0.00137 | 0.516 |
| C | 3.7115 | -2.91151 | -0.84293 |
| C | 3.64973 | -0.37118 | 1.74932 |
| C | 3.8954 | 1.23159 | -0.15048 |
| O | 5.0528 | 1.71834 | 0.51917 |
| C | 2.90451 | 2.40427 | -0.21745 |
| C | 3.44245 | 3.60389 | -1.00307 |
| C | -2.89942 | -1.13 | -0.2348 |
| C | -4.12711 | -0.32678 | -0.03614 |
| C | -3.94483 | 1.10962 | 0.12638 |
| C | -2.68052 | 1.60381 | 0.07901 |
| O | -1.57904 | 0.84431 | -0.10839 |
| C | -3.10151 | -2.60964 | -0.40539 |
| C | -5.15782 | 1.97065 | 0.33502 |
| O | -5.24295 | -0.85958 | -0.00869 |
| O | -2.41346 | 2.91389 | 0.21684 |
| C | -1.05716 | 3.36464 | 0.1485 |
| H | 2.30896 | -0.40812 | -1.33789 |
| H | 0.6304 | 0.23165 | 0.9057 |
| H | 1.29794 | -3.99826 | 0.2931 |
| H | -0.21788 | -0.65115 | -2.63228 |
| H | 0.72083 | -2.09205 | -2.19529 |
| H | -1.03821 | -2.18726 | -2.32833 |
| H | 0.2792 | -2.36553 | 2.73701 |
| H | -1.1547 | -1.49809 | 2.1627 |
| H | -0.98703 | -3.22865 | 1.85067 |
| H | 3.69869 | -3.99982 | -0.72726 |
| H | 3.83137 | -2.68887 | -1.91271 |
| H | 4.6039 | -2.52303 | -0.33642 |
| H | 4.35967 | 0.21075 | 2.32895 |
| H | 3.23121 | -1.25877 | 2.21593 |
| H | 4.15664 | 0.95288 | -1.18923 |
| H | 5.68669 | 0.98538 | 0.57062 |
| H | 1.96945 | 2.04752 | -0.66774 |
| H | 2.67302 | 2.69683 | 0.81485 |
| H | 2.71412 | 4.42281 | -1.01623 |
| H | 4.36819 | 3.97575 | -0.55577 |
| H | 3.65597 | 3.33162 | -2.04429 |
| H | -2.16053 | -3.16219 | -0.43132 |
| H | -3.72428 | -2.9923 | 0.40982 |
| H | -3.65759 | -2.81196 | -1.32877 |
| H | -4.89078 | 3.01625 | 0.50049 |
| H | -5.82513 | 1.908 | -0.53272 |
| H | -5.73424 | 1.60684 | 1.19296 |
| H | -1.11296 | 4.44637 | 0.2752 |
| H | -0.4511 | 2.92762 | 0.94803 |
| H | -0.60719 | 3.12544 | -0.81986 |

**Conformer 1b1**

Calculation method: mPW1PW91

Basic Set: 6-31G(d)

E (mPW1PW91): -1156.95725137 a. u.

Boltzmann population: 20.538%

| **Conformer 1b1** | Coordinates (Angstroms) | | |
| --- | --- | --- | --- |
| Atom | X | Y | Z |
| C | 2.65603 | -2.00566 | -0.14012 |
| C | 2.33008 | -0.50992 | -0.18136 |
| C | 0.87922 | -0.45608 | 0.3347 |
| C | 0.45085 | -1.86087 | 0.71276 |
| C | 1.62242 | -2.71236 | 0.34173 |
| C | -0.23261 | -1.11431 | -0.46941 |
| C | -0.01736 | -1.51099 | -1.92804 |
| C | -1.6178 | -0.58114 | -0.24726 |
| C | -0.2903 | -2.194 | 1.99243 |
| C | 3.30387 | 0.33873 | 0.63805 |
| C | 3.96334 | -2.53642 | -0.64618 |
| C | 3.52974 | 0.10666 | 1.93577 |
| C | 4.04371 | 1.44399 | -0.09749 |
| O | 4.67766 | 0.92748 | -1.27666 |
| C | 3.1451 | 2.59346 | -0.57147 |
| C | 2.60049 | 3.45304 | 0.57216 |
| C | -2.77727 | -1.27716 | -0.21549 |
| C | -4.06998 | -0.57014 | -0.07126 |
| C | -4.00984 | 0.88338 | 0.01476 |
| C | -2.78836 | 1.47599 | -0.0245 |
| O | -1.62435 | 0.80178 | -0.14942 |
| C | -2.85816 | -2.77337 | -0.33255 |
| C | -5.29205 | 1.65464 | 0.14783 |
| O | -5.13832 | -1.19242 | -0.03639 |
| O | -2.63051 | 2.80739 | 0.0705 |
| C | -1.33639 | 3.37454 | -0.15876 |
| H | 2.3933 | -0.16509 | -1.22223 |
| H | 0.60247 | 0.38142 | 0.96759 |
| H | 1.61305 | -3.7947 | 0.45021 |
| H | -0.78404 | -2.21952 | -2.25778 |
| H | -0.08623 | -0.62547 | -2.57187 |
| H | 0.95795 | -1.9746 | -2.08739 |
| H | 0.40709 | -2.20721 | 2.83901 |
| H | -1.0751 | -1.46496 | 2.21242 |
| H | -0.76309 | -3.18193 | 1.93758 |
| H | 4.01196 | -3.62712 | -0.57005 |
| H | 4.12475 | -2.2615 | -1.69749 |
| H | 4.80456 | -2.12233 | -0.07365 |
| H | 4.2312 | 0.70892 | 2.5088 |
| H | 3.02218 | -0.6913 | 2.47222 |
| H | 4.80475 | 1.86308 | 0.581 |
| H | 5.27659 | 0.21724 | -0.99618 |
| H | 3.74743 | 3.20741 | -1.25186 |
| H | 2.32115 | 2.18524 | -1.17043 |
| H | 1.96734 | 4.25951 | 0.1859 |
| H | 2.00506 | 2.85938 | 1.27511 |
| H | 3.4137 | 3.91818 | 1.14253 |
| H | -3.40952 | -3.04994 | -1.23916 |
| H | -1.87579 | -3.24819 | -0.35741 |
| H | -3.43521 | -3.17982 | 0.50475 |
| H | -5.14107 | 2.72406 | -0.0143 |
| H | -6.0257 | 1.27611 | -0.57113 |
| H | -5.73145 | 1.51288 | 1.14326 |
| H | -1.48598 | 4.45303 | -0.09581 |
| H | -0.62065 | 3.05369 | 0.60357 |
| H | -0.95498 | 3.10753 | -1.14933 |

**Conformer 1b2**

Calculation method: mPW1PW91

Basic Set: 6-31G(d)

E (mPW1PW91): -1156.95752804 a. u.

Boltzmann population: 17.886%

| **Conformer 1b2** | Coordinates (Angstroms) | | |
| --- | --- | --- | --- |
| Atom | X | Y | Z |
| C | 2.45137 | -2.10407 | -0.15915 |
| C | 2.4325 | -0.57405 | -0.19495 |
| C | 1.01197 | -0.23232 | 0.29746 |
| C | 0.3059 | -1.5244 | 0.65987 |
| C | 1.29041 | -2.59165 | 0.30453 |
| C | -0.19587 | -0.65667 | -0.5265 |
| C | -0.07008 | -1.11565 | -1.9765 |
| C | -1.47197 | 0.09434 | -0.30444 |
| C | -0.51321 | -1.70505 | 1.92312 |
| C | 3.53727 | 0.06283 | 0.65004 |
| C | 3.63251 | -2.8836 | -0.65339 |
| C | 3.73671 | -0.27637 | 1.92829 |
| C | 4.43291 | 1.08031 | -0.03776 |
| O | 4.98157 | 0.52801 | -1.24392 |
| C | 3.71974 | 2.37384 | -0.45118 |
| C | 3.28473 | 3.24068 | 0.73304 |
| C | -1.69995 | 1.42687 | -0.2694 |
| C | -3.07639 | 1.93871 | -0.05293 |
| C | -4.13484 | 0.94367 | 0.05685 |
| C | -3.7902 | -0.36727 | -0.02848 |
| O | -2.52565 | -0.80435 | -0.19974 |
| C | -0.636 | 2.4743 | -0.45471 |
| C | -5.54955 | 1.41023 | 0.25145 |
| O | -3.30319 | 3.15273 | 0.0114 |
| O | -4.69353 | -1.35815 | 0.06665 |
| C | -4.27701 | -2.70703 | -0.1701 |
| H | 2.58369 | -0.24135 | -1.23075 |
| H | 0.89878 | 0.63565 | 0.93928 |
| H | 1.06359 | -3.65089 | 0.40521 |
| H | -0.97406 | -1.65338 | -2.28404 |
| H | 0.0458 | -0.25164 | -2.64169 |
| H | 0.78185 | -1.7818 | -2.12488 |
| H | 0.14763 | -1.90711 | 2.77495 |
| H | -1.10113 | -0.81375 | 2.16087 |
| H | -1.20834 | -2.54937 | 1.83463 |
| H | 3.46746 | -3.96231 | -0.56889 |
| H | 3.84785 | -2.65504 | -1.70632 |
| H | 4.53591 | -2.63431 | -0.08094 |
| H | 4.52653 | 0.17868 | 2.52176 |
| H | 3.11723 | -1.01747 | 2.42753 |
| H | 5.24869 | 1.34615 | 0.6541 |
| H | 5.47022 | -0.27362 | -0.99797 |
| H | 4.41683 | 2.92902 | -1.09045 |
| H | 2.85617 | 2.1256 | -1.0813 |
| H | 2.78658 | 4.15142 | 0.38454 |
| H | 2.58899 | 2.70816 | 1.39074 |
| H | 4.14603 | 3.54456 | 1.34063 |
| H | -0.35074 | 2.92396 | 0.50516 |
| H | 0.2616 | 2.08164 | -0.935 |
| H | -1.04719 | 3.28794 | -1.05898 |
| H | -6.26731 | 0.59988 | 0.10686 |
| H | -5.68948 | 1.82323 | 1.25828 |
| H | -5.76994 | 2.22192 | -0.4494 |
| H | -5.18446 | -3.30367 | -0.0706 |
| H | -3.86242 | -2.82422 | -1.17644 |
| H | -3.53578 | -3.02955 | 0.56694 |

**Conformer 1b3**

Calculation method: mPW1PW91

Basic Set: 6-31G(d)

E (mPW1PW91): -1156.95788654 a. u.

Boltzmann population: 14.571%

| **Conformer 1b3** | Coordinates (Angstroms) | | |
| --- | --- | --- | --- |
| Atom | X | Y | Z |
| C | 2.57884 | -2.15261 | -0.20222 |
| C | 2.31501 | -0.64706 | -0.1423 |
| C | 0.85729 | -0.56811 | 0.35542 |
| C | 0.37056 | -1.97577 | 0.63655 |
| C | 1.51151 | -2.84748 | 0.22075 |
| C | -0.26821 | -1.12612 | -0.5025 |
| C | -0.04845 | -1.43397 | -1.9818 |
| C | -1.63237 | -0.55002 | -0.25969 |
| C | -0.3997 | -2.36147 | 1.88382 |
| C | 3.28855 | 0.11605 | 0.75548 |
| C | 3.86376 | -2.70396 | -0.74354 |
| C | 3.65153 | -0.33396 | 1.96125 |
| C | 3.84993 | 1.42108 | 0.21501 |
| O | 4.48004 | 1.21029 | -1.05704 |
| C | 2.78863 | 2.50737 | 0.00224 |
| C | 3.37958 | 3.82947 | -0.49838 |
| C | -2.82072 | -1.19541 | -0.29119 |
| C | -4.08435 | -0.44475 | -0.11446 |
| C | -3.96326 | 0.99484 | 0.07834 |
| C | -2.7171 | 1.53448 | 0.09943 |
| O | -1.58118 | 0.82133 | -0.06173 |
| C | -2.96086 | -2.675 | -0.51524 |
| C | -5.21291 | 1.81032 | 0.25146 |
| O | -5.17884 | -1.02023 | -0.13976 |
| O | -2.50411 | 2.84679 | 0.29842 |
| C | -1.18671 | 3.37552 | 0.11453 |
| H | 2.41196 | -0.22602 | -1.15262 |
| H | 0.60369 | 0.23548 | 1.03975 |
| H | 1.45598 | -3.93348 | 0.25136 |
| H | -0.84228 | -2.08039 | -2.36958 |
| H | -0.06401 | -0.50457 | -2.56431 |
| H | 0.90626 | -1.93266 | -2.15951 |
| H | 0.28591 | -2.45398 | 2.73485 |
| H | -1.15981 | -1.61893 | 2.142 |
| H | -0.90818 | -3.32535 | 1.76095 |
| H | 3.88065 | -3.79759 | -0.70214 |
| H | 4.01514 | -2.40389 | -1.78995 |
| H | 4.72551 | -2.32826 | -0.17702 |
| H | 4.33954 | 0.22305 | 2.59365 |
| H | 3.27073 | -1.27038 | 2.36126 |
| H | 4.59032 | 1.80435 | 0.93635 |
| H | 5.19343 | 0.56652 | -0.92277 |
| H | 2.04236 | 2.13846 | -0.71276 |
| H | 2.27012 | 2.6608 | 0.95763 |
| H | 2.59543 | 4.5827 | -0.63581 |
| H | 4.1069 | 4.23279 | 0.21704 |
| H | 3.89152 | 3.68745 | -1.45386 |
| H | -3.49165 | -2.86545 | -1.4558 |
| H | -1.99835 | -3.18887 | -0.54224 |
| H | -3.58237 | -3.11102 | 0.2738 |
| H | -5.01331 | 2.88075 | 0.16734 |
| H | -5.95171 | 1.51599 | -0.50081 |
| H | -5.67231 | 1.61851 | 1.22933 |
| H | -1.29033 | 4.45047 | 0.26674 |
| H | -0.48615 | 2.96197 | 0.84589 |
| H | -0.81551 | 3.1755 | -0.89556 |

**Conformer 1b4**

Calculation method: mPW1PW91

Basic Set: 6-31G(d)

E (mPW1PW91): -1156.95723580 a. u.

Boltzmann population: 4.278%

| **Conformer 1b4** | Coordinates (Angstroms) | | |
| --- | --- | --- | --- |
| Atom | X | Y | Z |
| C | 2.35328 | -2.40269 | -0.40517 |
| C | 2.17735 | -0.88344 | -0.40918 |
| C | 0.77142 | -0.6955 | 0.18932 |
| C | 0.23708 | -2.04868 | 0.60736 |
| C | 1.29066 | -3.00735 | 0.14982 |
| C | -0.44942 | -1.24201 | -0.53374 |
| C | -0.38986 | -1.67262 | -1.99712 |
| C | -1.73168 | -0.52288 | -0.23066 |
| C | -0.43518 | -2.31279 | 1.93988 |
| C | 3.24741 | -0.10792 | 0.36222 |
| C | 3.5385 | -3.06578 | -1.04307 |
| C | 3.88937 | -0.64462 | 1.40587 |
| C | 3.46025 | 1.33548 | -0.08029 |
| O | 2.32073 | 2.13598 | 0.30904 |
| C | 4.76719 | 1.99548 | 0.37688 |
| C | 4.95482 | 3.39659 | -0.21292 |
| C | -2.96873 | -1.04016 | -0.05359 |
| C | -4.12737 | -0.14172 | 0.15495 |
| C | -3.85487 | 1.29139 | 0.12773 |
| C | -2.5714 | 1.69594 | -0.05822 |
| O | -1.53609 | 0.84651 | -0.23353 |
| C | -3.26804 | -2.51265 | -0.06873 |
| C | -4.99745 | 2.24909 | 0.31013 |
| O | -5.26498 | -0.59671 | 0.32508 |
| O | -2.20254 | 2.9865 | -0.0522 |
| C | -0.97308 | 3.35403 | -0.70296 |
| H | 2.19656 | -0.51913 | -1.449 |
| H | 0.61082 | 0.17472 | 0.81405 |
| H | 1.17663 | -4.08537 | 0.24303 |
| H | -1.24676 | -2.30603 | -2.25094 |
| H | -0.42513 | -0.79127 | -2.64934 |
| H | 0.51951 | -2.23182 | -2.22579 |
| H | 0.31662 | -2.36549 | 2.73672 |
| H | -1.14946 | -1.52628 | 2.19886 |
| H | -0.97946 | -3.26483 | 1.93376 |
| H | 3.51334 | -4.15111 | -0.90228 |
| H | 3.56664 | -2.86917 | -2.12435 |
| H | 4.48092 | -2.68596 | -0.63073 |
| H | 4.63999 | -0.10142 | 1.97171 |
| H | 3.68288 | -1.66097 | 1.73049 |
| H | 3.42763 | 1.36798 | -1.17706 |
| H | 2.26423 | 2.07742 | 1.27741 |
| H | 4.77988 | 2.05622 | 1.47446 |
| H | 5.60753 | 1.34961 | 0.09251 |
| H | 5.87254 | 3.86127 | 0.16338 |
| H | 5.02417 | 3.35839 | -1.30698 |
| H | 4.11 | 4.04212 | 0.04494 |
| H | -3.97156 | -2.74236 | -0.87747 |
| H | -2.37019 | -3.12069 | -0.19474 |
| H | -3.77201 | -2.80298 | 0.85947 |
| H | -4.71549 | 3.26924 | 0.04021 |
| H | -5.84756 | 1.92891 | -0.30071 |
| H | -5.34565 | 2.25001 | 1.3508 |
| H | -0.91554 | 4.43811 | -0.59866 |
| H | -0.10453 | 2.88312 | -0.23704 |
| H | -1.00504 | 3.08628 | -1.76495 |

**Conformer 1b4**

Calculation method: mPW1PW91

Basic Set: 6-31G(d)

E (mPW1PW91): -1156.95729865 a. u.

Boltzmann population: 4.137%

| **Conformer 1b5** | Coordinates (Angstroms) | | |
| --- | --- | --- | --- |
| Atom | X | Y | Z |
| C | -2.16577 | -2.37335 | 0.31352 |
| C | -2.26564 | -0.84725 | 0.34006 |
| C | -0.89772 | -0.39897 | -0.21045 |
| C | -0.10057 | -1.63163 | -0.58252 |
| C | -0.98337 | -2.77045 | -0.1833 |
| C | 0.36571 | -0.70393 | 0.5758 |
| C | 0.32318 | -1.14639 | 2.03563 |
| C | 1.55954 | 0.15601 | 0.3029 |
| C | 0.68795 | -1.76038 | -1.87113 |
| C | -3.43585 | -0.27213 | -0.46033 |
| C | -3.256 | -3.24483 | 0.86214 |
| C | -3.77203 | -0.76577 | -1.65663 |
| C | -4.12818 | 0.91558 | 0.19246 |
| O | -3.15139 | 1.9202 | 0.52637 |
| C | -5.30581 | 1.52244 | -0.57867 |
| C | -5.98363 | 2.66545 | 0.18332 |
| C | 1.66387 | 1.50324 | 0.24013 |
| C | 2.98233 | 2.13109 | -0.02597 |
| C | 4.12347 | 1.23472 | -0.15169 |
| C | 3.90321 | -0.0999 | -0.02999 |
| O | 2.68816 | -0.64557 | 0.18246 |
| C | 0.52188 | 2.46223 | 0.44111 |
| C | 5.48195 | 1.82685 | -0.39744 |
| O | 3.09616 | 3.35897 | -0.12063 |
| O | 4.89132 | -1.00675 | -0.12402 |
| C | 4.59286 | -2.38967 | 0.0917 |
| H | -2.37958 | -0.50575 | 1.37906 |
| H | -0.88039 | 0.46372 | -0.86789 |
| H | -0.67348 | -3.80918 | -0.27758 |
| H | 1.2855 | -1.58055 | 2.32944 |
| H | 0.13168 | -0.28624 | 2.68816 |
| H | -0.44995 | -1.89606 | 2.2153 |
| H | 0.01787 | -2.02751 | -2.69739 |
| H | 1.19086 | -0.82617 | -2.13774 |
| H | 1.45401 | -2.54252 | -1.79798 |
| H | -3.02517 | -4.30669 | 0.72973 |
| H | -3.40646 | -3.06523 | 1.93609 |
| H | -4.21442 | -3.03682 | 0.37069 |
| H | -4.58093 | -0.35637 | -2.253 |
| H | -3.2408 | -1.61489 | -2.07966 |
| H | -4.49531 | 0.58927 | 1.1763 |
| H | -2.7772 | 2.24266 | -0.31036 |
| H | -4.94818 | 1.88486 | -1.55357 |
| H | -6.03649 | 0.73185 | -0.79129 |
| H | -6.79641 | 3.10174 | -0.40728 |
| H | -6.41072 | 2.30994 | 1.12895 |
| H | -5.2652 | 3.45517 | 0.42093 |
| H | 0.28053 | 2.97367 | -0.50015 |
| H | -0.38009 | 1.9897 | 0.83137 |
| H | 0.84145 | 3.25104 | 1.12941 |
| H | 6.26362 | 1.06449 | -0.39468 |
| H | 5.50343 | 2.35151 | -1.36008 |
| H | 5.70886 | 2.57785 | 0.36742 |
| H | 4.18604 | -2.55725 | 1.09389 |
| H | 3.8854 | -2.76493 | -0.65379 |
| H | 5.54917 | -2.90372 | -0.01166 |

**Figure S15f**. The Cartesian Coordinates of the dominant conformers for **11a** and **11b**

**Conformer 11a1**

Calculation method: mPW1PW91

Basic Set: 6-31G(d)

E (mPW1PW91): -1232.07195113 a. u.

Boltzmann population: 16.810%

| **Conformer 11a1** | Coordinates (Angstroms) | | |
| --- | --- | --- | --- |
| Atom | X | Y | Z |
| C | -3.46688 | 0.00712 | 0.17118 |
| C | -2.48747 | 1.09904 | -0.20787 |
| C | -1.20041 | 1.09949 | 0.19196 |
| C | -0.62331 | -0.00811 | 1.09373 |
| C | -1.64425 | -1.06867 | 1.44061 |
| C | -2.93222 | -1.06017 | 1.09057 |
| C | -3.09713 | 2.1874 | -1.0608 |
| C | -3.89495 | -2.11908 | 1.56351 |
| C | 0.58851 | -0.67817 | 0.41547 |
| C | -0.28466 | 2.24921 | -0.15126 |
| C | -0.49308 | 3.51507 | 0.65714 |
| C | 0.61001 | 2.12374 | -1.14687 |
| C | 1.61481 | 3.12657 | -1.65041 |
| C | 3.0516 | 2.57555 | -1.61862 |
| C | -0.20201 | 0.6172 | 2.45653 |
| C | 0.60282 | -1.55071 | -0.62164 |
| C | 1.8985 | -2.10372 | -1.10072 |
| C | 3.10357 | -1.63992 | -0.42748 |
| C | 2.96231 | -0.74551 | 0.58241 |
| O | 1.77164 | -0.27967 | 1.00963 |
| O | 4.01193 | -0.2302 | 1.24676 |
| C | 3.77897 | 0.5733 | 2.40793 |
| O | 1.9481 | -2.91803 | -2.03004 |
| C | 4.43404 | -2.16591 | -0.88457 |
| C | -0.61741 | -2.03033 | -1.36252 |
| O | -3.88878 | -0.57644 | -1.09579 |
| O | -5.2976 | -0.94353 | -0.97448 |
| H | -4.36993 | 0.46774 | 0.59729 |
| H | -1.26962 | -1.86425 | 2.08497 |
| H | -2.33727 | 2.82978 | -1.50935 |
| H | -3.6939 | 1.74323 | -1.86291 |
| H | -3.77684 | 2.81585 | -0.46711 |
| H | -3.41536 | -2.79731 | 2.27632 |
| H | -4.77276 | -1.67204 | 2.04747 |
| H | -4.27426 | -2.72064 | 0.72915 |
| H | 0.22354 | 4.29926 | 0.40111 |
| H | -1.50014 | 3.91772 | 0.4901 |
| H | -0.41312 | 3.32455 | 1.73338 |
| H | 0.63423 | 1.17222 | -1.67783 |
| H | 1.36383 | 3.38328 | -2.69071 |
| H | 1.56835 | 4.06355 | -1.08596 |
| H | 3.75944 | 3.30107 | -2.03528 |
| H | 3.36503 | 2.34464 | -0.59409 |
| H | 3.13382 | 1.65192 | -2.20294 |
| H | 0.18709 | -0.15297 | 3.13083 |
| H | 0.56672 | 1.38215 | 2.33747 |
| H | -1.0832 | 1.06676 | 2.92294 |
| H | 4.77244 | 0.81772 | 2.78578 |
| H | 3.24156 | 1.49185 | 2.15435 |
| H | 3.21752 | 0.02079 | 3.16802 |
| H | 5.25171 | -1.79774 | -0.26162 |
| H | 4.43095 | -3.26132 | -0.86603 |
| H | 4.62376 | -1.87661 | -1.92501 |
| H | -1.07959 | -2.8865 | -0.85615 |
| H | -1.38092 | -1.25998 | -1.47265 |
| H | -0.29581 | -2.37635 | -2.34777 |
| H | -5.24781 | -1.8659 | -1.28224 |

**Conformer 11a2**

Calculation method: mPW1PW91

Basic Set: 6-31G(d)

E (mPW1PW91): -1232.07234891 a. u.

Boltzmann population: 11.279%

| **Conformer 11a2** | Coordinates (Angstroms) | | |
| --- | --- | --- | --- |
| Atom | X | Y | Z |
| C | -3.23937 | -0.72128 | 0.41783 |
| C | -2.47485 | 0.44086 | -0.17908 |
| C | -1.1714 | 0.67108 | 0.07712 |
| C | -0.36385 | -0.21557 | 1.04592 |
| C | -1.15554 | -1.40242 | 1.54943 |
| C | -2.44987 | -1.63052 | 1.32217 |
| C | -3.2988 | 1.30617 | -1.10592 |
| C | -3.19915 | -2.78604 | 1.93137 |
| C | 0.91861 | -0.72792 | 0.3648 |
| C | -0.44961 | 1.83572 | -0.55773 |
| C | 0.16184 | 1.57566 | -1.91908 |
| C | -0.42613 | 3.02602 | 0.06863 |
| C | 0.20278 | 4.3198 | -0.3807 |
| C | -0.83379 | 5.44298 | -0.563 |
| C | -0.01358 | 0.61079 | 2.31906 |
| C | 1.04611 | -1.69603 | -0.57491 |
| C | 2.39733 | -2.05265 | -1.08576 |
| C | 3.52196 | -1.28848 | -0.56326 |
| C | 3.26586 | -0.33115 | 0.36351 |
| O | 2.03097 | -0.04796 | 0.82644 |
| O | 4.23356 | 0.41902 | 0.91762 |
| C | 3.87103 | 1.48649 | 1.79973 |
| O | 2.5541 | -2.94585 | -1.92607 |
| C | 4.90205 | -1.59988 | -1.06708 |
| C | -0.09935 | -2.46541 | -1.17995 |
| O | -3.75783 | -1.57598 | -0.64756 |
| O | -5.14722 | -1.22143 | -0.89809 |
| H | -4.12758 | -0.35185 | 0.94895 |
| H | -0.60182 | -2.07044 | 2.20868 |
| H | -2.80374 | 2.25286 | -1.3275 |
| H | -3.48158 | 0.79338 | -2.06047 |
| H | -4.28055 | 1.52281 | -0.66795 |
| H | -2.54798 | -3.38886 | 2.57182 |
| H | -4.04213 | -2.42933 | 2.53943 |
| H | -3.62187 | -3.42926 | 1.15268 |
| H | 0.9761 | 0.84476 | -1.86495 |
| H | -0.58996 | 1.14964 | -2.59519 |
| H | 0.55494 | 2.48362 | -2.38285 |
| H | -0.948 | 3.09666 | 1.02431 |
| H | 0.93222 | 4.63972 | 0.37897 |
| H | 0.76868 | 4.18956 | -1.3084 |
| H | -0.34697 | 6.38798 | -0.82943 |
| H | -1.54766 | 5.1929 | -1.35582 |
| H | -1.40482 | 5.60796 | 0.35845 |
| H | 0.55871 | 0.0029 | 3.02766 |
| H | 0.56722 | 1.50236 | 2.08054 |
| H | -0.9463 | 0.91472 | 2.80281 |
| H | 4.8151 | 1.95879 | 2.0736 |
| H | 3.22193 | 2.21178 | 1.29895 |
| H | 3.37003 | 1.10855 | 2.69586 |
| H | 5.64233 | -0.89314 | -0.6866 |
| H | 5.19883 | -2.6138 | -0.7729 |
| H | 4.91297 | -1.58191 | -2.16225 |
| H | -0.36452 | -3.336 | -0.56837 |
| H | -1.00005 | -1.86245 | -1.30293 |
| H | 0.22844 | -2.84507 | -2.1507 |
| H | -5.07425 | -0.87052 | -1.80373 |

**Conformer 11a3**

Calculation method: mPW1PW91

Basic Set: 6-31G(d)

E (mPW1PW91): -1232.07229589 a. u.

Boltzmann population: 10.821%

| **Conformer 11a3** | Coordinates (Angstroms) | | |
| --- | --- | --- | --- |
| Atom | X | Y | Z |
| C | -3.28155 | -0.45687 | 0.25982 |
| C | -2.41603 | 0.66284 | -0.28114 |
| C | -1.11187 | 0.79607 | 0.03206 |
| C | -0.41154 | -0.14785 | 1.03046 |
| C | -1.32281 | -1.2443 | 1.53473 |
| C | -2.62193 | -1.3721 | 1.2581 |
| C | -3.1625 | 1.60033 | -1.20142 |
| C | -3.47265 | -2.44091 | 1.8943 |
| C | 0.83971 | -0.77781 | 0.39275 |
| C | -0.28759 | 1.91635 | -0.55442 |
| C | 0.41357 | 1.60488 | -1.86042 |
| C | -0.25776 | 3.11255 | 0.05934 |
| C | 0.46475 | 4.36974 | -0.35098 |
| C | -0.50115 | 5.53036 | -0.65009 |
| C | -0.02019 | 0.66469 | 2.30202 |
| C | 0.91954 | -1.80303 | -0.4899 |
| C | 2.2526 | -2.25945 | -0.96867 |
| C | 3.41326 | -1.51903 | -0.49289 |
| C | 3.2048 | -0.49843 | 0.37656 |
| O | 1.98524 | -0.13118 | 0.82098 |
| O | 4.20757 | 0.23311 | 0.89128 |
| C | 3.89559 | 1.39952 | 1.66096 |
| O | 2.36646 | -3.21125 | -1.74958 |
| C | 4.77605 | -1.92476 | -0.97626 |
| C | -0.26209 | -2.54371 | -1.05992 |
| O | -3.69251 | -1.20201 | -0.92478 |
| O | -5.0452 | -1.70358 | -0.69739 |
| H | -4.20566 | -0.03104 | 0.676 |
| H | -0.85485 | -1.93117 | 2.23999 |
| H | -2.5179 | 2.37551 | -1.61716 |
| H | -3.62786 | 1.04086 | -2.01887 |
| H | -3.97727 | 2.09784 | -0.65622 |
| H | -2.90741 | -2.99868 | 2.64775 |
| H | -4.35957 | -2.00961 | 2.37601 |
| H | -3.84243 | -3.15738 | 1.15164 |
| H | 1.14911 | 0.80105 | -1.74749 |
| H | -0.31465 | 1.25247 | -2.60192 |
| H | 0.92712 | 2.47385 | -2.27921 |
| H | -0.84964 | 3.21899 | 0.96992 |
| H | 1.13299 | 4.67584 | 0.46832 |
| H | 1.10824 | 4.1962 | -1.21921 |
| H | 0.04882 | 6.44819 | -0.88715 |
| H | -1.14888 | 5.29209 | -1.50097 |
| H | -1.14736 | 5.73954 | 0.2108 |
| H | 0.48845 | 0.02106 | 3.02759 |
| H | 0.63337 | 1.50462 | 2.06562 |
| H | -0.93416 | 1.04819 | 2.76471 |
| H | 3.37027 | 1.13726 | 2.58405 |
| H | 4.86118 | 1.84761 | 1.89809 |
| H | 3.28771 | 2.10385 | 1.08429 |
| H | 5.54881 | -1.22952 | -0.64193 |
| H | 5.02748 | -2.93008 | -0.61748 |
| H | 4.78402 | -1.97753 | -2.07031 |
| H | -1.11659 | -1.89513 | -1.25739 |
| H | 0.06152 | -3.02293 | -1.98699 |
| H | -0.59853 | -3.34028 | -0.38528 |
| H | -4.90029 | -2.64673 | -0.89103 |

**Conformer 11a4**

Calculation method: mPW1PW91

Basic Set: 6-31G(d)

E (mPW1PW91): -1232.07162536 a. u.

Boltzmann population: 10.456%

| **Conformer 11a4** | Coordinates (Angstroms) | | |
| --- | --- | --- | --- |
| Atom | X | Y | Z |
| C | -3.36909 | -0.41427 | 0.27988 |
| C | -2.47966 | 0.80186 | 0.13257 |
| C | -1.18939 | 0.81949 | 0.52338 |
| C | -0.5092 | -0.41525 | 1.14609 |
| C | -1.4311 | -1.6119 | 1.22918 |
| C | -2.72164 | -1.62862 | 0.89226 |
| C | -3.16539 | 1.9883 | -0.50405 |
| C | -3.60385 | -2.83416 | 1.0818 |
| C | 0.735 | -0.80466 | 0.32323 |
| C | -0.36747 | 2.08326 | 0.43697 |
| C | -0.61976 | 3.10044 | 1.53398 |
| C | 0.49286 | 2.26473 | -0.58004 |
| C | 1.39521 | 3.43823 | -0.85918 |
| C | 1.07065 | 4.11739 | -2.20182 |
| C | -0.11098 | -0.09747 | 2.61705 |
| C | 0.79005 | -1.37986 | -0.90315 |
| C | 2.11311 | -1.68592 | -1.51275 |
| C | 3.29446 | -1.31454 | -0.746 |
| C | 3.10972 | -0.74163 | 0.46933 |
| O | 1.89701 | -0.49204 | 1.00304 |
| O | 4.13241 | -0.38357 | 1.26534 |
| C | 3.86269 | 0.37357 | 2.44933 |
| O | 2.20486 | -2.21741 | -2.62545 |
| C | 4.65051 | -1.58809 | -1.33066 |
| C | -0.41004 | -1.74038 | -1.73996 |
| O | -3.86234 | -0.84454 | -1.02534 |
| O | -5.21461 | -0.32938 | -1.20144 |
| H | -4.26858 | -0.14724 | 0.8521 |
| H | -0.97949 | -2.50352 | 1.66371 |
| H | -2.52076 | 2.86685 | -0.54783 |
| H | -3.4539 | 1.75019 | -1.53569 |
| H | -4.08883 | 2.24877 | 0.0291 |
| H | -3.04885 | -3.66842 | 1.52177 |
| H | -4.45024 | -2.60068 | 1.74268 |
| H | -4.02964 | -3.15831 | 0.12648 |
| H | -0.04259 | 4.0182 | 1.39715 |
| H | -1.68157 | 3.37439 | 1.56743 |
| H | -0.37756 | 2.698 | 2.52437 |
| H | 0.55641 | 1.47365 | -1.32685 |
| H | 1.35567 | 4.17967 | -0.05455 |
| H | 2.43439 | 3.07794 | -0.89398 |
| H | 1.77785 | 4.92755 | -2.41254 |
| H | 1.12477 | 3.40088 | -3.02971 |
| H | 0.05991 | 4.54066 | -2.19385 |
| H | 0.33979 | -0.97443 | 3.09342 |
| H | 0.60322 | 0.72481 | 2.6811 |
| H | -1.01325 | 0.16951 | 3.17449 |
| H | 3.25959 | -0.19959 | 3.15971 |
| H | 4.8425 | 0.58338 | 2.87995 |
| H | 3.35123 | 1.31199 | 2.21187 |
| H | 5.4489 | -1.15899 | -0.72168 |
| H | 4.81991 | -2.66774 | -1.42261 |
| H | 4.70859 | -1.1802 | -2.34554 |
| H | -0.07091 | -1.86904 | -2.77045 |
| H | -0.85332 | -2.69016 | -1.41936 |
| H | -1.1967 | -0.98463 | -1.70191 |
| H | -5.08527 | 0.20879 | -2.00272 |

**Conformer 11a5**

Calculation method: mPW1PW91

Basic Set: 6-31G(d)

E (mPW1PW91): -1232.07215989 a. u.

Boltzmann population: 10.253%

| **Conformer 11a5** | Coordinates (Angstroms) | | |
| --- | --- | --- | --- |
| Atom | X | Y | Z |
| C | -3.37824 | -0.22665 | 0.43161 |
| C | -2.48636 | 0.77333 | -0.27321 |
| C | -1.15982 | 0.86078 | -0.04785 |
| C | -0.45218 | -0.02726 | 0.99528 |
| C | -1.37693 | -1.05413 | 1.61214 |
| C | -2.69274 | -1.14052 | 1.41227 |
| C | -3.21451 | 1.64519 | -1.27121 |
| C | -3.5688 | -2.13328 | 2.12953 |
| C | 0.74403 | -0.76388 | 0.36427 |
| C | -0.31475 | 1.8671 | -0.79243 |
| C | 0.24122 | 1.40994 | -2.12469 |
| C | -0.15732 | 3.10519 | -0.29012 |
| C | 0.60456 | 4.27206 | -0.86423 |
| C | 1.68689 | 4.79843 | 0.09422 |
| C | 0.01916 | 0.86556 | 2.18137 |
| C | 0.73061 | -1.81549 | -0.49057 |
| C | 2.01565 | -2.40213 | -0.95757 |
| C | 3.23898 | -1.76833 | -0.48516 |
| C | 3.12394 | -0.71177 | 0.35827 |
| O | 1.94321 | -0.21382 | 0.77972 |
| O | 4.19087 | -0.06819 | 0.86201 |
| C | 3.98886 | 1.09308 | 1.6735 |
| O | 2.04056 | -3.37424 | -1.72105 |
| C | 4.55996 | -2.31628 | -0.94393 |
| C | -0.51463 | -2.46426 | -1.0366 |
| O | -4.01478 | -1.10298 | -0.54874 |
| O | -5.35721 | -0.60754 | -0.81714 |
| H | -4.20589 | 0.29669 | 0.93038 |
| H | -0.89887 | -1.72219 | 2.32814 |
| H | -2.60769 | 2.49237 | -1.59442 |
| H | -3.48748 | 1.06925 | -2.16615 |
| H | -4.14852 | 2.03023 | -0.84451 |
| H | -2.98561 | -2.75642 | 2.81456 |
| H | -4.34668 | -1.62058 | 2.7124 |
| H | -4.08603 | -2.78271 | 1.41573 |
| H | 0.89052 | 0.53554 | -2.01208 |
| H | -0.57607 | 1.10298 | -2.79023 |
| H | 0.81108 | 2.19144 | -2.63267 |
| H | -0.64722 | 3.32335 | 0.66032 |
| H | 1.06206 | 4.01948 | -1.82584 |
| H | -0.10506 | 5.08848 | -1.0676 |
| H | 2.17835 | 5.68736 | -0.31702 |
| H | 1.25799 | 5.07209 | 1.06568 |
| H | 2.45736 | 4.0389 | 0.27204 |
| H | 0.52187 | 0.26095 | 2.94338 |
| H | 0.70278 | 1.64994 | 1.85478 |
| H | -0.8603 | 1.32984 | 2.63677 |
| H | 4.9919 | 1.44042 | 1.9237 |
| H | 3.45029 | 1.87192 | 1.12417 |
| H | 3.44002 | 0.8492 | 2.58801 |
| H | 5.39238 | -1.6797 | -0.63699 |
| H | 4.72089 | -3.32362 | -0.54101 |
| H | 4.56237 | -2.41684 | -2.03449 |
| H | -0.88849 | -3.24241 | -0.3604 |
| H | -1.32642 | -1.75531 | -1.204 |
| H | -0.25217 | -2.95677 | -1.97604 |
| H | -5.264 | -0.35978 | -1.75441 |

**Conformer 11a6**

Calculation method: mPW1PW91

Basic Set: 6-31G(d)

E (mPW1PW91): -1232.07215472 a. u.

Boltzmann population: 9.809%

| **Conformer 11a6** | Coordinates (Angstroms) | | |
| --- | --- | --- | --- |
| Atom | X | Y | Z |
| C | -3.38489 | 0.01515 | 0.27996 |
| C | -2.40795 | 0.96461 | -0.38269 |
| C | -1.08819 | 0.96701 | -0.10774 |
| C | -0.47609 | 0.04106 | 0.96239 |
| C | -1.49297 | -0.89739 | 1.57321 |
| C | -2.80501 | -0.8962 | 1.33027 |
| C | -3.06397 | 1.89597 | -1.37585 |
| C | -3.75464 | -1.80729 | 2.06462 |
| C | 0.67917 | -0.79069 | 0.3741 |
| C | -0.15894 | 1.92368 | -0.81492 |
| C | 0.45321 | 1.41426 | -2.10278 |
| C | 0.0211 | 3.16375 | -0.32644 |
| C | 0.85927 | 4.28771 | -0.87914 |
| C | 1.89701 | 4.80458 | 0.13235 |
| C | 0.03187 | 0.91613 | 2.14743 |
| C | 0.61466 | -1.87231 | -0.43985 |
| C | 1.86899 | -2.54685 | -0.87056 |
| C | 3.12223 | -1.95748 | -0.41949 |
| C | 3.05903 | -0.86474 | 0.38227 |
| O | 1.90432 | -0.29069 | 0.77791 |
| O | 4.15599 | -0.25959 | 0.86852 |
| C | 4.01264 | 0.95461 | 1.61272 |
| O | 1.84508 | -3.55204 | -1.59007 |
| C | 4.41537 | -2.58902 | -0.84925 |
| C | -0.65849 | -2.47328 | -0.97545 |
| O | -3.94626 | -0.76079 | -0.81956 |
| O | -5.34426 | -1.04262 | -0.50664 |
| H | -4.22498 | 0.5924 | 0.69178 |
| H | -1.08728 | -1.57563 | 2.32382 |
| H | -2.35548 | 2.5988 | -1.81551 |
| H | -3.54868 | 1.32525 | -2.17437 |
| H | -3.85572 | 2.47751 | -0.88303 |
| H | -3.23761 | -2.36262 | 2.85361 |
| H | -4.57701 | -1.24121 | 2.52029 |
| H | -4.21748 | -2.53666 | 1.3894 |
| H | 1.0654 | 0.52197 | -1.93473 |
| H | -0.3376 | 1.1173 | -2.80367 |
| H | 1.07636 | 2.16351 | -2.59656 |
| H | -0.51292 | 3.42069 | 0.58999 |
| H | 1.36708 | 3.99333 | -1.8029 |
| H | 0.19315 | 5.12156 | -1.1484 |
| H | 2.44356 | 5.66507 | -0.26974 |
| H | 1.41779 | 5.11925 | 1.06719 |
| H | 2.62831 | 4.02587 | 0.37928 |
| H | 0.47397 | 0.28862 | 2.92846 |
| H | 0.77505 | 1.64656 | 1.8264 |
| H | -0.82246 | 1.44746 | 2.57666 |
| H | 3.44606 | 0.79161 | 2.53427 |
| H | 5.03157 | 1.26085 | 1.85213 |
| H | 3.5201 | 1.72917 | 1.01618 |
| H | 5.27455 | -1.96046 | -0.60568 |
| H | 4.5496 | -3.56575 | -0.36805 |
| H | 4.39467 | -2.77652 | -1.92777 |
| H | -1.43143 | -1.73053 | -1.17474 |
| H | -0.41407 | -3.01003 | -1.8954 |
| H | -1.07517 | -3.20974 | -0.27748 |
| H | -5.33869 | -2.01139 | -0.60382 |

**Conformer 11b1**

Calculation method: mPW1PW91

Basic Set: 6-31G(d)

E (mPW1PW91): -1232.07191431 a. u.

Boltzmann population: 14.617%

| **Conformer 11b1** | Coordinates (Angstroms) | | |
| --- | --- | --- | --- |
| Atom | X | Y | Z |
| C | -3.53513 | -0.07933 | -0.37118 |
| C | -2.55378 | 1.05491 | -0.15089 |
| C | -1.28004 | 0.81663 | 0.21888 |
| C | -0.78241 | -0.63752 | 0.42296 |
| C | -1.63597 | -1.63152 | -0.34475 |
| C | -2.89845 | -1.40401 | -0.71545 |
| C | -3.16973 | 2.42427 | -0.29746 |
| C | -3.72227 | -2.41096 | -1.47545 |
| C | 0.67358 | -0.77718 | -0.03955 |
| C | -0.31714 | 1.94143 | 0.48509 |
| C | -0.06218 | 2.2921 | 1.93815 |
| C | 0.25064 | 2.57933 | -0.55282 |
| C | 1.25527 | 3.70085 | -0.5353 |
| C | 2.62291 | 3.26359 | -1.09334 |
| C | -0.93975 | -1.0282 | 1.92761 |
| C | 1.14964 | -0.81015 | -1.30591 |
| C | 2.60476 | -0.96646 | -1.54784 |
| C | 3.45857 | -1.06388 | -0.37123 |
| C | 2.86773 | -1.01069 | 0.84931 |
| O | 1.53832 | -0.86859 | 1.03556 |
| O | 3.56578 | -1.11293 | 1.99375 |
| C | 2.88689 | -0.9898 | 3.24662 |
| O | 3.06394 | -1.00789 | -2.69521 |
| C | 4.93911 | -1.22747 | -0.56477 |
| C | 0.30227 | -0.67461 | -2.54262 |
| O | -4.25667 | -0.1289 | 0.89583 |
| O | -5.50102 | -0.86605 | 0.68862 |
| H | -4.27037 | 0.20611 | -1.13479 |
| H | -1.17017 | -2.5941 | -0.55031 |
| H | -2.48714 | 3.22222 | -0.00203 |
| H | -3.47054 | 2.60577 | -1.33888 |
| H | -4.07967 | 2.48824 | 0.31126 |
| H | -3.16548 | -3.33779 | -1.64694 |
| H | -4.65249 | -2.64888 | -0.94797 |
| H | -4.02037 | -2.00884 | -2.45376 |
| H | 0.60138 | 1.56275 | 2.42018 |
| H | 0.40346 | 3.27464 | 2.04786 |
| H | -1.00011 | 2.29591 | 2.5056 |
| H | -0.01377 | 2.23487 | -1.55319 |
| H | 0.87231 | 4.52639 | -1.15329 |
| H | 1.38776 | 4.10685 | 0.47309 |
| H | 3.32363 | 4.10613 | -1.11294 |
| H | 3.06034 | 2.46731 | -0.48151 |
| H | 2.53071 | 2.87863 | -2.11529 |
| H | -0.63307 | -2.0675 | 2.08902 |
| H | -0.3485 | -0.39387 | 2.58927 |
| H | -1.99479 | -0.92868 | 2.19149 |
| H | 3.66321 | -1.11848 | 4.00168 |
| H | 2.42669 | -0.00215 | 3.35446 |
| H | 2.12132 | -1.76249 | 3.36357 |
| H | 5.48106 | -1.173 | 0.38154 |
| H | 5.16072 | -2.18935 | -1.04277 |
| H | 5.31435 | -0.45335 | -1.24262 |
| H | 0.86232 | -0.09635 | -3.28315 |
| H | 0.11265 | -1.65394 | -2.99975 |
| H | -0.65796 | -0.19681 | -2.34778 |
| H | -5.32733 | -1.64329 | 1.24922 |

**Conformer 11b2**

Calculation method: mPW1PW91

Basic Set: 6-31G(d)

E (mPW1PW91): -1232.07219205 a. u.

Boltzmann population: 14.414%

| **Conformer 11b2** | Coordinates (Angstroms) | | |
| --- | --- | --- | --- |
| Atom | X | Y | Z |
| C | -3.38358 | -0.54459 | -0.22109 |
| C | -2.51127 | 0.66381 | -0.48042 |
| C | -1.27534 | 0.80472 | 0.03979 |
| C | -0.63205 | -0.28757 | 0.91525 |
| C | -1.51916 | -1.5018 | 1.08001 |
| C | -2.76396 | -1.62982 | 0.61721 |
| C | -3.1569 | 1.71616 | -1.35431 |
| C | -3.60789 | -2.85314 | 0.85969 |
| C | 0.70459 | -0.74339 | 0.29575 |
| C | -0.4944 | 2.08234 | -0.147 |
| C | -0.9499 | 3.2463 | 0.71162 |
| C | 0.49892 | 2.15074 | -1.05073 |
| C | 1.39205 | 3.31533 | -1.38957 |
| C | 2.879 | 2.99633 | -1.15146 |
| C | -0.40751 | 0.27242 | 2.34992 |
| C | 0.90543 | -1.48675 | -0.81993 |
| C | 2.29162 | -1.83168 | -1.23652 |
| C | 3.374 | -1.32025 | -0.40785 |
| C | 3.04711 | -0.57986 | 0.68124 |
| O | 1.77888 | -0.29651 | 1.04114 |
| O | 3.97086 | -0.05951 | 1.50737 |
| C | 3.54637 | 0.68949 | 2.65061 |
| O | 2.51388 | -2.51401 | -2.24368 |
| C | 4.79034 | -1.63646 | -0.79513 |
| C | -0.19156 | -1.99816 | -1.71723 |
| O | -4.59748 | -0.1322 | 0.48199 |
| O | -5.67375 | 0.0086 | -0.48723 |
| H | -3.73125 | -0.97205 | -1.17163 |
| H | -1.07756 | -2.31337 | 1.658 |
| H | -2.42393 | 2.42567 | -1.74376 |
| H | -3.66867 | 1.25019 | -2.20474 |
| H | -3.91532 | 2.28839 | -0.80143 |
| H | -3.07375 | -3.59173 | 1.46526 |
| H | -4.54041 | -2.58713 | 1.36848 |
| H | -3.89027 | -3.32855 | -0.08979 |
| H | -0.95475 | 2.98099 | 1.77484 |
| H | -0.32108 | 4.13158 | 0.58935 |
| H | -1.97968 | 3.53199 | 0.46266 |
| H | 0.71191 | 1.25073 | -1.62666 |
| H | 1.25518 | 3.56325 | -2.45275 |
| H | 1.11556 | 4.21427 | -0.82923 |
| H | 3.51485 | 3.83406 | -1.45947 |
| H | 3.0729 | 2.79434 | -0.09171 |
| H | 3.19022 | 2.11102 | -1.71764 |
| H | 0.025 | -0.4932 | 3.00214 |
| H | 0.26026 | 1.1358 | 2.35197 |
| H | -1.37546 | 0.56819 | 2.7639 |
| H | 2.93385 | 0.07928 | 3.32124 |
| H | 4.46856 | 0.98238 | 3.15369 |
| H | 2.98459 | 1.5809 | 2.35486 |
| H | 5.50932 | -1.17694 | -0.11401 |
| H | 4.94945 | -2.72097 | -0.80328 |
| H | 4.99015 | -1.289 | -1.81506 |
| H | -0.95595 | -1.24068 | -1.90881 |
| H | 0.2656 | -2.30625 | -2.66026 |
| H | -0.69163 | -2.87303 | -1.28671 |
| H | -5.80226 | 0.97391 | -0.46528 |

**Conformer 11b3**

Calculation method: mPW1PW91

Basic Set: 6-31G(d)

E (mPW1PW91): -1232.07213907 a. u.

Boltzmann population: 11.108%

| **Conformer 11b3** | Coordinates (Angstroms) | | |
| --- | --- | --- | --- |
| Atom | X | Y | Z |
| C | -3.2573 | -1.13029 | -0.0597 |
| C | -2.51889 | 0.04833 | -0.66121 |
| C | -1.24547 | 0.32972 | -0.31861 |
| C | -0.47145 | -0.57645 | 0.66485 |
| C | -1.10736 | -1.94745 | 0.79589 |
| C | -2.37272 | -2.22572 | 0.47706 |
| C | -3.32569 | 0.87576 | -1.63444 |
| C | -3.0122 | -3.57122 | 0.68871 |
| C | 0.98876 | -0.75621 | 0.22126 |
| C | -0.54878 | 1.5635 | -0.83715 |
| C | 0.13429 | 1.43679 | -2.18324 |
| C | -0.60304 | 2.70039 | -0.11987 |
| C | -0.00075 | 4.04552 | -0.43532 |
| C | -1.06964 | 5.13799 | -0.6177 |
| C | -0.53973 | 0.06234 | 2.08719 |
| C | 1.47194 | -1.54086 | -0.77145 |
| C | 2.93594 | -1.61098 | -1.01524 |
| C | 3.78007 | -0.76904 | -0.17863 |
| C | 3.17851 | -0.01222 | 0.77397 |
| O | 1.84712 | 0.00481 | 0.99002 |
| O | 3.86799 | 0.78519 | 1.60707 |
| C | 3.15226 | 1.6725 | 2.4736 |
| O | 3.40503 | -2.33659 | -1.89986 |
| C | 5.26514 | -0.78009 | -0.40256 |
| C | 0.61841 | -2.36693 | -1.69768 |
| O | -4.02897 | -0.7437 | 1.1205 |
| O | -5.26621 | -0.10005 | 0.68797 |
| H | -3.98049 | -1.53609 | -0.77888 |
| H | -0.47445 | -2.71905 | 1.23152 |
| H | -2.80258 | 1.78152 | -1.94639 |
| H | -3.56121 | 0.28483 | -2.53112 |
| H | -4.28965 | 1.15865 | -1.19903 |
| H | -2.31726 | -4.27874 | 1.15156 |
| H | -3.89544 | -3.47908 | 1.33171 |
| H | -3.352 | -4.00127 | -0.26351 |
| H | 0.49924 | 2.39554 | -2.55956 |
| H | 0.9827 | 0.74579 | -2.14117 |
| H | -0.56471 | 1.02866 | -2.92373 |
| H | -1.17579 | 2.67872 | 0.80853 |
| H | 0.65972 | 4.33964 | 0.3947 |
| H | 0.63434 | 4.00116 | -1.32555 |
| H | -0.60706 | 6.11719 | -0.78556 |
| H | -1.71503 | 4.91471 | -1.47452 |
| H | -1.71 | 5.21726 | 0.269 |
| H | -0.00835 | -0.55664 | 2.818 |
| H | -0.10202 | 1.06227 | 2.10078 |
| H | -1.59033 | 0.12433 | 2.38092 |
| H | 3.92343 | 2.24158 | 2.99396 |
| H | 2.50852 | 2.34922 | 1.90259 |
| H | 2.54632 | 1.11847 | 3.19647 |
| H | 5.76147 | 0.03209 | 0.13288 |
| H | 5.70158 | -1.73149 | -0.07378 |
| H | 5.47773 | -0.69576 | -1.4732 |
| H | 1.12641 | -2.43161 | -2.66358 |
| H | 0.51284 | -3.39541 | -1.33092 |
| H | -0.38106 | -1.95056 | -1.83061 |
| H | -5.12034 | 0.79449 | 1.04444 |

**Conformer 11b4**

Calculation method: mPW1PW91

Basic Set: 6-31G(d)

E (mPW1PW91): -1232.07247420 a. u.

Boltzmann population: 9.924%

| **Conformer 11b4** | Coordinates (Angstroms) | | |
| --- | --- | --- | --- |
| Atom | X | Y | Z |
| C | -3.3491 | -0.77311 | -0.17801 |
| C | -2.4938 | 0.35002 | -0.72689 |
| C | -1.20618 | 0.4999 | -0.36181 |
| C | -0.54092 | -0.49522 | 0.61568 |
| C | -1.29993 | -1.80617 | 0.69473 |
| C | -2.57898 | -1.96062 | 0.3432 |
| C | -3.24446 | 1.30392 | -1.62302 |
| C | -3.29605 | -3.28237 | 0.44122 |
| C | 0.90904 | -0.79164 | 0.20193 |
| C | -0.38753 | 1.66846 | -0.84931 |
| C | 0.25914 | 1.50336 | -2.20867 |
| C | -0.3156 | 2.78524 | -0.10296 |
| C | 0.4208 | 4.06766 | -0.39335 |
| C | -0.52977 | 5.2707 | -0.52858 |
| C | -0.59021 | 0.11787 | 2.05012 |
| C | 1.34795 | -1.59366 | -0.79741 |
| C | 2.80581 | -1.78667 | -1.00808 |
| C | 3.69834 | -1.03776 | -0.13433 |
| C | 3.14044 | -0.25277 | 0.82208 |
| O | 1.81052 | -0.12559 | 1.00864 |
| O | 3.87535 | 0.46327 | 1.6895 |
| C | 3.2199 | 1.40734 | 2.54389 |
| O | 3.23128 | -2.53311 | -1.89743 |
| C | 5.18224 | -1.17001 | -0.32521 |
| C | 0.45191 | -2.32426 | -1.76286 |
| O | -4.10429 | -0.10834 | 0.88045 |
| O | -5.29396 | -0.90594 | 1.16868 |
| H | -4.08476 | -1.08627 | -0.93053 |
| H | -0.74336 | -2.64603 | 1.10828 |
| H | -2.66304 | 2.19671 | -1.86009 |
| H | -3.52862 | 0.81313 | -2.5645 |
| H | -4.17409 | 1.61766 | -1.13415 |
| H | -2.64776 | -4.06015 | 0.85744 |
| H | -4.19756 | -3.20589 | 1.0587 |
| H | -3.63304 | -3.61603 | -0.55005 |
| H | 0.76269 | 2.41186 | -2.54751 |
| H | 0.99264 | 0.69022 | -2.20836 |
| H | -0.49957 | 1.24061 | -2.95676 |
| H | -0.88134 | 2.80117 | 0.82944 |
| H | 1.12034 | 4.26772 | 0.43286 |
| H | 1.03489 | 3.98211 | -1.29519 |
| H | 0.03135 | 6.19993 | -0.67951 |
| H | -1.20802 | 5.14039 | -1.37919 |
| H | -1.14526 | 5.39226 | 0.37067 |
| H | -0.14149 | -0.56539 | 2.77917 |
| H | -0.06056 | 1.07076 | 2.10242 |
| H | -1.63865 | 0.27538 | 2.3142 |
| H | 4.02526 | 1.9031 | 3.08699 |
| H | 2.65345 | 2.14111 | 1.96173 |
| H | 2.5492 | 0.9062 | 3.24798 |
| H | 5.73057 | -0.39546 | 0.21546 |
| H | 5.53523 | -2.14987 | 0.02015 |
| H | 5.42242 | -1.11211 | -1.3916 |
| H | 0.96315 | -2.38366 | -2.72756 |
| H | 0.27948 | -3.35816 | -1.43893 |
| H | -0.51791 | -1.84076 | -1.8857 |
| H | -5.10516 | -1.15418 | 2.09125 |

**Conformer 11b5**

Calculation method: mPW1PW91

Basic Set: 6-31G(d)

E (mPW1PW91): -1232.07195798 a. u.

Boltzmann population: 9.735%

| **Conformer 11b5** | Coordinates (Angstroms) | | |
| --- | --- | --- | --- |
| Atom | X | Y | Z |
| C | -3.28529 | -0.90886 | -0.01467 |
| C | -2.49564 | 0.20957 | -0.66009 |
| C | -1.22589 | 0.50258 | -0.31308 |
| C | -0.47885 | -0.29532 | 0.77513 |
| C | -1.24601 | -1.51839 | 1.22999 |
| C | -2.51195 | -1.80064 | 0.91839 |
| C | -3.26985 | 0.99819 | -1.69341 |
| C | -3.24378 | -3.00209 | 1.45513 |
| C | 0.90149 | -0.75401 | 0.27342 |
| C | -0.50908 | 1.67714 | -0.93405 |
| C | 0.32659 | 1.39265 | -2.16498 |
| C | -0.69199 | 2.90261 | -0.41008 |
| C | -0.10909 | 4.22024 | -0.8513 |
| C | 0.69066 | 4.91379 | 0.26551 |
| C | -0.33541 | 0.60391 | 2.03968 |
| C | 1.18632 | -1.73261 | -0.61923 |
| C | 2.60176 | -2.04561 | -0.94792 |
| C | 3.6207 | -1.23081 | -0.30005 |
| C | 3.21249 | -0.273 | 0.57053 |
| O | 1.91832 | -0.02537 | 0.86174 |
| O | 4.07459 | 0.51342 | 1.23651 |
| C | 3.5678 | 1.58266 | 2.04233 |
| O | 2.89464 | -2.94488 | -1.74439 |
| C | 5.06602 | -1.49503 | -0.61184 |
| C | 0.15111 | -2.54911 | -1.34753 |
| O | -4.37285 | -0.3575 | 0.79247 |
| O | -5.58263 | -0.32586 | -0.0184 |
| H | -3.77559 | -1.51883 | -0.78526 |
| H | -0.69852 | -2.17815 | 1.90235 |
| H | -2.62329 | 1.63129 | -2.30299 |
| H | -3.83252 | 0.32764 | -2.35343 |
| H | -4.00534 | 1.65974 | -1.21549 |
| H | -2.61543 | -3.57955 | 2.14018 |
| H | -4.15259 | -2.69616 | 1.98456 |
| H | -3.55959 | -3.6668 | 0.63912 |
| H | 0.71101 | 2.30444 | -2.628 |
| H | 1.17955 | 0.74475 | -1.93825 |
| H | -0.27226 | 0.86098 | -2.91555 |
| H | -1.35149 | 2.97969 | 0.45611 |
| H | 0.52681 | 4.10352 | -1.73443 |
| H | -0.93293 | 4.88541 | -1.15195 |
| H | 1.04971 | 5.89676 | -0.05963 |
| H | 0.07553 | 5.06126 | 1.16126 |
| H | 1.56213 | 4.31475 | 0.55512 |
| H | 0.18402 | 0.06808 | 2.84087 |
| H | 0.2122 | 1.5225 | 1.82318 |
| H | -1.33764 | 0.86263 | 2.39214 |
| H | 2.95043 | 1.2047 | 2.86238 |
| H | 4.45309 | 2.0789 | 2.44138 |
| H | 2.98318 | 2.28793 | 1.44279 |
| H | 5.71449 | -0.70351 | -0.23025 |
| H | 5.38924 | -2.44964 | -0.17839 |
| H | 5.19908 | -1.58607 | -1.69466 |
| H | -0.15492 | -3.4246 | -0.76242 |
| H | -0.74529 | -1.97111 | -1.57958 |
| H | 0.60303 | -2.92179 | -2.27002 |
| H | -5.72523 | 0.63576 | -0.07591 |

**Conformer 11b6**

Calculation method: mPW1PW91

Basic Set: 6-31G(d)

E (mPW1PW91): -1232.07245244 a. u.

Boltzmann population: 8.756%

| **Conformer 11b6** | Coordinates (Angstroms) | | |
| --- | --- | --- | --- |
| Atom | X | Y | Z |
| C | -3.50637 | -0.40033 | -0.13004 |
| C | -2.56698 | 0.58534 | -0.79279 |
| C | -1.26079 | 0.64238 | -0.46988 |
| C | -0.66706 | -0.31532 | 0.58818 |
| C | -1.53789 | -1.54163 | 0.79201 |
| C | -2.83409 | -1.60676 | 0.47685 |
| C | -3.24905 | 1.52325 | -1.75799 |
| C | -3.66402 | -2.84514 | 0.69562 |
| C | 0.74553 | -0.7745 | 0.19267 |
| C | -0.34783 | 1.66781 | -1.09313 |
| C | 0.19943 | 1.3165 | -2.46072 |
| C | -0.11336 | 2.8287 | -0.45573 |
| C | 0.76221 | 3.97847 | -0.88164 |
| C | 1.92873 | 4.21696 | 0.09431 |
| C | -0.64083 | 0.42692 | 1.96096 |
| C | 1.09814 | -1.67621 | -0.75423 |
| C | 2.52783 | -2.02347 | -0.95341 |
| C | 3.4927 | -1.33889 | -0.10412 |
| C | 3.02202 | -0.43313 | 0.79033 |
| O | 1.7127 | -0.15253 | 0.95784 |
| O | 3.83181 | 0.28231 | 1.58928 |
| C | 3.26396 | 1.03351 | 2.66854 |
| O | 2.87236 | -2.8562 | -1.80023 |
| C | 4.95252 | -1.65273 | -0.26611 |
| C | 0.12934 | -2.37889 | -1.6679 |
| O | -4.16929 | 0.41895 | 0.88 |
| O | -5.41969 | -0.23731 | 1.25595 |
| H | -4.28746 | -0.70654 | -0.83839 |
| H | -1.04852 | -2.39193 | 1.26508 |
| H | -2.5973 | 2.33974 | -2.0742 |
| H | -3.59045 | 0.98381 | -2.65272 |
| H | -4.13964 | 1.95608 | -1.28771 |
| H | -3.07873 | -3.6418 | 1.16608 |
| H | -4.5416 | -2.63708 | 1.31733 |
| H | -4.05025 | -3.22706 | -0.25963 |
| H | 0.83248 | 2.10437 | -2.8753 |
| H | 0.78672 | 0.39324 | -2.42947 |
| H | -0.6241 | 1.14164 | -3.16498 |
| H | -0.61016 | 2.98386 | 0.50272 |
| H | 1.15955 | 3.82922 | -1.89028 |
| H | 0.14925 | 4.89098 | -0.92421 |
| H | 2.51998 | 5.09048 | -0.20333 |
| H | 1.56204 | 4.39431 | 1.11301 |
| H | 2.5969 | 3.3486 | 0.12299 |
| H | -0.25178 | -0.22758 | 2.7483 |
| H | -0.02046 | 1.32409 | 1.9249 |
| H | -1.66598 | 0.70861 | 2.21252 |
| H | 2.69104 | 0.3885 | 3.34243 |
| H | 4.1188 | 1.45615 | 3.19774 |
| H | 2.62161 | 1.83709 | 2.29774 |
| H | 5.09595 | -2.73767 | -0.29517 |
| H | 5.33055 | -1.26449 | -1.2201 |
| H | 5.5505 | -1.2277 | 0.54285 |
| H | 0.59918 | -2.49435 | -2.64871 |
| H | -0.08433 | -3.39291 | -1.3076 |
| H | -0.81613 | -1.84617 | -1.7709 |
| H | -5.23561 | -0.41425 | 2.1957 |

**Conformer 11b7**

Calculation method: mPW1PW91

Basic Set: 6-31G(d)

E (mPW1PW91): -1232.07180505 a. u.

Boltzmann population: 8.101%

| **Conformer 11b7** | Coordinates (Angstroms) | | |
| --- | --- | --- | --- |
| Atom | X | Y | Z |
| C | -3.45448 | -0.25674 | -0.3704 |
| C | -2.48033 | 0.89984 | -0.28848 |
| C | -1.22072 | 0.73433 | 0.16063 |
| C | -0.69677 | -0.66822 | 0.5474 |
| C | -1.558 | -1.77664 | -0.02661 |
| C | -2.81658 | -1.62072 | -0.44442 |
| C | -3.08431 | 2.23137 | -0.66077 |
| C | -3.63509 | -2.75647 | -1.00178 |
| C | 0.74439 | -0.85814 | 0.05206 |
| C | -0.31359 | 1.91853 | 0.37894 |
| C | -0.49524 | 2.64897 | 1.695 |
| C | 0.55155 | 2.28981 | -0.58035 |
| C | 1.53226 | 3.43271 | -0.58004 |
| C | 1.27679 | 4.4241 | -1.7292 |
| C | -0.77876 | -0.83346 | 2.0976 |
| C | 1.1724 | -1.10421 | -1.20862 |
| C | 2.62243 | -1.27594 | -1.47784 |
| C | 3.52267 | -1.12497 | -0.34258 |
| C | 2.97605 | -0.86781 | 0.87231 |
| O | 1.64999 | -0.7415 | 1.08796 |
| O | 3.71712 | -0.73615 | 1.98604 |
| C | 3.09564 | -0.26187 | 3.18495 |
| O | 3.04034 | -1.516 | -2.61684 |
| C | 5.00111 | -1.26532 | -0.56672 |
| C | 0.27439 | -1.19687 | -2.41423 |
| O | -4.24357 | -0.09015 | 0.8474 |
| O | -5.51129 | -0.79616 | 0.67605 |
| H | -4.1475 | -0.10686 | -1.20851 |
| H | -1.09726 | -2.76348 | -0.04374 |
| H | -2.37253 | 3.05308 | -0.56612 |
| H | -3.43742 | 2.21147 | -1.70107 |
| H | -3.95972 | 2.44085 | -0.03438 |
| H | -3.07884 | -3.69918 | -0.98115 |
| H | -4.5716 | -2.88687 | -0.44879 |
| H | -3.92161 | -2.55382 | -2.04308 |
| H | -0.19848 | 2.03089 | 2.55122 |
| H | 0.08262 | 3.57521 | 1.74075 |
| H | -1.5517 | 2.90043 | 1.84901 |
| H | 0.55859 | 1.70408 | -1.4993 |
| H | 1.52783 | 3.96866 | 0.37487 |
| H | 2.54532 | 3.01887 | -0.6943 |
| H | 2.03801 | 5.21223 | -1.74357 |
| H | 1.30137 | 3.91701 | -2.70087 |
| H | 0.29512 | 4.90035 | -1.62713 |
| H | -0.45686 | -1.83711 | 2.39608 |
| H | -0.15577 | -0.10945 | 2.6253 |
| H | -1.81978 | -0.6952 | 2.39944 |
| H | 3.90465 | -0.1882 | 3.9126 |
| H | 2.64142 | 0.72268 | 3.03246 |
| H | 2.33568 | -0.96235 | 3.54325 |
| H | 5.57584 | -0.94124 | 0.30352 |
| H | 5.2634 | -2.30704 | -0.78944 |
| H | 5.29963 | -0.67804 | -1.44119 |
| H | 0.83343 | -0.84198 | -3.28434 |
| H | -0.00341 | -2.23683 | -2.62643 |
| H | -0.64306 | -0.6182 | -2.30014 |
| H | -5.42286 | -1.46409 | 1.37919 |

**Conformer 11b8**

Calculation method: mPW1PW91

Basic Set: 6-31G(d)

E (mPW1PW91): -1232.07239544 a. u.

Boltzmann population: 7.939%

| **Conformer 11b8** | Coordinates (Angstroms) | | |
| --- | --- | --- | --- |
| Atom | X | Y | Z |
| C | -3.3191 | -0.45001 | -0.33525 |
| C | -2.4246 | 0.77042 | -0.3295 |
| C | -1.19523 | 0.7766 | 0.22311 |
| C | -0.58897 | -0.4817 | 0.87166 |
| C | -1.48782 | -1.6927 | 0.75101 |
| C | -2.72326 | -1.70364 | 0.24667 |
| C | -3.04676 | 1.98245 | -0.98528 |
| C | -3.57449 | -2.94584 | 0.21915 |
| C | 0.7647 | -0.8157 | 0.21442 |
| C | -0.39115 | 2.05063 | 0.30596 |
| C | -0.85493 | 3.03005 | 1.36675 |
| C | 0.63028 | 2.27526 | -0.5393 |
| C | 1.53543 | 3.47586 | -0.63076 |
| C | 1.41684 | 4.19233 | -1.98805 |
| C | -0.4115 | -0.23191 | 2.39781 |
| C | 0.9969 | -1.30763 | -1.02752 |
| C | 2.39231 | -1.58106 | -1.46407 |
| C | 3.45103 | -1.267 | -0.51469 |
| C | 3.09342 | -0.77618 | 0.69841 |
| O | 1.81607 | -0.55784 | 1.07225 |
| O | 3.99205 | -0.47731 | 1.65208 |
| C | 3.55503 | 0.20263 | 2.83363 |
| O | 2.64127 | -2.03753 | -2.58624 |
| C | 4.87792 | -1.50723 | -0.91707 |
| C | -0.0734 | -1.59299 | -2.0489 |
| O | -4.50161 | -0.04707 | 0.42222 |
| O | -5.6606 | -0.74022 | -0.12413 |
| H | -3.67213 | -0.64063 | -1.35955 |
| H | -1.06726 | -2.61235 | 1.15748 |
| H | -2.30534 | 2.75474 | -1.19978 |
| H | -3.52083 | 1.69846 | -1.9343 |
| H | -3.83455 | 2.41544 | -0.35748 |
| H | -3.03467 | -3.80311 | 0.63381 |
| H | -4.49673 | -2.80218 | 0.79274 |
| H | -3.88132 | -3.1932 | -0.80569 |
| H | -0.22827 | 3.92378 | 1.41777 |
| H | -1.88435 | 3.35349 | 1.16846 |
| H | -0.86518 | 2.56909 | 2.36102 |
| H | 0.84034 | 1.50915 | -1.28475 |
| H | 1.34461 | 4.18954 | 0.17725 |
| H | 2.57523 | 3.13848 | -0.50527 |
| H | 2.12535 | 5.02571 | -2.05516 |
| H | 1.62684 | 3.50586 | -2.81653 |
| H | 0.4067 | 4.59122 | -2.1343 |
| H | -0.00613 | -1.12087 | 2.89168 |
| H | 0.26029 | 0.60435 | 2.60094 |
| H | -1.39242 | -0.01627 | 2.83014 |
| H | 4.46374 | 0.37881 | 3.41033 |
| H | 3.07799 | 1.15688 | 2.58794 |
| H | 2.86037 | -0.41275 | 3.41274 |
| H | 5.07811 | -2.58169 | -1.01062 |
| H | 5.06357 | -1.072 | -1.90458 |
| H | 5.57946 | -1.08374 | -0.19527 |
| H | 0.40959 | -1.67149 | -3.02557 |
| H | -0.57805 | -2.54532 | -1.85045 |
| H | -0.83709 | -0.81204 | -2.07562 |
| H | -6.14122 | 0.01662 | -0.50594 |

**Figure S16**. The Cartesian Coordinates of the dominant conformers for **12**

**Conformer 121**

Calculation method: B3LYP

Basic Set: 6-311G(d,p)

E (mPW1PW91): -1082.16740466 a. u.

Boltzmann population: 46.749%

| **Conformer 121** | Coordinates (Angstroms) | | |
| --- | --- | --- | --- |
| Atom | X | Y | Z |
| C | -3.64287 | -1.24847 | -0.30504 |
| C | -3.11418 | -0.00938 | 0.28296 |
| C | -1.78032 | 0.16546 | 0.4624 |
| C | -0.83849 | -1.0408 | 0.17179 |
| C | -1.3891 | -1.91601 | -0.99394 |
| C | -2.87293 | -2.16445 | -0.90836 |
| C | -4.17594 | 0.98684 | 0.69092 |
| C | -3.40902 | -3.40022 | -1.56812 |
| C | 0.57 | -0.59105 | -0.23025 |
| C | -1.20128 | 1.42038 | 1.03303 |
| C | -0.40421 | 1.2976 | 2.31557 |
| C | -1.42677 | 2.59483 | 0.41973 |
| C | -0.98536 | 3.97634 | 0.81921 |
| C | -0.81059 | -1.94157 | 1.43618 |
| C | 0.94681 | 0.19142 | -1.2687 |
| C | 2.37907 | 0.46514 | -1.50507 |
| C | 3.32678 | -0.13295 | -0.58999 |
| C | 2.83776 | -0.90968 | 0.41242 |
| O | 1.52889 | -1.13465 | 0.60132 |
| O | 3.62899 | -1.51907 | 1.29618 |
| C | 3.04615 | -2.2911 | 2.36751 |
| O | 2.74485 | 1.18199 | -2.45148 |
| C | 4.79591 | 0.11201 | -0.7802 |
| C | 0.0012 | 0.82703 | -2.24971 |
| C | -0.05604 | 4.6258 | -0.22172 |
| H | -4.71939 | -1.39254 | -0.26776 |
| H | -1.17616 | -1.46139 | -1.96696 |
| H | -0.84473 | -2.86634 | -0.98687 |
| H | -4.64358 | 1.44668 | -0.18734 |
| H | -4.97467 | 0.47172 | 1.23526 |
| H | -3.78348 | 1.78535 | 1.31693 |
| H | -3.10761 | -3.44134 | -2.62195 |
| H | -3.00564 | -4.30353 | -1.09557 |
| H | -4.49896 | -3.44303 | -1.52083 |
| H | -1.02558 | 0.88087 | 3.11555 |
| H | 0.45229 | 0.62921 | 2.20115 |
| H | -0.02161 | 2.25928 | 2.65486 |
| H | -2.00021 | 2.56682 | -0.50474 |
| H | -1.87893 | 4.60597 | 0.92431 |
| H | -0.49554 | 3.97823 | 1.79481 |
| H | -0.19372 | -2.82767 | 1.27242 |
| H | -0.42978 | -1.41894 | 2.31114 |
| H | -1.82915 | -2.26804 | 1.64882 |
| H | 2.42594 | -1.66081 | 3.00544 |
| H | 2.45678 | -3.11836 | 1.97207 |
| H | 3.89553 | -2.67094 | 2.92909 |
| H | 5.37769 | -0.26819 | 0.05836 |
| H | 5.15728 | -0.36704 | -1.69597 |
| H | 4.98805 | 1.18253 | -0.8885 |
| H | 0.27925 | 1.87301 | -2.39051 |
| H | 0.0916 | 0.34632 | -3.22972 |
| H | -1.03684 | 0.7788 | -1.93325 |
| H | 0.19156 | 5.65211 | 0.06329 |
| H | 0.87787 | 4.06538 | -0.31807 |
| H | -0.52961 | 4.65673 | -1.20741 |

**Conformer 122**

Calculation method: B3LYP

Basic Set: 6-311G(d,p)

E (mPW1PW91): -1082.16704941 a. u.

Boltzmann population: 35.489%

| **Conformer 122** | Coordinates (Angstroms) | | |
| --- | --- | --- | --- |
| Atom | X | Y | Z |
| C | -3.21962 | -1.96668 | -0.23771 |
| C | -2.96277 | -0.58536 | 0.1947 |
| C | -1.69573 | -0.13088 | 0.36533 |
| C | -0.52771 | -1.15382 | 0.23632 |
| C | -0.8581 | -2.25233 | -0.81852 |
| C | -2.26595 | -2.77909 | -0.71338 |
| C | -4.21118 | 0.22428 | 0.46206 |
| C | -2.52787 | -4.16846 | -1.21428 |
| C | 0.78007 | -0.48851 | -0.20495 |
| C | -1.38768 | 1.27183 | 0.78286 |
| C | -0.63465 | 1.45817 | 2.08421 |
| C | -1.79465 | 2.29863 | 0.01743 |
| C | -1.62405 | 3.77667 | 0.24009 |
| C | -0.36813 | -1.87309 | 1.60279 |
| C | 1.03342 | 0.22332 | -1.32808 |
| C | 2.3945 | 0.73498 | -1.58717 |
| C | 3.41023 | 0.44249 | -0.59942 |
| C | 3.04666 | -0.28792 | 0.48771 |
| O | 1.7983 | -0.73375 | 0.69474 |
| O | 3.91198 | -0.62494 | 1.44475 |
| C | 3.45548 | -1.3657 | 2.59649 |
| O | 2.64655 | 1.38821 | -2.61333 |
| C | 4.81263 | 0.93564 | -0.81067 |
| C | 0.01558 | 0.54502 | -2.38662 |
| C | -2.97045 | 4.51436 | 0.35349 |
| H | -4.24798 | -2.31515 | -0.19155 |
| H | -0.71003 | -1.88052 | -1.83775 |
| H | -0.13693 | -3.06685 | -0.693 |
| H | -4.00351 | 1.15502 | 0.98541 |
| H | -4.72815 | 0.47141 | -0.47236 |
| H | -4.91317 | -0.36739 | 1.05932 |
| H | -2.19195 | -4.27888 | -2.25264 |
| H | -1.9698 | -4.90841 | -0.62853 |
| H | -3.58904 | -4.421 | -1.16798 |
| H | -1.17035 | 0.97505 | 2.90784 |
| H | 0.36239 | 1.01157 | 2.04919 |
| H | -0.51018 | 2.51003 | 2.33779 |
| H | -2.32762 | 2.05129 | -0.89858 |
| H | -1.0201 | 3.98544 | 1.12511 |
| H | -1.07259 | 4.19406 | -0.61248 |
| H | 0.40847 | -2.63934 | 1.55375 |
| H | -0.11676 | -1.18541 | 2.40754 |
| H | -1.31316 | -2.35832 | 1.84916 |
| H | 2.70087 | -0.80176 | 3.14537 |
| H | 3.05552 | -2.33526 | 2.29927 |
| H | 4.34285 | -1.49878 | 3.20965 |
| H | 5.42184 | 0.80832 | 0.08319 |
| H | 5.29649 | 0.40159 | -1.63521 |
| H | 4.80037 | 1.99321 | -1.08532 |
| H | 0.2285 | -0.01827 | -3.30149 |
| H | -1.00347 | 0.33233 | -2.07634 |
| H | 0.09275 | 1.60175 | -2.64963 |
| H | -2.81747 | 5.59325 | 0.44492 |
| H | -3.59202 | 4.33779 | -0.52932 |
| H | -3.53056 | 4.17574 | 1.22948 |

**Conformer 123**

Calculation method: B3LYP

Basic Set: 6-311G(d,p)

E (mPW1PW91): -1082.16466677 a. u.

Boltzmann population: 8.371%

| **Conformer 123** | Coordinates (Angstroms) | | |
| --- | --- | --- | --- |
| Atom | X | Y | Z |
| C | -3.52246 | -1.45488 | 0.23791 |
| C | -3.01045 | -0.1946 | -0.31878 |
| C | -1.71451 | 0.15879 | -0.17619 |
| C | -0.76596 | -0.75105 | 0.65066 |
| C | -1.23484 | -2.23895 | 0.67019 |
| C | -2.73031 | -2.4185 | 0.72604 |
| C | -4.04045 | 0.66711 | -1.00937 |
| C | -3.24318 | -3.72379 | 1.2599 |
| C | 0.66381 | -0.71265 | 0.09596 |
| C | -1.19353 | 1.4792 | -0.68307 |
| C | -0.80887 | 1.539 | -2.14749 |
| C | -1.12737 | 2.53657 | 0.14121 |
| C | -0.64004 | 3.92983 | -0.15545 |
| C | -0.80712 | -0.24952 | 2.11757 |
| C | 1.12472 | -1.17058 | -1.09278 |
| C | 2.56614 | -1.0869 | -1.40779 |
| C | 3.42203 | -0.4667 | -0.41982 |
| C | 2.84924 | -0.03401 | 0.7347 |
| O | 1.53934 | -0.14787 | 0.99792 |
| O | 3.55448 | 0.53627 | 1.71211 |
| C | 2.876 | 1.04648 | 2.87983 |
| O | 3.01237 | -1.52379 | -2.48168 |
| C | 4.89287 | -0.3329 | -0.6906 |
| C | 0.26401 | -1.77594 | -2.16945 |
| C | -1.74209 | 4.98899 | 0.01872 |
| H | -4.59994 | -1.59786 | 0.22815 |
| H | -0.86923 | -2.78935 | -0.20114 |
| H | -0.76638 | -2.724 | 1.53338 |
| H | -3.63088 | 1.61344 | -1.35841 |
| H | -4.47391 | 0.13806 | -1.86556 |
| H | -4.86874 | 0.88787 | -0.32665 |
| H | -2.8056 | -4.56831 | 0.71359 |
| H | -2.96069 | -3.85546 | 2.31099 |
| H | -4.33015 | -3.79213 | 1.18353 |
| H | 0.12692 | 1.00697 | -2.33719 |
| H | -1.57595 | 1.05686 | -2.76004 |
| H | -0.68817 | 2.56244 | -2.50342 |
| H | -1.48503 | 2.40277 | 1.15989 |
| H | 0.1793 | 4.16428 | 0.5365 |
| H | -0.21795 | 4.00032 | -1.15982 |
| H | -0.16726 | -0.86272 | 2.75652 |
| H | -0.48416 | 0.78548 | 2.20757 |
| H | -1.83311 | -0.32805 | 2.48131 |
| H | 2.15636 | 1.81704 | 2.60214 |
| H | 2.37454 | 0.24209 | 3.41796 |
| H | 3.66436 | 1.4737 | 3.49388 |
| H | 5.37317 | 0.33377 | 0.02452 |
| H | 5.39293 | -1.30602 | -0.64043 |
| H | 5.05349 | 0.05172 | -1.70061 |
| H | 0.60391 | -1.4098 | -3.14001 |
| H | 0.37797 | -2.86493 | -2.19421 |
| H | -0.7911 | -1.54043 | -2.05243 |
| H | -1.34596 | 5.9957 | -0.14035 |
| H | -2.55594 | 4.82809 | -0.69371 |
| H | -2.1681 | 4.95168 | 1.02578 |

**Conformer 124**

Calculation method: B3LYP

Basic Set: 6-311G(d,p)

E (mPW1PW91): -1082.16434772 a. u.

Boltzmann population: 7.572%

| **Conformer 124** | Coordinates (Angstroms) | | |
| --- | --- | --- | --- |
| Atom | X | Y | Z |
| C | -3.81652 | -0.72952 | 0.31393 |
| C | -3.10073 | 0.37415 | -0.34175 |
| C | -1.76041 | 0.50397 | -0.23235 |
| C | -0.97301 | -0.49133 | 0.66248 |
| C | -1.69502 | -1.86758 | 0.80079 |
| C | -3.19763 | -1.77705 | 0.87424 |
| C | -3.97567 | 1.3495 | -1.09259 |
| C | -3.92261 | -2.92752 | 1.50866 |
| C | 0.43231 | -0.75243 | 0.10669 |
| C | -1.03025 | 1.67451 | -0.84206 |
| C | -0.66308 | 1.55459 | -2.30666 |
| C | -0.78404 | 2.76867 | -0.10399 |
| C | -0.0767 | 4.03676 | -0.50189 |
| C | -0.90419 | 0.12378 | 2.08425 |
| C | 0.78454 | -1.37199 | -1.04542 |
| C | 2.21214 | -1.57827 | -1.36543 |
| C | 3.18079 | -1.07313 | -0.4173 |
| C | 2.71379 | -0.4561 | 0.70039 |
| O | 1.40896 | -0.29677 | 0.96562 |
| O | 3.52449 | 0.04448 | 1.63336 |
| C | 2.96478 | 0.66064 | 2.81294 |
| O | 2.55681 | -2.16002 | -2.40745 |
| C | 4.64621 | -1.25002 | -0.69257 |
| C | -0.18783 | -1.89085 | -2.07108 |
| C | 1.15782 | 4.31991 | 0.3722 |
| H | -4.90243 | -0.6812 | 0.31766 |
| H | -1.44506 | -2.53847 | -0.02582 |
| H | -1.30422 | -2.35916 | 1.69822 |
| H | -4.51865 | 0.84036 | -1.89674 |
| H | -4.73333 | 1.77179 | -0.42281 |
| H | -3.41111 | 2.1743 | -1.52362 |
| H | -3.64945 | -3.8744 | 1.02703 |
| H | -3.64998 | -3.02804 | 2.56576 |
| H | -5.0058 | -2.8085 | 1.44142 |
| H | -0.32501 | 2.50053 | -2.72947 |
| H | 0.12923 | 0.81784 | -2.45959 |
| H | -1.5258 | 1.2149 | -2.88749 |
| H | -1.13628 | 2.76849 | 0.92536 |
| H | 0.22174 | 4.01787 | -1.55161 |
| H | -0.77706 | 4.87539 | -0.39367 |
| H | -0.37278 | -0.54008 | 2.77008 |
| H | -0.40329 | 1.08957 | 2.09002 |
| H | -1.92299 | 0.25573 | 2.45286 |
| H | 3.82614 | 0.96884 | 3.39962 |
| H | 2.36146 | 1.52859 | 2.54665 |
| H | 2.36419 | -0.05523 | 3.37449 |
| H | 5.25815 | -0.74975 | 0.05653 |
| H | 4.91294 | -2.31149 | -0.70582 |
| H | 4.89909 | -0.85027 | -1.67847 |
| H | -0.27618 | -2.98077 | -2.00773 |
| H | -1.18035 | -1.45708 | -1.97385 |
| H | 0.2006 | -1.6724 | -3.06749 |
| H | 1.6181 | 5.2736 | 0.09968 |
| H | 0.8884 | 4.36871 | 1.43145 |
| H | 1.90956 | 3.53456 | 0.25419 |

**Figure S17a**. The HPLC analysis of residue (80%-100% CH_3_CN/H_2_0, 10 min, 3.0 mL/min), which was obtained after the treatment of Methylene Blue with compound **6** in CH_2_Cl_2_ at room temperature and exposed to sunlight for 2 hours.

**Figure S17b**. The HPLC analysis of residue (80%-100% CH_3_CN/H_2_0, 10 min, 3.0 mL/min), which was obtained after the treatment of Methylene Blue and Rose Bengal with compound **8** in CH_2_Cl_2_ at room temperature and exposed to sunlight for 4 hours.

**Figure S17c**. The HPLC analysis of residue (80%-100% CH_3_CN/H_2_0, 10 min, 3.0 mL/min), which was obtained after the treatment of trifluoroacetic acid (TFA) and/or silica gel with compound **8** in CH_2_Cl_2_ at room temperature overnight.

**Table S2**. The antibacterial activity assays.

|  | MIC (μg/mL) | | | | | |
| --- | --- | --- | --- | --- | --- | --- |
| Compounds | *Staphylococcus aureus*  ATCC27154 | *Streptococcus parauberis*  FP KSP28 | *Lactococcus garvieae FP*  MP5245 | *Aeromonas salmonicida*  AS42 | *pseudomonas aeruginosa*  ATCC10145 | *Photobacterium halotolerans*  LMG 22194T |
| tetracycline | 0.750204 | 3.005625 | 0.750204 | 6.1125 |  |  |
| Oxytetracycline hydrochloride | ＜0.193 | 1.552 | ＜0.193 | 0.387 |  |  |
| Levofloxacin Hydrochloride | ＜0.155 | 1.24 | 4.97 | ＜0.155 |  |  |
| Ampicillin sodium | ＜0.145 | 4.642 | 0.29 | ＞18.57 |  |  |
| **2** | >33.022 | >33.022 | >33.022 | >33.022 | >33.022 | >33.022 |
| **3** | >34.222 | >34.222 | >34.222 | >34.222 | >34.222 | >34.222 |
| **4** | >37.421 | >37.421 | >37.421 | >37.421 | >37.421 | >37.421 |
| **5** | >37.421 | >37.421 | >37.421 | >37.421 | >37.421 | >37.421 |
| **7** | >35.821 | 35.821 | >35.821 | >35.821 | >35.821 | >35.821 |
| **8** | >34.222 | 34.222 | >34.222 | >34.222 | >34.222 | >34.222 |
| **9** | >37.421 | >37.421 | >37.421 | >37.421 | >37.421 | >37.421 |
| **11** | >37.421 | 37.421 | >37.421 | >37.421 | >37.421 | >37.421 |

**Table S3**. Neuroprotective effect of tested compounds on hydrogen peroxide (H_2_O_2_) induced SH-SY5Y cell damage.

| **Groups** | | **Cell viability ± SEM**  **(% compared with control group)** | | |
| --- | --- | --- | --- | --- |
| Control | | 100.00 ± 0.00 | | |
| Model - H_2_O_2_ (100 μmol/L) | | 35.63 ± 10.85 | | |
| Positive control- NAC (500μmol/L) | | 53.51 ± 8.74 | | |
| Compounds | Final concentration (μmol/L) | **Cell viability ± SEM**  **(% compared with control group)** | | |
| **1** | 1 | 36.66 | ± | 7.35 |
|  | 5 | 32.52 | ± | 6.84 |
|  | 10 | 32.85 | ± | 6.41 |
|  | 20 | 28.97 | ± | 6.99 |
| **2** | 1 | 34.55 | ± | 7.13 |
|  | 5 | 38.57 | ± | 9.63 |
|  | 10 | 36.21 | ± | 9.01 |
|  | 20 | 32.14 | ± | 6.75 |
| **3** | 1 | 32.26 | ± | 5.46 |
|  | 5 | 34.63 | ± | 6.89 |
|  | 10 | 34.33 | ± | 8.87 |
|  | 20 | 34.35 | ± | 6.90 |
| **4** | 1 | 31.91 | ± | 8.53 |
|  | 5 | 33.81 | ± | 10.29 |
|  | 10 | 32.76 | ± | 6.75 |
|  | 20 | 34.51 | ± | 6.28 |
| **5** | 1 | 36.04 | ± | 4.81 |
|  | 5 | 32.01 | ± | 4.12 |
|  | 10 | 35.04 | ± | 5.67 |
|  | 20 | 36.41 | ± | 5.09 |
| **6** | 1 | 31.65 | ± | 8.60 |
|  | 5 | 32.39 | ± | 7.25 |
|  | 10 | 39.42 | ± | 16.67 |
|  | 20 | 32.39 | ± | 5.73 |
| **7** | 1 | 30.90 | ± | 4.95 |
|  | 5 | 30.73 | ± | 3.28 |
|  | 10 | 32.76 | ± | 5.52 |
|  | 20 | 31.65 | ± | 4.39 |
| **8** | 1 | 38.09 | ± | 7.40 |
|  | 5 | 36.92 | ± | 8.22 |
|  | 10 | 40.43 | ± | 8.84 |
|  | 20 | 38.54 | ± | 6.26 |
| **9** | 1 | 30.41 | ± | 4.70 |
|  | 5 | 35.08 | ± | 8.42 |
|  | 10 | 38.35 | ± | 10.25 |
|  | 20 | 37.31 | ± | 7.91 |
| **10** | 1 | 35.45 | ± | 8.01 |
|  | 5 | 34.40 | ± | 4.01 |
|  | 10 | 33.01 | ± | 3.80 |
|  | 20 | 36.76 | ± | 5.47 |
| **11** | 1 | 31.24 | ± | 4.81 |
|  | 5 | 33.61 | ± | 5.00 |
|  | 10 | 35.98 | ± | 5.63 |
|  | 20 | 35.76 | ± | 3.10 |
| **12** | 1 | 26.89 | ± | 7.10 |
|  | 5 | 26.89 | ± | 6.69 |
|  | 10 | 28.62 | ± | 5.90 |
|  | 20 | 27.13 | ± | 5.36 |

**Table S4**. The protein tyrosine phosphatase 1B (PTP1B) inhibitory activity assay.

| Compounds | Protocol id | Concentration | type | unit | Results | error |
| --- | --- | --- | --- | --- | --- | --- |
| Oleanolic acid | 25 |  | IC_50_ | μg/mL | 2.53 | 0.20 |
| **1** | 25 | 20μmol/L | % Inhibition | percent | / | / |
| **2** | 25 | 20μmol/L | % Inhibition | percent | -0.44 | 0.40 |
| **3** | 25 | 20μmol/L | % Inhibition | percent | 3.53 | 1.26 |
| **4** | 25 | 20μmol/L | % Inhibition | percent | 17.80 | 14.60 |
| **5** | 25 | 20μmol/L | % Inhibition | percent | 19.04 | 1.79 |
| **6** | 25 | 20μmol/L | % Inhibition | percent | 36.83 | 0.62 |
| **7** | 25 | 20μmol/L | % Inhibition | percent | 8.77 | 12.24 |
| **8** | 25 | 20μmol/L | % Inhibition | percent | -4.39 | 1.65 |
| **9** | 25 | 20μmol/L | % Inhibition | percent | 4.72 | 8.45 |
| **10** | 25 | 20μmol/L | % Inhibition | percent | / | / |
| **11** | 25 | 20μmol/L | % Inhibition | percent | -2.44 | 2.74 |
| **12** | 25 | 20μmol/L | % Inhibition | percent | 3.00 | 0.24 |

**Table S5**. The antiviral activity against 2019-nCoV RDRPro.

| Compounds | Protocol id | Concentration | type | unit | Results | error |
| --- | --- | --- | --- | --- | --- | --- |
| suramin | 207 |  | IC_50_ | nmol/L | 267.30 | 9.05 |
| **1** | 207 | 20μmol/L | % Inhibition | percent | / | / |
| **2** | 207 | 20μmol/L | % Inhibition | percent | 2.70 | 1.80 |
| **3** | 207 | 20μmol/L | % Inhibition | percent | -4.76 | 2.08 |
| **4** | 207 | 20μmol/L | % Inhibition | percent | 14.95 | 0.42 |
| **5** | 207 | 20μmol/L | % Inhibition | percent | 0.79 | 0.66 |
| **6** | 207 | 20μmol/L | % Inhibition | percent | 3.67 | 1.24 |
| **7** | 207 | 20μmol/L | % Inhibition | percent | 0.13 | 2.01 |
| **8** | 207 | 20μmol/L | % Inhibition | percent | 0.95 | 1.66 |
| **9** | 207 | 20μmol/L | % Inhibition | percent | -1.57 | 4.98 |
| **10** | 207 | 20μmol/L | % Inhibition | percent | / | / |
| **11** | 207 | 20μmol/L | % Inhibition | percent | -1.04 | 3.67 |
| **12** | 207 | 20μmol/L | % Inhibition | percent | 0.81 | 0.98 |

**Table S6**. The ameliorative effect of tested compounds on lipopolysaccharide (LPS) induced cellular inflammation.

| Compounds | NO Inhibition rate % | | |  | RAW264.7 Cell viability % | | |
| --- | --- | --- | --- | --- | --- | --- | --- |
|  | 20 μmol/L | 10μmol/L | 5μmol/L |  | 20μmol/L | 10μmol/L | 5μmol/L |
| **2** | -3.17 | -13.87 | -20.74 |  | 98.77 | 97.54 | 103.01 |
| **3** | 6.61 | 12.13 | 10.94 |  | 99.64 | 96.87 | 100.28 |
| **5** | 6.8 | 1.62 | 1.83 |  | 98.67 | 95.04 | 95.78 |
| **7** | 4.24 | -4.53 | -11.8 |  | 98.76 | 97.11 | 95.53 |
| **9** | -4.16 | -2.19 | -2.57 |  | 97.76 | 97.01 | 99.76 |
| **11** | -0.82 | -2.21 | -5.1 |  | 94.55 | 97.84 | 99.12 |
